# Palladium-catalyzed allene synthesis enabled by *β*-hydrogen elimination from *sp*^2^*-*carbon

**DOI:** 10.1038/s41467-020-20740-w

**Published:** 2021-02-01

**Authors:** Ge Zhang, Yi-Kang Song, Fang Zhang, Ze-Jian Xue, Meng-Yao Li, Gui-Shan Zhang, Bin-Bin Zhu, Jing Wei, Chunsen Li, Chen-Guo Feng, Guo-Qiang Lin

**Affiliations:** 1 The Research Center of Chiral Drugs, Innovation Research Institute of Traditional Chinese Medicine, Shanghai University of Traditional Chinese Medicine, 1200 Cailun Road, Shanghai, 201203 China; 2 CAS Key Laboratory of Synthetic Chemistry of Natural Substances, Center for Excellence in Molecular Synthesis, Shanghai Institute of Organic Chemistry, University of Chinese Academy of Sciences, Chinese Academy of Sciences, 345 Lingling Road, Shanghai, 200032 China; 3 School of Physical Science and Technology, ShanghaiTech University, 393 Huaxia Road, Shanghai, 201210 China; 4 State Key Laboratory of Structural Chemistry, Fujian Institute of Research on the Structure of Matter, Chinese Academy of Sciences, 155 West Yangqiao Road, Fuzhou, 350002 China

**Keywords:** Catalytic mechanisms, Homogeneous catalysis, Synthetic chemistry methodology

## Abstract

The rational design based on a deep understanding of the present reaction mechanism is an important, viable approach to discover new organic transformations. *β*-Hydrogen elimination from palladium complexes is a fundamental reaction in palladium catalysis. Normally, the eliminated *β-*hydrogen has to be attached to a *sp*^*3*^-carbon. We envision that the hydrogen elimination from *sp*^*2*^-carbon is possible by using thoroughly designed reaction systems, which may offer a new strategy for the preparation of allenes. Here, we describe a palladium-catalyzed cross-coupling of 2,2-diarylvinyl bromides and diazo compounds, where a *β*-vinylic hydrogen elimination from allylic palladium intermediate is proposed to be the key step. Both aryl diazo carbonyl compounds and *N*-tosylhydrazones are competent carbene precursors in this reaction. The reaction mechanism is explored by control experiments, KIE studies and DFT calculations.

## Abstract (web summary)

*β*-Hydrogen elimination is a fundamental reaction in palladium catalysis, however, the eliminated *β*-hydrogen is usually attached to a *sp*^*3*^-carbon. Here, the authors report a palladium-catalyzed cross-coupling reaction involving a *β*-vinylic hydrogen elimination from an allylic palladium intermediate.

## Introduction

**Fig. 1:**
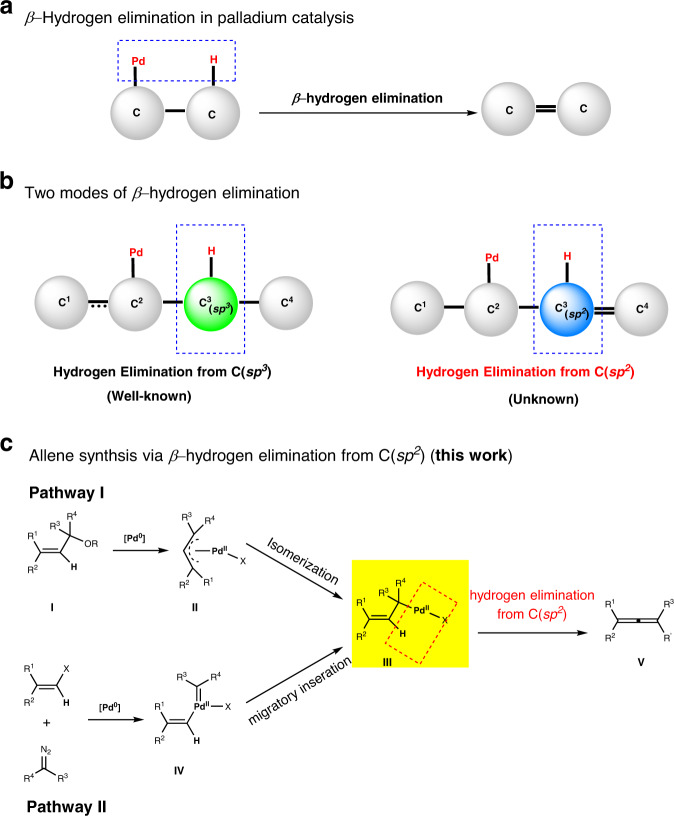
Allene synthesis based on *β*-hydrogen elimination from *sp*^2^ hybrid carbon. **a** *β*-Hydrogen elimination in palladium catalysis. **b** Two modes of *β*-hydrogen elimination. **c** Allene synthesis via *β*-hydrogen elimination from C(*sp*^2^) (this work).

Palladium catalysis has proved to be a powerful synthetic tool, which is demonstrated by numerous useful transformations and highlighted by the 2010 Nobel Prize in chemistry^1–3^. Although the mechanism involved in those reactions have been extensively explored, efforts to acquire a deep understanding of the current mechanistic hypothesis and apply them to design new transformations have never ceased. As an elementary reaction in palladium catalysis, *β*-hydrogen elimination has been well studied (Fig. 1a)^4–7^. Theoretically, the hydrogen elimination can be divided into two categories according to the hybrid state of the attached carbon atom. Hydrogen elimination from *sp*^3^-carbon is the most common pattern, and both alkyl and alkenyl palladium complexes^8–11^ can undergo this elimination pathway, affording olefins and allenes respectively. In contrast, the second hydrogen mode, where the eliminated hydrogen is attached to a *sp*^2^-carbon (also means *β*-elimination of vinylic hydrogen from ^1^*η*-*δ*-allylic palladium) and allene would be generated, has not been reported yet (Fig. 1b).

On the other hand, allenes are of great importance due to their wide existence in natural products^12^, pharmaceuticals^13^, and molecular materials^14^. The active nature imparted by its unique orthogonal cumulative *π*-system also makes them highly versatile and useful building blocks in organic synthesis^15–19^. Although numerous methods for the preparation of allenes have been developed^20–24^, they still lag far behind the growing demand in the application. At present, the majority of the existing methodologies rely on the utilization of elaborate alkynes. Therefore, it is highly desirable to develop new approaches via new mechanistic pathways, which may deliver the allenes efficiently from easily accessible starting materials and complement the current methodologies^25–33^. Therefore, *β*-hydrogen elimination of allylic palladium from *sp*^*2*^-carbon represents an attractive new strategy for allene synthesis.

Here, we report the successful application of the *β*-hydrogen elimination from *sp*^2^-carbon for the allene synthesis. In our research plan, the desired *δ*-allylic palladium intermediate is planned to be produced from the classic allylic alcohol derivatives, which can undergo an oxidative addition/isomerization sequence in the presence of Pd^0^ (Fig. 1c, pathway I). A second pathway was also devised where the cross-coupling of alkenyl halides and diazo compounds offer the desired *δ*-allylic palladium intermediate via migratory of palladium carbene **IV** (Fig. 1c, pathway II)^34–37^. In these two pathways, there is an equilibrium of ^1^*η*-*δ*- and ^3^*η-π*-allylic palladium intermediates. The *π*-allylic palladium normally showed higher stability compared with the corresponding *δ*-allylic one. However, The lack of a *syn* coplanar arrangement of C–H and C-Pd bonds, a key factor for most *β*-hydrogen elimination, would make the hydrogen elimination from *π*-allylic palladium rather difficult^38–40^.

## Results

### Initial study

**Fig. 2:**
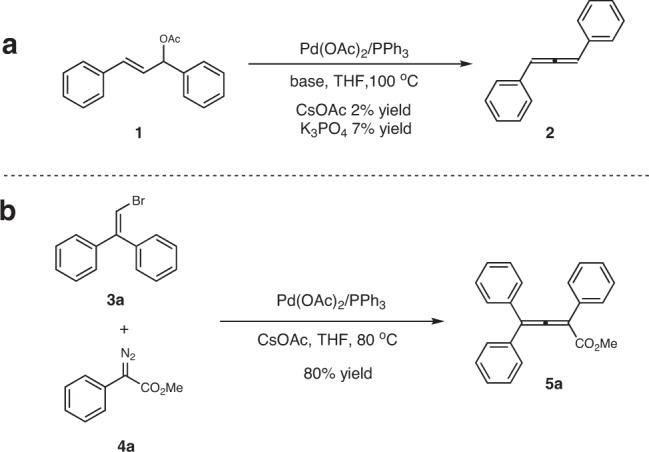
Initial studies. **a** Allene synthesis starting from allyl acetate **1**. **b** Allene synthesis starting from vinylbromide **3a** and diazoacetate **4a**.

With these considerations in mind, we set out to explore the feasibility of the planned strategy. A small amount of allene **2** was observed when allyl acetate **1** was treated by Pd(OAc)_2_/PPh_3_ at 100 ^o^C with poor conversion (Fig. 2a). However, attempts to further improve this reaction were unsuccessful, and a complicated mixture was observed when a full conversion was achieved by changing ligands or solvents. Next, we tested the cross-coupling of 2,2-diarylvinyl bromide **3a** and diazoacetate **4a** in the presence of Pd(OAc)_2_/PPh_3_ (Fig. 2b). These two model substrates were selected because the planned elimination is expected to be promoted by the generation of stable multi-aryl substituted allenes, and the competitive elimination from a *sp*^3^-carbon will be avoided. Delightfully, the desired allene **5a** was generated in high yield, and its structure was unambiguously confirmed by X-ray analysis.

### Reaction conditions development

Encouraged by the above results, more reaction conditions were screened for a higher reaction yield (Table 1). Other mono-phosphine ligands, with either electron-withdrawing fluorine (L2) or electron-donating MeO group (L3), gave reduced reaction yields (entries 2 and 3). Bis-phosphine ligands were also competent to promote this reaction, and the ligand bearing a linkage of six carbon atoms further improved the reaction yield to 87% (entries 4–7). Instead of CsOAc, several other bases were also examined, but offered inferior results (entries 8–10). The reaction also went well in other ether solvents, but was rather sluggish with toluene or DCE as solvent (entries 13 and 14). While a comparable result was obtained in an elevated reaction temperature of 90 ^o^C (entry 15), an obvious loss in reaction yield was observed at a lower temperature (entries 16 and 17).

**Table 1:** Optimization of reaction conditions.


Entry	Ligand	T (^o^C)	Solvent	Base	Yield (%)^a^
1	L1	80	THF	CsOAc	80
2	L2	80	THF	CsOAc	60
3	L3	80	THF	CsOAc	66
4	L4	80	THF	CsOAc	77
5	L5	80	THF	CsOAc	82
6	L6	80	THF	CsOAc	63
7	L7	80	THF	CsOAc	87
8	L7	80	THF	CsOPiv	65
9	L7	80	THF	Cs_2_CO_3_	66
10	L7	80	THF	K_2_CO_3_	77
11	L7	80	1,4-Dioxane	CsOAc	82
12	L7	80	TBME	CsOAc	70
13	L7	80	Toluene	CsOAc	10
14	L7	80	DCE	CsOAc	37
15	L7	90	THF	CsOAc	86
16	L7	70	THF	CsOAc	78
17	L7	60	THF	CsOAc	20

Reaction conditions: **3a** (0.20 mmol), **4a** (0.30 mmol, 1.5 equiv), Pd(OAc)_2_ (0.02 mmol, 0.1 equiv), ligand (0.06 mmol for L1–L3 or 0.03 mmol for L4–L7), CsOAc (0.30 mmol, 1.5 equiv), THF (2 mL).

*THF* tetrahydrofuran, *TBME* tert-butyl methyl ether, *DCE* 1,2-dichloroethane.

^a^Determined by ^1^H NMR spectroscopy using CH_2_Br_2_ as an internal standard.

### Substrate scope of 2,2-diarylvinyl bromides with diazo carbonyl compounds

**Fig. 3:**
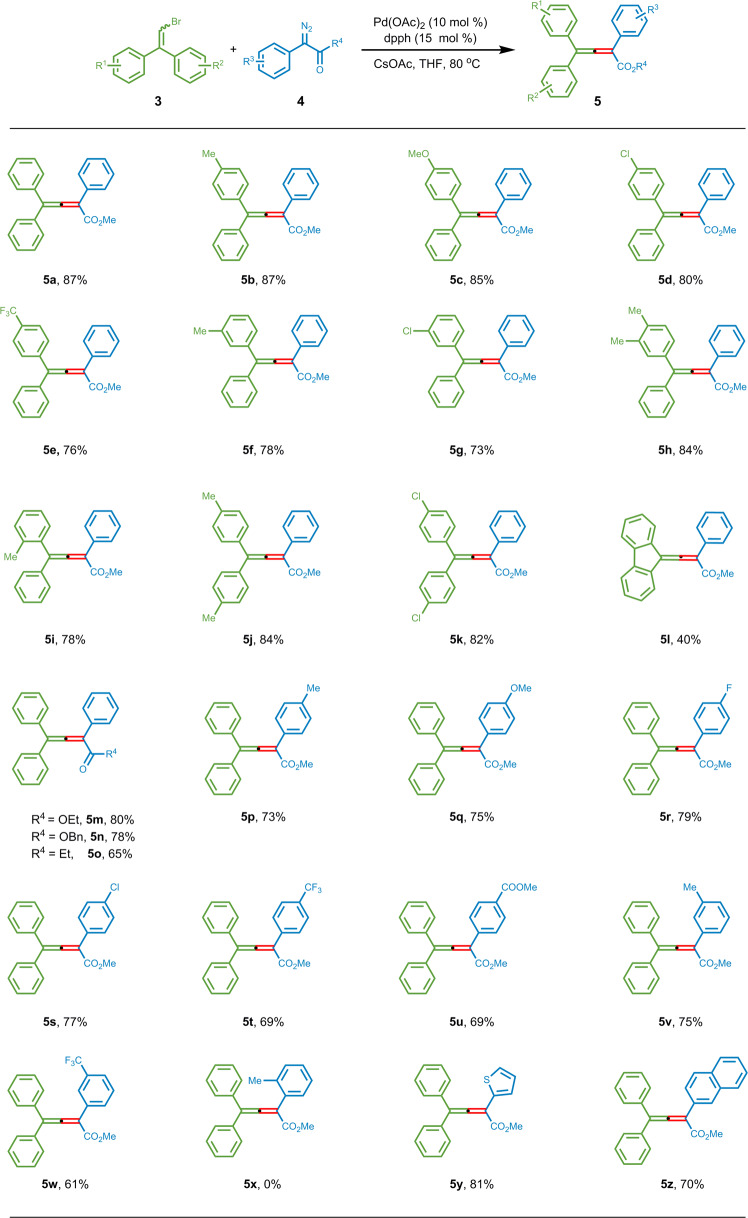
Cross-coupling of 2,2-diarylvinyl bromides with diazo carbonyl compounds. ^a^Reactions conditions: **3** (0.20 mmol), **4** (0.30 mmol, 1.5 equiv), Pd(OAc)_2_ (0.02 mmol, 0.1 equiv), dpph (0.03 mmol, 0.15 equiv), CsOAc (0.30 mmol, 1.5 equiv), THF (2 mL), 80 ^o^C. Isolated yields.

With the optimal reaction conditions in hand, we began to explore the generality of this cross-coupling reaction (Fig. 3). First, a variety of 2,2-diarylvinyl bromides **3** were used in the coupling with phenyl diazoacetate **4a**. All of them afforded high yields, with a deleterious effect on the reaction outcome by introducing electron-withdrawing groups to the phenyl ring, or moving the substituents from *para*- to *meta-* or *ortho-* position (**5d–i**). Vinyl bromide with a flat terminal fluorene substitution, instead of two separate aryl groups, also proceeded well (**5l**). Next, the variation of the aryl diazoacetates **4** was also investigated. The methyl ester could be successfully replaced by an ethyl or benzyl ester, as well as an ethyl ketone (**5m–o**). Introduction of different substituents onto the *para-* or *meta-* position of the phenyl ring was well tolerated, albeit in slightly reduced reaction yields (**5p–w**). Compared with the vinyl bromide substrates, the diazoacetate part was more sensitive to the steric properties, as the *ortho-*methyl substituted phenyl ring completely blocked the coupling reaction (**5x**). Delightfully, other aromatic rings, like 2-thienyl or naphthyl group, could provide the desired products in good yields (**5y** and **5z**).

### Substrate scope of 2,2-diarylvinyl bromides with *N*-tosylhydrazones

**Fig. 4:**
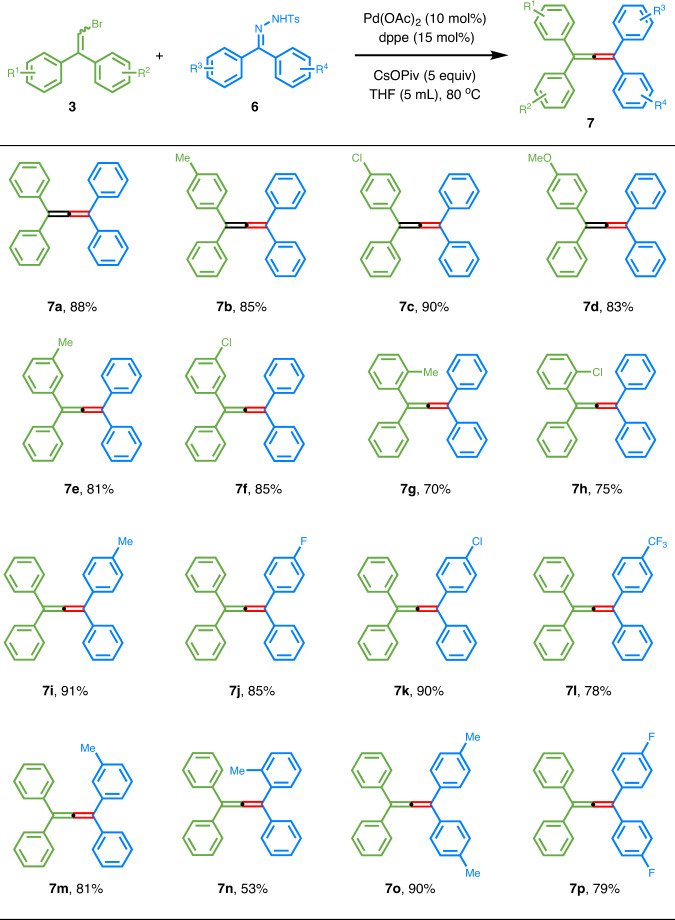
Cross-coupling of 2,2-diarylvinyl bromides with *N*-tosylhydrazones. ^a^Reaction conditions: **3** (0.20 mmol), **6** (0.30 mmol, 1.5 equiv.), Pd(OAc)_2_ (0.02 mmol, 0.1 equiv.), dppe (0.03 mmol, 0.15 equiv.), CsOPiv (1.00 mmol, 5 equiv.), THF (5 mL), 80 ^o^C. Isolated yields.

Encouraged by the above success, we sought to use diaryldiazomethanes to produce tetra-aryl-substituted allenes, which showed some unique properties in material science^41^, catalysis^42,43^, and molecular recognition^44,45^. Although a preliminary experiment with diphenyldiazomethane furnished the tetra-phenyl-substituted allene **7a** in moderate reaction yield under the standard reaction conditions, further efforts were hampered by the relatively lower stability of this kind of diazo compounds. Therefore, we switched to the corresponding *N*-tosylhydrazones **6**, a family of stable carbene precursors^46–48^. Gratifyingly, the slight adjustment of the base and ligand to cesium pivalate and dppe could lead to the desired cross-coupling products in good to excellent yields (Fig. 4). While electronic variation on the phenyl ring of the 2,2-diarylvinyl bromides showed marginal effect on the reaction outcome (**7a–h**), the introduction of electron-withdrawing groups to the diaryl ketones derived *N*-tosylhydrazones gave slightly reduced yields (**7j–l**). *Ortho*-substituted phenyl rings on either vinyl bromides or *N*-tosylhydrazone part resulted in an obvious loss in reaction yield, consistent with results from diazoacetate species (**7g** and **7n**).

### Conversion of the obtained product

**Fig. 5:**
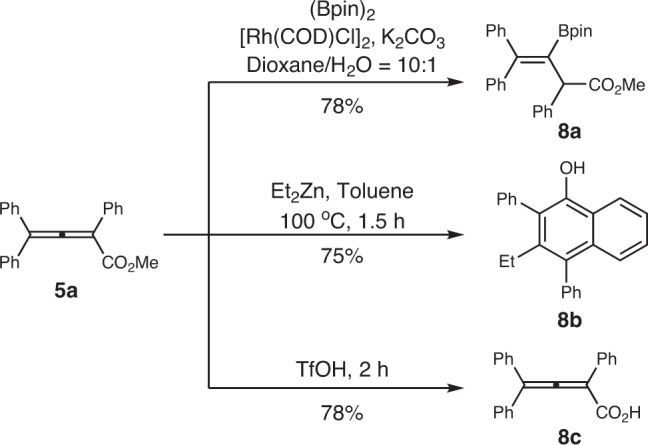
Conversion of the obtained product 5a. Reaction conditions and isolated yield are reported along the arrows.

The conversion of allene **5a** was tested (Fig. 5). In the presence of a rhodium catalyst, the allenic esters can be selectively borylated by B_2_(pin)_2_ to afford vinyl boronate pinacol ester **8a** in 78% yield. According to the previous report^49^, the allenic esters can also undergo a sequential nucleophilic attack/cyclization process to give polysubstituted α-naphthol **8b** in moderate yield. Treatment of **5a** with TfOH afforded allenic carboxylic acid **8c** in 78% yield, which may be used to attach this unique allene architecture to other molecules.

### Control experiments

**Fig. 6:**
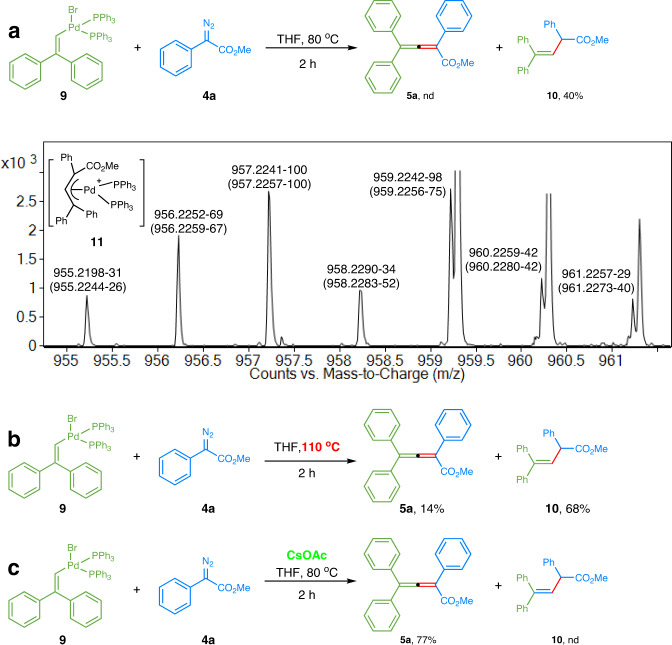
Control experiments with palladium complex. **a** Reaction of palladium complex **9** and diazoacetate **4a** at 80 ^o^C in the absence of base. **b** Reaction of palladium complex **9** and diazoacetate **4a** at 110 ^o^C in the absence of base. **c** Reaction of palladium complex **9** and diazoacetate **4a** at 80 ^o^C in the presence of CsOAc.

To probe the mechanism of this catalytic reaction, palladium complex **9** was prepared and subjected to several control experiments (Fig. 6). Palladium complex **9** was prepared by reaction of 2,2-diphenylvinyl bromide **3a** and Pd(PPh_3_)_4_, and the structure was verified by X-ray crystallographic analysis. See SI for details.

When the mixture of complex **9** and diazoacetate **4a** in THF was heated at 80 ^o^C for 2 h, the reaction solely afforded olefin **10** (Fig. 6a). The reaction mixture was also analyzed by SAESI-HRMS, which is a direct and reliable method for the characterization of reaction intermediates in situ through a gentle ionization process^50–53^. The obtained MS spectrum showed a signal of palladium complex [C_59_H_49_O_2_P_2_Pd]^+^. The peaks in MS spectrum labeled as experimental *m/z*-relative percentage abundance matched the theoretical shown in brackets, unambiguously indicating the existence of allylic palladium species **11**. The relative abundance of the isotopic ion at *m/z* 959.2242 was higher than the theoretical value due to the influence of the background signal nearby.

A small amount of allene **5a** could be observed upon elevation of reaction temperature, with olefin **10** still as the major product (Fig. 6b). However, the preference of reaction products was completely inverted when cesium acetate was added, and only allene **5a** was produced even at 80 ^o^C (Fig. 6c). These experiments hint that the reaction generated an allylpalladium intermediate, which could undergo either protodepalladation^54^ to afford olefin **10**, or hydrogen elimination to give allene **5a**. Such a hydrogen elimination step can be facilitated by the basic carboxylate salts.

### KIE and deuterium-labeling experiments

**Fig. 7:**
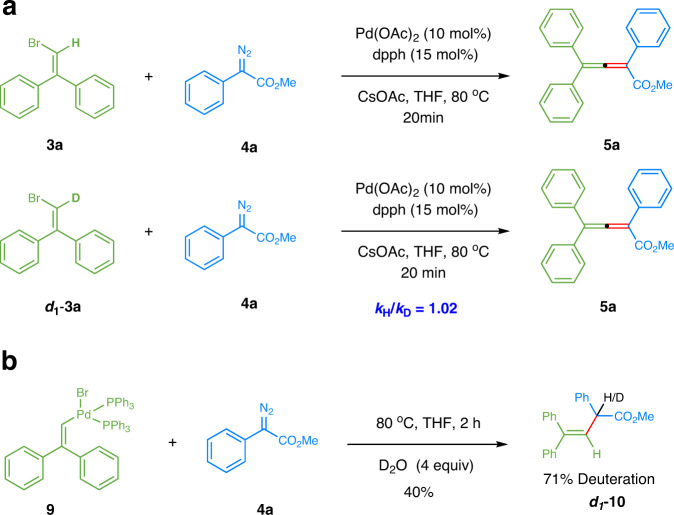
KIE and deuterium-labeling experiments. **a** KIE determined from two parallel reactions. **b** Deuterium incorporation experiment.

To gain more mechanistic insights, two deuterium labeling experiments were carried out (Fig. 7). The kinetic isotope effect (KIE) was measured in two parallel reactions using **3a** and deuterium-labeled ***d***_***1***_**-3a**. A KIE value of 1.02 implicated that the final hydrogen elimination was not involved in the rate-limiting step^55^. In the presence of 4 equivalents of D_2_O, the reaction of complex **9** and diazoacetate **4a** afforded deuterated olefin ***d***_1_-**10** with 71% D incorporation, showing the possibility of the protodepalladation by the moisture of the reaction system in the absence of a carboxylate salt.

### Proposed reaction mechanism

**Fig. 8:**
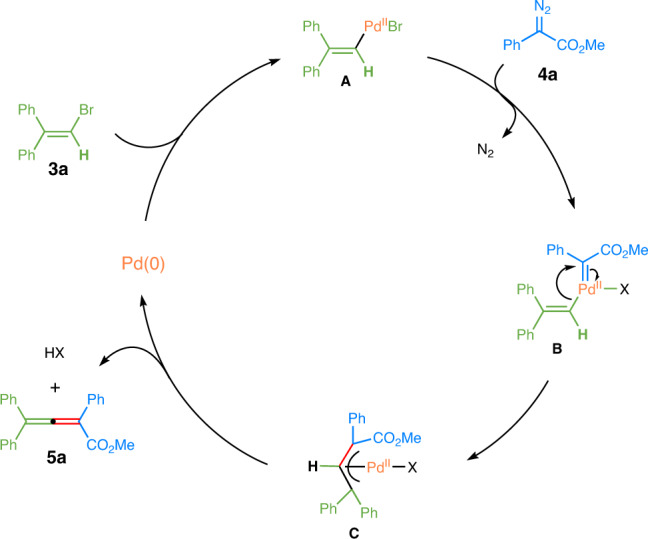
Plausible reaction mechanism. X = Br or OAc; ligands are omitted for clarity.

Based on the above investigations and literature precedents, a plausible mechanism is outlined in Fig. 8. Initially, oxidative addition of vinyl bromide **3a** to the Pd^0^ catalyst offers the Pd^II^ species **A**. Then the Pd^II^ species **A** reacts with the diazoacetate **4a** to form Pd^II^ carbene species **B**. A subsequent migratory insertion of carbene into the Pd−C bond affords *π*-allylpalladium species **C**^56–59^, which is followed by a hydrogen-elimination to provide the desired product **5a**.

### DFT calculations

**Fig. 9:**
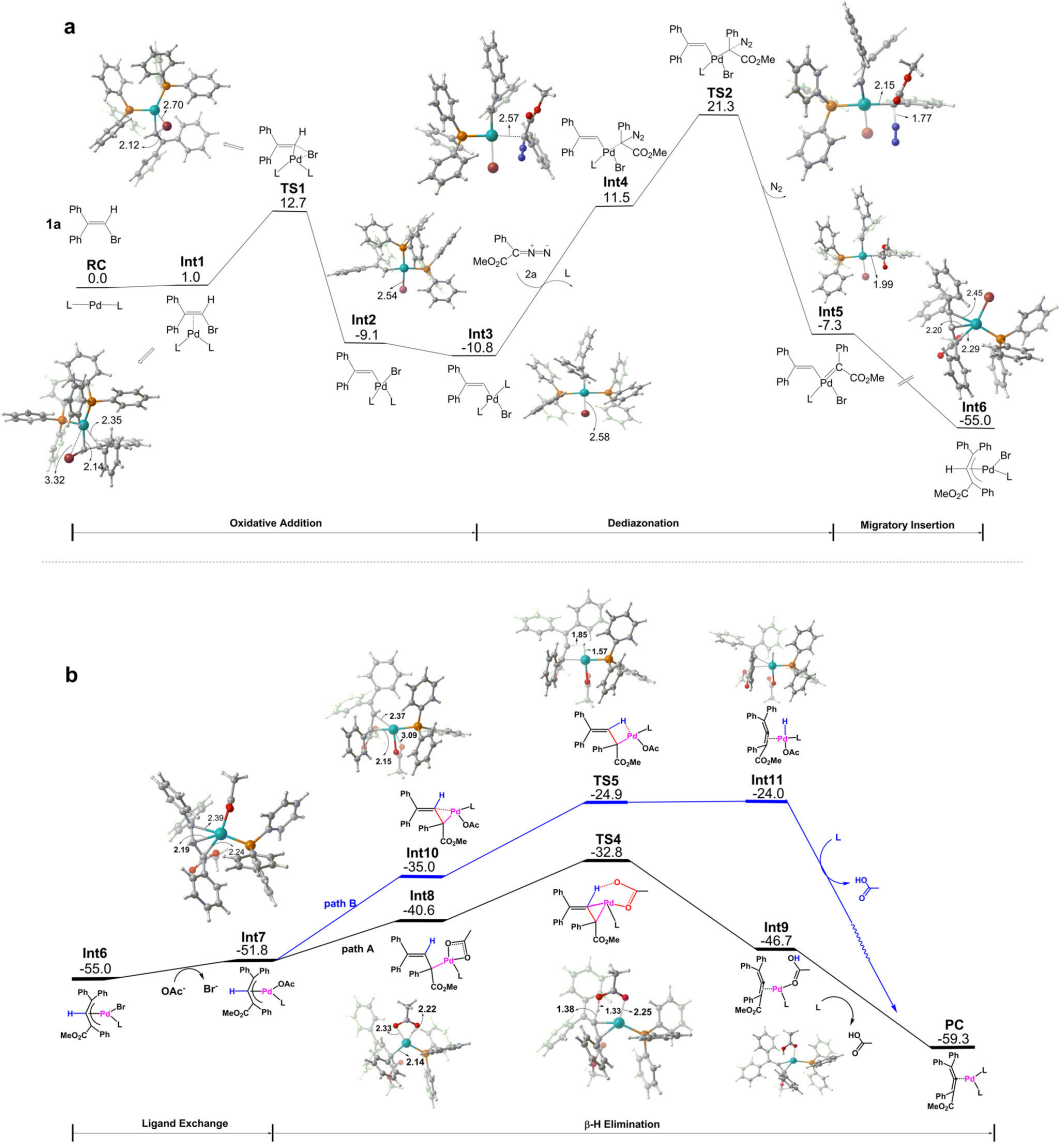
Energy profiles and geometries of key species for the tentative reaction pathways. **a** Energy profiles and geometries of key species for the formation of allylpalladium species. **b** Energy profiles and geometries of key species for *β*-hydrogen elimination mechanism. All results are calculated at the SMD (tetrahydrofuran) M06/def2-TZVP//B3LYP/6-31G(d)(LANL2DZ) level of theory. Relative free energies are in kcal/mol and bond lengths are in Å.

To gain a deeper understanding of the palladium-catalyzed cross-coupling of vinyl bromides with diazo compounds, DFT calculations were carried out for the envisioned reaction intermediates and related transition states (Fig. 9, see computational details in the Supplementary Information).

As can be seen from Fig. 9a, the oxidative addition of vinylbromide **3a** to the Pd(0)L_2_ complex (L = PPh_3_), which is exergonic by 9.1 kcal/mol with an energy barrier of 12.7 kcal/mol, initiates the reaction and offers bromide coordinated species **Int2**. Isomerization of **Int2** by exchanging the positions of vinyl ligand and PPh_3_ ligand forms a more stable isomer **Int3**. We also explored the reaction pathway initiated by the addition of diazo compound with Pd(0)L_2_. However, as the dediazonation process in this pathway requires a high free energy barrier of 33.2 kcal/mol (see Supplementary Fig. 4 in Supplementary Information), such a mechanism will not be further considered. The subsequent addition of diazoacetate **4a** to **Int3** with simultaneous dissociation of one PPh_3_ group is endergonic by 22.3 kcal/mol and leads to **Int4** in which diazoacetate weakly occupies the vacant site of Pd(II). Further reaction of diazoacetate with Pd(II) center releases a molecule of nitrogen and leads to a Pd(II) carbene intermediate **Int5**. This step is calculated to be 18.8 kcal/mol exothermic with an energy barrier of 9.8 kcal/mol. A subsequent migratory insertion of the generated carbene into the Pd−C bond of **Int5** affording the *π*-allylpalladium species **Int6** is further exothermic by 47.7 kcal/mol without any energy barrier. We also investigated an alternative reaction pathway for *π*-allylpalladium species formation, in which ligand exchange of bromide with the base CsOAc happens before dediazonation and migratory insertion (see Supplementary Fig. 2 in Supplementary Information). However, with an overall energy barrier of about 36.4 kcal/mol, this reaction pathway seems to be unfavorable in practice and thus will not be discussed further. Nevertheless, as shown in Fig. 9b, the ligand exchange indeed takes place after the formation of **Int6** to produce base coordinated *π*-allylpalladium species **Int7** with an endergonic reaction energy of 3.2 kcal/mol. The so-generated **Int7** then undergoes *β*-hydrogen elimination to provide the desired allene product. At this stage, two alternative pathways, of which one involves the coordinated base (path A in Fig. 9b) while the other involves the palladium center (path B in Fig. 9b), were investigated respectively. As depicted in path A, isomerization of **Int7** to δ-palladium *tetra*-coordinated intermediate **Int8** is endergonic by 11.2 kcal/mol, with the base AcO^−^ acting as a bidentate chelate ligand and *β*-hydrogen of allyl ligand getting close to one of the oxygen atoms of AcO^−^. The resulting **Int8** then undergoes base promoted *β*-hydrogen elimination through a bicyclo[4.1.0] transition state (**TS4**) to afford allene coordinated complex (**Int9**) with a small energy barrier of 7.8 kcal/mol accompanying with an exothermicity of 6.1 kcal/mol. Finally, the Pd(0)L_2_ coordinated with allene is restored by ligand exchange between the coordinated AcOH of **Int9** and PPh_3_ with an exergonic reaction energy of 12.6 kcal/mol indicating that the whole reaction is thermodynamically favorable. In contrast, due to the sterically more crowded structure possessed in the isomer **Int10** and the larger tension of the four-membered ring transition state (**TS5**), the reaction mechanism via path B is energetically unfavorable. As such, the results from our DFT calculations suggest that palladium-catalyzed cross-coupling of 2,2-diarylvinylbromides with diazo compounds to produce allenes involves base promoted *β*-hydrogen elimination mechanism. The rate-determining step is found to be dediazonation with the overall energy barrier of 32.1 kcal/mol (**TS2** vs **Int3** in Fig. 9a), which is somewhat high considering the reaction temperature of 80 ^o^C, possibly due to the insufficient accuracy of DFT methods in some cases. The experimentally observed small KIE supports the DFT results that *β*-hydrogen elimination is not the rate-determining step (for computational details, see Supplementary Data 1).

In summary, We have developed a highly efficient palladium-catalyzed cross-coupling of 2,2-diarylvinyl bromides with diazo compounds for the modular synthesis of tetrasubstituted allenes. The reaction can be promoted by either mono*-* or bis-phosphine ligands, and ligand dpph with a flexible six-carbon linkage proved to be the optimal choice. Under optimized conditions, both aryl diazoacetates and *N*-tosylhydrazones are competent coupling partners. To gain insight into the reaction mechanism, control experiments, KIE studies, and DFT calculations were carried out. The key step in the catalytic cycle is believed to undergo a *β*-vinylic hydrogen elimination from allyl palladium intermediate, where acetate anion acts as an inner base to form a bicyclo[4.1.0] transition state. The computational study also indicates that the rate-determining step is dediazonation with the overall energy barrier of 32.1 kcal/mol. Notably, the *β*-hydrogen elimination mode revealed by the present work deepened our understanding of this elementary step in palladium catalysis and paved a new way for the allene synthesis.

## Methods

### Typical procedure for coupling of 2,2-diarylvinyl bromides with diazo carbonyl compounds

To a 25 mL Schlenk tube charged with a stir bar, 2,2-diarylvinyl bromides (**3**) (0.2 mmol), α-diazoesters (**4**) (0.3 mmol), Pd(OAc)_2_ (4.48 mg, 0.02 mmol), dpph (13.6 mg, 0.03 mmol) and CsOAc (58 mg, 0.3 mmol) were added. After filled with argon, anhydrous THF (2 mL) were added via a syringe. The mixture was stirred at 80 °C in an oil bath for 2 h. Upon completion, the reaction mixture was washed with brine (15 mL) and extracted with EtOAc (3 × 10 mL). The combined organic phases were dried over anhydrous Na_2_SO_4_, filtered, and concentrated under reduced pressure. The crude products were purified by silica gel chromatography (petroleum ether/EtOAc = 20:1 ~ 5:1) to afford pure products (**5**).

### Typical procedure for 2,2-diarylvinyl bromides with *N*-tosylhydrazones

To a 25 mL Schlenk tube charged with a stir bar, 2,2-diarylvinyl bromides (**3**) (0.2 mmol), *N*-tosylhydrazones (**6**) (0.3 mmol), Pd(OAc)_2_ (4.48 mg, 0.02 mmol), DPPE (11.9 mg, 0.03 mmol) and CsOPiv (234 mg, 1 mmol) were added. After filled with argon, anhydrous THF (5 mL) was added via a syringe. The mixture was stirred at 80 °C in an oil bath for 4 h. Upon completion, the reaction mixture was washed with brine (15 mL) and extracted with EtOAc (3 × 10 mL). The combined organic phases were dried over anhydrous Na_2_SO_4_, filtered, and concentrated under reduced pressure. The crude products were purified by silica gel chromatography (petroleum ether/EtOAc = 100:1 ~ 20:1) to afford pure products (**7**).

## Supplementary information

Supplementary Information

Description of Additional Supplementary Files

Supplementary Data 1

## Data Availability

Detailed experimental procedures and characterization of compounds can be found in the Supplementary Information. The X-ray crystallographic coordinates for structures reported in this study have been deposited at the Cambridge Crystallographic Data Centre (CCDC) under deposition numbers CCDC 1918015 (**5a**), 1918277 (**9**).
