# Supplementary material for: Palladium-catalyzed allene synthesis enabled by β-hydrogen elimination from sp2-carbon
Source: Nat Commun. 2021 Feb 1;12:728. doi: 10.1038/s41467-020-20740-w (PMC7851150; doi:10.1038/s41467-020-20740-w)
Supplement: Supplementary file 1 — Supplementary Information [file 41467_2020_20740_MOESM1_ESM.pdf]

# Supplementary Information

## Palladium-catalyzed allene synthesis enabled by $\beta$-hydrogen eliminations from a $sp^2$ hybrid carbon

Ge Zhang,[1, 2, 4] Yi-Kang Song,[1] Fang Zhang,[1] Ze-Jian Xue,[2] Meng-Yao Li,[2] Gui-Shan Zhang,[2] Bin-Bin Zhu,[2] Jing Wei,*[, 3] Chunsen Li,[3] Chen-Guo Feng,*[, 1, 2] Guo-Qiang Lin*[, 1, 2, 4]

[1]The Research Center of Chiral Drugs, Innovation Research Institute of Traditional Chinese Medicine, Shanghai University of Traditional Chinese Medicine, 1200 Cailun Road, Shanghai 201203, China.

[2]CAS Key Laboratory of Synthetic Chemistry of Natural Substances, Center for Excellence in Molecular Synthesis, Shanghai Institute of Organic Chemistry, University of Chinese Academy of Sciences, Chinese Academy of Sciences, 345 Lingling Road, Shanghai 200032, China.

[3]State Key Laboratory of Structural Chemistry, Fujian Institute of Research on the Structure of Matter, Chinese Academy of Sciences, Fuzhou, 155 West Yangqiao Road, Fujian 350002 China.

[4]School of Physical Science and Technology, ShanghaiTech University, 393 Huaxia Road, Shanghai, 201210, China.

# Table of Contents

**1. Supplementary Notes** **2**

**2. Supplementary Methods** **2**

**3. Supplementary Tables** **7**

**4. Supplementary Figures** **8**

**5. Copies of $^{1}$H NMR, $^{13}$C NMR, $^{19}$F NMR and $^{31}$P NMR Spectra** **21**

**6. Supplementary References** **68**

# 1. Supplementary Notes

Commercially available reagents were used without further purification unless specified. CsOAc and CsOPiv was purchased from Adams-beta Company. Tetrahydrofuran was distilled from sodium/benzophenone before use. Unless otherwise stated, reactions were performed with freshly dried solvents utilizing standard Schlenk techniques under pre-dried argon. Analytical thin layer chromatography (TLC) was performed on silica gel 60 $F_{254}$ plates. TLC plates were visualized by exposure to short wave ultraviolet light (254 nm, 365 nm) and were dipped into a solution of $KMnO_4$. Flash chromatography was performed on silica gel 60 (40-63 µm) under a positive pressure of air. NMR-spectra were recorded on a Bruker Avance II 400 spectrometer. Chemicals shifts (δ) are quoted in ppm downfield of tetramethylsilane. The residual solvent signals were used as references for **$^{1}$H** and **$^{13}$C NMR** spectra ($CDCl_3$: δ H = 7.26 ppm, δ C = 77.16 ppm;). **$^{19}$F** and **$^{31}$P NMR** spectra are not calibrated by an internal reference. The multiplicity of all signals was described with standard abbreviations: s = singlet, d = doublet, t = triplet, q = quartet, quintet = quint, heptet = hept, m = multiplet, br = broad resonance. Coupling constants (*J*) are quoted in Hz. GC-MS spectra were recorded on an Agilent Technologies 7890A GC-system with an Agilent 5975C VL MSD or an Agilent 5975 inert Mass Selective Detector (EI) and a HP-5MS column (0.25 mm x 30 m, film: 0.25 µm). High-resolution mass spectrometry was measured using an Agilent 6210 TOF LC/MS spectrometer or a Waters Quattro micro GC/MS/MS spectrometer.

The synthesis method and data of 2,2-diarylvinyl bromides (**3**) were published following the related references.[1] The synthesis method of α-diazoester (**5**), *N*-Tosylhydrazones (**6**) was reported following the related references.[2-5]

# 2. Supplementary Methods

## 2.1 General Procedure for Triphenylallenic Esters (5)

To a 25 mL Schlenk tube charged with a stir bar, 2,2-diarylvinyl bromides (**3**) (52.2 mg, 0.2 mmol), α-diazoesters (**4**) (50.1 mg, 0.3 mmol), $Pd(OAc)_2$ ( 4.48 mg, 0.02 mmol), DPPH (13.6 mg, 0.03 mmol) and CsOAc (58 mg, 0.3 mmol) were added. After filled with argon, anhydrous THF (2 mL) were added via a syringe. The mixture was stirred at 80 ℃ in an oil bath for 2 h. Upon completion, the reaction mixture was washed with brine (15 mL) and extracted with EtOAc (3×10 mL). The combined organic phase was dried over anhydrous $Na_2SO_4$. After that the organic phase was filtered, and concentrated under reduced pressure. The crude products were purified by silica

gel chromatography (PE/EA = 20:1 ~ 5:1) to afford pure products (**5**).

## 2.2 General Procedure for Tetraphenylallenes (7)

To a 25 mL Schlenk tube charged with a stir bar, 2,2-diarylvinyl bromides (**3**) (0.2 mmol), *N*-Tosylhydrazones (**6**) (0.3 mmol), $Pd(OAc)_2$ (4.48 mg, 0.02 mmol), DPPE (11.9 mg, 0.03 mmol) and CsOPiv (234 mg, 1 mmol) were added. After filled with argon, anhydrous THF (5 mL) were added via a syringe. The mixture was stirred at 80 ℃ in an oil bath for 4 h. Upon completion, the reaction mixture was washed with brine (15 mL) and extracted with EtOAc (3×10 mL). The combined organic phase was dried over anhydrous $Na_2SO_4$. After that the organic phase was filtered, and concentrated under reduced pressure. The crude products were purified by silica gel chromatography (PE/EA = 100:1 ~ 20:1) to afford pure products (**7**).

## 2.3 Conversion of the Obtained Products

**Synthesis of 8a:**

To a 25 mL Schlenk tube charged with a stir bar, allenes (**5a**) (0.2 mmol), $(Bpin)_2$ (76 mg, 0.3 mmol), $[Rh(COD)Cl]_2$ (2.5 mg, 0.005 mol), $K_2CO_3$ (41 mg, 0.3 mol) were added. After filled with argon, Dioxane/$H_2O$ = 10:1 were added via a syringe. The mixture was stirred at 80 ℃ in an oil bath for 2 hours. Upon completion, the reaction mixture was washed with brine (15 mL) and extracted with EtOAc (3×10 mL). The combined organic phase was dried over anhydrous $Na_2SO_4$. After that the organic phase was filtered, and concentrated under reduced pressure. The crude products were purified by silica gel chromatography (PE/EA = 5:1) to afford pure products (**8a**) with 78% yield.

**Synthesis of 8b:**

To a 25 mL Schlenk tube charged with a stir bar, allenes (5**a**) (0.2 mmol) were added. After filled with argon, toluene (3 mL) were added via a syringe. Then, $Et_2Zn$ (0.6 mL, 1 M in toulene ) was added dropwise in 0 ℃. The mixture was stirred at 100 ℃ in an oil bath for 1.5 hours. Upon completion, the reaction mixture was washed with brine (15 mL) and extracted with EtOAc (3×10 mL). The combined organic phase was dried over anhydrous $Na_2SO_4$. After that the organic phase was filtered, and concentrated under reduced pressure. The crude products were purified by silica gel chromatography (PE/EA = 5:1) to afford pure products (**8b**) with 75% yield.

**Synthesis of 8c:**

To a 25 mL Schlenk tube charged with a stir bar, allenes (**5a**) (0.2 mmol) were added. After

added $CF_3CO_2H$ (2 mL) at room temperature. The mixture was stirred for 2 hours. Upon completion, the reaction mixture was washed with brine (15 mL) and extracted with EtOAc (3×10 mL). The combined organic phase was dried over anhydrous $Na_2SO_4$. After that the organic phase was filtered, and concentrated under reduced pressure. The crude products were purified by silica gel chromatography (PE/EA = 3:1) to afford pure products (**8c**) with 78% yield.

## 2.4 Control Experiments

**Synthesis of 9:**

To a 25 mL Schlenk tube charged with a stir bar, $Pd(PPh_3)_4$ (1 mmol), 2,2-diarylvinyl bromides (**3**) (258 mg, 1 mmol) were added. After filled with argon, anhydrous THF (2 mL) were added via a syringe. The mixture was stirred at 80 ℃ in an oil bath for 2 hours. Upon completion, the reaction mixture was washed with brine (15 mL) and extracted with EtOAc (3×10 mL). The combined organic phase was dried over anhydrous $Na_2SO_4$. After that the organic phase was filtered, and concentrated under reduced pressure. The palladium complex **9** was obtained by recrystallization in DCM:PE = 5:1 with 79% yield.

**Procedure of a:**

To a 25 mL Schlenk tube charged with a stir bar, palladium complex (**9**) (178 mg, 0.2 mmol), α-diazoester (**4a**) (51 mg, 0.3 mmol) were added. After filled with argon, anhydrous THF (2 mL) were added via a syringe. The mixture was stirred at 80 ℃ in an oil bath for 2 hours. Upon completion, the reaction mixture was washed with brine (15 mL) and extracted with EtOAc (3×10 mL). The combined organic phase was dried over anhydrous $Na_2SO_4$. After that the organic phase was filtered, and concentrated under reduced pressure. The crude product was purified by silica gel chromatography (PE/EA = 20:1) to get the product **10**.

**Procedure of b:**

To a 25 mL Schlenk tube charged with a stir bar, palladium complex (**9**) (0.2 mmol), α-diazoester (**4a**) (51 mg, 0.3 mmol) were added. After filled with argon, anhydrous THF (2 mL) were added via a syringe. The mixture was stirred at 110 ℃ in an oil bath for 2 hours. Upon completion, the yield of the product was detected by GC analysis (n-Dodecane as the internal standard).

**Procedure of c:**

To a 25 mL Schlenk tube charged with a stir bar, palladium complex (**9**) (0.2 mmol), α-diazoester (**4a**) (51 mg, 0.3 mmol) were added. After filled with argon, anhydrous THF (2 mL)

were added via a syringe. The mixture was stirred at 80 ℃ in an oil bath for 2 hours. Upon completion, the reaction mixture was washed with brine (15 mL) and extracted with EtOAc (3×10 mL). The combined organic phase was dried over anhydrous $Na_2SO_4$. After that the organic phase was filtered, and concentrated under reduced pressure. The crude product was purified by silica gel chromatography (PE/EA = 20:1) to get the product **5a**.

## 2.5 Mass spectrometric experiments

### SAESI-HRMS conditions

SAESI-HRMS spectra were recorded on an Agilent 6545 quadrupole time-of-flight mass spectrometer (QTOF MS) equipped with a home-made SAESI ion source in positive mode. Using SAESI ion source, the Pd complex sample solution in “non-ESI-friendly” solvent dioxane could be well ionized with an assistant polar solvent such as methanol with two separated sprayers meeting at the spray end tip. The reaction solutions were injected by a 250-μL air-tight syringe with a speed at 10 μL/min to SAESI-HRMS. The assisted solvent of methanol was injected by an Agilent 1290 HPLC system with a speed at 10 μL/min to SAESI-HRMS. TOF for MS was operated at 2 spectra/s acquiring the mass range *m/z* 100-1500 and about 20,000 FWHM. The basic SAESI-HRMS conditions were: vacuum, $2.7\times10^{-7}$ torr; spray voltage, 4000 V; capillary temperature, 325 ℃; sheath gas of two sprayers, 20 psig; and drying gas, 10 L/min. Nitrogen was used as the sheath gas and drying gas. MassHunter software package was applied for the control of the equipment, acquisition and treatment of data. In isotope distribution calculation, the constant resolving power was set at 20,000 FWHM.

## 2.6 Deuterium-Labeling Experiments

To a 25 mL Schlenk tube charged with a stir bar, 2,2-diarylvinyl bromides (**3a**) (52 mg, 0.2 mmol), α-diazoesters (**4**) (50.1 mg, 0.3 mmol), $Pd(OAc)_2$ ( 4.48 mg, 0.02 mmol), DPPH (13.6 mg, 0.03 mmol) and CsOAc (58 mg, 0.3 mmol) were added. After filled with argon, anhydrous THF (2 mL) were added via a syringe. The mixture was stirred at 110 ℃ in an oil bath for 20 min. Then, the yield of the product was detected by GC analysis (*n*-Dodecane as the internal standard).

To a 25 mL Schlenk tube charged with a stir bar, $d_1$-2,2-diarylvinyl bromides ($d_1$-**3a**) (52 mg, 0.2 mmol), α-diazoesters (**4**) (50.1 mg, 0.3 mmol), $Pd(OAc)_2$ ( 4.48 mg, 0.02 mmol), DPPH (13.6 mg, 0.03 mmol) and CsOAc (58 mg, 0.3 mmol) were added. After filled with argon, anhydrous THF (2 mL) were added via a syringe. The mixture was stirred at 80 ℃ in an oil bath for 20 min. Then, the yield of the product was detected by GC analysis (n-Dodecane as the internal standard).

To a 25 mL Schlenk tube charged with a stir bar, palladium complex (**8**) (0.1 mmol), α-diazoester (**4a**) (26 mg, 0.15 mmol) were added. After filled with argon, $D_2O$ (7.5 mg, 4 equiv) and anhydrous THF (2 mL) were added via a syringe. The mixture was stirred at 80 ℃ in an oil bath for 2 hours. Upon completion, the reaction mixture was washed with brine (15 mL) and extracted with EtOAc (3×10 mL). The combined organic phase was dried over anhydrous $Na_2SO_4$. After that the organic phase was filtered, and concentrated under reduced pressure. The ratio was determined by H NMR analysis.

## 3. Supplementary Tables

**Supplementary Table 1**. Thermal correction of Gibbs free energy (TCG, hartree) and total electronic energies (E, hartree) in tetrahydrofuran solvent for all species involved in this study

| Entry | Compounds | TCG | E |
|---|---|---|---|
| 1 | **1a** | 0.164157 | -3113.93552393 |
| 2 | **Pd(0)** | 0.475787 | -2200.02336310 |
| 3 | **2a** | 0.119366 | -607.52188437 |
| 4 | **L** | 0.227910 | -1035.99699729 |
| 5 | **$N_2$** | -0.012849 | -109.50056684 |
| 6 | **OAc-** | 0.020702 | -228.56854855 |
| 7 | **Br-** | -0.016176 | -2574.21460358 |
| 8 | **HOAc** | 0.034677 | -229.06381177 |
| 9 | **Int1** | 0.665707 | -5313.98313208 |
| 10 | **TS1** | 0.661902 | -5313.96065026 |
| 11 | **Int2** | 0.668052 | -5314.00154674 |
| 12 | **Int3** | 0.673006 | -5314.00912636 |
| 13 | **Int4** | 0.555743 | -4885.48981848 |
| 14 | **TS2** | 0.552667 | -4885.47110462 |
| 15 | **Int5** | 0.550118 | -4776.00059819 |
| 16 | **Int6** | 0.552561 | -4776.07907770 |
| 17 | **Int7** | 0.599281 | -2430.43773649 |
| 18 | **Int8** | 0.598498 | -2430.41922901 |
| 19 | **TS4** | 0.592873 | -2430.40124586 |
| 20 | **Int9** | 0.594562 | -2430.42508047 |
| 21 | **Int10** | 0.598194 | -2430.41001017 |
| 22 | **TS5** | 0.593596 | -2430.38925825 |
| 23 | **Int11** | 0.592738 | -2430.38707389 |
| 24 | **Int6'** | 0.550912 | -4776.07795383 |
| 25 | **Int7'** | 0.599059 | -2430.43645049 |
| 26 | **Int8'** | 0.596511 | -2430.41552132 |
| 27 | **TS4'** | 0.592586 | -2430.39689582 |
| 28 | **Int9'** | 0.593616 | -2430.42318462 |
| 29 | **Int10'** | 0.596003 | -2430.40334574 |
| 30 | **TS5'** | 0.592014 | -2430.38228739 |
| 31 | **Int11'** | 0.592827 | -2430.38563620 |
| 32 | **PC** | 0.797159 | -3237.38769700 |

# 4. Supplementary Figures

## 4.1 Detection for the Pd complex

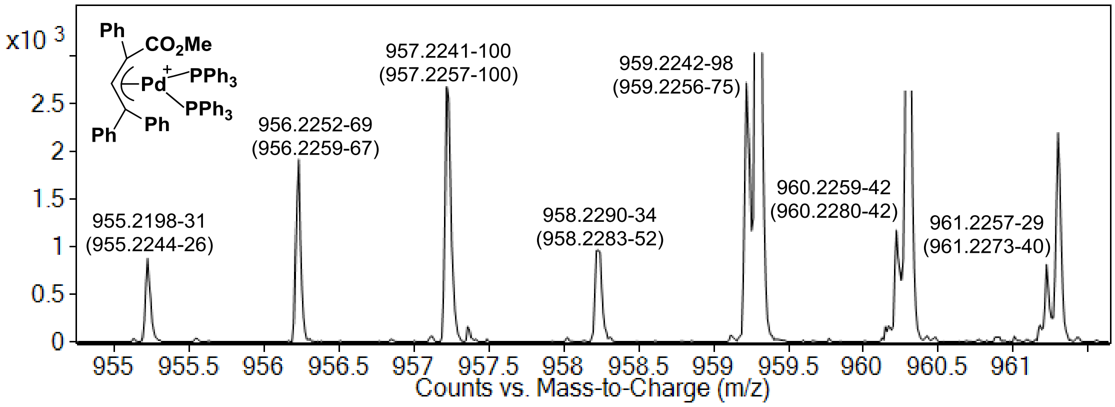


**Supplementary Figure 1.** (a) SAESI-HRMS spectrum of the reaction solution prepared by mixing **9** and **4a** in dioxane, showing the Pd complex ion $[C_{59}H_{49}O_2P_2Pd]^+$ at *m/z* 957.2241.

## 4.2 DFT caculation

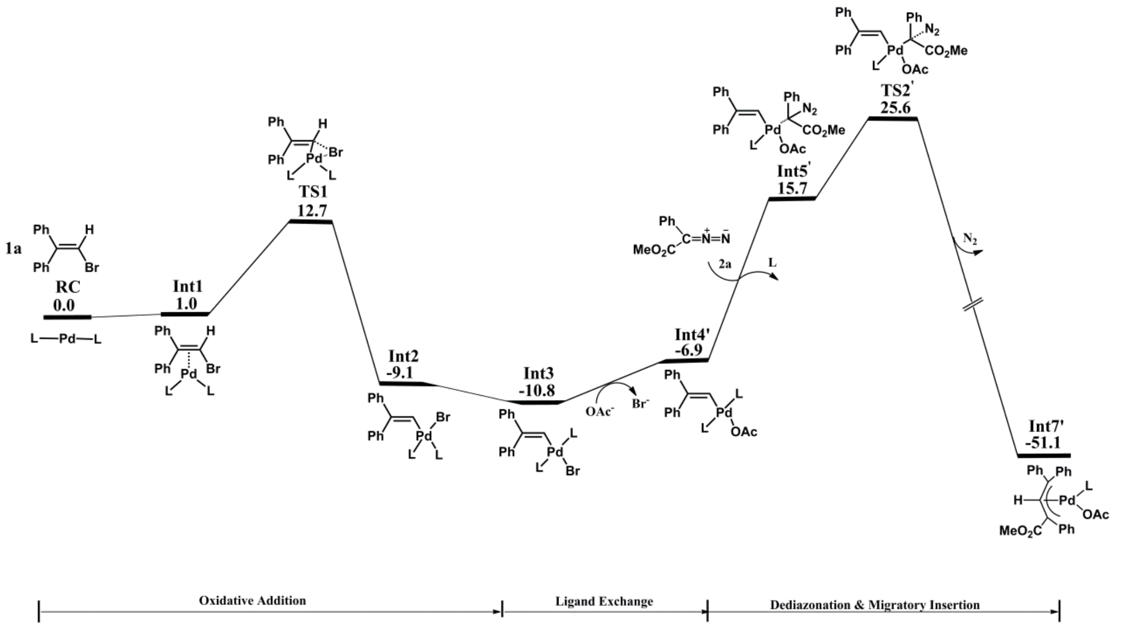


**Supplementary Figure 2**. Energy profiles for the reaction pathways for π-allylpalladium species formation, in which ligand exchange of bromide with the base CsOAc happens before dediazonation and migratory insertion. Relative free energies are in kcal/mol.

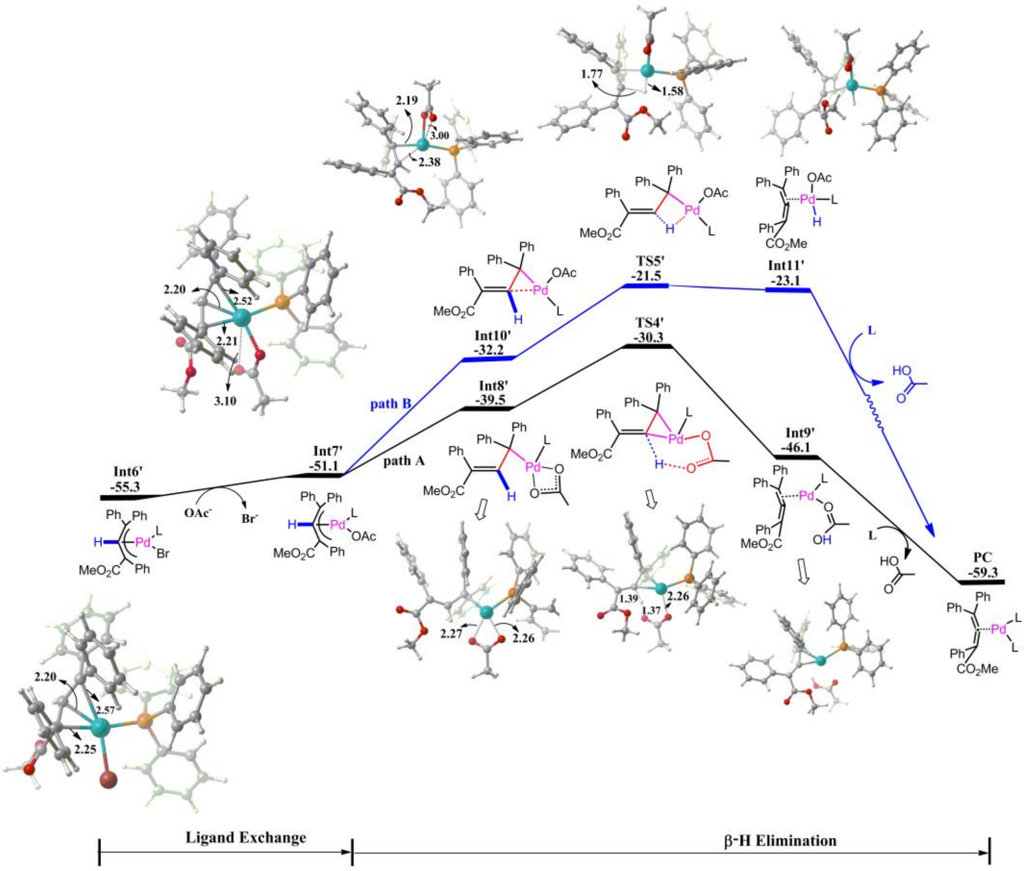


**Supplementary Figure 3.** Energy profiles for the reaction pathways similar as those shown in Figure 1b. The main differences exist in the optimized geometric structure features. However, as the higher barrier obtained, these reaction pathways were not further discussed. Relative free energies are in kcal/mol and bond lengths are in Å.

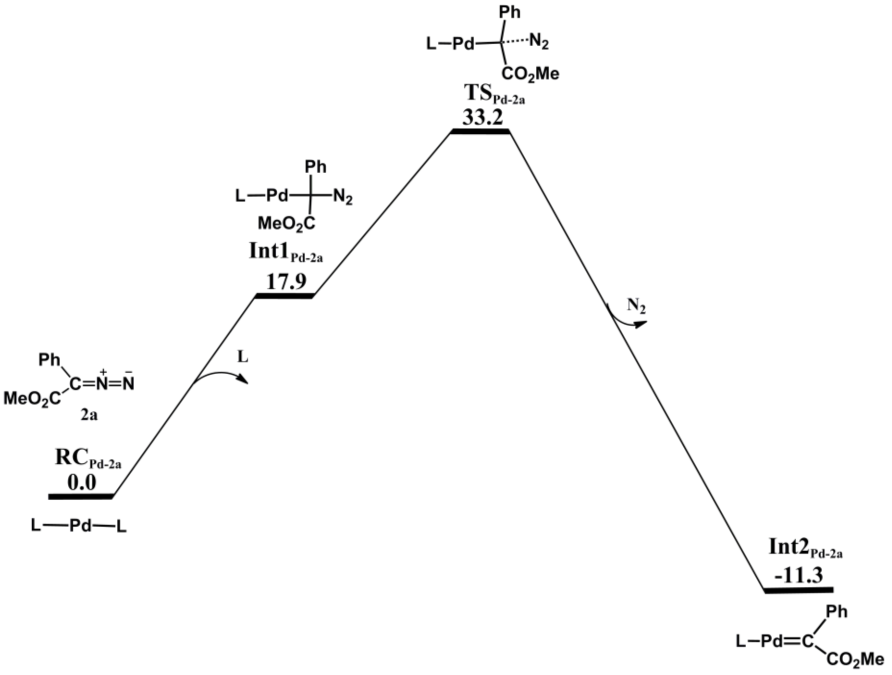


**Supplementary Figure 4**. Energy profiles for the reaction of $PdL_2$ (L=$PPh_3$) with the diazo compound. Relative free energies are in kcal/mol. One $PPh_3$ ligand is substituted

with 2a to give intermediate Int1$_{Pd\text{-}2a}$, and then release of $N_2$ is followed via transition state TS$_{Pd\text{-}2a}$ to afford intermediate Int2$_{Pd\text{-}2a}$. The overall activation barrier calculated is 33.2 kcal/mol that is much higher than the oxidative addition barrier of bromide substrate (a barrier of 12.7 kcal/mol). Therefore, we predict the complex $PdL_2$ first reacts with the bromide substrate rather than the diazo compound.

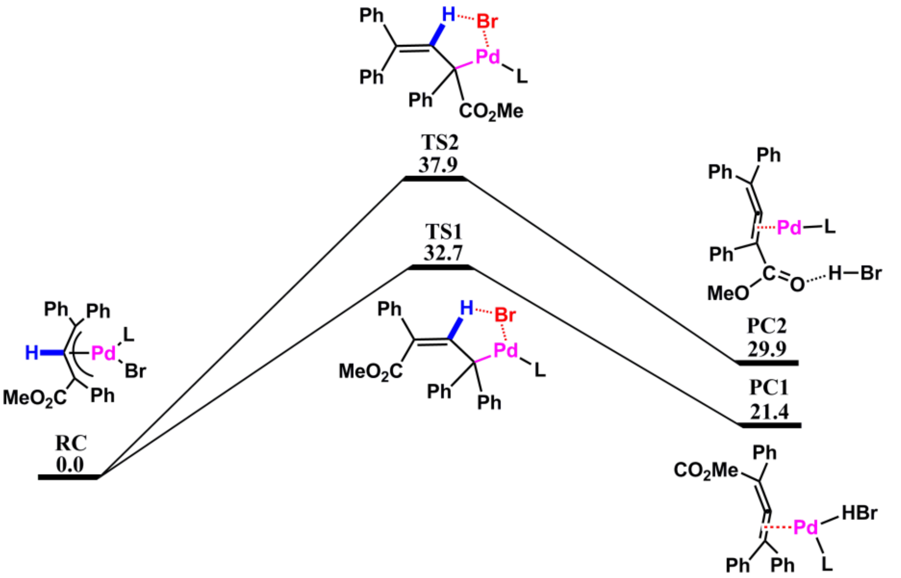


**Supplementary Figure 5.** Energy profiles for the formation of allene in the absence of base. This reaction proceeds via *β*–H elimination step, but needs much higher free energy barrier (32.7 kcal/mol and 37.9 kcal/mol vs. 22.2 kcal/mol in Fig. 1b). Thus, allene formation without base is energetically unfavorable which is in agreement with the experiments. Relative free energies are in kcal/mol.

## 4.3 Characterization data for compounds

### Methyl 2,4,4-triphenylbuta-2,3-dienoate (5a)

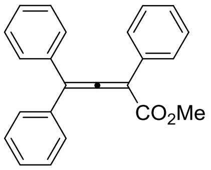

5a

White solid; TLC (PE:EA = 20:1): Rf = 0.3. **$^1$H NMR** ($CDCl_3$, 400 MHz) δ 7.64-7.58 (m, 2H), 7.47-7.26 (m, 13H), 3.86 (s, 3H); **$^{13}$C NMR** ($CDCl_3$, 100 MHz) δ 214.62, 166.39, 134.75, 132.44, 128.83, 128.78, 128.58, 128.39, 128.33, 128.05, 114.82, 105.30, 52.64; **EI-MS** (m/z, %): 326 ($M^+$, 5.63), 91 (100), 167 (37.23), 150 (36.03); **HRMS** (EI): m/z calcd for $C_{23}H_{18}O_2$ $[M]^+$: 326.1307; found: 326.1315.

### Methyl 2,4-diphenyl-4-(*p*-tolyl)buta-2,3-dienoate (5b)

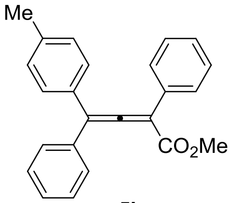

5b

White solid; TLC (PE:EA = 20:1): Rf = 0.3. **$^1$H NMR** ($CDCl_3$, 400 MHz) δ 7.62 (d, *J* = 7.1 Hz, 2H), 7.39-7.23 (m, 10H), 7.20-7.15 (m, 2H), 3.83 (s, 3H), 2.36 (s, 3H); **$^{13}$C NMR** ($CDCl_3$, 100 MHz) δ 214.64, 166.48, 138.36, 134.90, 132.58, 131.71, 129.56, 128.79, 128.79, 128.67, 128.55, 128.34,

128.34, 127.97, 114.70, 105.15, 52.61, 21.37; **EI-MS** (m/z, %): 340 ($M^+$, 80.37), 281 (100), 325 (70.52), 265 (59.67); **HRMS** (EI): m/z calcd for $C_{24}H_{20}O_2$ $[M]^+$: 340.1463; found: 340.1457.

**Methyl 4-(4-methoxyphenyl)-2,4-diphenylbuta-2,3-dienoate (5c)**

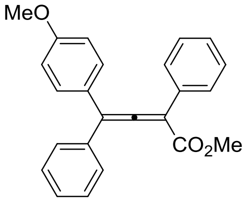


5c

White solid; TLC (PE:EA = 10:1): Rf = 0.3. **$^1$H NMR** ($CDCl_3$, 400 MHz) δ 7.61 (d, *J* = 7.3 Hz, 2H), 7.46-7.23 (m, 10H), 6.91 (d, *J* = 8.8 Hz, 2H), 3.85 (s, 3H), 3.82 (s, 3H); **$^{13}$C NMR** ($CDCl_3$, 100 MHz) δ 214.60, 166.53, 159.84, 135.05, 132.68, 130.01, 128.81, 128.80, 128.57, 128.36, 128.33, 127.96, 126.78, 114.31, 105.08, 55.49, 52.64; **EI-MS** (m/z, %): 356 ($M^+$, 60.00), 297 (100), 252 (57.94), 105 (57.59); **HRMS** (EI): m/z calcd for $C_{24}H_{20}O_3$ $[M]^+$: 356.1412; found: 356.1408.

**Methyl 4-(4-chlorophenyl)-2,4-diphenylbuta-2,3-dienoate (5d)**

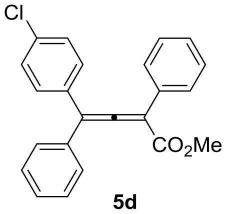


5d

White solid; TLC (PE:EA = 20:1): Rf = 0.3. **$^1$H NMR** ($CDCl_3$, 400 MHz) δ 7.61-7.57 (m, 2H), 7.44-7.23 (m, 12H), 3.86 (s, 3H); **$^{13}$C NMR** ($CDCl_3$, 100 MHz) δ 214.44, 166.22, 134.38, 134.34, 133.33, 132.18, 130.05, 129.08, 128.97, 128.72, 128.66, 128.62, 128.34, 128.23, 114.02, 105.63, 52.76; **EI-MS** (m/z, %): 360 ($M^+$, 58.27), 301 (100), 265 (92.01), 345 (56.13); **HRMS** (EI): m/z calcd for $C_{23}H_{17}O_2Cl$ [M]+: 360.0917; found: 360.0912.

**Methyl 2,4-diphenyl-4-(4-(trifluoromethyl)phenyl)buta-2,3-dienoate (5e)**

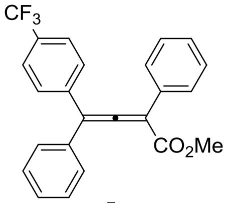


5e

White solid; TLC (PE:EA = 20:1): Rf = 0.3. **$^1$H NMR** ($CDCl_3$, 400 MHz) δ 7.67-7.60 (m, 4H), 7.55-7.52 (m, 2H), 7.45-7.22 (m, 8H), 3.87 (s, 3H); **$^{13}$C NMR** ($CDCl_3$, 100 MHz) δ 214.69, 166.08, 138.77, 134.08, 131.91, 130.40 (q, *J* = 33 Hz), 129.06, 128.77, 128.72, 128.36, 125.87, 125.84, 125.80, 125.77 (q, *J* = 3.3 Hz), 124.17 (q, *J* = 271 Hz), 114.03, 105.96, 52.82. **$^{19}$F NMR** (376 MHz, $CDCl_3$) δ -62.63; **EI-MS** (m/z, %): 394 ($M^+$, 67.16), 335 (100), 105 (66.43), 173 (52.34); **HRMS** (EI): m/z calcd for $C_{24}H_{17}O_2F_3$ $[M]^+$: 394.1181; found: 394.1175.

**Methyl 2,4-diphenyl-4-(m-tolyl)buta-2,3-dienoate (5f)**

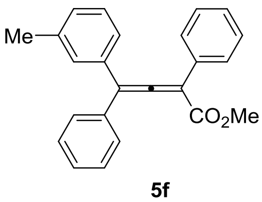


5f

White solid; TLC (PE:EA = 20:1): Rf = 0.3. **$^1$H NMR** ($CDCl_3$, 400 MHz) δ 7.67-7.57 (m, 2H), 7.50-7.19 (m, 11H), 7.15 (d, *J* = 7.2 Hz, 1H), 3.85 (s, 3H), 2.34 (s, 3H); **$^{13}$C NMR** ($CDCl_3$, 100 MHz) δ 214.63, 166.49, 138.53, 134.89, 134.62, 132.52, 129.32, 129.22, 128.81, 128.81, 128.73, 128.70,

128.58, 128.35, 128.01, 125.95, 114.86, 105.17, 52.65, 21.61; **EI-MS** (m/z, %): 340 ($M^+$,53.26), 281 (100), 105 (69.33), 165 (41.22); **HRMS** (EI): m/z calcd for $C_{24}H_{20}O_2$ $[M]^+$: 340.1463; found: 340.1467.

**Methyl 4-(3-chlorophenyl)-2,4-diphenylbuta-2,3-dienoate (5g)**

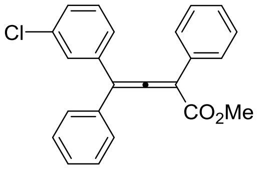


**5g**

White solid; TLC (PE:EA = 20:1): Rf = 0.3. **$^1$H NMR** ($CDCl_3$, 400 MHz) δ 7.62-7.57 (m, 2H), 7.42-7.34 (m, 8H), 7.32-7.28 (m, 4H), 3.87 (s, 3H); **$^{13}$C NMR** ($CDCl_3$, 100 MHz) δ 214.46, 166.16, 136.81, 134.80, 134.23, 132.07, 130.08, 129.05, 129.01, 128.73, 128.68, 128.52, 128.41, 128.37, 128.28, 126.98, 113.94, 105.76, 52.78; **EI-MS** (m/z, %): 360 ($M^+$, 5.73), 84 (100), 86 (63.25), 57 (26.43); **HRMS** (EI): m/z calcd for $C_{23}H_{17}O_2Cl$ $[M]^+$: 360.0917; found: 360.0923.

**Methyl 4-(3,4-dimethylphenyl)-2,4-diphenylbuta-2,3-dienoate (5h)**

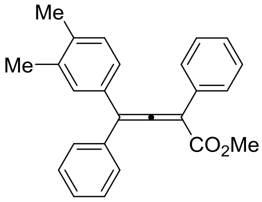


**5h**

White solid; TLC (PE:EA = 20:1): Rf = 0.3. **$^1$H NMR** ($CDCl_3$, 400 MHz) δ 7.64-7.61 (m, 2H), 7.46-7.11 (m, 11H), 3.84 (s, 3H), 2.28 (s, 3H), 2.25 (s, 3H); **$^{13}$C NMR** ($CDCl_3$, 100 MHz) δ 214.65, 166.55, 137.13, 137.09, 135.02, 132.65, 132.06, 130.11, 129.81, 128.82, 128.78, 128.55, 128.35, 128.28, 127.94, 126.27, 114.75, 105.03, 52.61, 20.00, 19.72; **EI-MS** (m/z, %): 354 ($M^+$,9.57), 167 (100), 84 (60.26), 165 (46.35); **HRMS** (EI): m/z calcd for $C_{25}H_{22}O_2$ $[M]^+$: 354.1620; found: 354.1615.

**Methyl 2,4-diphenyl-4-(o-tolyl)buta-2,3-dienoate (5i)**

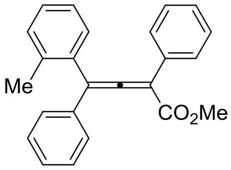


**5i**

White solid; TLC (PE:EA = 20:1): Rf = 0.3. **$^1$H NMR** ($CDCl_3$, 400 MHz) δ 7.58 (d, *J* = 7.3 Hz, 2H), 7.39-7.22 (m, 12H), 3.84 (s, 3H), 2.21 (s, 3H); **$^{13}$C NMR** ($CDCl_3$, 100 MHz) δ 213.19, 166.44, 137.36, 134.44, 133.96, 132.56, 130.69, 130.38, 128.95, 128.60, 128.56, 128.52, 128.11, 128.01, 127.18, 126.29, 112.89, 104.88, 52.51, 20.16; **EI-MS** (m/z, %): 340 ($M^+$, 6.62), 84 (100), 86 (64.45), 47 (16.97); **HRMS** (EI): m/z calcd for $C_{24}H_{20}O_2$ $[M]^+$: 340.1463; found: 340.1470.

**Methyl 2-phenyl-4,4-di-p-tolylbuta-2,3-dienoate (5j)**

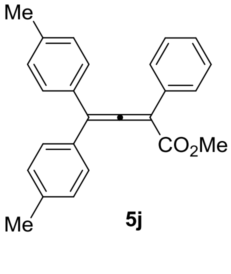


**5j**

White solid; TLC (PE:EA = 20:1): Rf = 0.3. **$^1$H NMR** ($CDCl_3$, 400 MHz) δ 7.64-7.57 (m, 2H), 7.34-7.26 (m, 7H), 7.18 (d, *J* = 8.0 Hz, 4H), 3.83 (s, 3H),

2.36 (s, 6H); **$^{13}$C NMR** ($CDCl_3$, 100 MHz) δ 214.65, 166.55, 138.29, 132.71, 131.85, 129.52, 128.67, 128.53, 128.33, 127.90, 114.57, 104.98, 52.59, 21.38; **EI-MS** (m/z, %): 354 ($M^+$, 0.71), 84 (100), 167 (63.96), 86 (63.93); **HRMS** (EI): m/z calcd for $C_{25}H_{22}O_2$ $[M]^+$: 354.1620; found: 354.1619.

**Methyl 4,4-bis(4-chlorophenyl)-2-phenylbuta-2,3-dienoate (5k)**

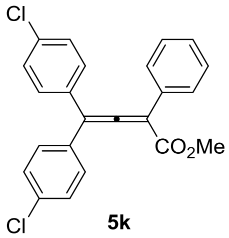


**5k**

White solid; TLC (PE:EA = 20:1): Rf = 0.3. **$^1$H NMR** ($CDCl_3$, 400 MHz) δ 7.56 (d, *J* = 7.1 Hz, 2H), 7.41-7.27 (m, 11H), 3.86 (s, 3H); **$^{13}$C NMR** ($CDCl_3$, 100 MHz) δ 214.27, 166.01, 134.58, 132.91, 131.88, 129.96, 129.21, 128.73, 128.39, 128.33, 113.20, 105.94, 52.86; **EI-MS** (m/z, %): 394 ($M^+$, 60.64), 335 (100), 139 (71.71), 265 (68.23); **HRMS** (EI): m/z calcd for $C_{23}H_{16}O_2Cl_2$ $[M]^+$: 394.0527; found: 394.0522.

**Methyl 3-(9H-fluoren-9-ylidene)-2-phenylacrylate (5l)**

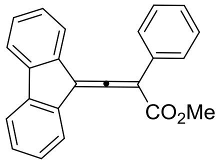


**5l**

White solid; TLC (PE:EA = 20:1): Rf = 0.3. **$^1$H NM**R ($CDCl_3$, 400 MHz) δ 7.69 (d, *J* = 7.5 Hz, 2H), 7.55 (d, *J* = 7.3 Hz, 4H), 7.44-7.20 (m, 7H), 3.74 (s, 3H); **$^{13}$C NMR** ($CDCl_3$, 100 MHz) δ 212.42, 165.63, 139.46, 136.52, 131.79, 129.00, 128.71, 128.62, 127.56, 123.82, 120.59, 110.33, 110.05, 52.84; **EI-MS** (m/z, %): 324 ($M^+$, 49.76), 265 (100), 324 (49.76), 204 (33.22); **HRMS** (EI): m/z calcd for $C_{23}H_{16}O_2$ $[M]^+$: 324.1150; found: 324.1157.

**Ethyl 2,4,4-triphenylbuta-2,3-dienoate (5m)**

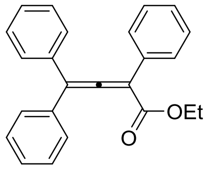


**5m**

White solid; TLC (PE:EA = 20:1): Rf = 0.3. **$^1$H NMR** ($CDCl_3$, 400 MHz) δ 7.64 (d, *J* = 7.4 Hz, 2H), 7.48-7.22 (m, 13H), 4.34 (q, *J* = 7.0 Hz, 2H), 1.36 (t, *J* = 7.0 Hz, 3H); **$^{13}$C NMR** ($CDCl_3$, 100 MHz) δ 214.49, 165.94, 134.95, 132.51, 128.82, 128.79, 128.57, 128.35, 128.35, 128.00, 114.75, 105.59, 61.49, 14.43; **EI-MS** (m/z, %): 340 ($M^+$, 7.67), 196 (100), 167 (67.53), 165 (54.29); **HRMS** (EI): m/z calcd for $C_{24}H_{20}O_2$ $[M]^+$: 340.1463; found: 340.1455.

**Benzyl 2,4,4-triphenylbuta-2,3-dienoate (5n)**

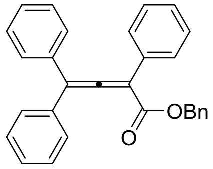


**5n**

White solid; TLC (PE:EA = 20:1): Rf = 0.3. **$^1$H NMR** ($CDCl_3$, 400 MHz) δ 7.63 (d, *J* = 7.3 Hz, 2H), 7.48-7.21 (m, 18H), 5.32 (s, 2H); **$^{13}$C NMR** ($CDCl_3$, 100 MHz) δ 214.80, 165.69, 136.13, 134.75, 132.28, 128.82, 128.79, 128.60, 128.59, 128.39, 128.35, 128.16, 128.09, 127.88, 114.99, 105.38, 66.88;

**EI-MS** (m/z, %): 402 ($M^+$, 10.01), 91 (100), 267 (59.94), 165 (42.64); **HRMS** (EI): m/z calcd for $C_{29}H_{22}O_2$ $[M]^+$: 402.1620; found: 402.1614.

**4,6,6-triphenylhexa-4,5-dien-3-one (5o)**

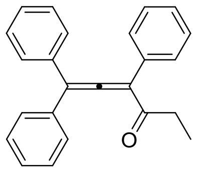

5o

White solid; TLC (PE:EA = 20:1): Rf = 0.3. **$^1$H NMR** ($CDCl_3$, 400 MHz) δ 7.50 (d, *J* = 7.1 Hz, 2H), 7.41-7.22 (m, 13H), 2.91 (q, *J* = 7.3 Hz, 2H), 1.14 (t, *J* = 7.3 Hz, 3H); **$^{13}$C NMR** ($CDCl_3$, 100 MHz) δ 215.55, 200.14, 134.50, 132.50, 129.03, 128.98, 128.51, 128.51, 128.47, 128.06, 114.71, 112.77, 34.32, 8.67; **EI-MS** (m/z, %): 402 ($M^+$, 10.01), 91 (100), 267 (59.94), 165 (42.64); **HRMS** (EI): m/z calcd for $C_{24}H_{20}O$ $[M]^+$: 324.1514; found: 324.1508.

**Methyl 4,4-diphenyl-2-(p-tolyl)buta-2,3-dienoate (5p)**

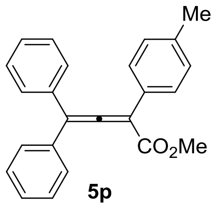

5p

White solid; TLC (PE:EA = 20:1): Rf = 0.3. **$^1$H NMR** ($CDCl_3$, 400 MHz) δ 7.50 (d, *J* = 8.1 Hz, 2H), 7.46-7.30 (m, 10H), 7.16 (d, *J* = 8.1 Hz, 2H), 3.84 (s, 3H), 2.34 (s, 3H); **$^{13}$C NMR** ($CDCl_3$, 100 MHz) δ 214.47, 166.58, 137.96, 134.88, 129.40, 129.32, 128.82, 128.81, 128.34, 128.22, 114.68, 105.21, 52.67, 21.36; **EI-MS** (m/z, %): 340 ($M^+$, 2.83), 84 (100), 86 (65.17), 47 (18.68); **HRMS** (EI): m/z calcd for $C_{24}H_{20}O_2$ $[M]^+$: 340.1463; found: 340.1467.

**Methyl 2-(4-methoxyphenyl)-4,4-diphenylbuta-2,3-dienoate (5q)**

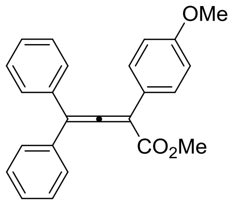

5q

White solid; TLC (PE:EA = 10:1): Rf = 0.3. **$^1$H NMR** ($CDCl_3$, 400 MHz) δ 7.55 (d, *J* = 8.8 Hz, 2H), 7.48-7.29 (m, 10H), 6.89 (d, *J* = 8.8 Hz, 2H), 3.84 (s, 3H), 3.80 (s, 3H); **$^{13}$C NMR** ($CDCl_3$, 100 MHz) δ 214.36, 166.70, 159.52, 134.99, 129.58, 128.82, 128.80, 128.32, 124.54, 114.68, 114.08, 104.89, 55.44, 52.64; **EI-MS** (m/z, %): 356 ($M^+$, 3.15), 121 (100), 180 (24.91), 77 (11.54); **HRMS** (EI): m/z calcd for $C_{24}H_{20}O_3$ $[M]^+$: 356.1412; found: 384.1401.

**Methyl 2-(4-fluorophenyl)-4,4-diphenylbuta-2,3-dienoate (5r)**

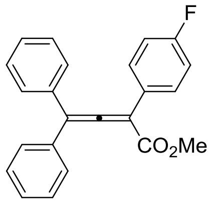

5r

White solid; TLC (PE:EA = 20:1): Rf = 0.3. **$^1$H NMR** ($CDCl_3$, 400 MHz) δ 7.62-7.56 (m, 2H), 7.44-7.32 (m, 10H), 7.04 (t, *J* = 8.7 Hz, 2H), 3.85 (s, 3H); **$^{13}$C NMR** ($CDCl_3$, 100 MHz) δ 214.58, 166.36, 163.83 (d, *J* = 246.4 Hz), 161.36, 134.63, 130.16, 130.08 (d, *J* = 8 Hz), 128.90, 128.78, 128.51, 128.41,

128.37 (d, *J* = 3.5 Hz), 115.65, 115.44 (d, *J* = 21.5 Hz), 114.99, 104.36, 52.73; **$^{19}$F NMR** (376 MHz, $CDCl_3$) δ -113.94; **EI-MS** (m/z, %): 344 ($M^+$, 65.95), 285 (100), 105 (96.07), 283 (63.95); **HRMS** (EI): m/z calcd for $C_{23}H_{17}FO_2$ $[M]^+$: 344.1213; found: 344.1208.

**Methyl 2-(4-chlorophenyl)-4,4-diphenylbuta-2,3-dienoate (5s)**

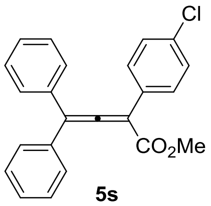


5s

White solid; TLC (PE:EA = 20:1): Rf = 0.3. **$^1$H NMR** ($CDCl_3$, 400 MHz) δ 7.57 (d, *J* = 8.5 Hz, 2H), 7.46-7.27 (m, 12H), 3.86 (s, 3H); **$^{13}$C NMR** ($CDCl_3$, 100 MHz) δ 214.77, 166.16, 134.49, 133.96, 130.94, 129.65, 128.92, 128.80, 128.78, 128.58, 115.24, 104.36, 52.76; **EI-MS** (m/z, %): 360 ($M^+$, 52.19), 105 (100), 301 (71.32), 265 (68.21); **HRMS** (EI): m/z calcd for $C_{23}H_{17}ClO_2$ $[M]^+$: 360.0917; found: 360.0910.

**Methyl 4,4-diphenyl-2-(4-(trifluoromethyl)phenyl)buta-2,3-dienoate (5t)**

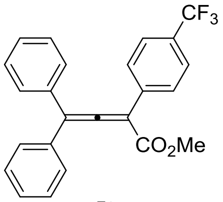


5t

White solid; TLC (PE:EA = 20:1): Rf = 0.3. **$^1$H NMR** ($CDCl_3$, 400 MHz) δ 7.75 (d, *J* = 8.2 Hz, 2H), 7.60 (d, *J* = 8.3 Hz, 2H), 7.45-7.33 (m, 10H), 3.87 (s, 3H); **$^{13}$C NMR** ($CDCl_3$, 100 MHz) δ 215.30, 165.94, 136.34, 134.24, 128.99, 128.82, 128.74, 128.65, 125.58, 125.54, 125.51, 125.47 (q, *J* = 3.0 Hz), 115.56, 104.34, 52.86.; **$^{19}$F NMR** (376 MHz, $CDCl_3$) δ -66.63; **EI-MS** (m/z, %): 394 ($M^+$); **HRMS** (EI): m/z calcd for $C_{24}H_{17}O_2F_3$ $[M]^+$: 394.1181; found: 394.1180.

**Methyl 4-(1-methoxy-1-oxo-4,4-diphenylbuta-2,3-dien-2-yl)benzoate (5u)**

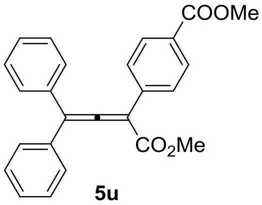


5u

White solid; TLC (PE:EA = 8:1): Rf = 0.3. **$^1$H NMR** ($CDCl_3$, 400 MHz) δ 8.02 (d, *J* = 8.4 Hz, 2H), 7.71 (d, *J* = 8.4 Hz, 2H), 7.45-7.33 (m, 10H), 3.91 (s, 3H), 3.87 (s, 3H); **$^{13}$C NMR** ($CDCl_3$, 100 MHz) δ 215.38, 166.91, 165.98, 137.23, 134.33, 129.86, 129.54, 128.96, 128.83, 128.67, 128.24, 115.47, 104.73, 52.81, 52.27. **EI-MS** (m/z, %): 384 ($M^+$, 23.40), 163 (100), 105 (32.69), 325 (27.24); **HRMS** (EI): m/z calcd for $C_{25}H_{20}O_4$ $[M]^+$: 384.1362; found: 384.1351.

**Methyl 4,4-diphenyl-2-(m-tolyl)buta-2,3-dienoate (5v)**

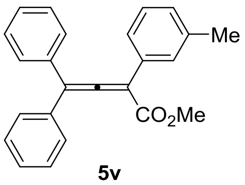


5v

White solid; TLC (PE:EA = 20:1): Rf = 0.3. **$^1$H NMR** ($CDCl_3$, 400 MHz) δ 7.30-7.45 (m, 12H), 7.24 (t, *J* = 7.6 Hz, 1H), 7.09 (d, *J* = 7.6 Hz, 1H), 3.84 (s, 3H), 2.34 (s, 3H); **$^{13}$C NMR** ($CDCl_3$, 100 MHz) δ 214.49, 166.51, 138.18, 134.85, 132.33, 128.94, 128.88, 128.82, 128.81, 128.48, 128.35,

125.47, 114.67, 105.38, 52.63, 21.67; **EI-MS** (m/z, %): 340 ($M^+$, 2.83), 84 (100), 86 (65.17), 47 (18.68); **HRMS** (EI): m/z calcd for $C_{24}H_{20}O_2$ $[M]^+$: 340.1463; found: 340.1459.

**Methyl 4,4-diphenyl-2-(3-(trifluoromethyl)phenyl)buta-2,3-dienoate (5w)**

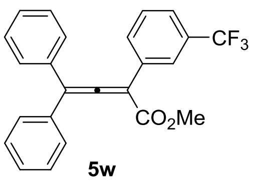

**5w**

White solid; TLC (PE:EA = 20:1): Rf = 0.3. **$^1$H NMR** ($CDCl_3$, 400 MHz) δ 7.96 (s, 1H), 7.79 (d, *J* = 7.6 Hz, 1H), 7.56 (d, *J* = 7.6 Hz, 1H), 7.48-7.34 (m, 11H), 3.89 (s, 3H); **$^{13}$C NMR** ($CDCl_3$, 100 MHz) δ 214.96, 165.99, 134.31, 133.52, 131.66, 131.50, 131.18, 130.85, 130.53 (q, *J* = 32.1 Hz), 129.03, 128.99, 128.82, 128.71, 125.24, 125.20, 125.16, 125.12 (d, *J* = 3.6 Hz), 124.83, 124.79, 124.75, 124.72 (d, *J* = 3.6 Hz), 124.06 (d, *J* = 271.0 Hz), 115.63, 104.24, 52.85; **$^{19}$F NMR** (376 MHz, $CDCl_3$) δ -62.66; **EI-MS** (m/z, %): 394 ($M^+$, 7.05), 191 (100), 105 (47.91), 250 (28.04); **HRMS** (EI): m/z calcd for $C_{25}H_{17}F_3O_2$ $[M]^+$: 394.1181; found: 394.1184.

**Methyl 4,4-diphenyl-2-(thiophen-2-yl)buta-2,3-dienoate (5y)**

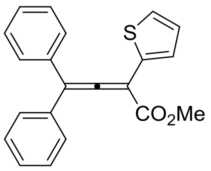

**5y**

White solid; TLC (PE:EA = 20:1): Rf = 0.3. **$^1$H NMR** ($CDCl_3$, 400 MHz) δ 7.89 (dd, *J* = 2.7, 1.3 Hz, 1H), 7.44-7.32 (m, 9H), 7.28-7.22 (m, 3H), 3.85 (s, 3H); **$^{13}$C NMR** ($CDCl_3$, 100 MHz) δ 215.05, 166.28, 134.76, 131.77, 128.87, 128.87, 128.46, 127.36, 125.32, 123.81, 115.08, 101.11, 52.66; **EI-MS** (m/z, %): 332 ($M^+$, 8.98), 84 (100), 86 (62.13), 47 (32.21); **HRMS** (EI): m/z calcd for $C_{21}H_{26}SO_2$ $[M]^+$: 332.0871; found: 332.0876.

**Methyl 2-(naphthalen-2-yl)-4,4-diphenylbuta-2,3-dienoate (5z)**

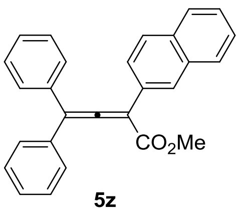

**5z**

White solid; TLC (PE:EA = 20:1): Rf = 0.3. **$^1$H NMR** ($CDCl_3$, 400 MHz) δ 8.23 (s, 1H), 7.86-7.77 (m, 3H), 7.64 (d, *J* = 8.5 Hz, 1H), 7.48-7.44 (m, 6H), 7.42-7.34 (m, 6H), 3.90 (s, 3H); **$^{13}$C NMR** ($CDCl_3$, 100 MHz) δ 215.18, 166.52, 134.75, 133.54, 132.98, 129.57, 128.89, 128.86, 128.57, 128.48, 128.17, 127.67, 127.46, 126.40, 126.33, 126.05, 115.15, 105.39, 52.76; **EI-MS** (m/z, %): 376 ($M^+$, 10.01), 84 (100), 86 (63.29), 57 (49.28); **HRMS** (EI): m/z calcd for $C_{27}H_{20}O_2$ $[M]^+$: 376.1463; found: 376.1466.

**1,1,3,3-tetraphenylpropa-1,2-diene (7a)**

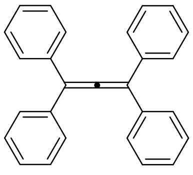
**7a**

White solid; TLC (PE:EA = 100:1): Rf = 0.3. **$^1$H NMR** ($CDCl_3$, 400 MHz) δ 7.46-7.40 (m, 8H), 7.35 (t, *J* = 7.3 Hz, 8H), 7.30 (d, *J* = 7.1 Hz, 4H); **$^{13}$C NMR** ($CDCl_3$, 100 MHz) δ 208.60, 136.46, 128.66, 128.57, 127.64, 112.76; **EI-MS** (m/z, %): 344 ($M^+$, 100), 265 (43.51), 267 (42.50), 345 (28.57); **HRMS** (EI):

m/z calcd for $C_{27}H_{20}$ $[M]^+$: 344.1565; found: 344.1560.

**(3-(*p*-tolyl)propa-1,2-diene-1,1,3-triyl)tribenzene (7b and 7i)**

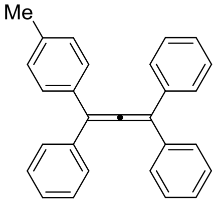


**7b and 7i**

White solid; TLC (PE:EA = 100:1): Rf = 0.3. **$^1$H NMR** ($CDCl_3$, 400 MHz) δ 7.42 (d, *J* = 7.2 Hz, 6H), 7.37-7.25 (m, 11H), 7.16 (d, *J* = 7.9 Hz, 2H), 2.36 (s, 3H); **$^{13}$C NMR** ($CDCl_3$, 100 MHz) δ 208.50, 137.46, 136.63, 136.59, 133.43, 129.38, 128.63, 128.62, 128.56, 128.56, 128.46, 127.57, 127.57, 112.62, 112.59, 21.36; **EI-MS** (m/z, %): 358 ($M^+$, 8.27), 214 (100), 165 (83.89), 257 (79.06); **HRMS** (EI): m/z calcd for $C_{28}H_{22}$ $[M]^+$: 358.1722; found: 358.1721.

**(3-(4-chlorophenyl)propa-1,2-diene-1,1,3-triyl)tribenzene (7c and 7k)**

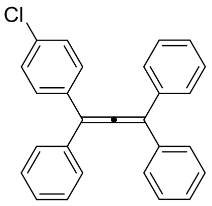


**7c and 7k**

White solid; TLC (PE:EA = 100:1): Rf = 0.3. **$^1$H NMR** ($CDCl_3$, 400 MHz) δ 7.42-7.38 (m, 6H), 7.36-7.26 (m, 13H); **$^{13}$C NMR** ($CDCl_3$, 100 MHz) δ 208.54, 136.19, 136.05, 135.05, 133.45, 129.80, 128.86, 128.78, 128.73, 128.55, 128.49, 127.85, 127.80, 113.12, 111.93; **EI-MS** (m/z, %): 378 ($M^+$, 36.32), 377 (100), 265 (96.32), 263 (54.44); **HRMS** (EI): m/z calcd for $C_{27}H_{19}Cl$ $[M]^+$: 378.1175; found: 378.1171.

**(3-(4-methoxyphenyl)propa-1,2-diene-1,1,3-triyl)tribenzene (7d)**

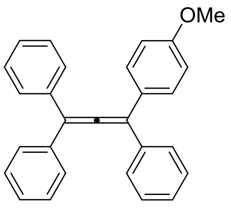


**7d**

White solid; TLC (PE:EA = 20:1): Rf = 0.3. **$^1$H NMR** ($CDCl_3$, 400 MHz) δ 7.48 (d, *J* = 6.9 Hz, 6H), 7.44-7.27 (m, 11H), 6.94 (d, *J* = 8.6 Hz, 2H), 3.84 (s, 3H).; **$^{13}$C NMR** ($CDCl_3$, 100 MHz) δ 208.42, 159.27, 136.74, 136.65, 136.65, 129.70, 128.63, 128.63, 128.54, 128.54, 127.59, 127.55, 114.11, 112.53, 112.36, 55.42; **EI-MS** (m/z, %): 374($M^+$, 11.78), 84 (100), 86 (66.16), 57 (23.08); **HRMS** (EI): m/z calcd for $C_{28}H_{22}O$ $[M]^+$: 374.1671; found: 374.1665.

**(3-(*m*-tolyl)propa-1,2-diene-1,1,3-triyl)tribenzene (7e and 7m)**

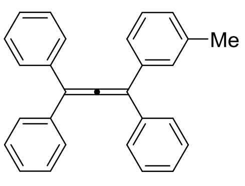


**7e and 7m**

White solid; TLC (PE:EA = 100:1): Rf = 0.3. **$^1$H NMR** ($CDCl_3$, 400 MHz) δ 7.44-7.40 (m, 6H), 7.31-7.37 (m, 6H), 7.30-7.25 (m, 3H), 7.24-7.20 (m, 3H), 7.13-7.07(s, 1H), 2.32 (s, 3H); **$^{13}$C NMR** ($CDCl_3$, 100 MHz) δ 208.55, 138.26, 136.61, 136.54, 136.36, 129.13, 128.64, 128.57, 128.46, 128.40, 127.60, 127.58, 127.43, 125.75, 112.78, 112.62, 21.65; **EI-MS** (m/z, %): 358

($M^+$, 22.81), 167 (100), 265 (42.02), 165 (40.01); **HRMS** (EI): m/z calcd for $C_{28}H_{22}$ [M]+: 358.1722; found: 358.1720.

**(3-(3-chlorophenyl)propa-1,2-diene-1,1,3-triyl)tribenzene (7f)**

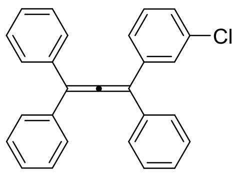

7f

White solid; TLC (PE:EA = 100:1): Rf = 0.3. **$^1$H NMR** ($CDCl_3$, 400 MHz) δ 7.22-7.44 (m, 19H); **$^{13}$C NMR** ($CDCl_3$, 100 MHz) δ 208.58, 138.57, 136.11, 135.91, 134.59, 129.90, 128.82, 128.75, 128.58, 128.50, 128.40, 127.90, 127.86, 127.75, 126.74, 113.29, 111.86; **EI-MS** (m/z, %): 378 ($M^+$, 6.56), 49 (100), 84 (73.69), 57 (59.46); **HRMS** (EI): m/z calcd for $C_{27}H_{19}Cl$ $[M]^+$: 378.1175; found: 378.1168.

**(3-(*o*-tolyl)propa-1,2-diene-1,1,3-triyl)tribenzene (7g and 7n)**

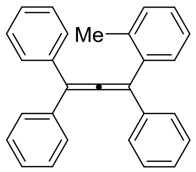

7g and 7n

White solid; TLC (PE:EA = 100:1): Rf = 0.3. **$^1$H NMR** ($CDCl_3$, 400 MHz) δ 7.50-7.17 (m, 19H), 2.19 (s, 3H); **$^{13}$C NMR** ($CDCl_3$, 100 MHz) δ 207.09, 137.22, 136.56, 136.27, 135.81, 130.48, 130.39, 128.77, 128.68, 128.59, 127.93, 127.62, 127.33, 126.84, 126.19, 112.61, 110.79, 20.44; **EI-MS** (m/z, %): 358 ($M^+$, 19.50), 167 (100), 265 (43.25), 165 (29.24); **HRMS** (EI): m/z calcd for $C_{28}H_{22}$ $[M]^+$: 358.1722; found: 358.1721.

**(3-(2-chlorophenyl)propa-1,2-diene-1,1,3-triyl)tribenzene (7h)**

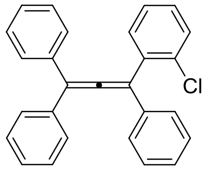

7h

White solid; TLC (PE:EA = 100:1): Rf = 0.3. **$^1$H NMR** ($CDCl_3$, 400 MHz) δ 7.49-7.15 (m, 19H); **$^{13}$C NMR** ($CDCl_3$, 100 MHz) δ 207.45, 136.04, 135.52, 135.33, 134.44, 131.96, 130.05, 129.24, 128.78, 128.62, 127.78, 127.46, 127.15, 126.70, 113.54, 109.93. **EI-MS** (m/z, %): 378 ($M^+$, 35.95), 377 (100), 265 (92.10), 263 (53.11); **HRMS** (EI): m/z calcd for $C_{27}H_{19}Cl$ $[M]^+$: 378.1175; found: 378.1180.

**(3-(4-fluorophenyl)propa-1,2-diene-1,1,3-triyl)tribenzene (7j)**

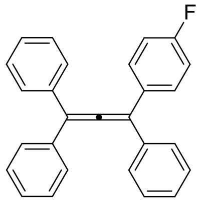

7j

White solid; TLC (PE:EA = 100:1): Rf = 0.3. **$^1$H NMR** ($CDCl_3$, 400 MHz) δ 7.47-7.25 (m, 17H), 7.03 (t, *J* = 8.5 Hz, 2H); **$^{13}$C NMR** ($CDCl_3$, 100 MHz) δ 208.41, 162.44 (q, *J* = 245.9 Hz), 136.36, 132.46 (d, *J* = 3 Hz), 130.18 (d, *J* = 8 Hz), 128.75, 128.71, 128.55, 128.45, 127.79, 127.74, 115.61 (d, *J* = 21.5 Hz), 112.92, 111.93; **$^{19}$F NMR** (376 MHz, $CDCl_3$) δ -114.67. **EI-MS** (m/z, %): 362 ($M^+$, 89.16), 105 (100), 57 (59.88), 77(58.16); **HRMS** (EI): m/z calcd for

$C_{27}H_{19}F$ $[M]^+$: 362.1471; found: 362.1467.

**(3-(4-(trifluoromethyl)phenyl)propa-1,2-diene-1,1,3-triyl)tribenzene (7l)**

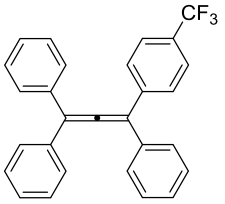


7l

White solid; TLC (PE:EA = 100:1): Rf = 0.3. **$^1$H NMR** ($CDCl_3$, 400 MHz) δ 7.60 (d, *J* = 8.3 Hz, 2H), 7.53 (d, *J* = 8.2 Hz, 2H), 7.43-7.28 (m, 15H); **$^{13}$C NMR** ($CDCl_3$, 100 MHz) δ 208.95, 140.50, 135.97, 135.76, 130.16, 129.81, 129.49, 129.16 (q, *J* = 32.1 Hz), 128.88, 128.79, 128.74, 128.57, 128.51, 128.01, 127.96, 125.71, 125.67, 125.63, 125.60 (q, *J* = 3.6 Hz), 124.133 (q, *J* = 230.6 Hz), 113.50, 111.97; **$^{19}$F NMR** (376 MHz, $CDCl_3$) δ -62.50; **EI-MS** (m/z, %): 412($M^+$, 100), 265 (39.41), 57 (31.50), 335(27.51); **HRMS** (EI): m/z calcd for $C_{28}H_{19}F_3$ $[M]^+$: 412.1435; found: 412.1439.

**4,4'-(3,3-diphenylpropa-1,2-diene-1,1-diyl)bis(methylbenzene) (7o)**

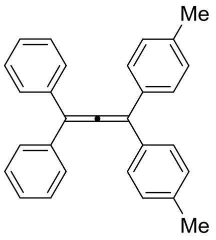


7o

White solid; TLC (PE:EA = 100:1): Rf = 0.3. **$^1$H NMR** ($CDCl_3$, 400 MHz) δ 7.43-7.39 (m, 4H), 7.38-7.27 (m, 10H), 7.15 (d, *J* = 8.0 Hz, 4H), 2.36 (s, 6H); **$^{13}$C NMR** ($CDCl_3$, 100 MHz) δ 208.41, 137.38, 136.71, 133.59, 129.33, 128.59, 128.56, 128.45, 127.49, 112.48, 112.42, 21.35; **EI-MS** (m/z, %): 372($M^+$, 31.45), 84 (100), 86 (67.44), 47(17.35); **HRMS** (EI): m/z calcd for $C_{29}H_{24}$ $[M]^+$: 372.1870; found: 372.1878.

**4,4'-(3,3-diphenylpropa-1,2-diene-1,1-diyl)bis(fluorobenzene) (7p)**

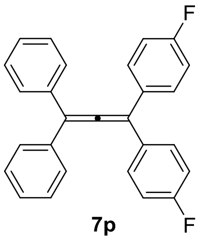


7p

White solid; TLC (PE:EA = 100:1): Rf = 0.3. **$^1$H NMR** ($CDCl_3$, 400 MHz) δ 7.43-7.28 (m, 14H), 7.07 (t, *J* = 8.6 Hz, 4H); **$^{13}$C NMR** ($CDCl_3$, 100 MHz) δ 208.24, 163.72, 161.26 (q, *J* = 245.9 Hz), 136.23, 132.35, 132.32 (q, *J* = 33 Hz), 130.11, 130.03 (q, *J* = 8 Hz), 128.76, 128.52, 127.84, 115.82, 115.60 (q, *J* = 21.5 Hz), 113.07, 111.09; **$^{19}$F NMR** (376 MHz, $CDCl_3$) δ -114.32; **EI-MS** (m/z, %): 380 ($M^+$, 4.62), 84 (100), 86 (64.22), 214 (32.81); **HRMS** (EI): m/z calcd for $C_{27}H_{18}F_2$ $[M]^+$: 380.1377; found: 380.1373.

**Methyl 2,4,4-triphenyl-3-(4,4,5,5-tetramethyl-1,3,2-dioxaborolan-2-yl)but-3-enoate (8a)**

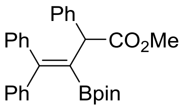


8a

White solid; TLC (PE:EA = 5:1): Rf = 0.3. **$^1$H NMR** ($CDCl_3$, 400 MHz) δ 7.32-7.14 (m, 13H), 7.10-7.04 (m, 2H), 4.75 (s, 1H), 3.67 (s, 3H), 1.05 (d, *J* = 38.4 Hz, 12H); **$^{13}$C NMR** ($CDCl_3$, 100 MHz) δ 173.91, 153.66, 143.88, 141.93, 139.48, 129.37, 128.94, 128.91, 128.43, 128.22, 127.88, 127.37, 127.25, 126.79, 83.55, 54.94,

52.21, 25.10, 24.88; **EI-MS** (m/z, %): 454 ($M^+$,4.32), 84 (100), 86 (67.33); **HRMS** (EI): m/z calcd for $C_{29}H_{31}BO_4$ $[M]^+$: 454.2315; found: 454.2316.

**3-ethyl-2,4-diphenylnaphthalen-1-ol (8b)**

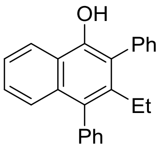


**8b**

White solid; TLC (PE:EA = 20:1): Rf = 0.3. **$^1$H NMR** ($CDCl_3$, 400 MHz) δ 8.29-8.22 (m, 1H), 7.59-7.23 (m, 13H), 5.18 (s, 1H), 2.35 (q, *J* = 7.5 Hz, 2H), 0.72 (t, *J* = 7.5 Hz, 3H); **$^{13}$C NMR** ($CDCl_3$, 100 MHz) δ 147.91, 140.13, 138.49, 135.61, 133.76, 131.25, 131.11, 130.87, 129.60, 128.52, 128.32, 126.99, 126.41, 126.34, 124.64, 122.37, 122.25, 24.75, 15.34; **EI-MS** (m/z, %): 324 ($M^+$,77.25), 308 (100), 77 (67.33); **HRMS** (EI): m/z calcd for $C_{24}H_{20}O_2$ [M]+: 324.1514; found: 324.1512.

**2,4,4-triphenylbuta-2,3-dienoic acid (8c)**

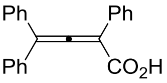


**8c**

White solid; TLC (PE:EA = 5:1): Rf = 0.3. **$^1$H NMR** ($CDCl_3$, 400 MHz) δ 8.03 (s, 1H), 7.94-7.87 (m, 2H), 7.45-7.30 (m, 13H); **$^{13}$C NMR** ($CDCl_3$, 100 MHz) δ 224.32, 170.99, 150.45, 139.88, 129.93, 129.69, 129.36, 128.87, 128.83, 128.74, 127.43, 126.83, 99.74, 89.26; **EI-MS** (m/z, %): 312 ($M^+$,7.32), 84 (100), 91 (67.33); **HRMS** (EI): m/z calcd for $C_{22}H_{16}O_2$ $[M]^+$: 312.1150; found: 312.1152.

**Pd Complex (9)**

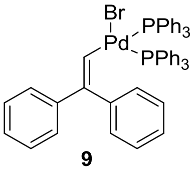


**9**

Yellow solid; **$^1$H NMR** ($CDCl_3$, 400 MHz) δ 7.59-7.50 (m, 12H), 7.40-7.16 (m, 24H), 7.02 (d, *J* = 3.8 Hz, 3H), 6.54 (d, *J* = 3.8 Hz, 2H), 6.44 (t, *J* = 10.1 Hz, 1H); **$^{13}$C NMR** ($CDCl_3$, 100 MHz) δ 153.65, 144.54, 131.54, 131.32, 131.09, 130.09, 129.69, 128.01, 127.96, 127.91, 127.37, 126.97, 126.41, 125.05; **$^{31}$P NMR** (162 MHz, $CDCl_3$) δ 22.41; **HRMS** (EI): m/z calcd for $C_{50}H_{41}P_2Pd^+$ $[M\text{-}Br]^+$: 809.1718; found: 809.1729.

**Methyl 2,4,4-triphenylbut-3-enoate (10)**

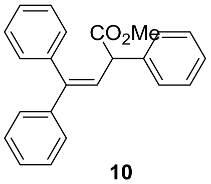


**10**

White solid; TLC (PE:EA = 20:1): Rf = 0.3. **$^1$H NMR** ($CDCl_3$, 400 MHz) δ 7.34-7.14 (m, 13H), 7.05 (dd, *J* = 7.6, 1.7 Hz, 2H), 6.51 (d, *J* = 10.4 Hz, 1H), 4.36 (d, *J* = 10.4 Hz, 1H), 3.61 (s, 3H); **$^{13}$C NMR** ($CDCl_3$, 100 MHz) δ 247.03, 172.28, 142.90, 140.83, 138.29, 138.20, 128.88, 127.95, 127.52, 127.29, 126.90, 126.70, 126.64, 126.38, 124.73, 51.42, 50.72; **EI-MS** (m/z, %): 380 ($M^+$,4.62), 84 (100), 86 (64.22), 214 (32.81); **HRMS** (EI): m/z calcd for $C_{23}H_{20}O_2$ $[M]^+$: 328.1463; found: 328.1458.

# 5. Copies of $^{1}$H NMR, $^{13}$C NMR, $^{19}$F NMR and $^{31}$P NMR Spectras

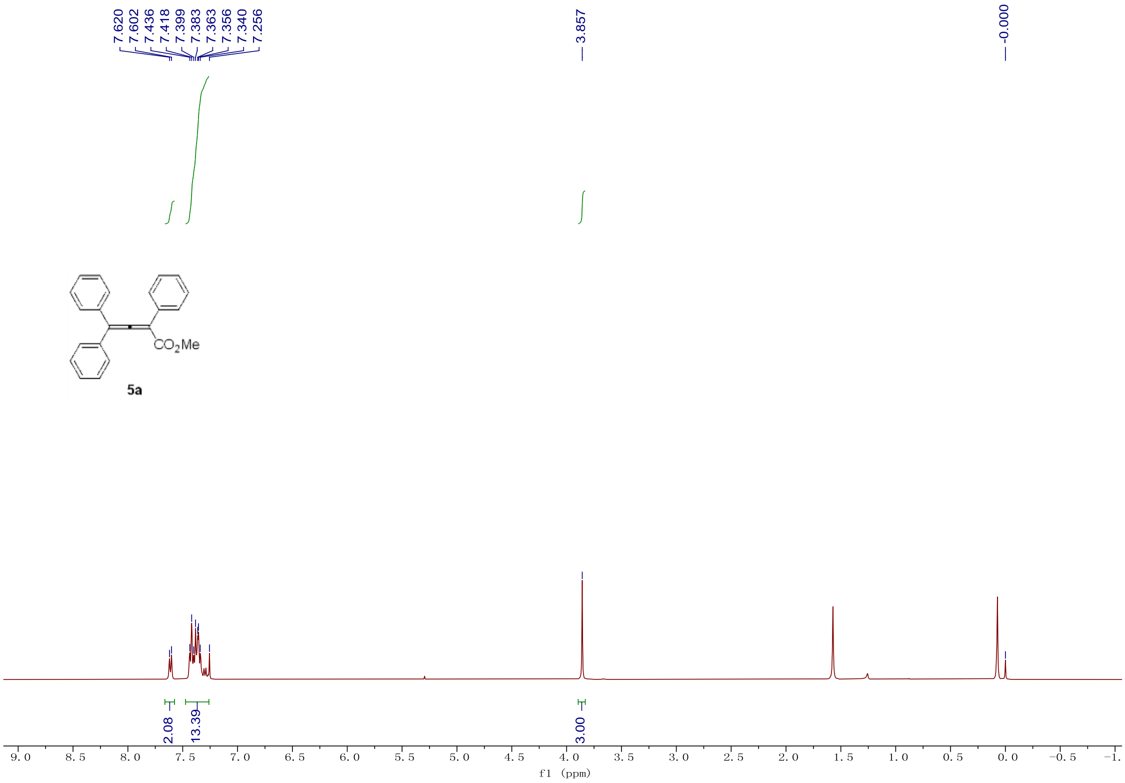


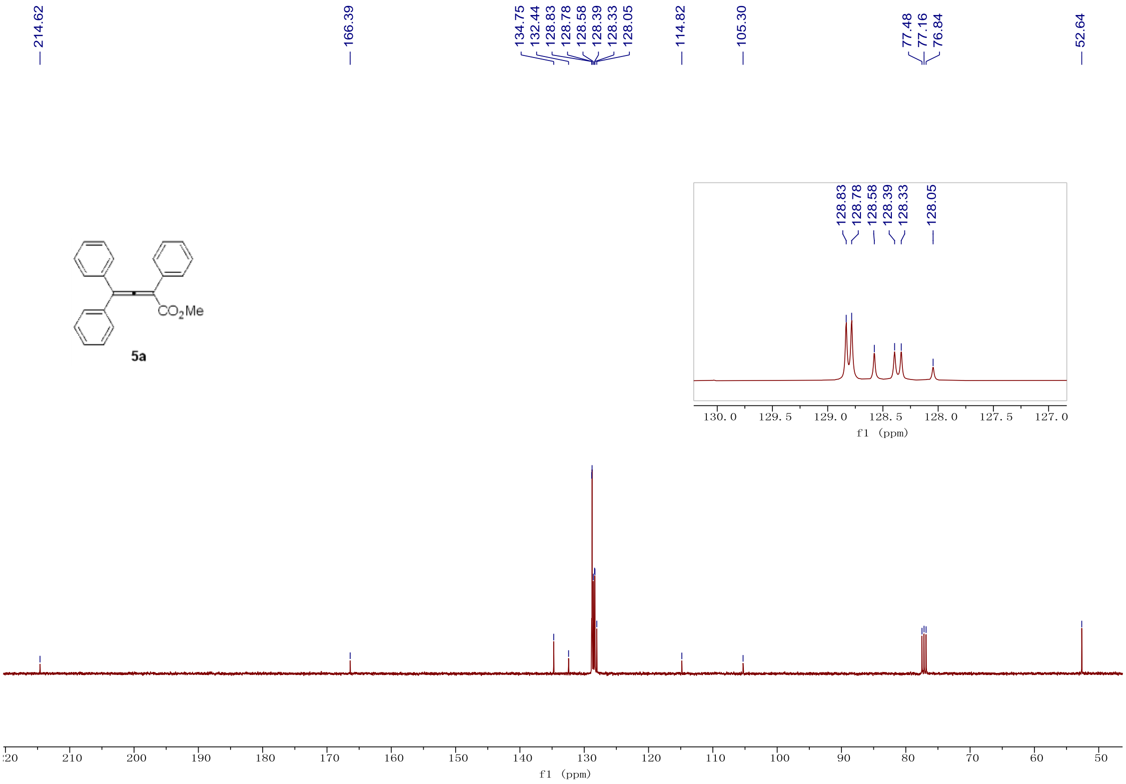

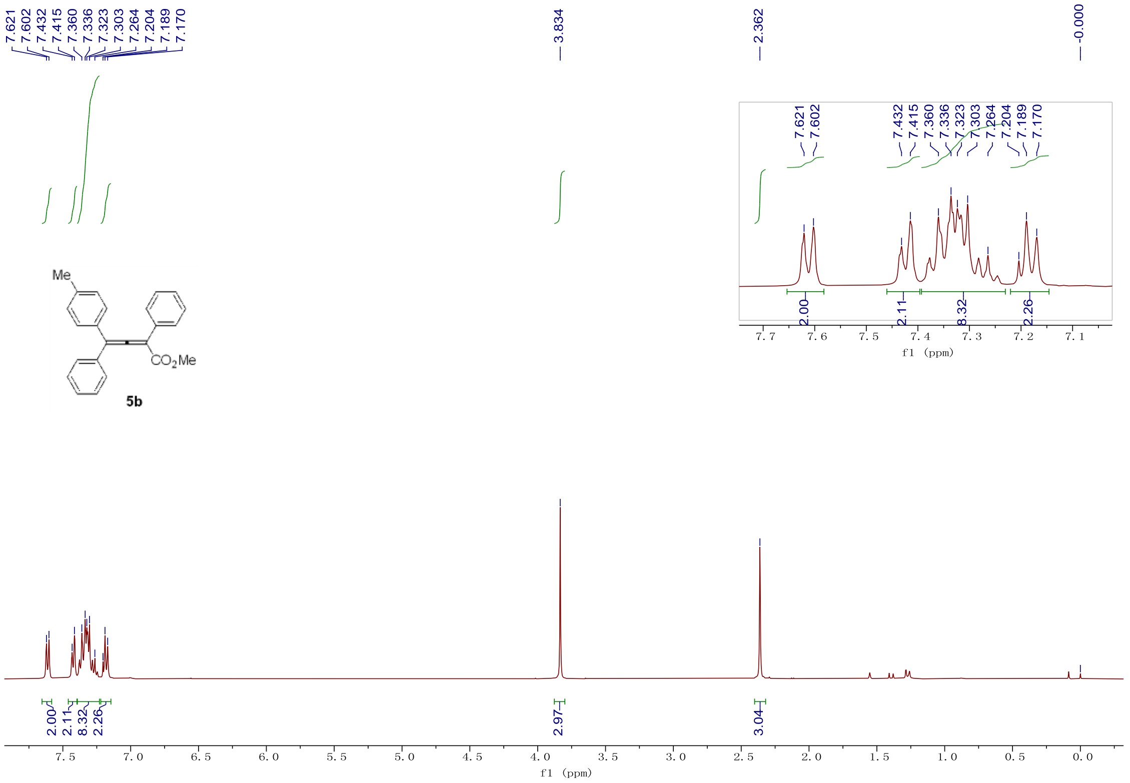
7.621
7.602
7.432
7.415
7.360
7.336
7.323
7.303
7.264
7.204
7.189
7.170
3.834
2.362
-0.000
Me
$CO_2Me$
5b
2.00
2.11
8.32
2.26
2.97
3.04
f1 (ppm)

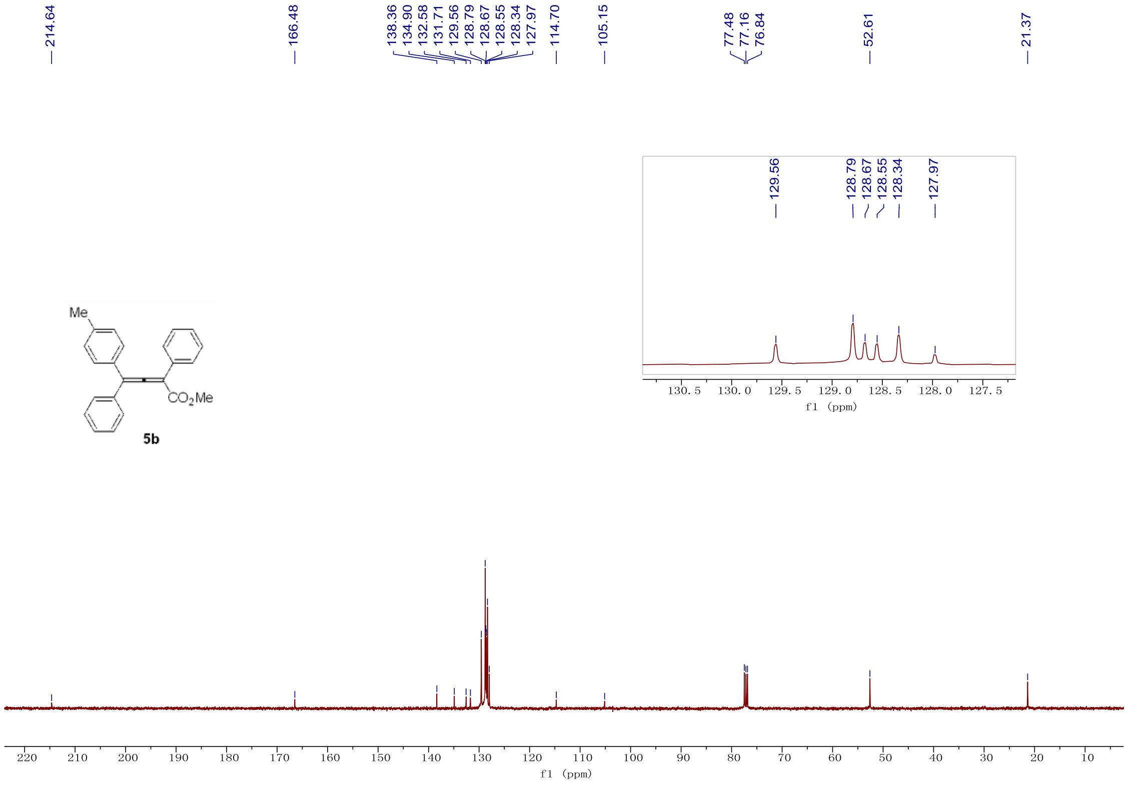
214.64
166.48
138.36
134.90
132.58
131.71
129.56
128.79
128.67
128.55
128.34
127.97
114.70
105.15
77.48
77.16
76.84
52.61
21.37
Me
$CO_2Me$
5b
f1 (ppm)

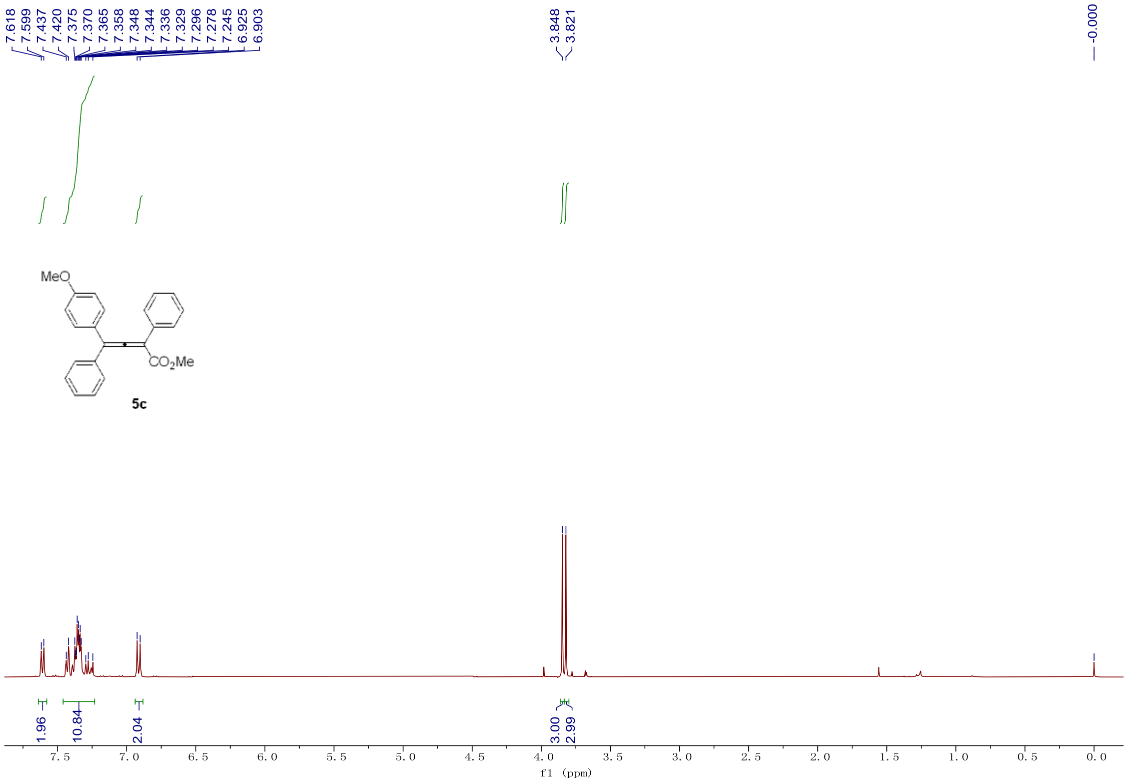
7.618
7.599
7.437
7.420
7.375
7.370
7.365
7.358
7.348
7.344
7.336
7.329
7.296
7.278
7.245
6.925
6.903
3.848
3.821
-0.000
MeO
CO2Me
5c
1.96
10.84
2.04
3.00
2.99
7.5 7.0 6.5 6.0 5.5 5.0 4.5 4.0 3.5 3.0 2.5 2.0 1.5 1.0 0.5 0.0
f1 (ppm)

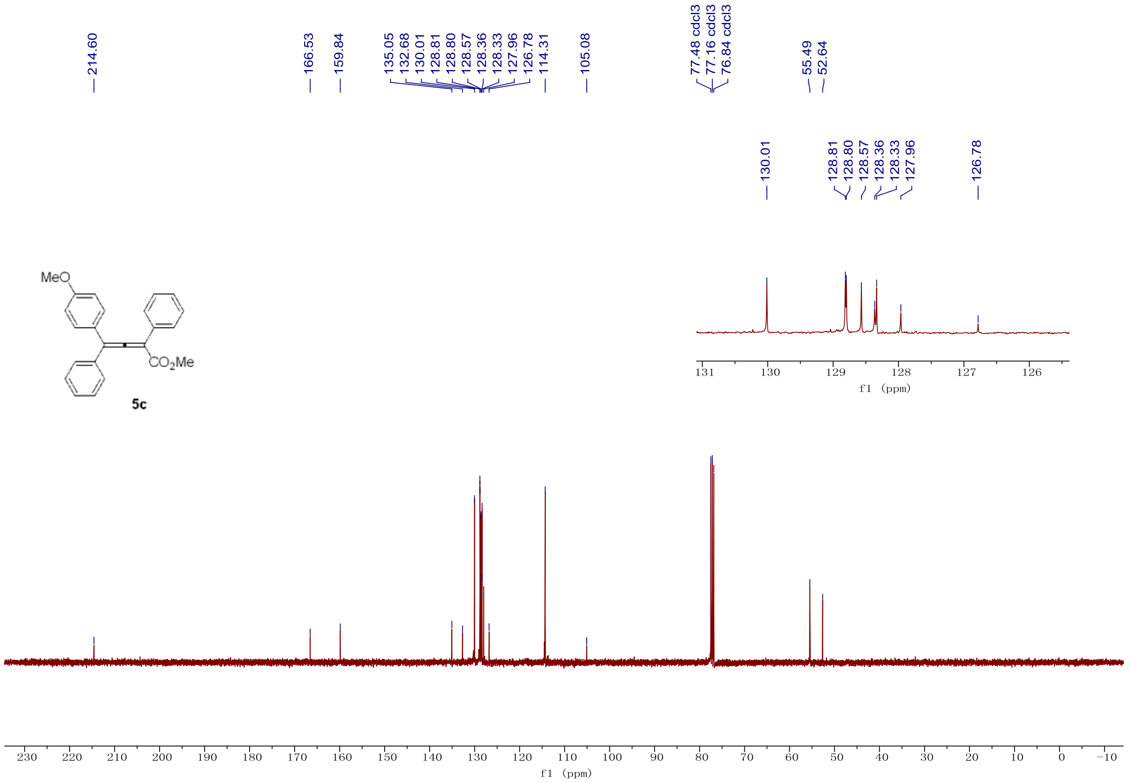
214.60
166.53
159.84
135.05
132.68
130.01
128.81
128.80
128.57
128.36
128.33
127.96
126.78
114.31
105.08
77.48 cdcl3
77.16 cdcl3
76.84 cdcl3
55.49
52.64
MeO
CO2Me
5c
131 130 129 128 127 126
f1 (ppm)
230 220 210 200 190 180 170 160 150 140 130 120 110 100 90 80 70 60 50 40 30 20 10 0 -10
f1 (ppm)

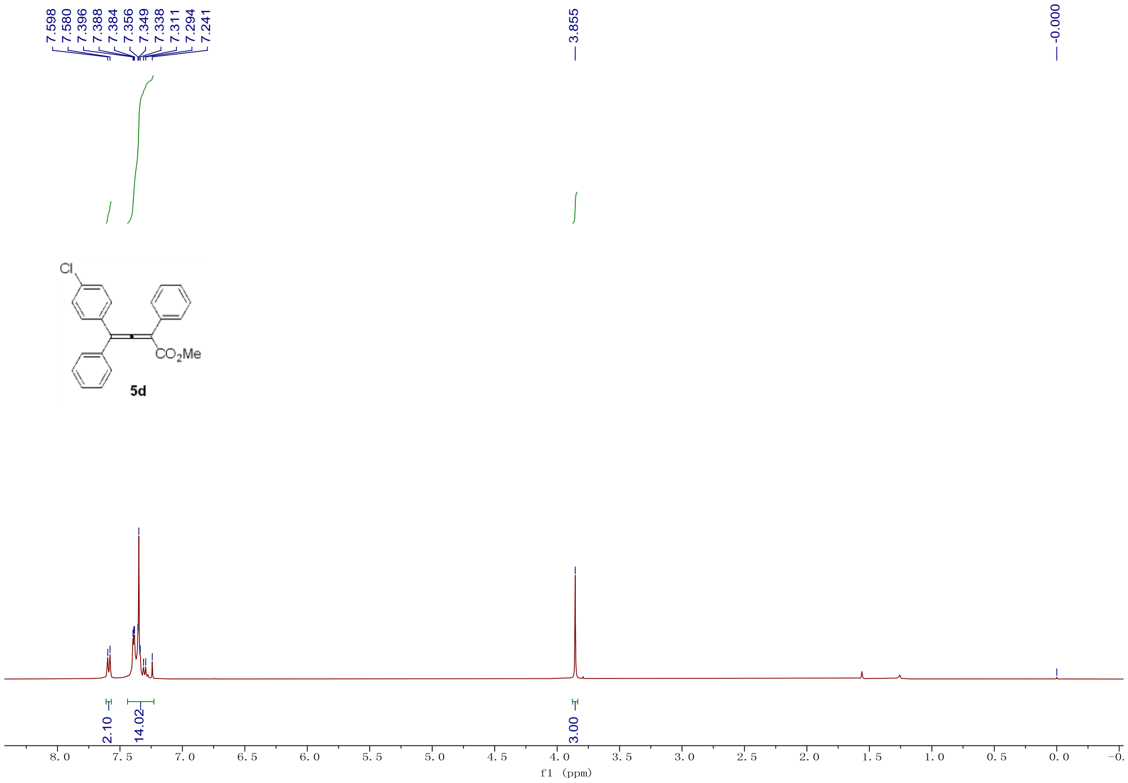
7.598
7.580
7.396
7.388
7.384
7.356
7.349
7.338
7.311
7.294
7.241
3.855
-0.000
Cl
CO2Me
5d
2.10
14.02
3.00
f1 (ppm)

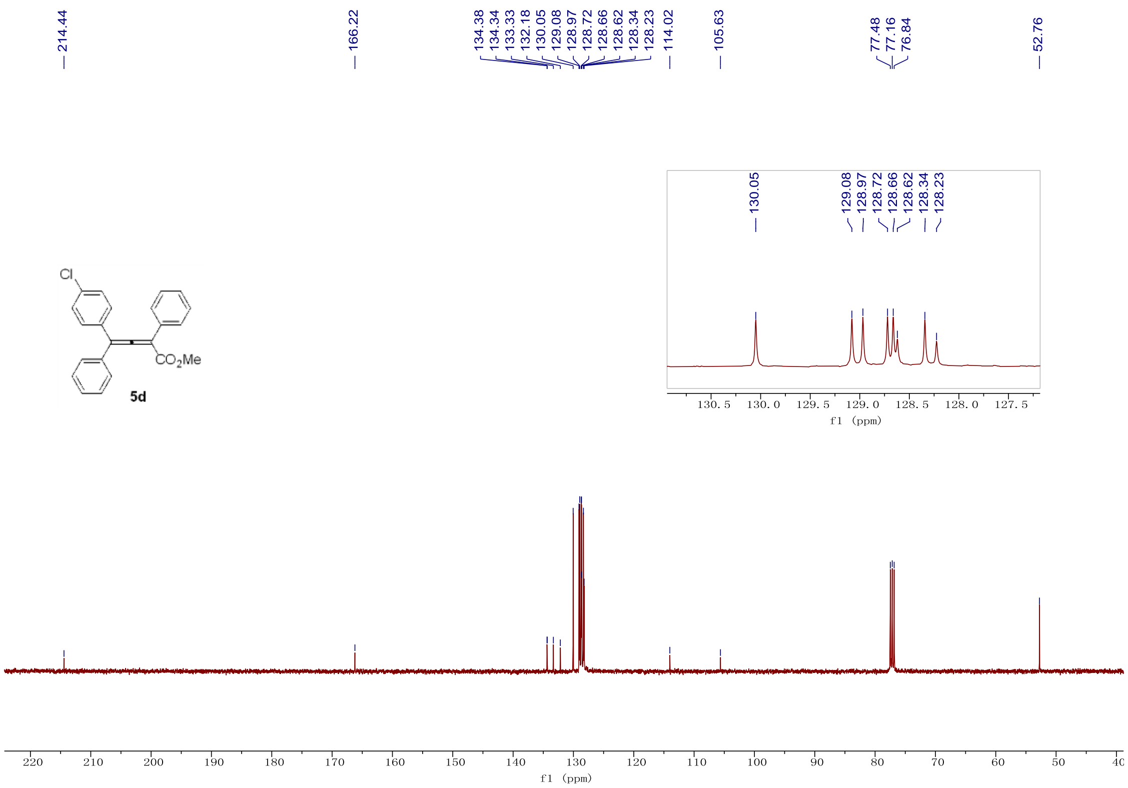
214.44
166.22
134.38
134.34
133.33
132.18
130.05
129.08
128.97
128.72
128.66
128.62
128.34
128.23
114.02
105.63
77.48
77.16
76.84
52.76
Cl
CO2Me
5d
f1 (ppm)

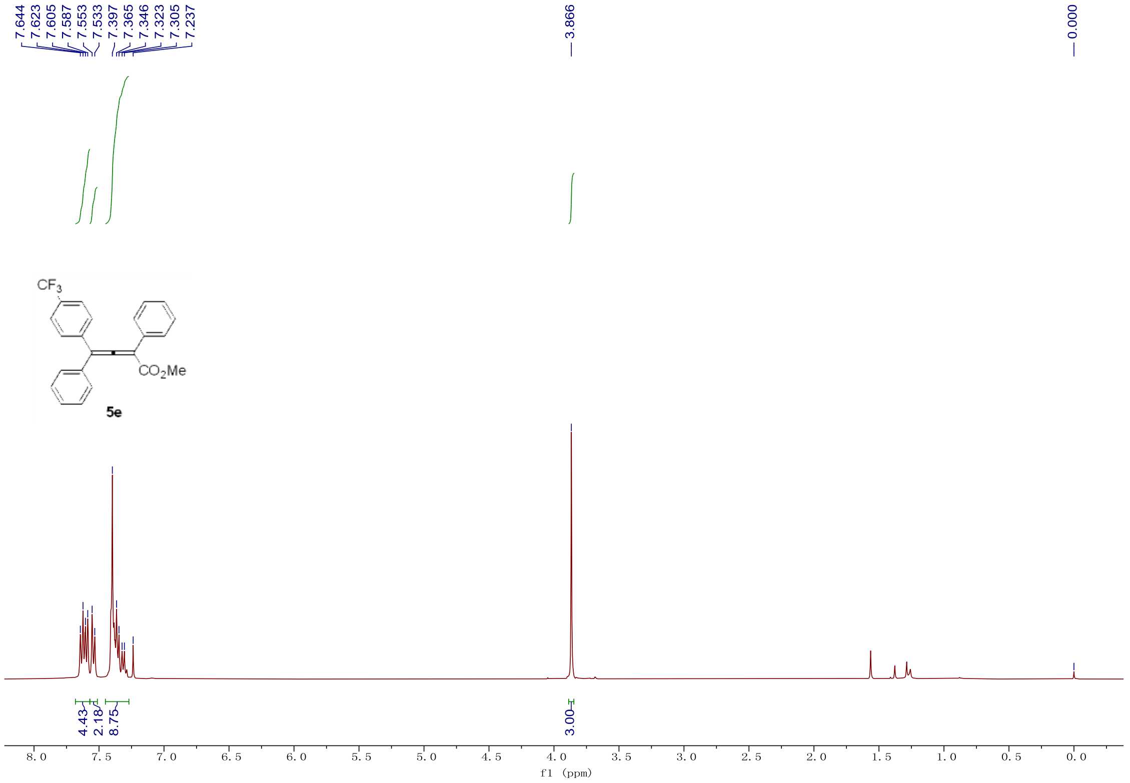
7.644
7.623
7.605
7.587
7.553
7.533
7.397
7.365
7.346
7.323
7.305
7.237
3.866
0.000
CF3
CO2Me
5e
4.43
2.18
8.75
3.00
8.0 7.5 7.0 6.5 6.0 5.5 5.0 4.5 4.0 3.5 3.0 2.5 2.0 1.5 1.0 0.5 0.0
f1 (ppm)

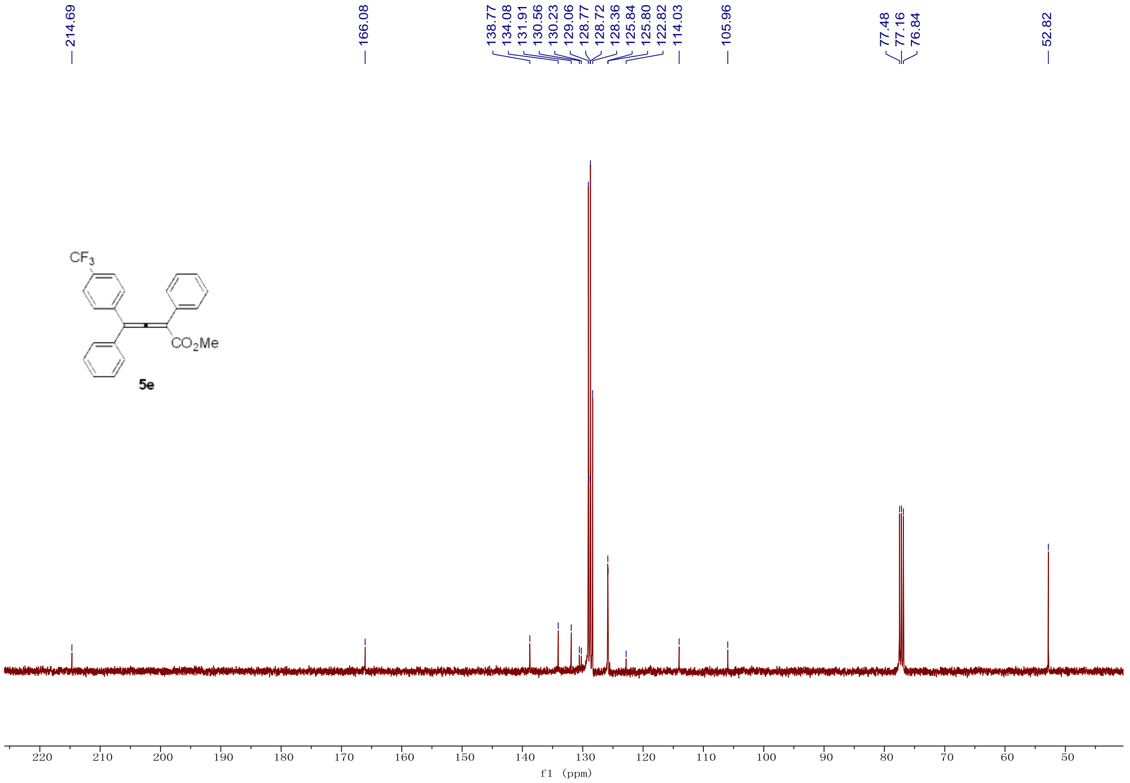
214.69
166.08
138.77
134.08
131.91
130.56
130.23
129.06
128.77
128.72
128.36
125.84
125.80
122.82
114.03
105.96
77.48
77.16
76.84
52.82
CF3
CO2Me
5e
220 210 200 190 180 170 160 150 140 130 120 110 100 90 80 70 60 50
f1 (ppm)

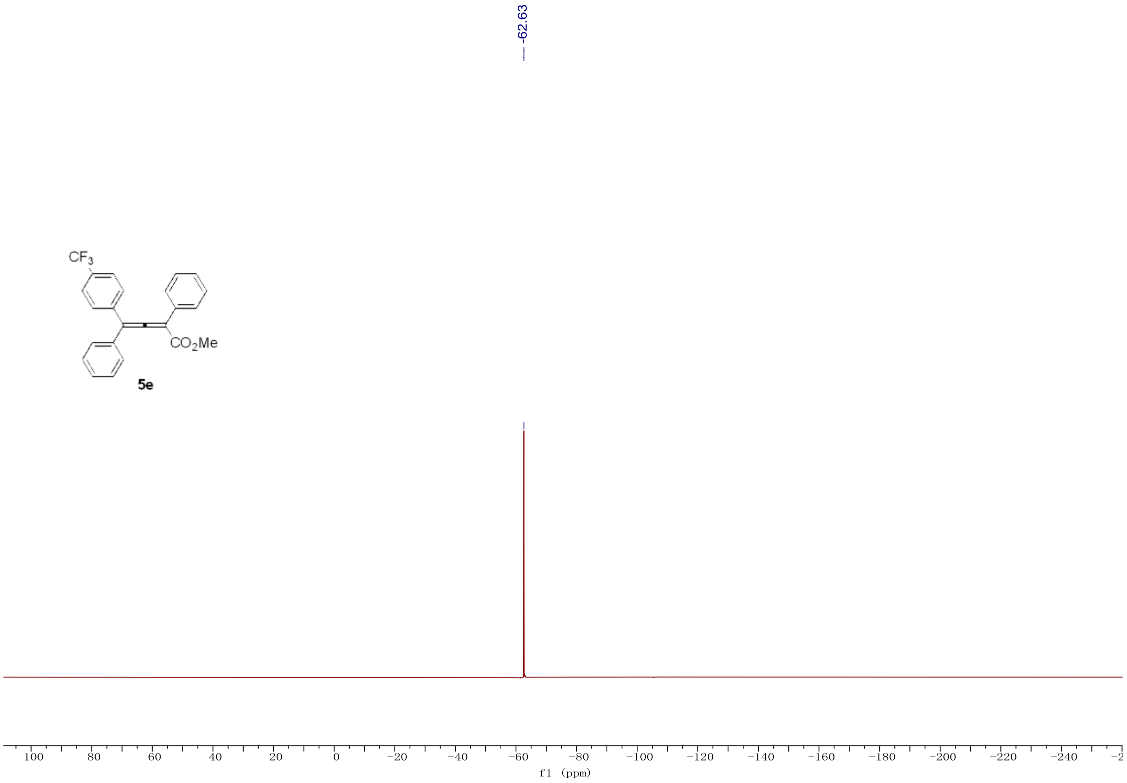
-62.63
CF3
CO2Me
5e
100 80 60 40 20 0 −20 −40 −60 −80 −100 −120 −140 −160 −180 −200 −220 −240
f1 (ppm)

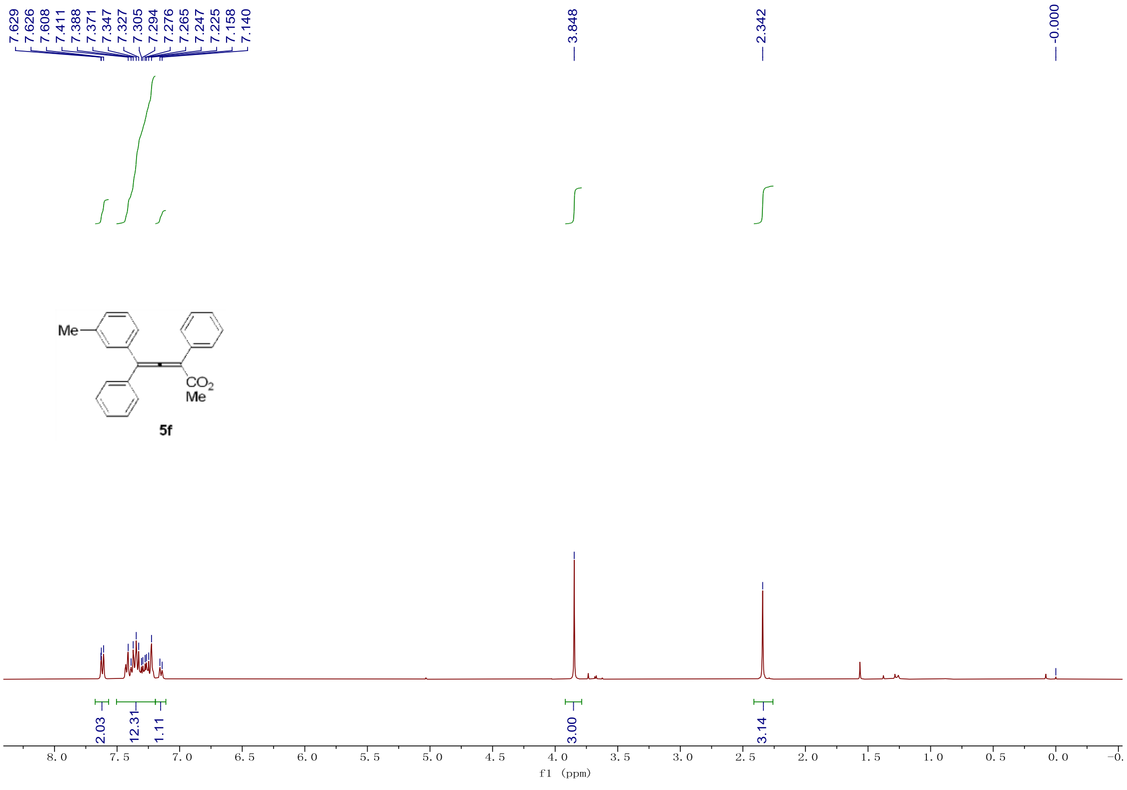
7.629 7.626 7.608 7.411 7.388 7.371 7.347 7.327 7.305 7.294 7.276 7.265 7.247 7.225 7.158 7.140
3.848
2.342
-0.000
Me
CO2
Me
5f
2.03 12.31 1.11
3.00
3.14
8.0 7.5 7.0 6.5 6.0 5.5 5.0 4.5 4.0 3.5 3.0 2.5 2.0 1.5 1.0 0.5 0.0
f1 (ppm)

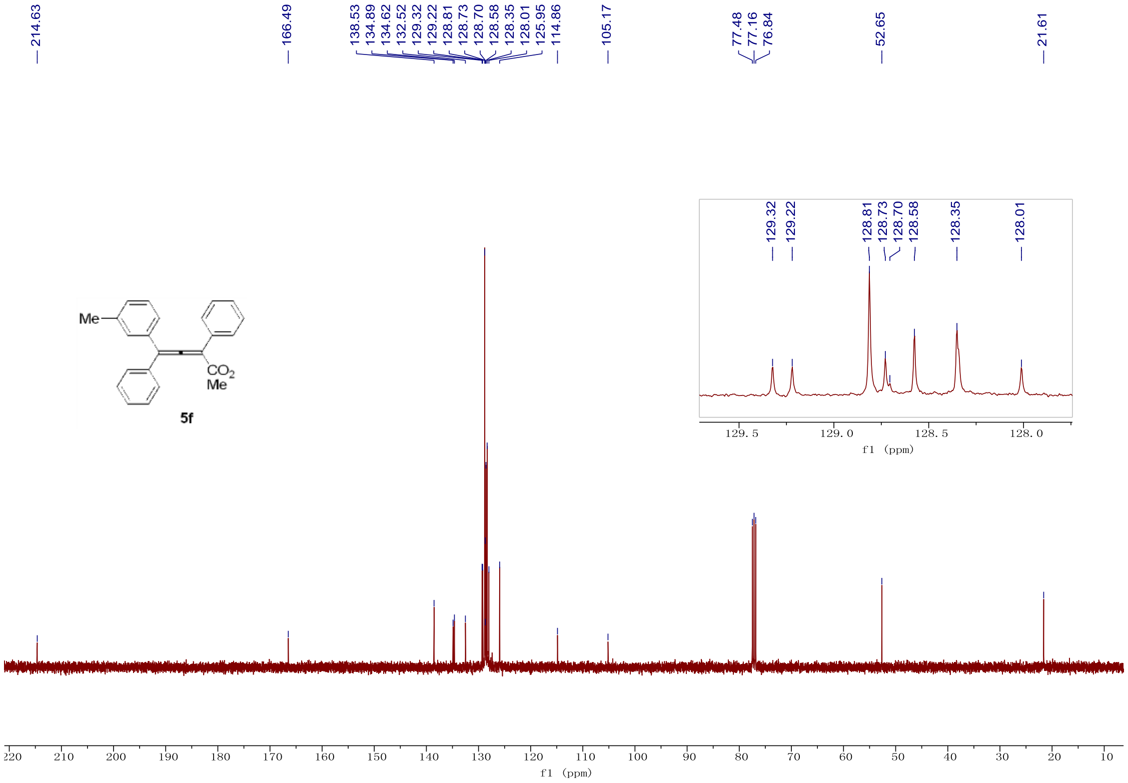
214.63
166.49
138.53
134.89
134.62
132.52
129.32
129.22
128.81
128.73
128.70
128.58
128.35
128.01
125.95
114.86
105.17
77.48
77.16
76.84
52.65
21.61
Me
$CO_2$
Me
5f
f1 (ppm)

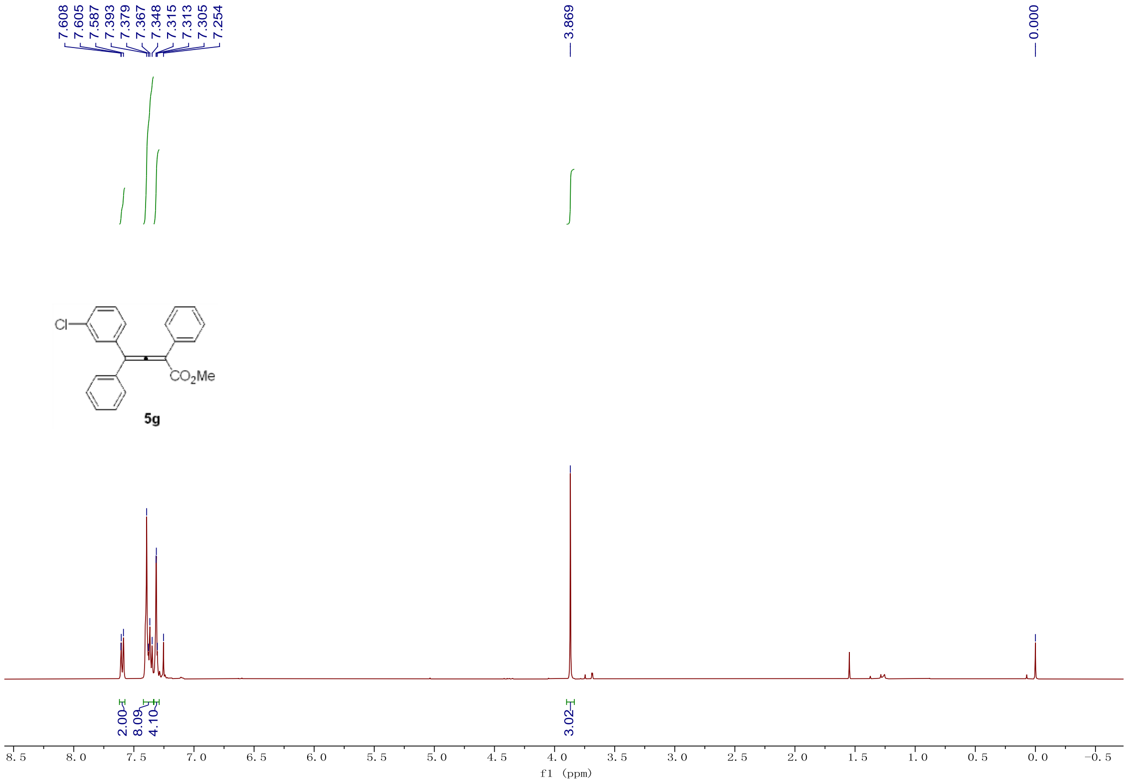
7.608
7.605
7.587
7.393
7.379
7.367
7.348
7.315
7.313
7.305
7.254
3.869
0.000
Cl
$CO_2Me$
5g
2.00
8.09
4.10
3.02
f1 (ppm)

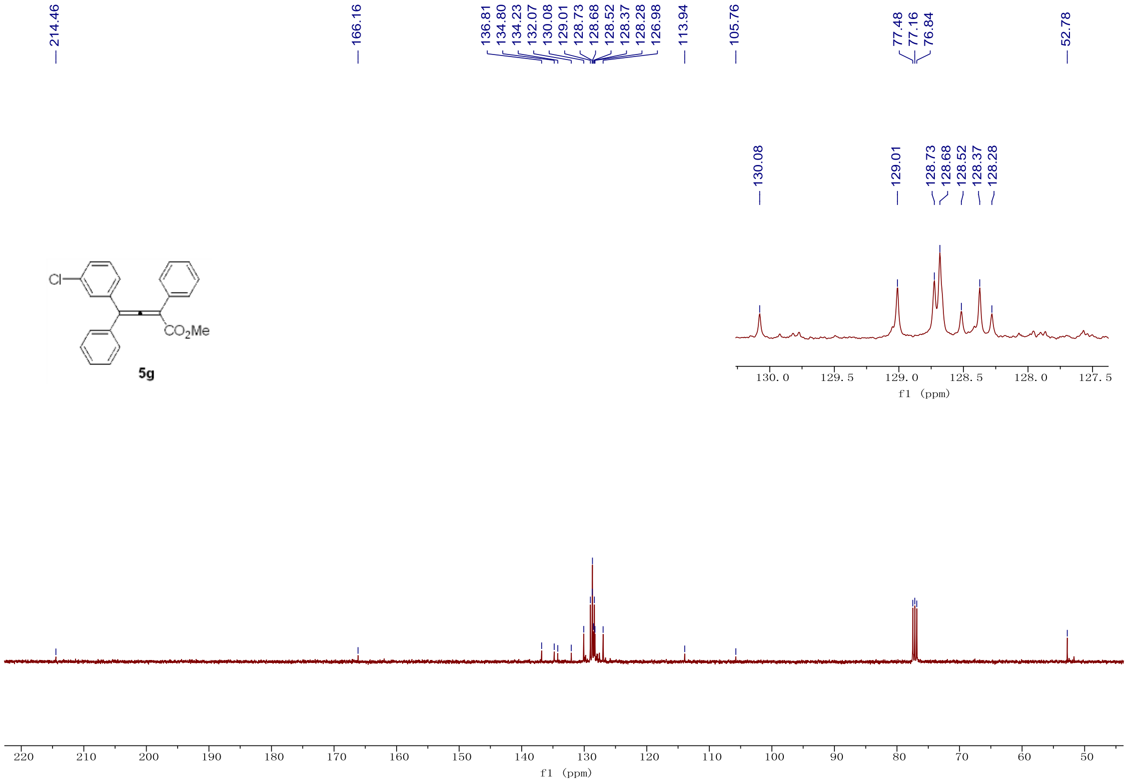
214.46
166.16
136.81
134.80
134.23
132.07
130.08
129.01
128.73
128.68
128.52
128.37
128.28
126.98
113.94
105.76
77.48
77.16
76.84
52.78
Cl
CO2Me
5g
130.08
129.01
128.73
128.68
128.52
128.37
128.28
f1 (ppm)
f1 (ppm)

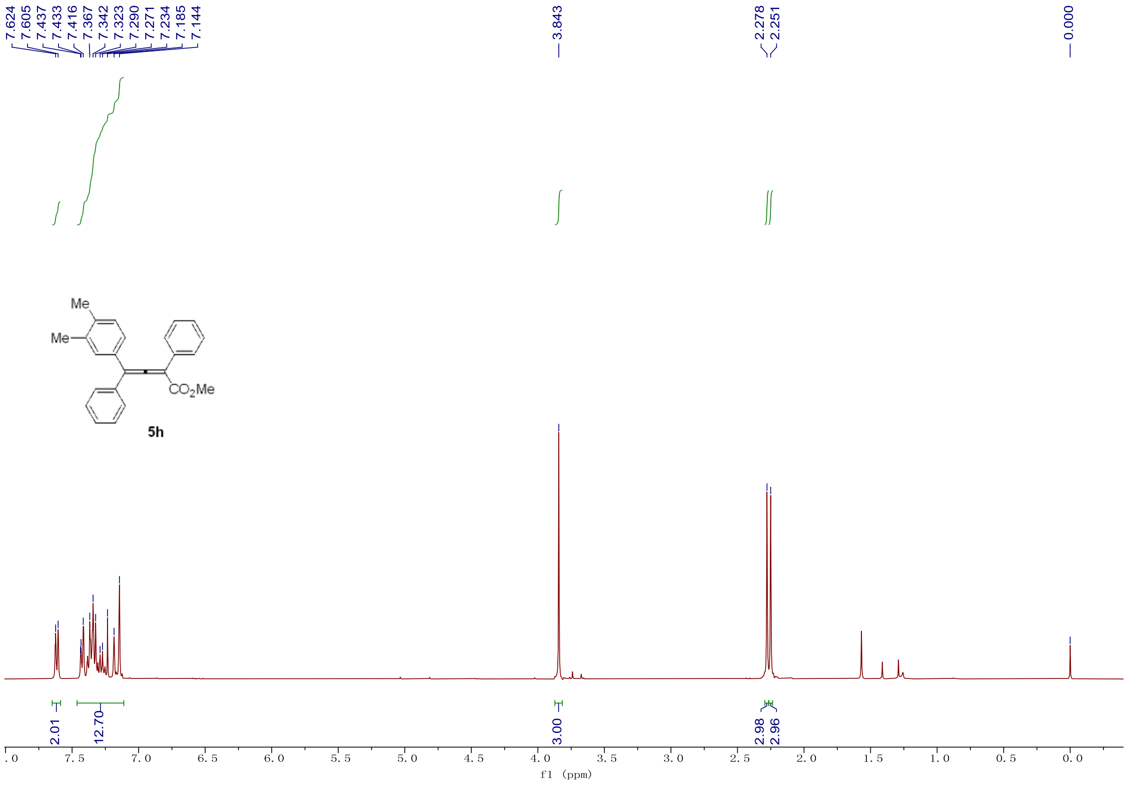
7.624
7.605
7.437
7.433
7.416
7.367
7.342
7.323
7.290
7.271
7.234
7.185
7.144
3.843
2.278
2.251
0.000
Me
Me
CO2Me
5h
2.01
12.70
3.00
2.98
2.96
f1 (ppm)

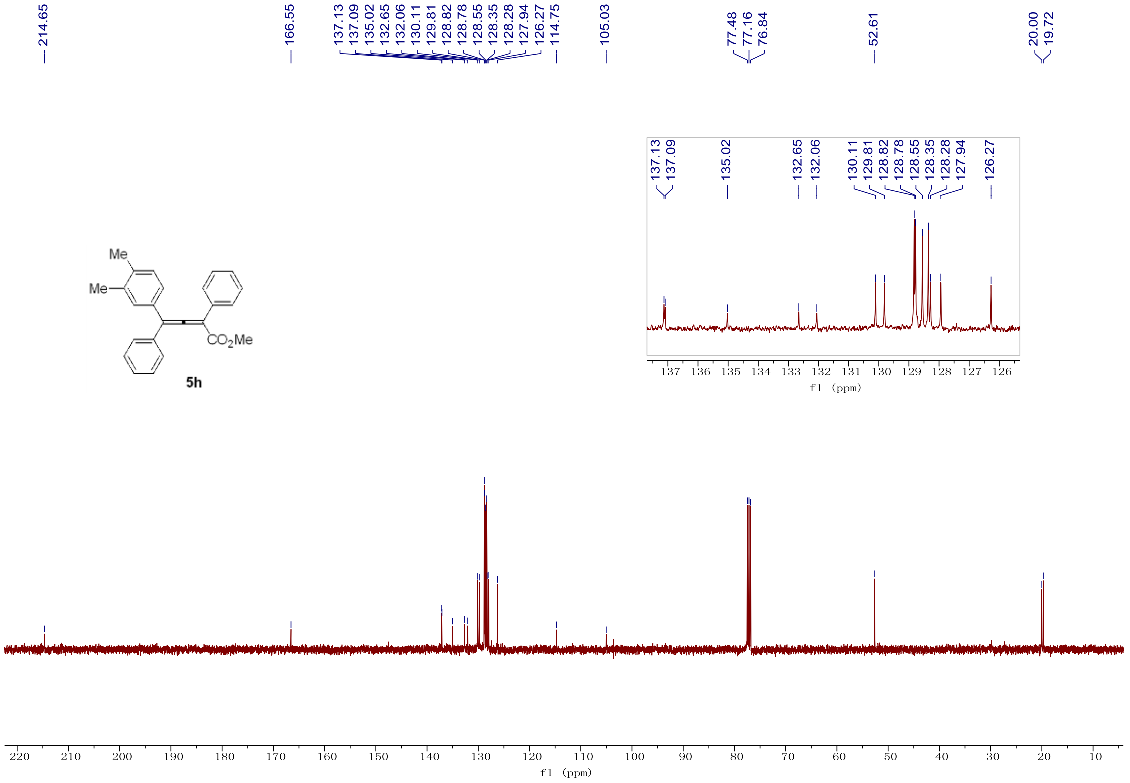
214.65
166.55
137.13
137.09
135.02
132.65
132.06
130.11
129.81
128.82
128.78
128.55
128.35
128.28
127.94
126.27
114.75
105.03
77.48
77.16
76.84
52.61
20.00
19.72
Me
Me
CO2Me
5h
f1 (ppm)

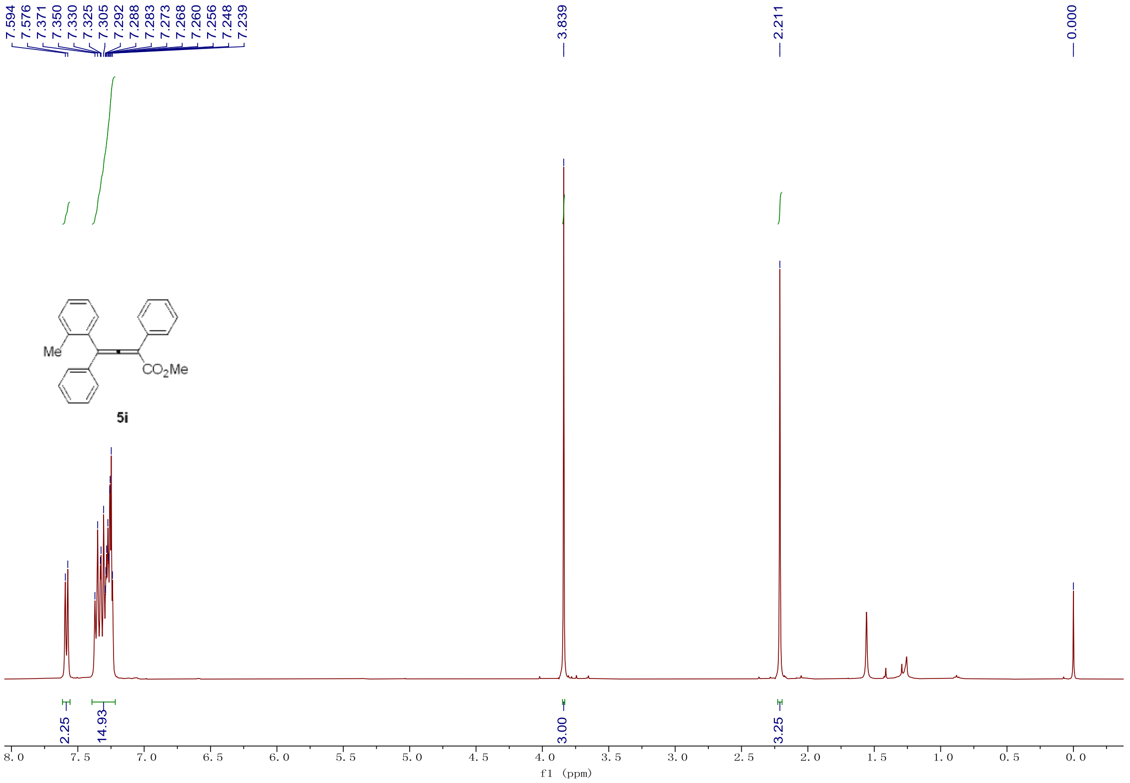
7.594
7.576
7.371
7.350
7.330
7.325
7.305
7.292
7.288
7.283
7.273
7.268
7.260
7.256
7.248
7.239
3.839
2.211
0.000
Me
CO2Me
5i
2.25
14.93
3.00
3.25
f1 (ppm)

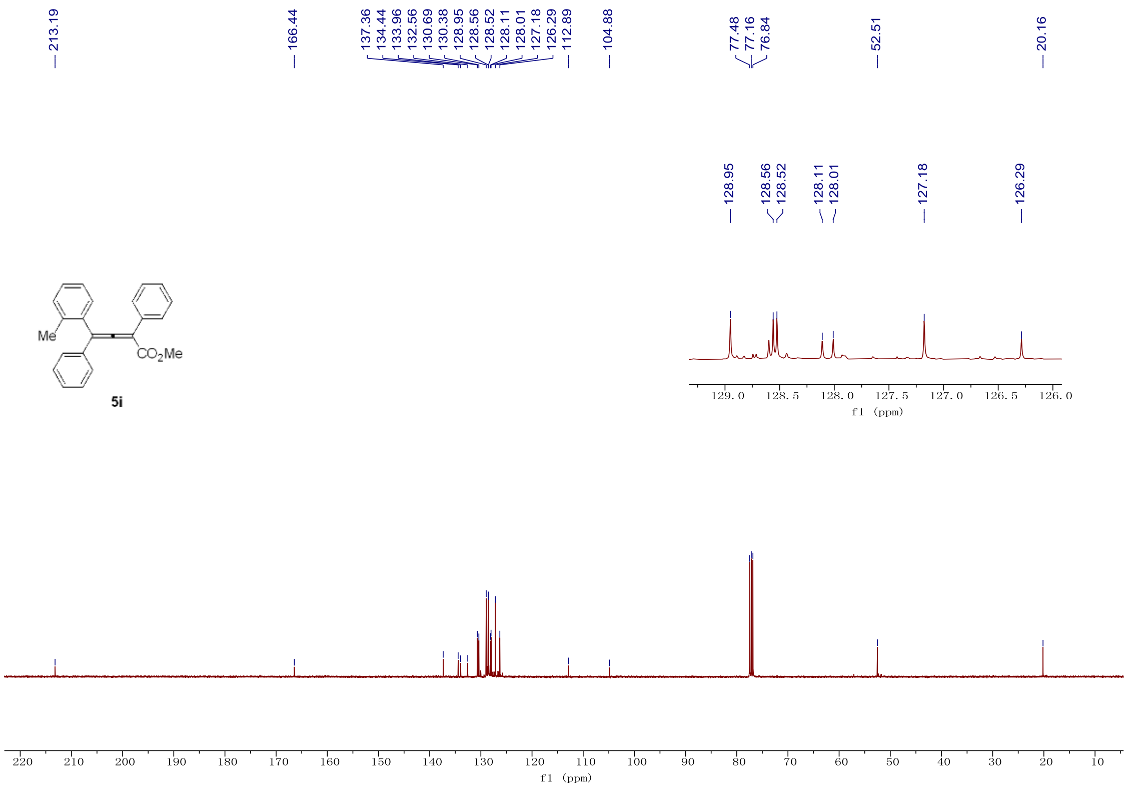
213.19
166.44
137.36 134.44 133.96 132.56 130.69 130.38 128.95 128.56 128.52 128.11 128.01 127.18 126.29 112.89
104.88
77.48 77.16 76.84
52.51
20.16
128.95 128.56 128.52 128.11 128.01 127.18 126.29
Me
CO2Me
5i
f1 (ppm)

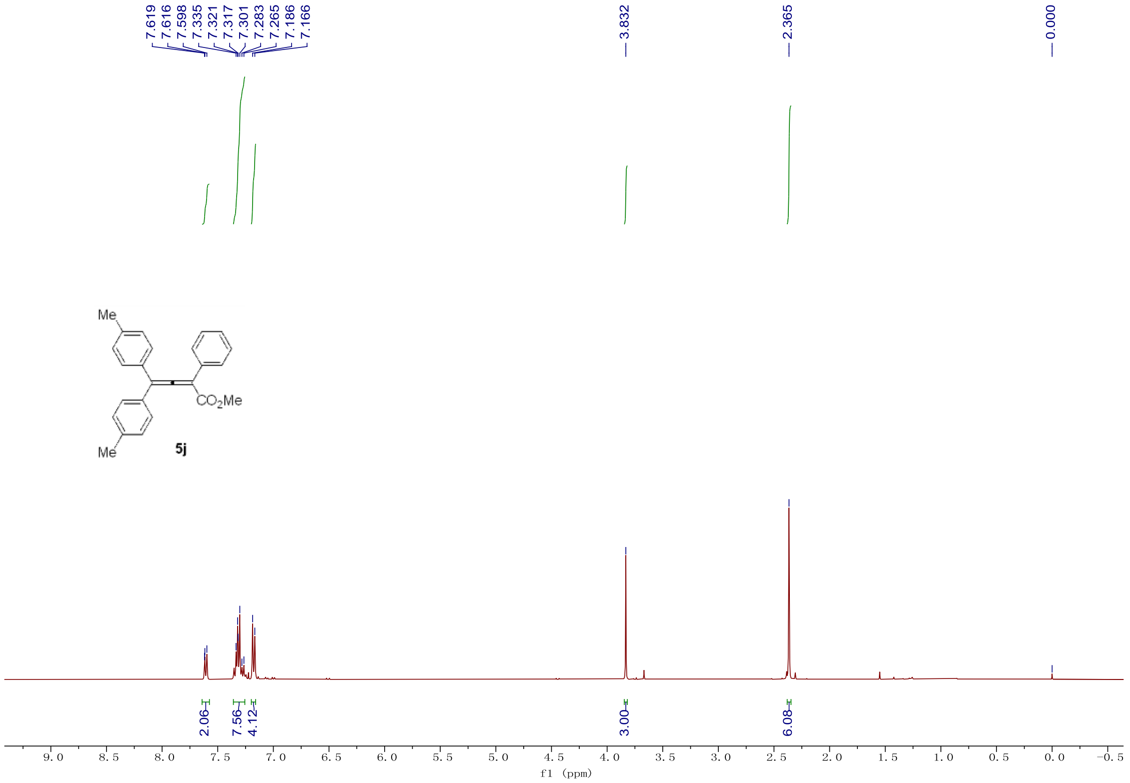
7.619 7.616 7.598 7.335 7.321 7.317 7.301 7.283 7.265 7.186 7.166
3.832
2.365
0.000
Me
CO2Me
Me
5j
2.06
7.56
4.12
3.00
6.08
f1 (ppm)

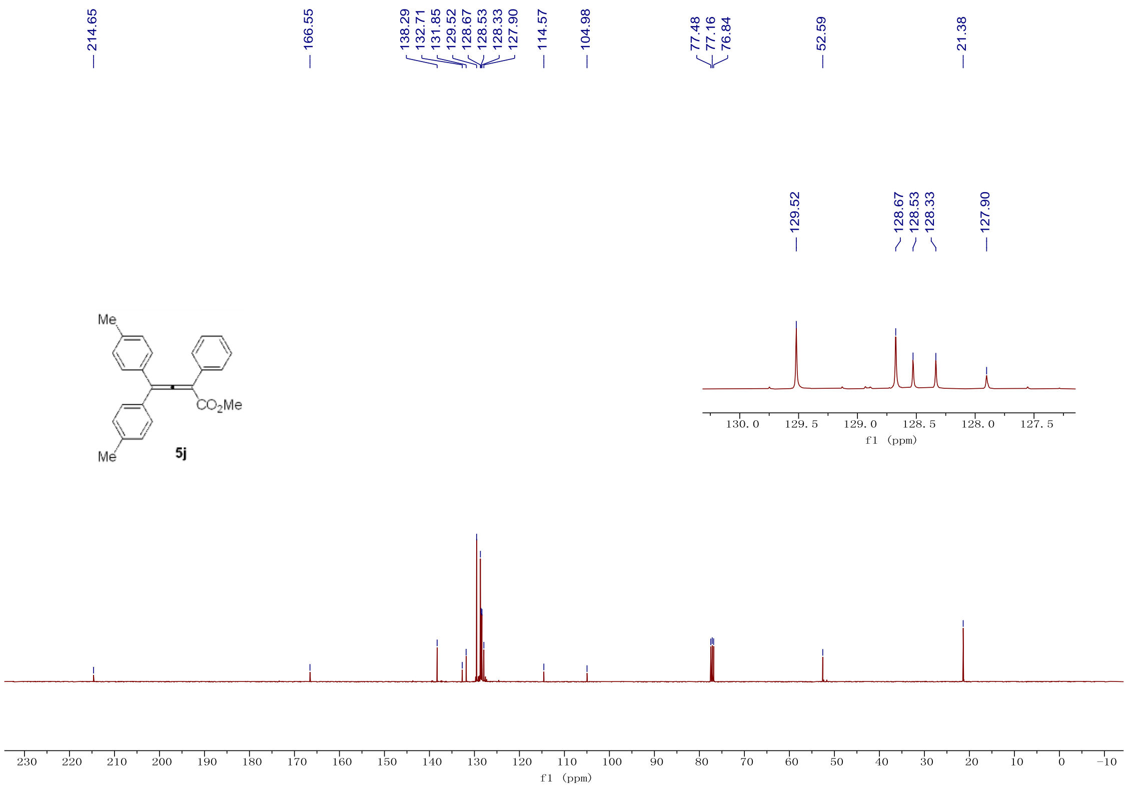
214.65
166.55
138.29
132.71
131.85
129.52
128.67
128.53
128.33
127.90
114.57
104.98
77.48
77.16
76.84
52.59
21.38
Me
Me
CO2Me
5j
129.52
128.67
128.53
128.33
127.90
130.0 129.5 129.0 128.5 128.0 127.5
f1 (ppm)
230 220 210 200 190 180 170 160 150 140 130 120 110 100 90 80 70 60 50 40 30 20 10 0 -10
f1 (ppm)

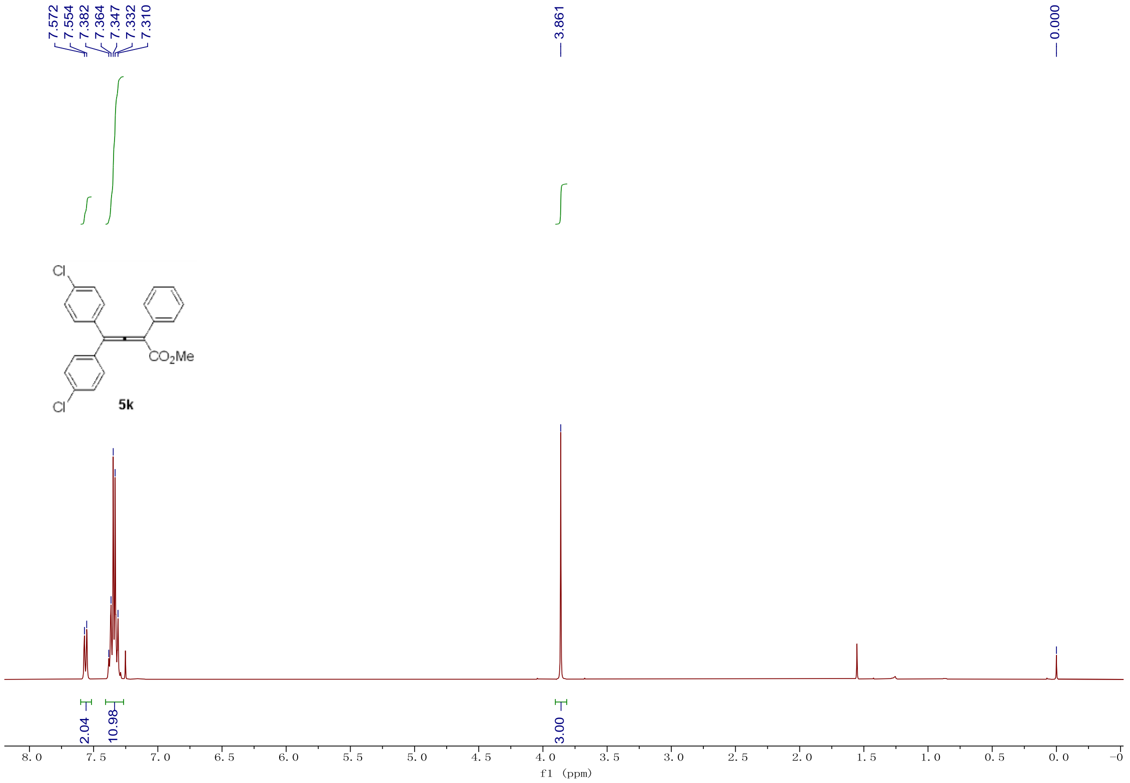
7.572
7.554
7.382
7.364
7.347
7.332
7.310
3.861
0.000
Cl
Cl
CO2Me
5k
2.04
10.98
3.00
8.0 7.5 7.0 6.5 6.0 5.5 5.0 4.5 4.0 3.5 3.0 2.5 2.0 1.5 1.0 0.5 0.0
f1 (ppm)

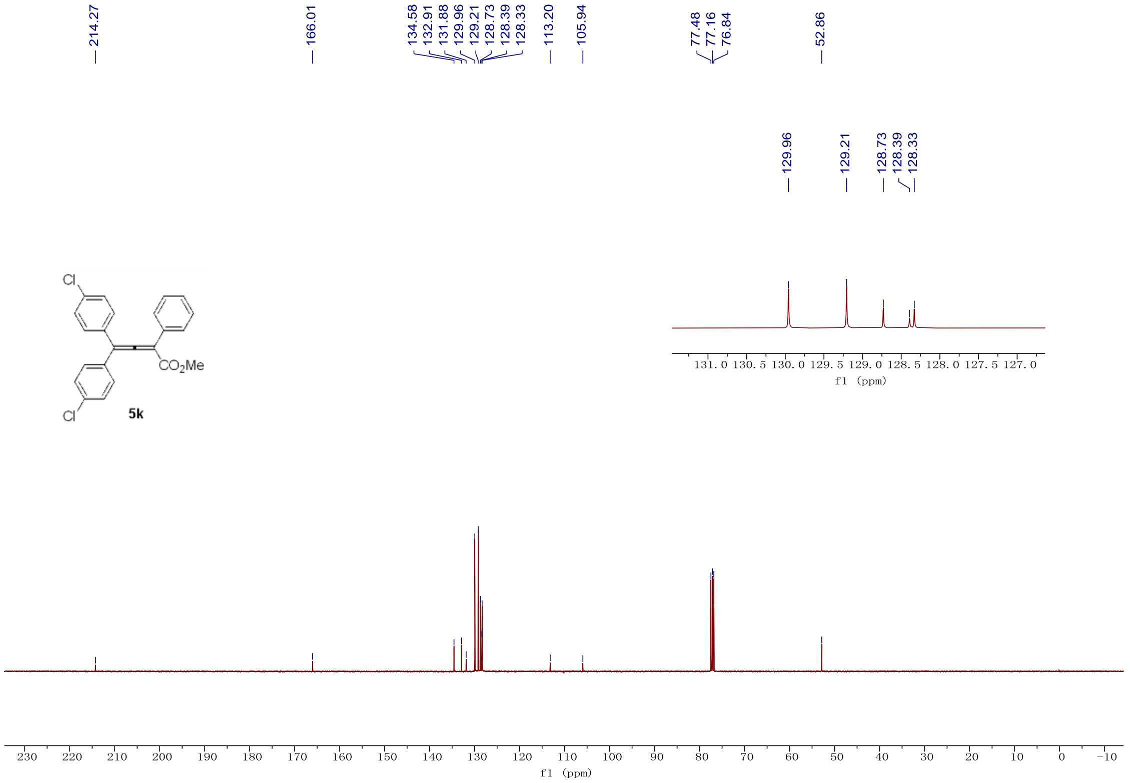
214.27
166.01
134.58
132.91
131.88
129.96
129.21
128.73
128.39
128.33
113.20
105.94
77.48
77.16
76.84
52.86
129.96
129.21
128.73
128.39
128.33
f1 (ppm)
f1 (ppm)
Cl
Cl
CO2Me

**5k**

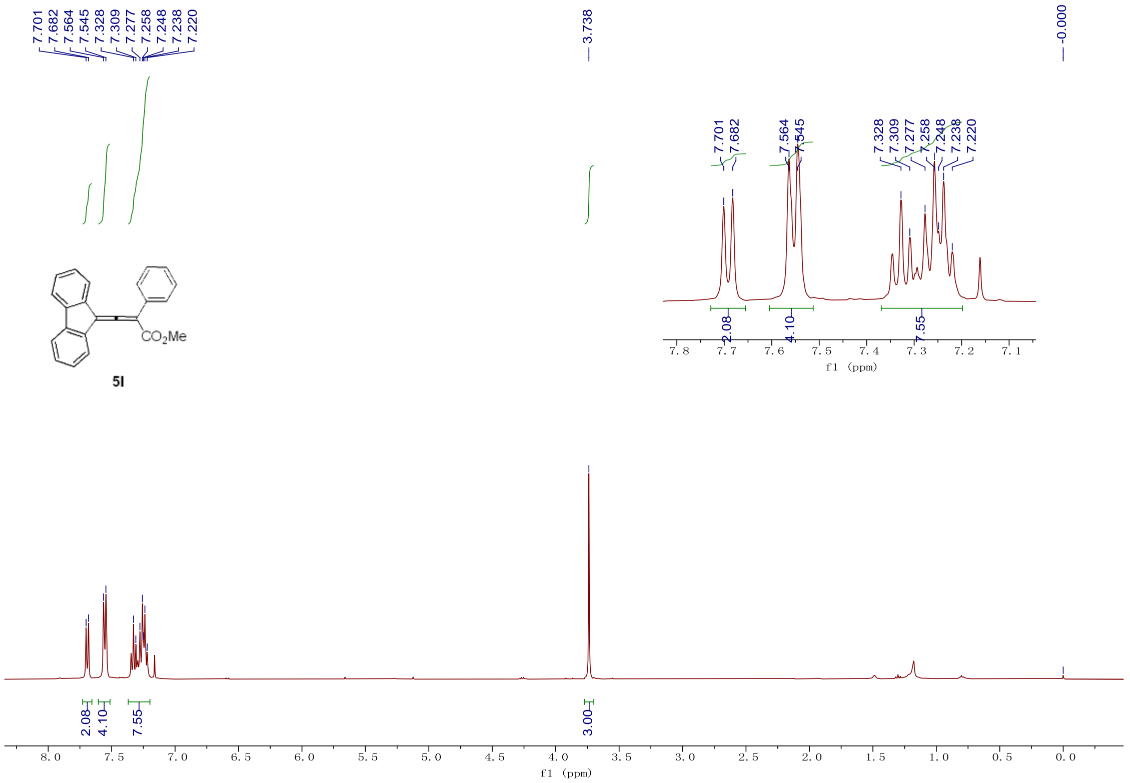
7.701
7.682
7.564
7.545
7.328
7.309
7.277
7.258
7.248
7.238
7.220
3.738
-0.000
2.08
4.10
7.55
3.00
f1 (ppm)
f1 (ppm)
CO2Me

**5l**

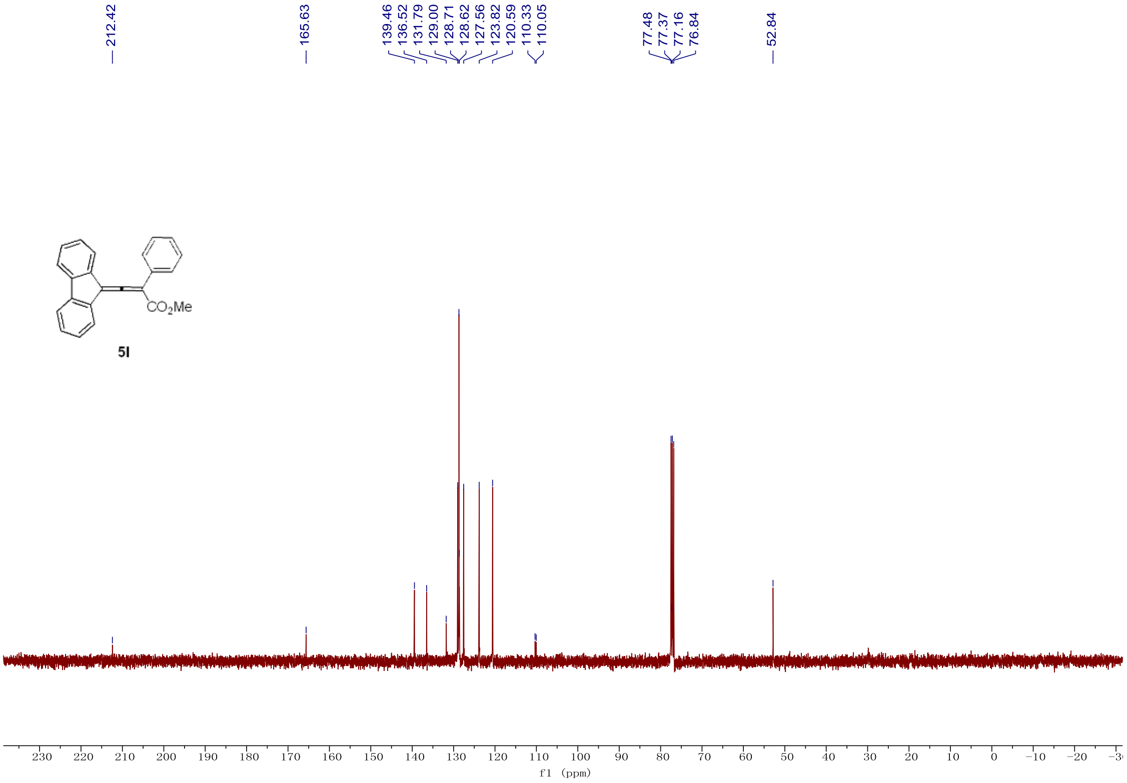
212.42
165.63
139.46
136.52
131.79
129.00
128.71
128.62
127.56
123.82
120.59
110.33
110.05
77.48
77.37
77.16
76.84
52.84
CO2Me
5l
f1 (ppm)

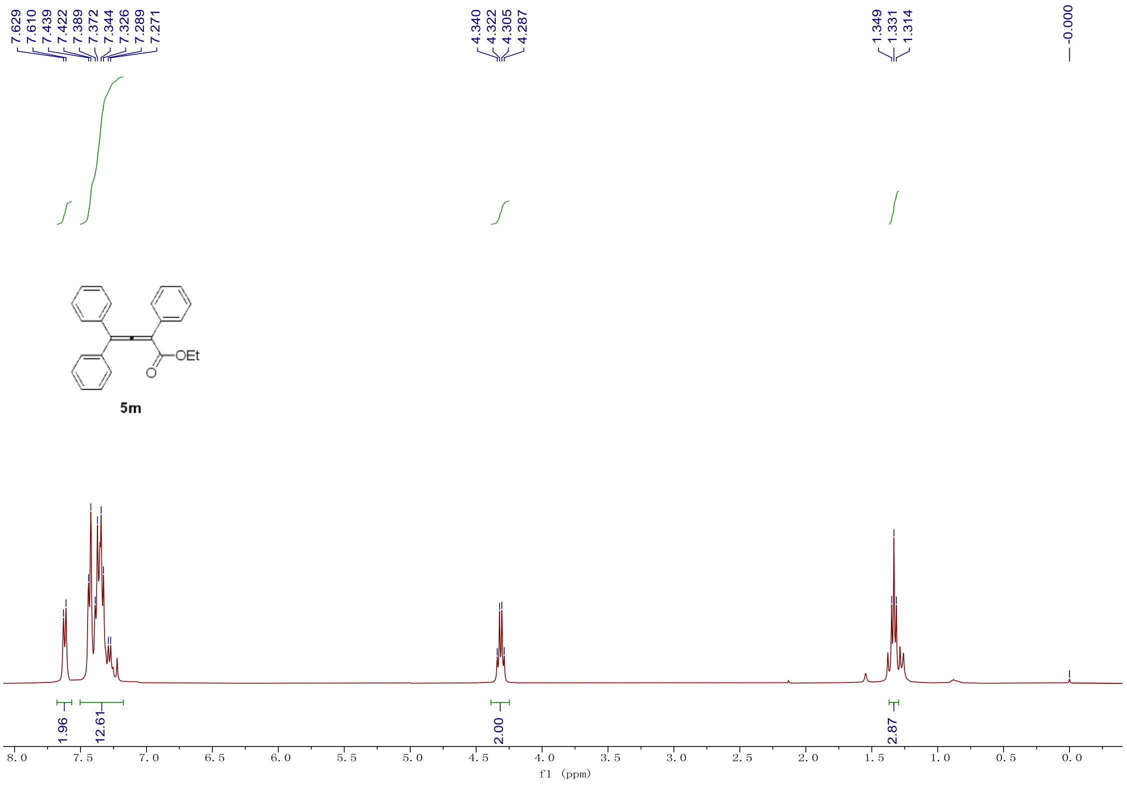
7.629
7.610
7.439
7.422
7.389
7.372
7.344
7.326
7.289
7.271
4.340
4.322
4.305
4.287
1.349
1.331
1.314
-0.000
OEt
O
5m
1.96
12.61
2.00
2.87
f1 (ppm)

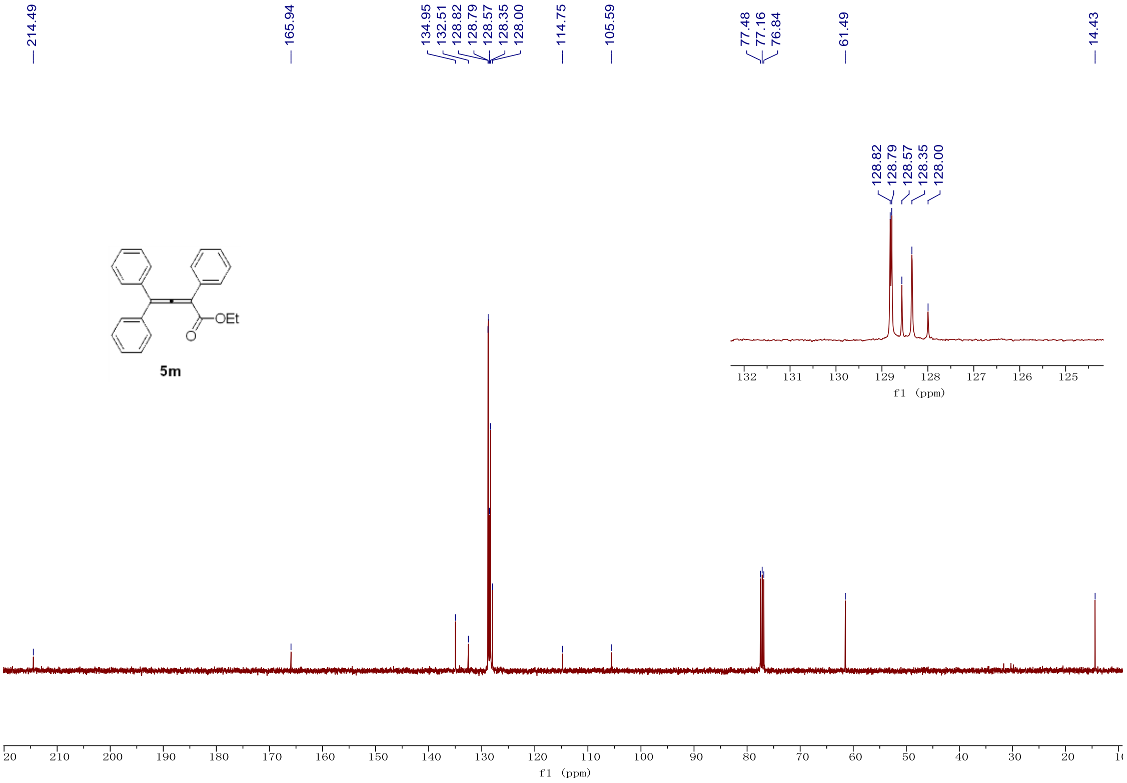
214.49
165.94
134.95
132.51
128.82
128.79
128.57
128.35
128.00
114.75
105.59
77.48
77.16
76.84
61.49
14.43
OEt
5m
f1 (ppm)

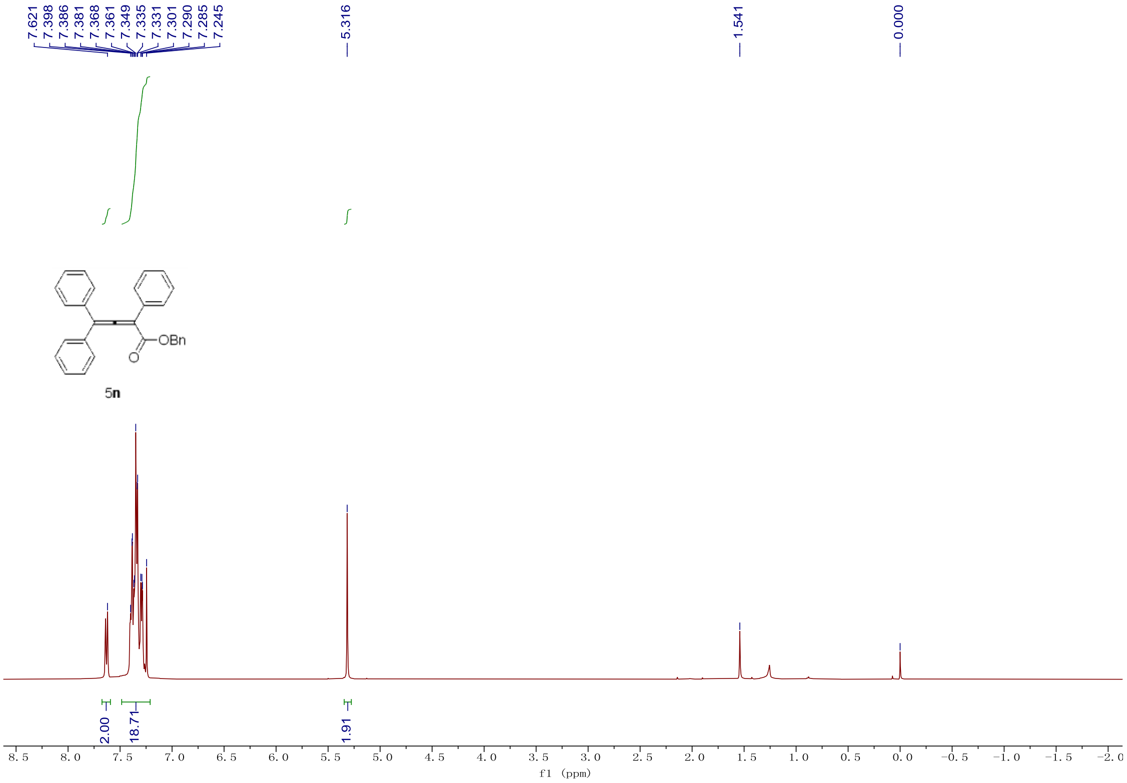
7.621
7.398
7.386
7.381
7.368
7.361
7.349
7.335
7.331
7.301
7.290
7.285
7.245
5.316
1.541
0.000
OBn
5n
2.00
18.71
1.91
f1 (ppm)

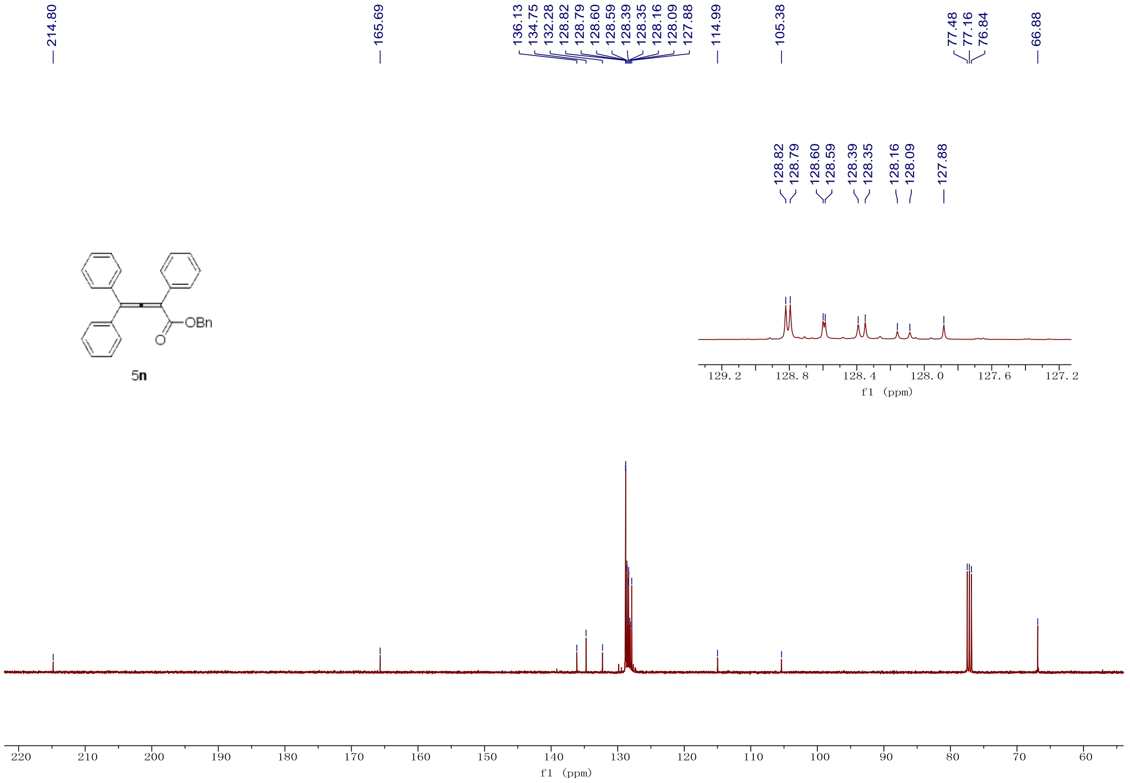
214.80
165.69
136.13
134.75
132.28
128.82
128.79
128.60
128.59
128.39
128.35
128.16
128.09
127.88
114.99
105.38
77.48
77.16
76.84
66.88
OBn
f1 (ppm)

5n

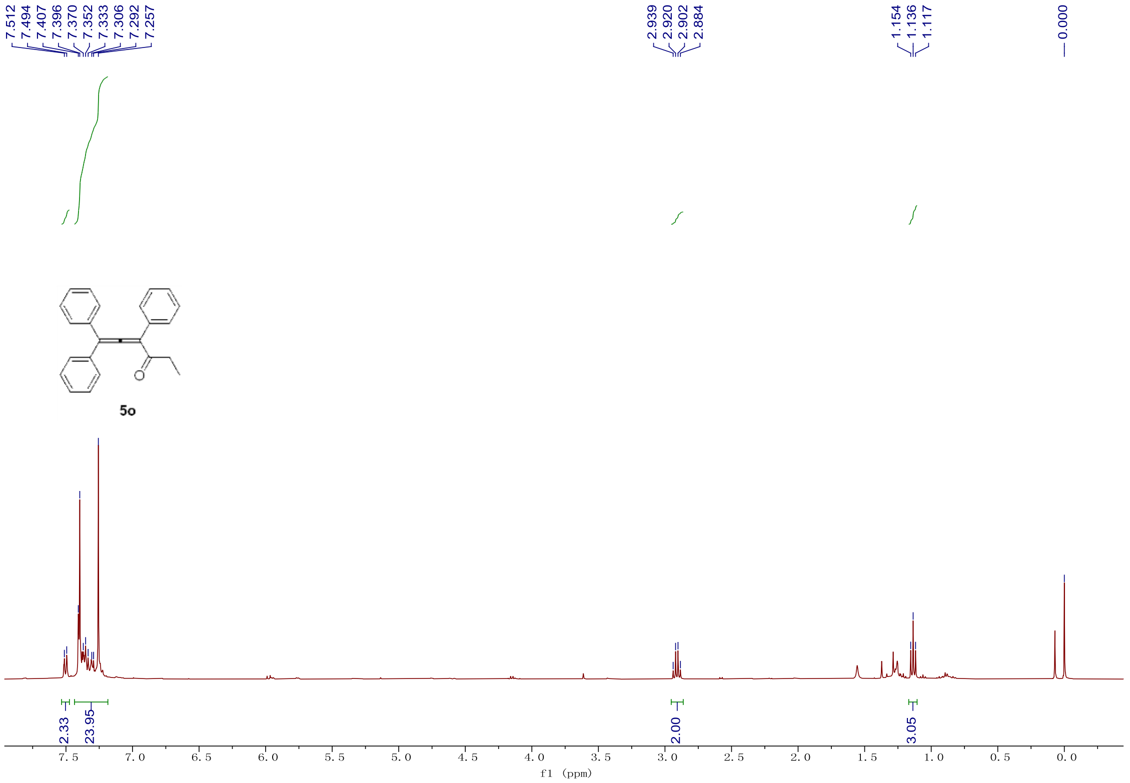
7.512
7.494
7.407
7.396
7.370
7.352
7.333
7.306
7.292
7.257
2.939
2.920
2.902
2.884
1.154
1.136
1.117
0.000
2.33
23.95
2.00
3.05
f1 (ppm)

5o

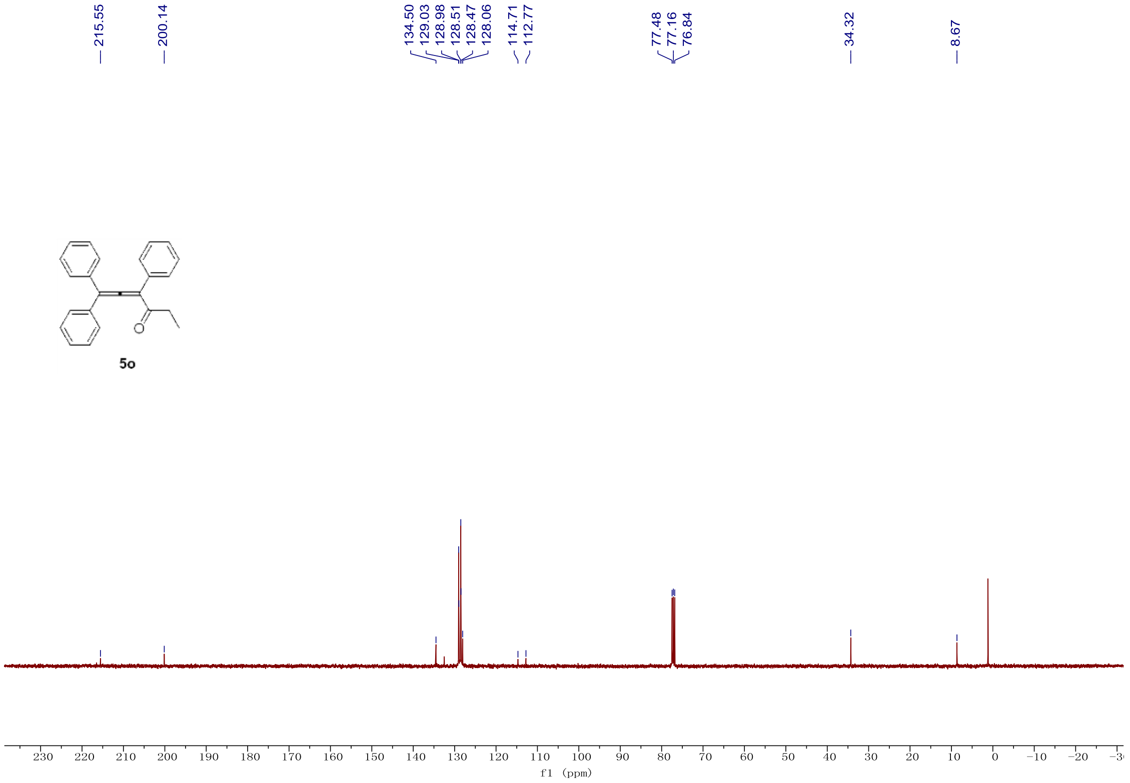
215.55
200.14
134.50
129.03
128.98
128.51
128.47
128.06
114.71
112.77
77.48
77.16
76.84
34.32
8.67
5o
230 220 210 200 190 180 170 160 150 140 130 120 110 100 90 80 70 60 50 40 30 20 10 0 −10 −20
f1 (ppm)

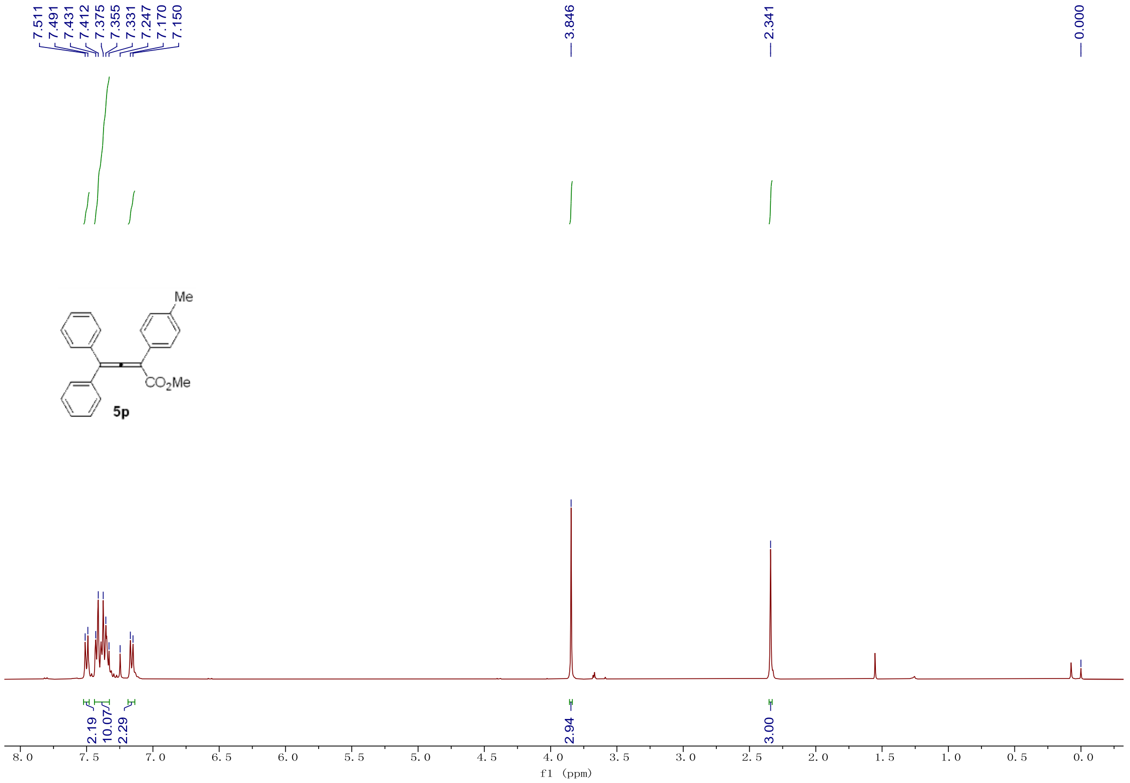
7.511
7.491
7.431
7.412
7.375
7.355
7.331
7.247
7.170
7.150
3.846
2.341
0.000
Me
$CO_2Me$
5p
2.19
10.07
2.29
2.94
3.00
8.0 7.5 7.0 6.5 6.0 5.5 5.0 4.5 4.0 3.5 3.0 2.5 2.0 1.5 1.0 0.5 0.0
f1 (ppm)

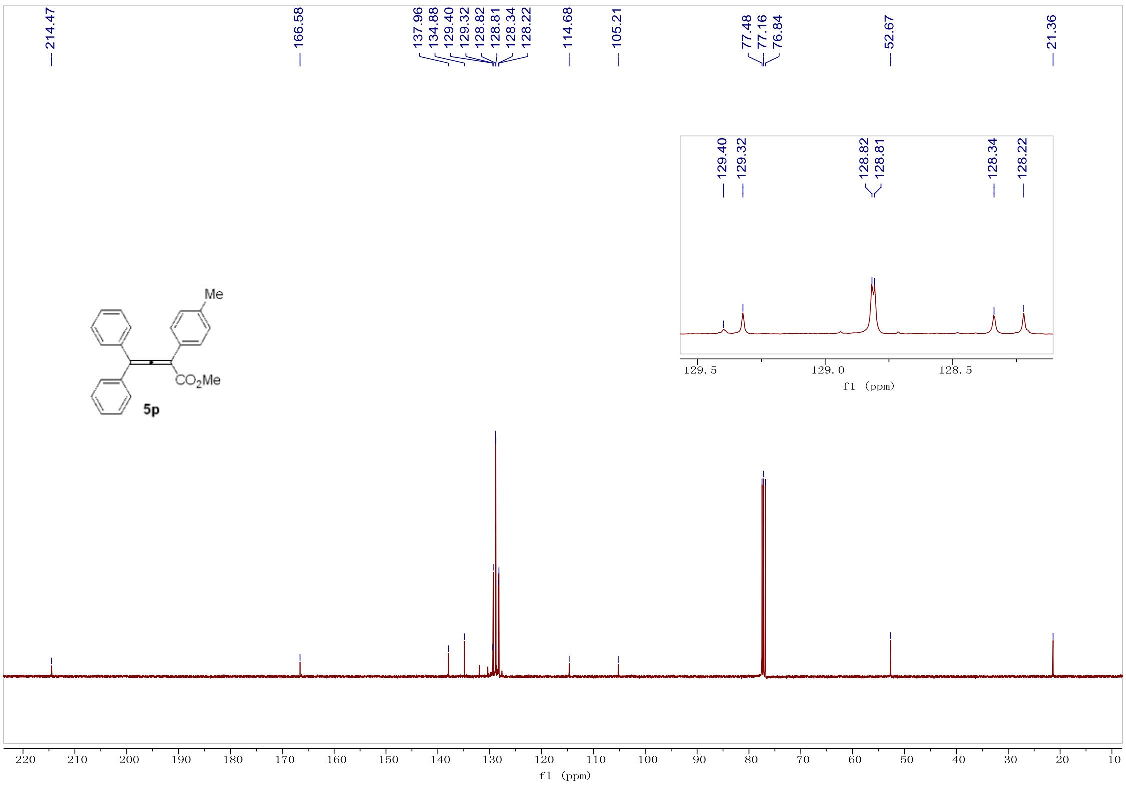

214.47
166.58
137.96
134.88
129.40
129.32
128.82
128.81
128.34
128.22
114.68
105.21
77.48
77.16
76.84
52.67
21.36
Me
CO2Me
5p
f1 (ppm)


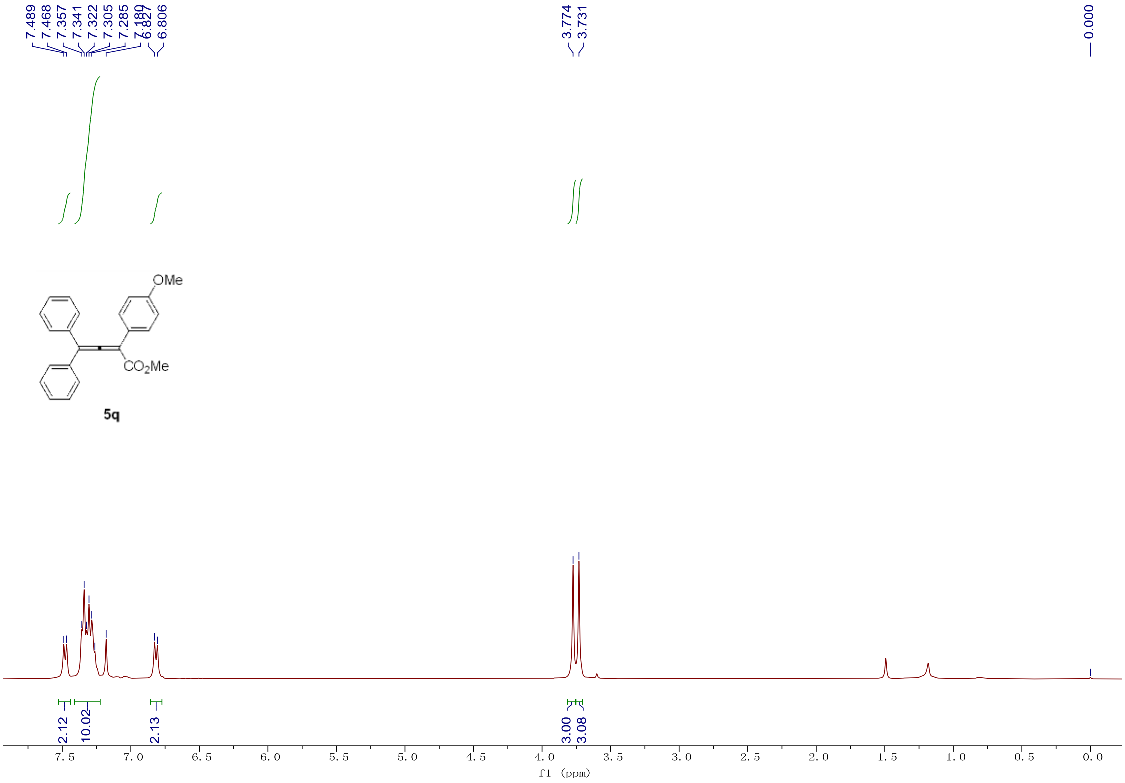

7.489
7.468
7.357
7.341
7.322
7.305
7.285
6.806
3.774
3.731
0.000
OMe
CO2Me
5q
2.12
10.02
2.13
3.00
3.08
f1 (ppm)

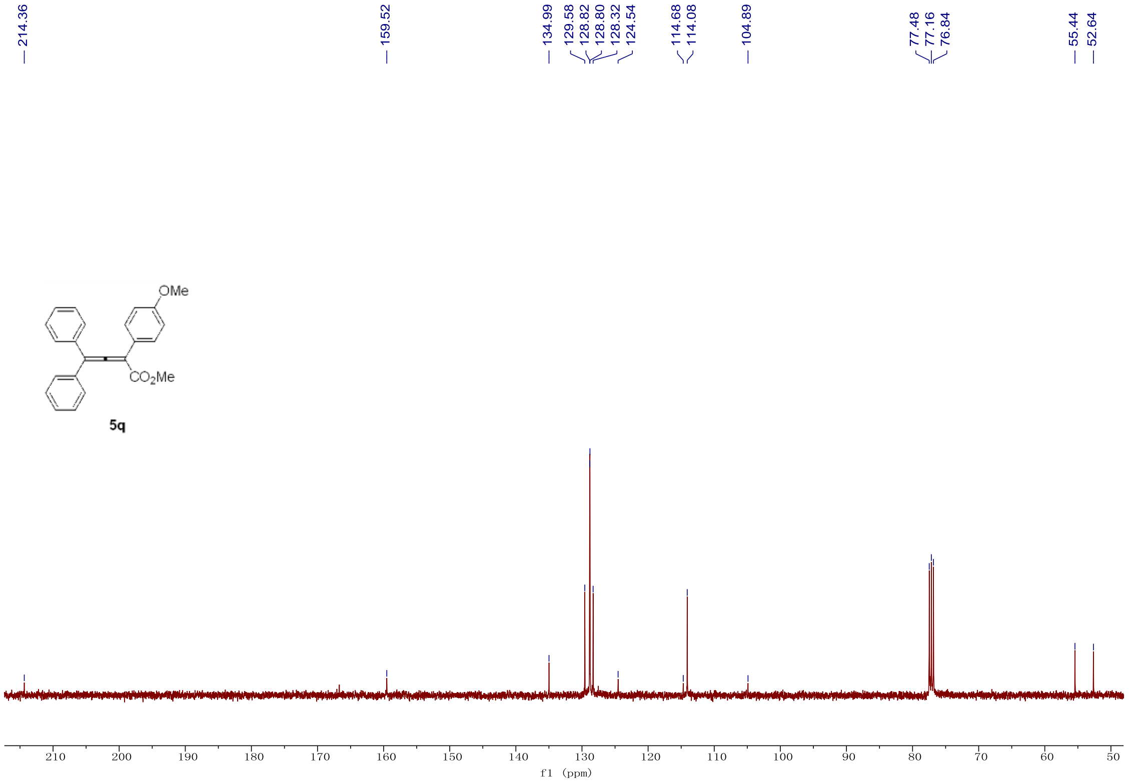
214.36
159.52
134.99
129.58
128.82
128.80
128.32
124.54
114.68
114.08
104.89
77.48
77.16
76.84
55.44
52.64
OMe
CO2Me
5q
210 200 190 180 170 160 150 140 130 120 110 100 90 80 70 60 50
f1 (ppm)

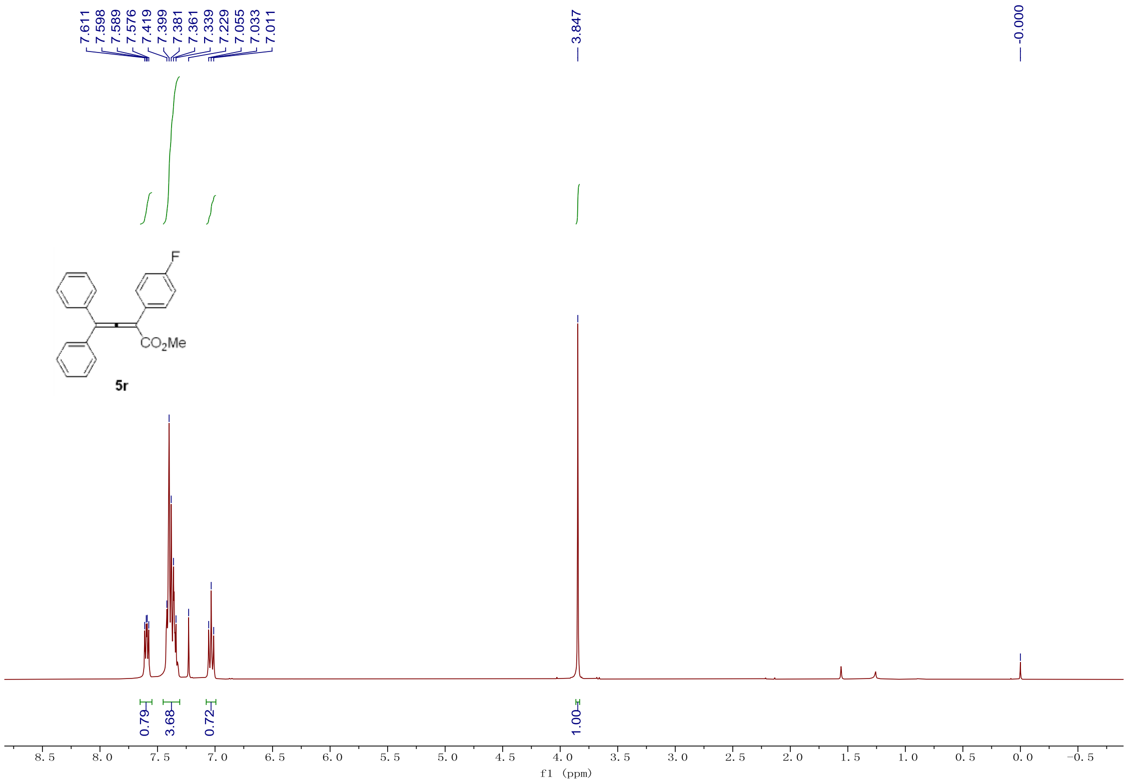
7.611
7.598
7.589
7.576
7.419
7.399
7.381
7.361
7.339
7.229
7.055
7.033
7.011
3.847
-0.000
F
CO2Me
5r
0.79
3.68
0.72
1.00
8.5 8.0 7.5 7.0 6.5 6.0 5.5 5.0 4.5 4.0 3.5 3.0 2.5 2.0 1.5 1.0 0.5 0.0 -0.5
f1 (ppm)

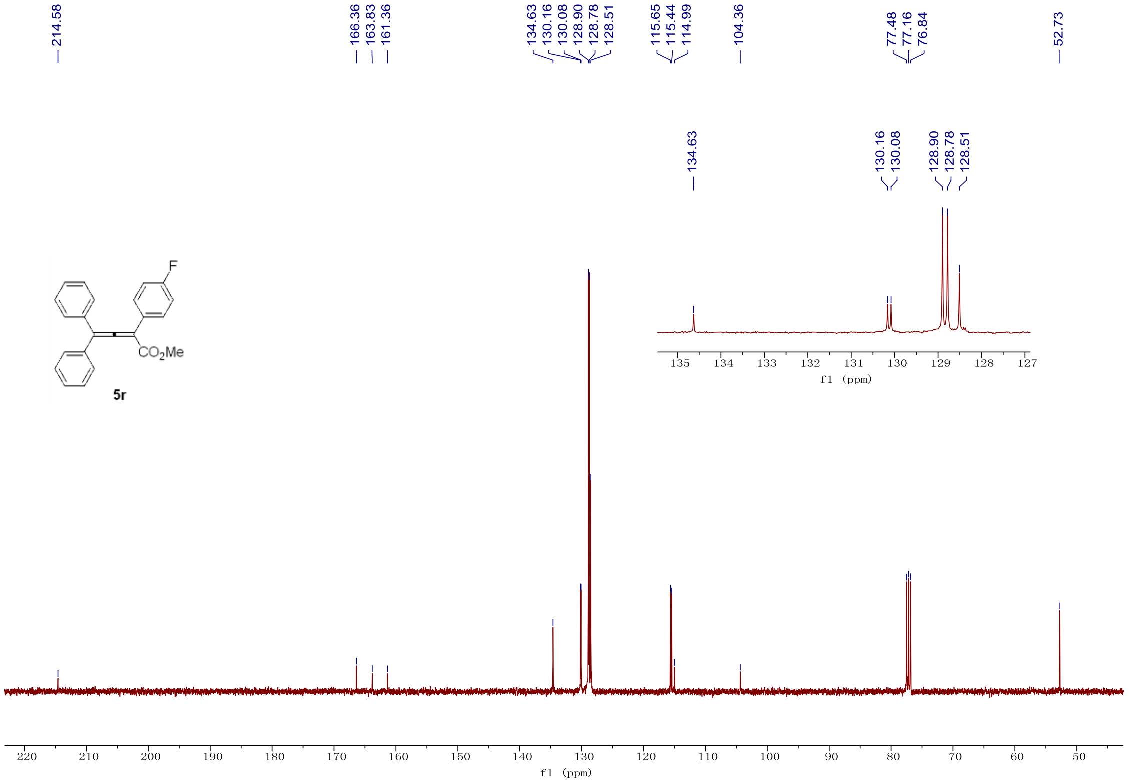
214.58
166.36
163.83
161.36
134.63
130.16
130.08
128.90
128.78
128.51
115.65
115.44
114.99
104.36
77.48
77.16
76.84
52.73
F
CO2Me
5r
f1 (ppm)

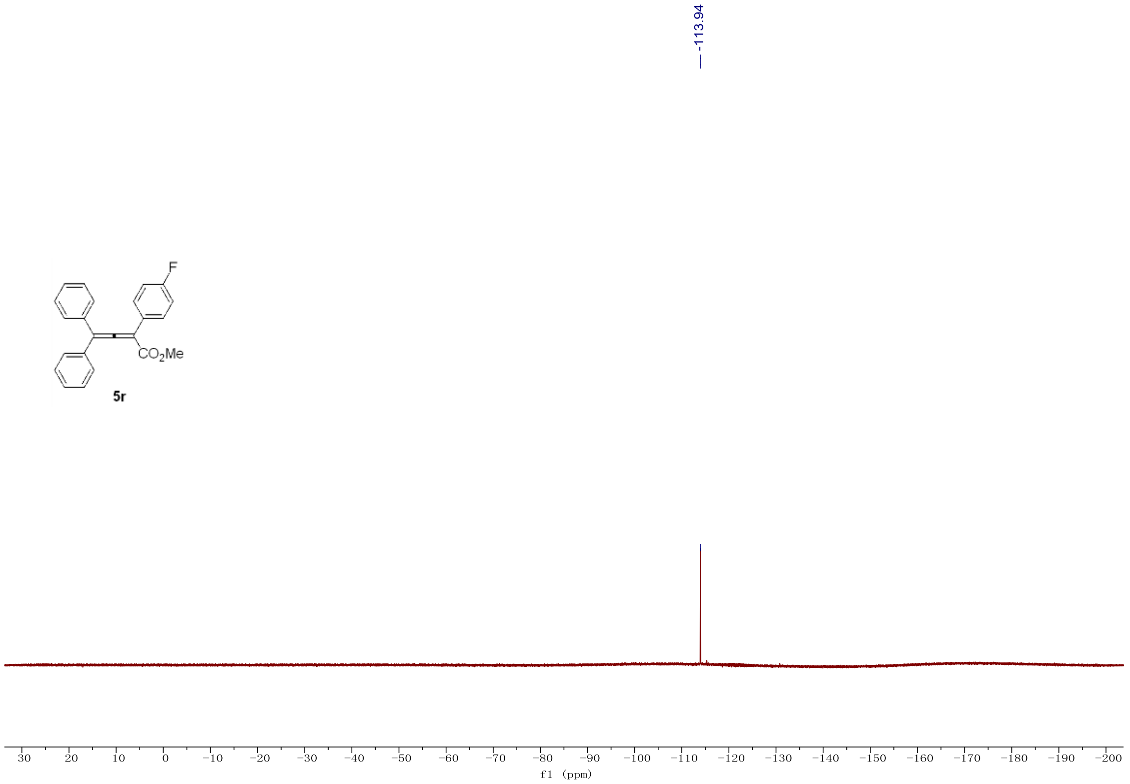
-113.94
F
CO2Me
5r
f1 (ppm)

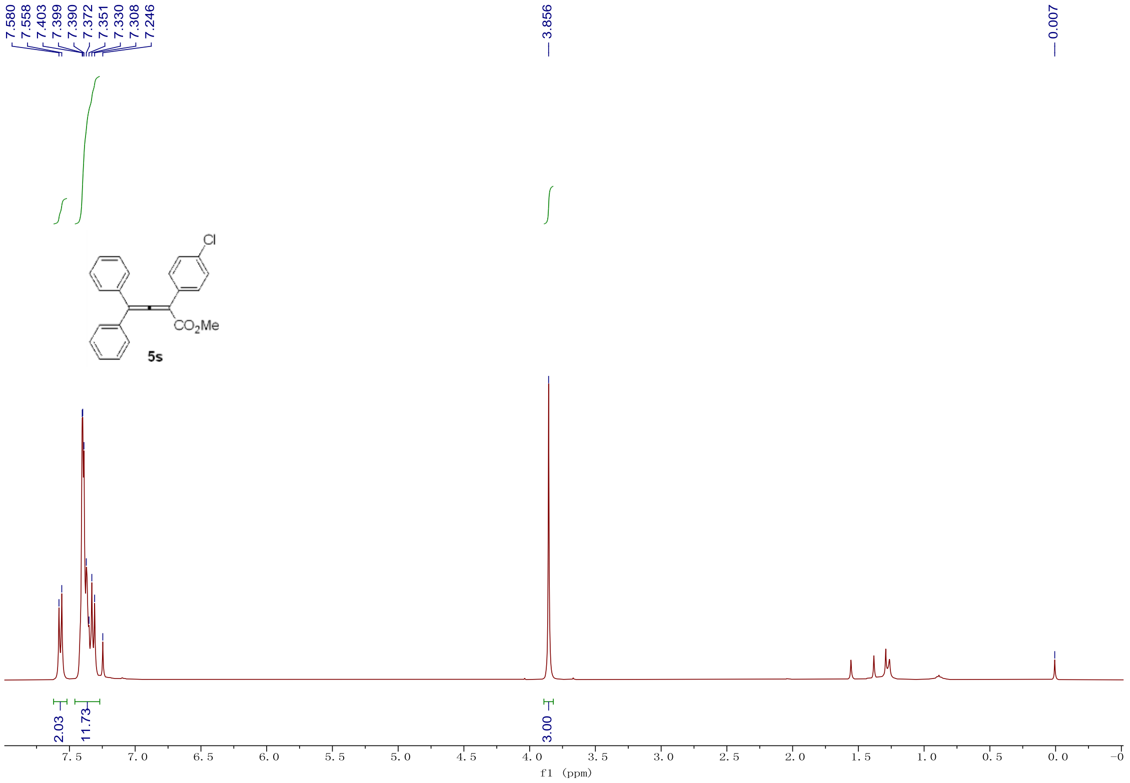
7.580
7.558
7.403
7.399
7.390
7.372
7.351
7.330
7.308
7.246
3.856
0.007
Cl
CO2Me
5s
2.03
11.73
3.00
f1 (ppm)

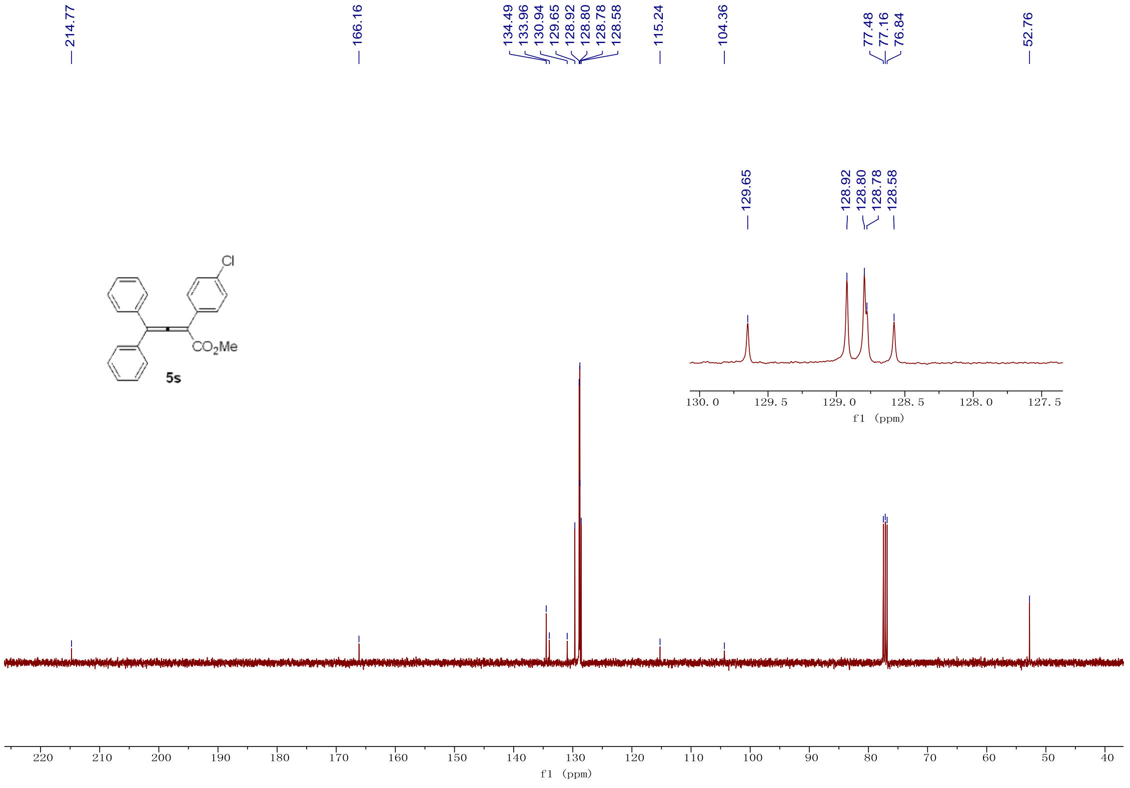
214.77
166.16
134.49
133.96
130.94
129.65
128.92
128.80
128.78
128.58
115.24
104.36
77.48
77.16
76.84
52.76
Cl
CO2Me
5s
f1 (ppm)

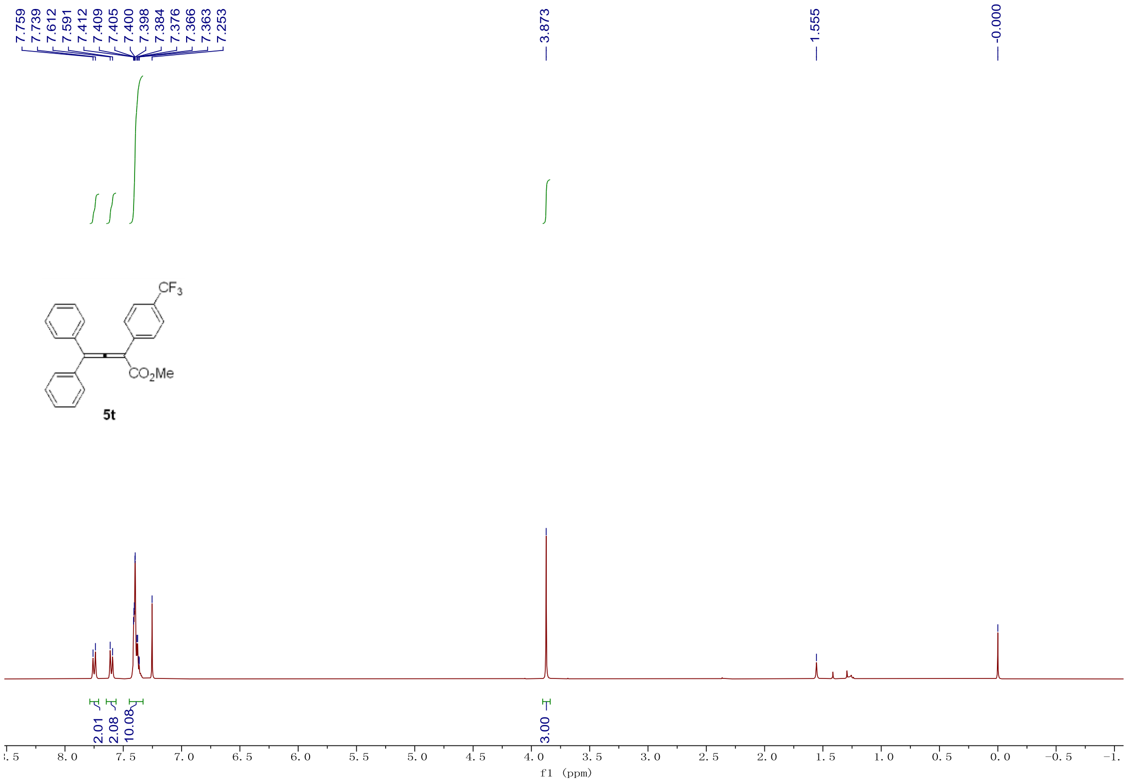
7.759
7.739
7.612
7.591
7.412
7.409
7.405
7.400
7.398
7.384
7.376
7.366
7.363
7.253
3.873
1.555
-0.000
CF3
CO2Me
5t
2.01
2.08
10.08
3.00
f1 (ppm)

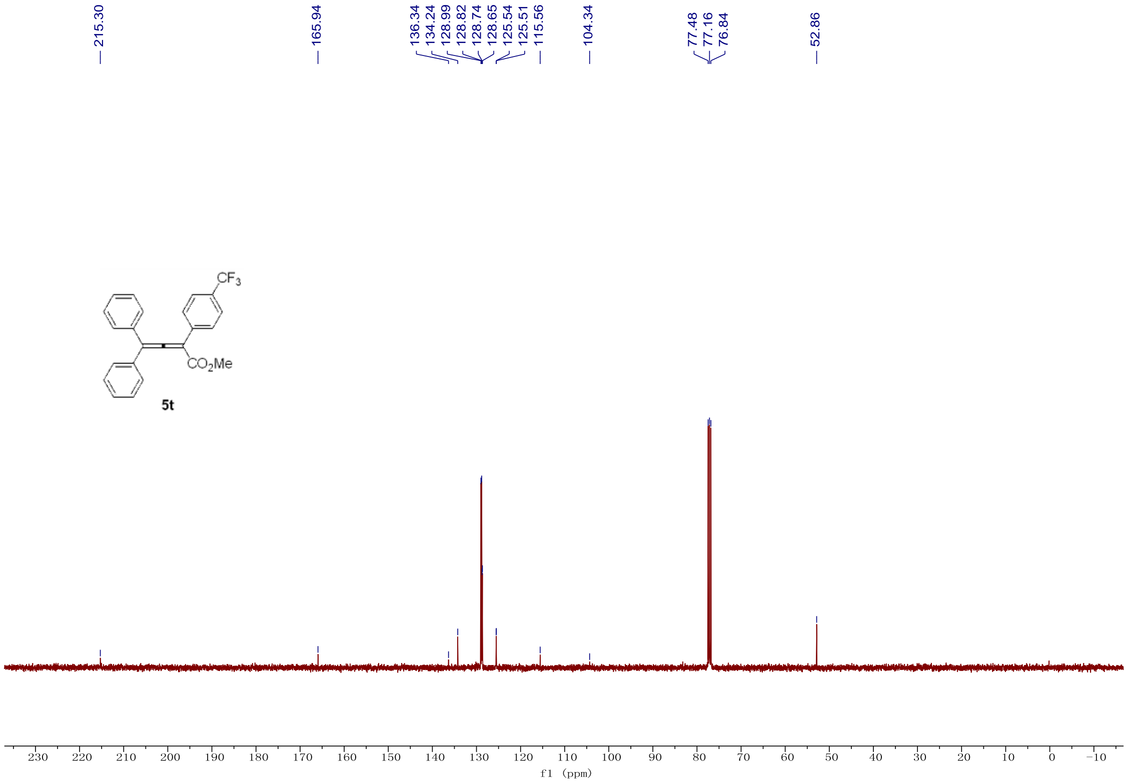
215.30
165.94
136.34
134.24
128.99
128.82
128.74
128.65
125.54
125.51
115.56
104.34
77.48
77.16
76.84
52.86
CF3
CO2Me
5t
f1 (ppm)

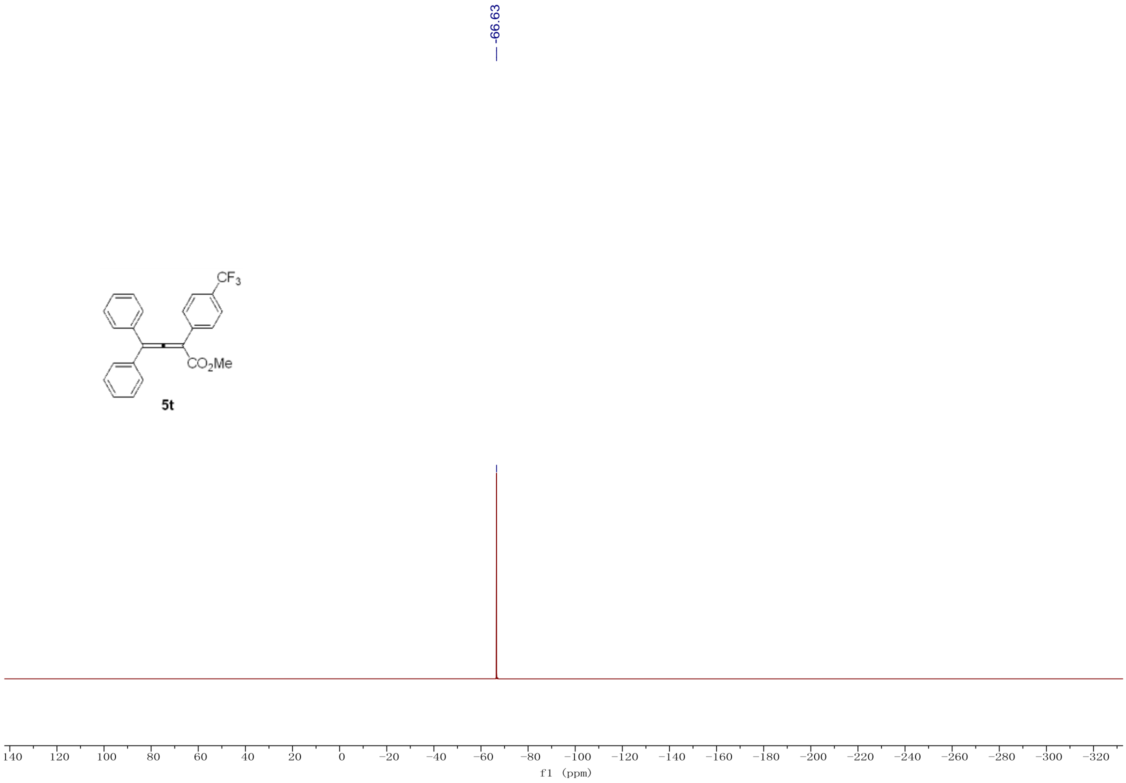
-66.63
CF3
CO2Me
5t
140 120 100 80 60 40 20 0 −20 −40 −60 −80 −100 −120 −140 −160 −180 −200 −220 −240 −260 −280 −300 −320
f1 (ppm)

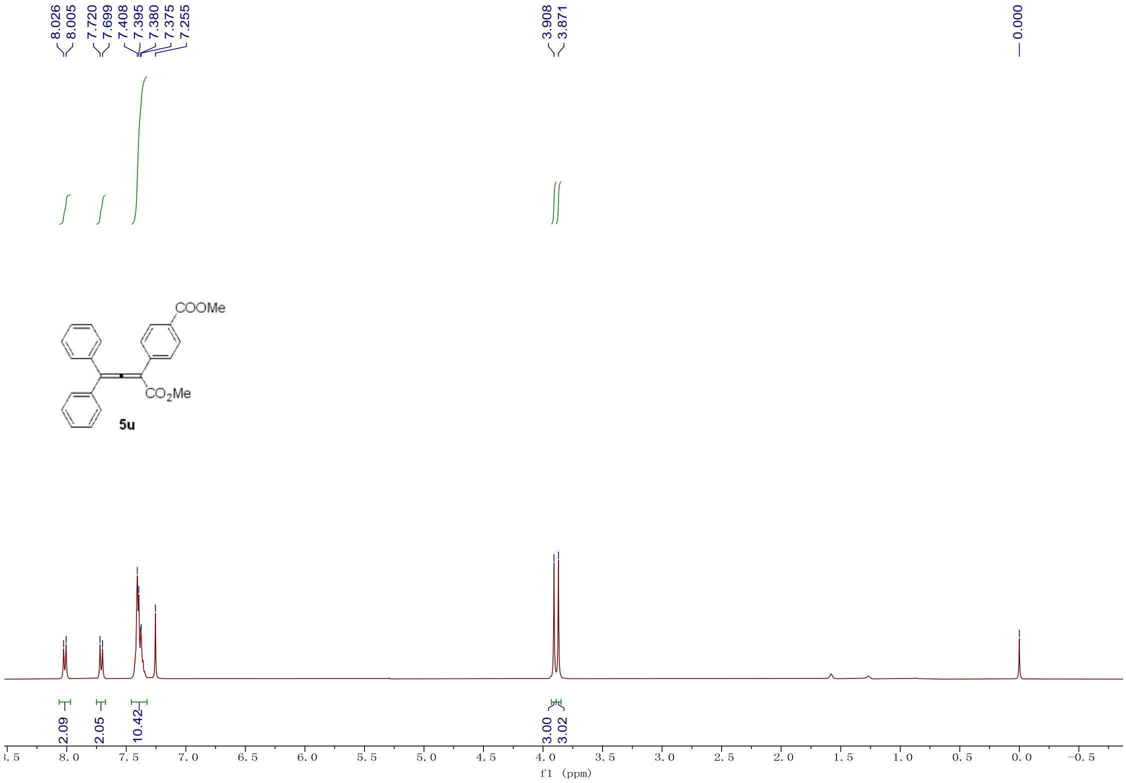
8.026
8.005
7.720
7.699
7.408
7.395
7.380
7.375
7.255
3.908
3.871
0.000
COOMe
CO2Me
5u
2.09
2.05
10.42
3.00
3.02
8.0 7.5 7.0 6.5 6.0 5.5 5.0 4.5 4.0 3.5 3.0 2.5 2.0 1.5 1.0 0.5 0.0 −0.5
f1 (ppm)

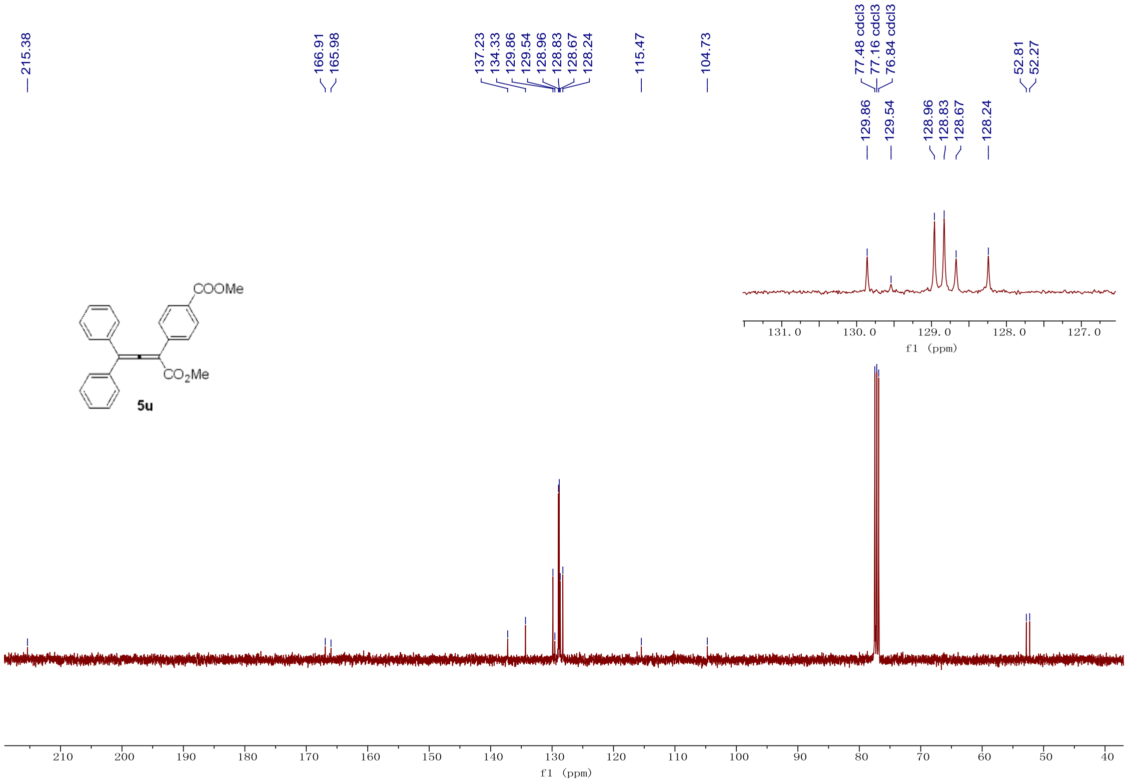

215.38
166.91
165.98
137.23
134.33
129.86
129.54
128.96
128.83
128.67
128.24
115.47
104.73
77.48 cdcl3
77.16 cdcl3
76.84 cdcl3
52.81
52.27
COOMe
$CO_2Me$
5u
f1 (ppm)


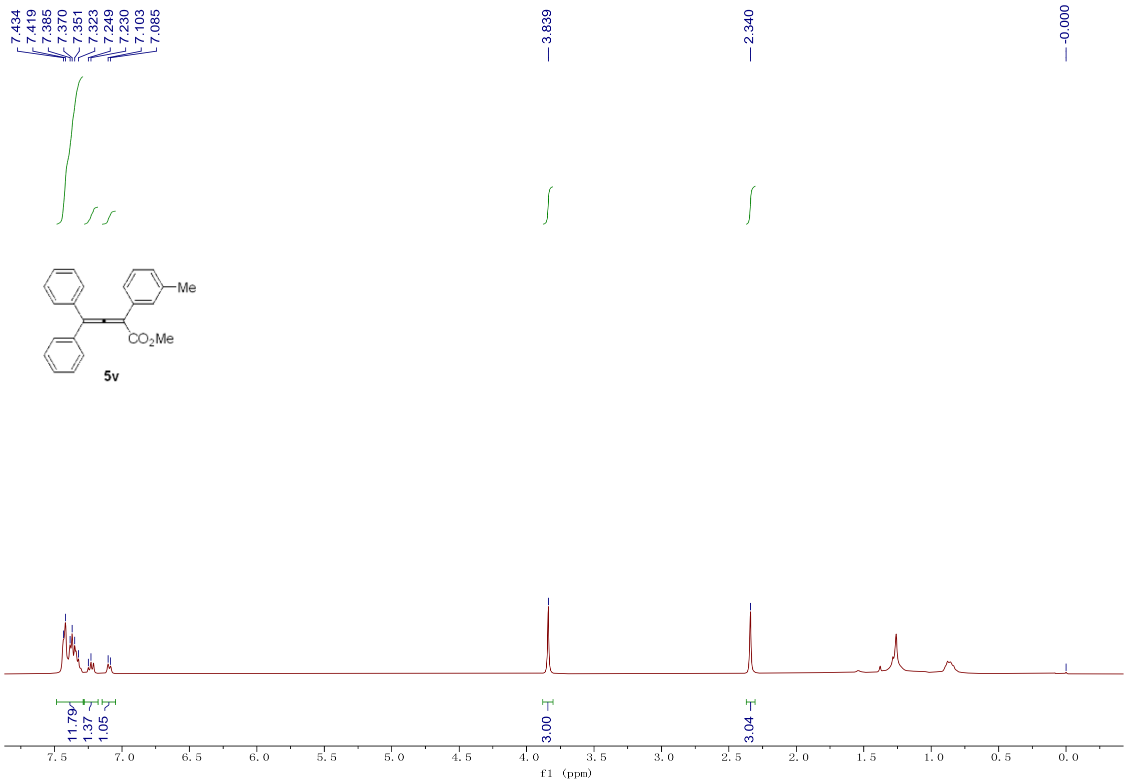

7.434
7.419
7.385
7.370
7.351
7.323
7.249
7.230
7.103
7.085
3.839
2.340
-0.000
Me
$CO_2Me$
5v
11.79
1.37
1.05
3.00
3.04
f1 (ppm)

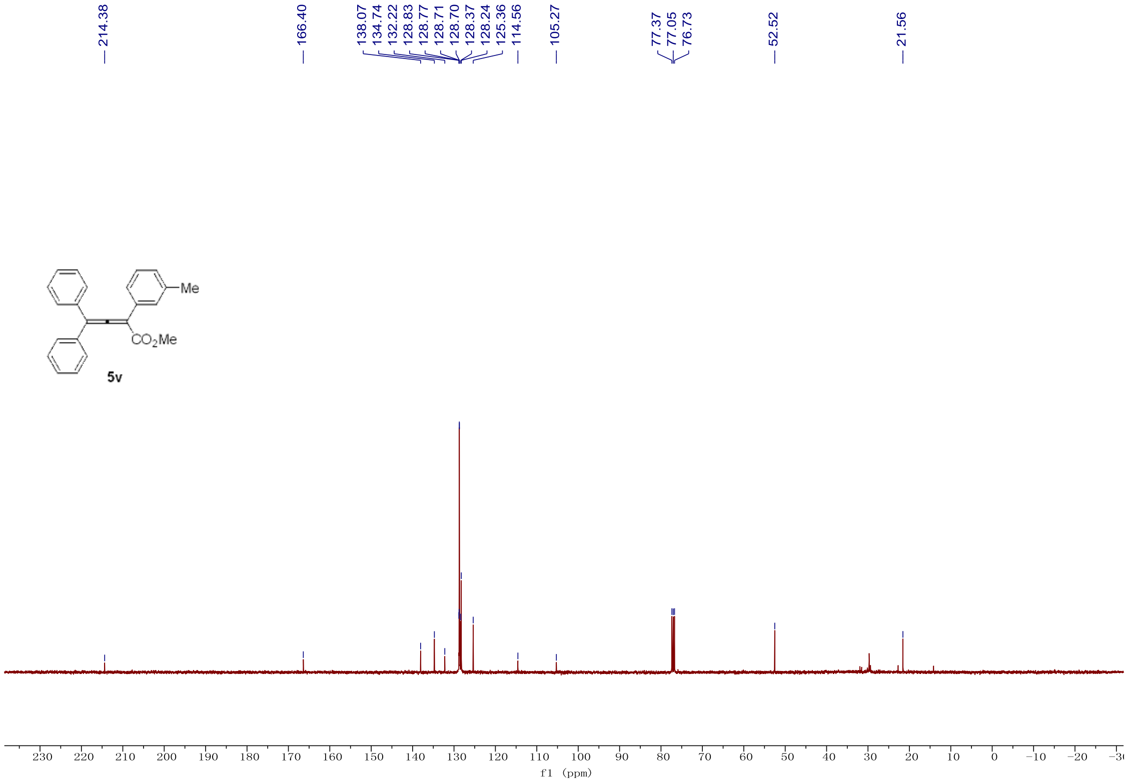
214.38
166.40
138.07
134.74
132.22
128.83
128.77
128.71
128.70
128.37
128.24
125.36
114.56
105.27
77.37
77.05
76.73
52.52
21.56
Me
CO2Me
5v
f1 (ppm)

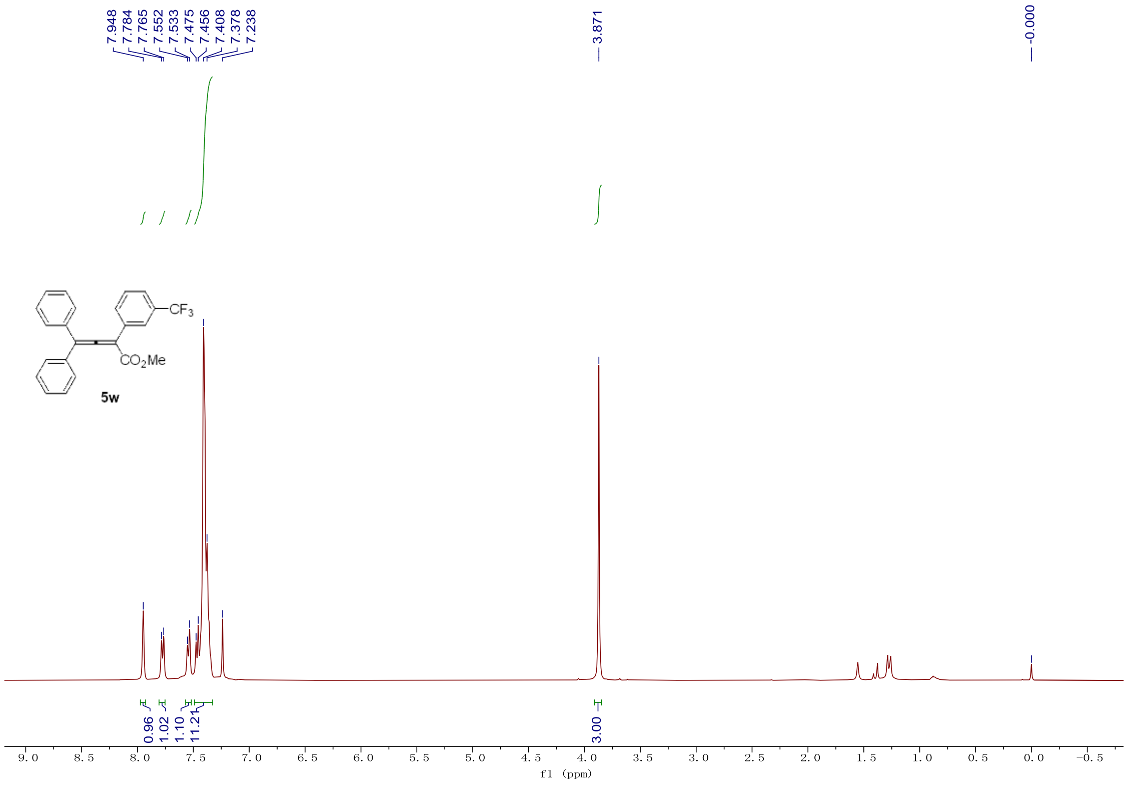
7.948
7.784
7.765
7.552
7.533
7.475
7.456
7.408
7.378
7.238
3.871
-0.000
CF3
CO2Me
5w
0.96
1.02
1.10
11.21
3.00
f1 (ppm)

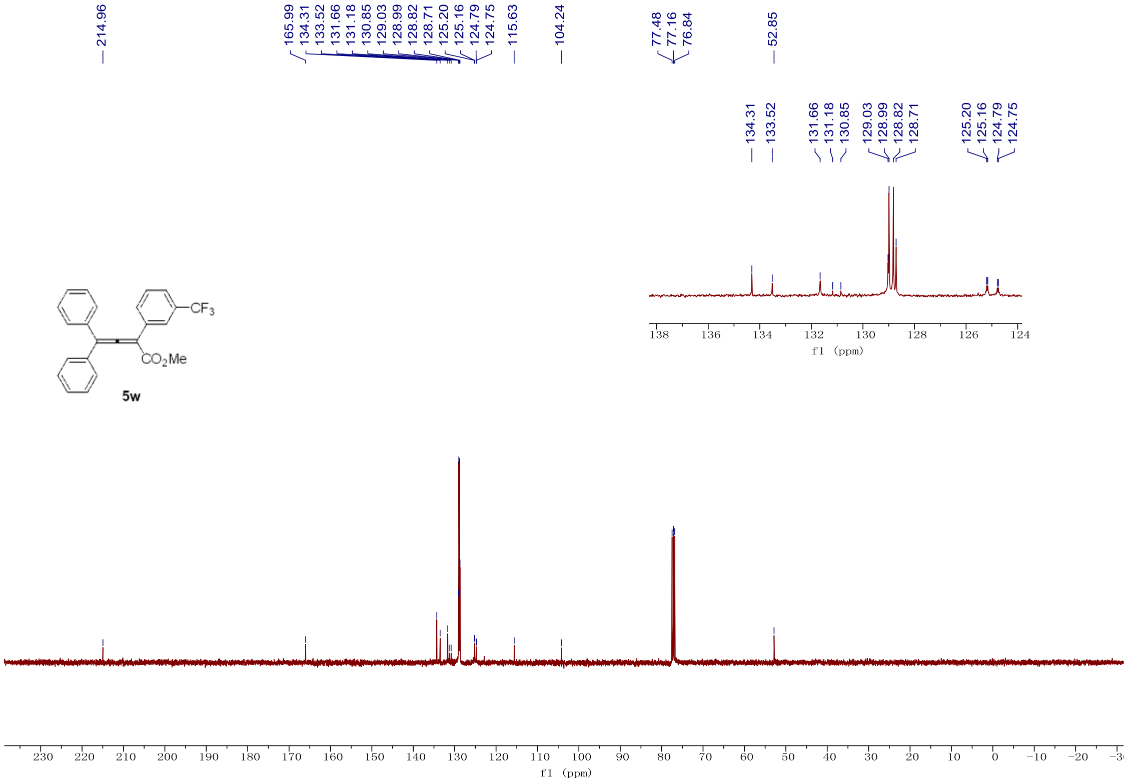

214.96
165.99
134.31
133.52
131.66
131.18
130.85
129.03
128.99
128.82
128.71
125.20
125.16
124.79
124.75
115.63
104.24
77.48
77.16
76.84
52.85
f1 (ppm)
CF3
CO2Me


**5w**

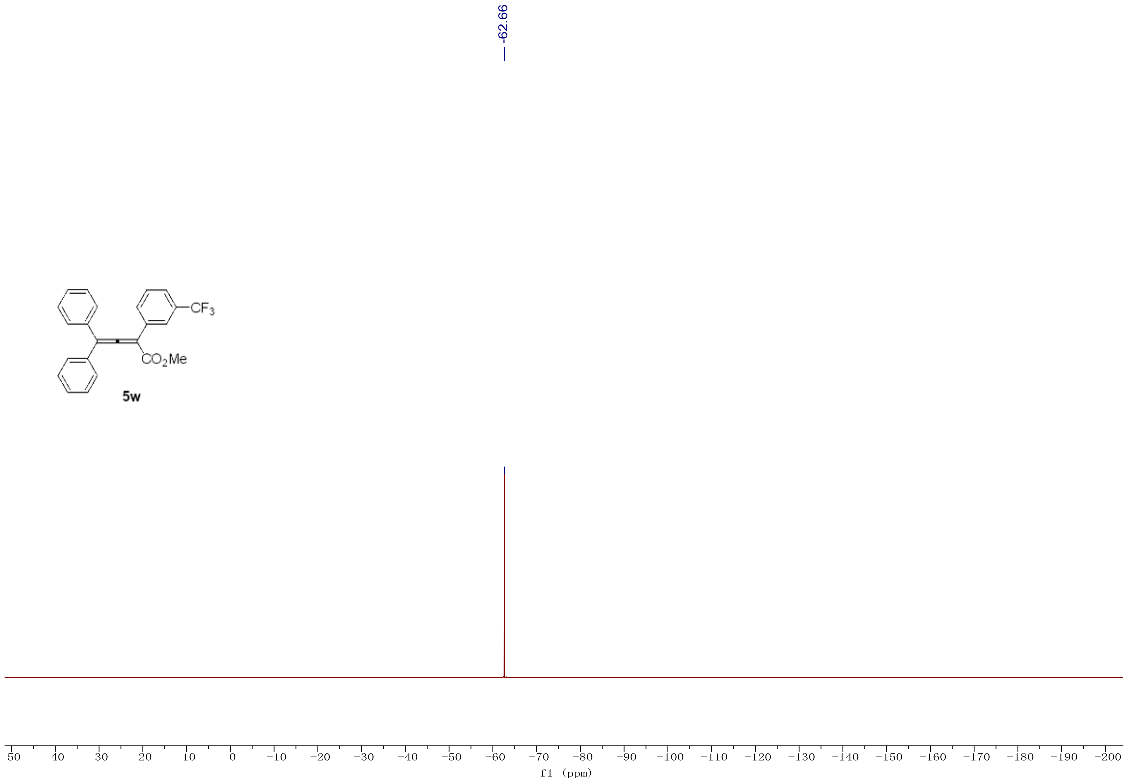

-62.66
f1 (ppm)
CF3
CO2Me


**5w**

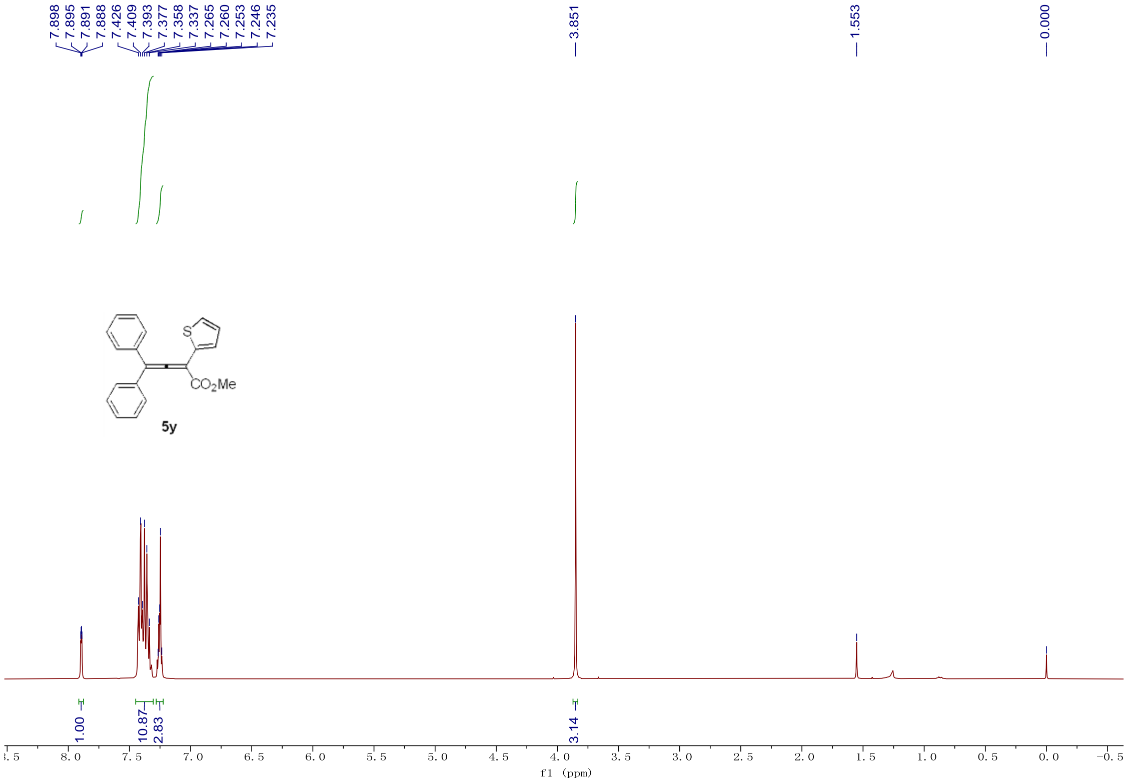
7.898
7.895
7.891
7.888
7.426
7.409
7.393
7.377
7.358
7.337
7.265
7.260
7.253
7.246
7.235
3.851
1.553
0.000
CO2Me
S
5y
1.00
10.87
2.83
3.14
f1 (ppm)

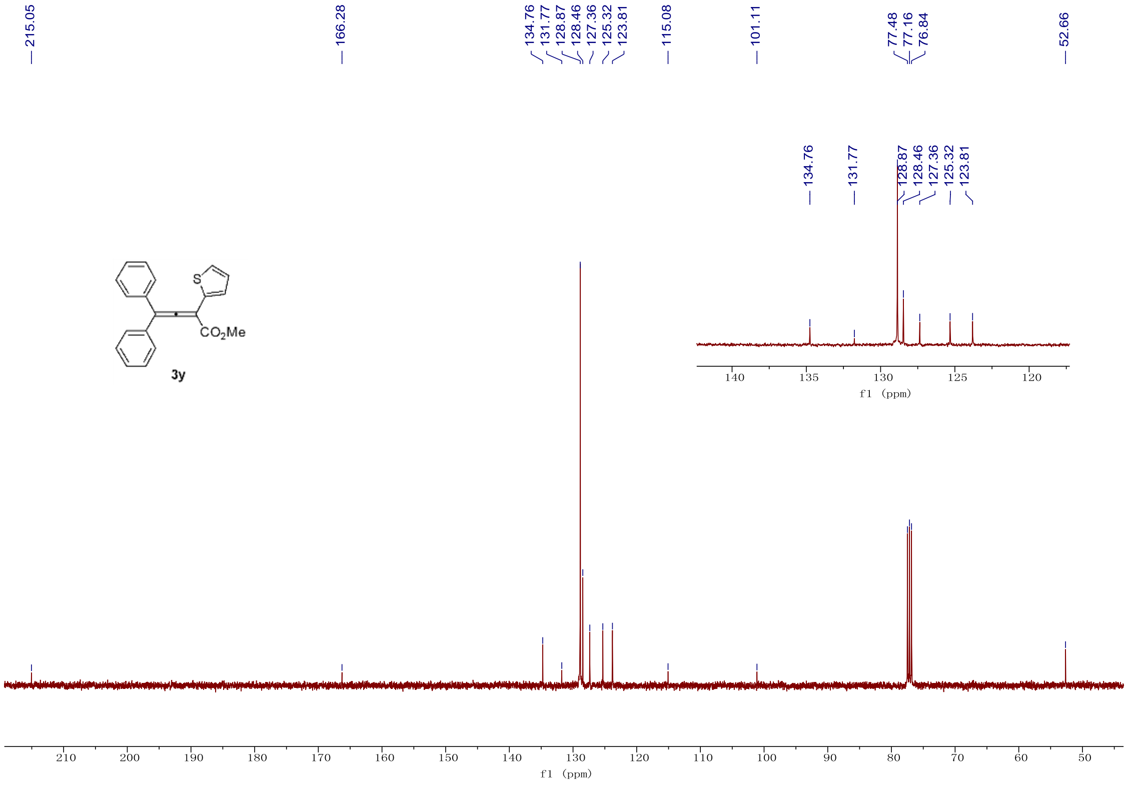
215.05
166.28
134.76
131.77
128.87
128.46
127.36
125.32
123.81
115.08
101.11
77.48
77.16
76.84
52.66
CO2Me
S
3y
f1 (ppm)

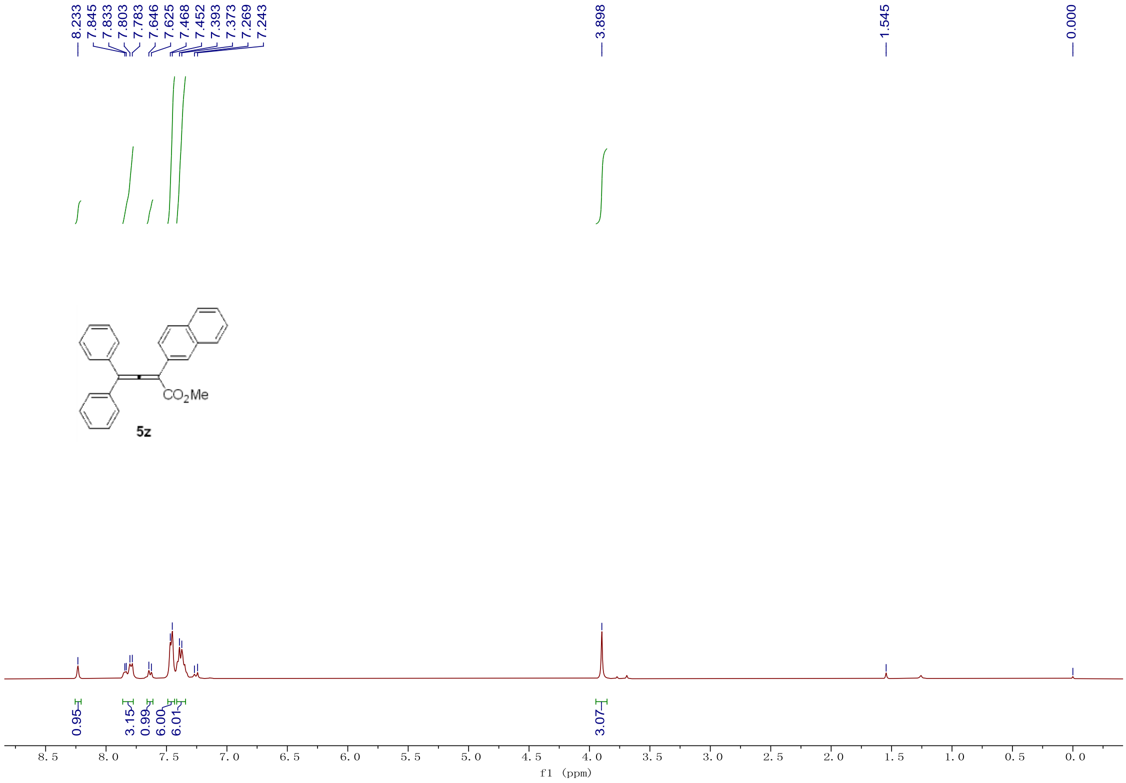
8.233
7.845
7.833
7.803
7.783
7.646
7.625
7.468
7.452
7.393
7.373
7.269
7.243
3.898
1.545
0.000
CO2Me
5z
0.95
3.15
0.99
6.00
6.01
3.07
f1 (ppm)

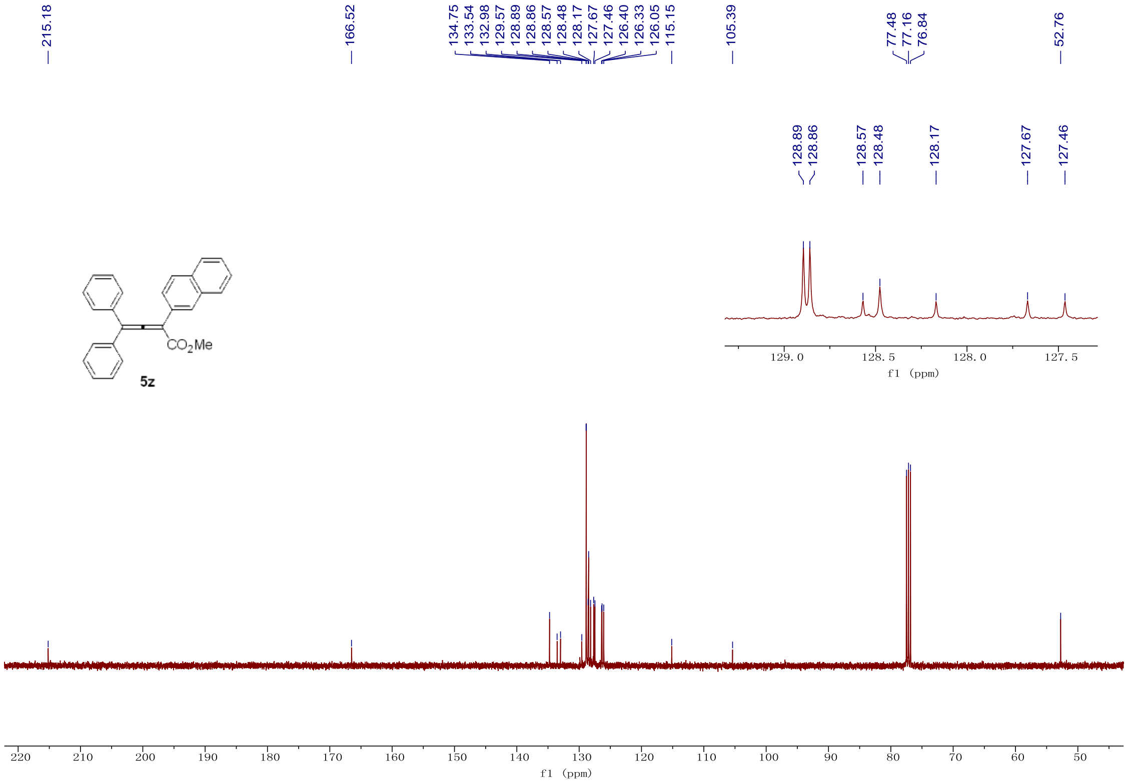
215.18
166.52
134.75
133.54
132.98
129.57
128.89
128.86
128.57
128.48
128.17
127.67
127.46
126.40
126.33
126.05
115.15
105.39
77.48
77.16
76.84
52.76
CO2Me
5z
f1 (ppm)

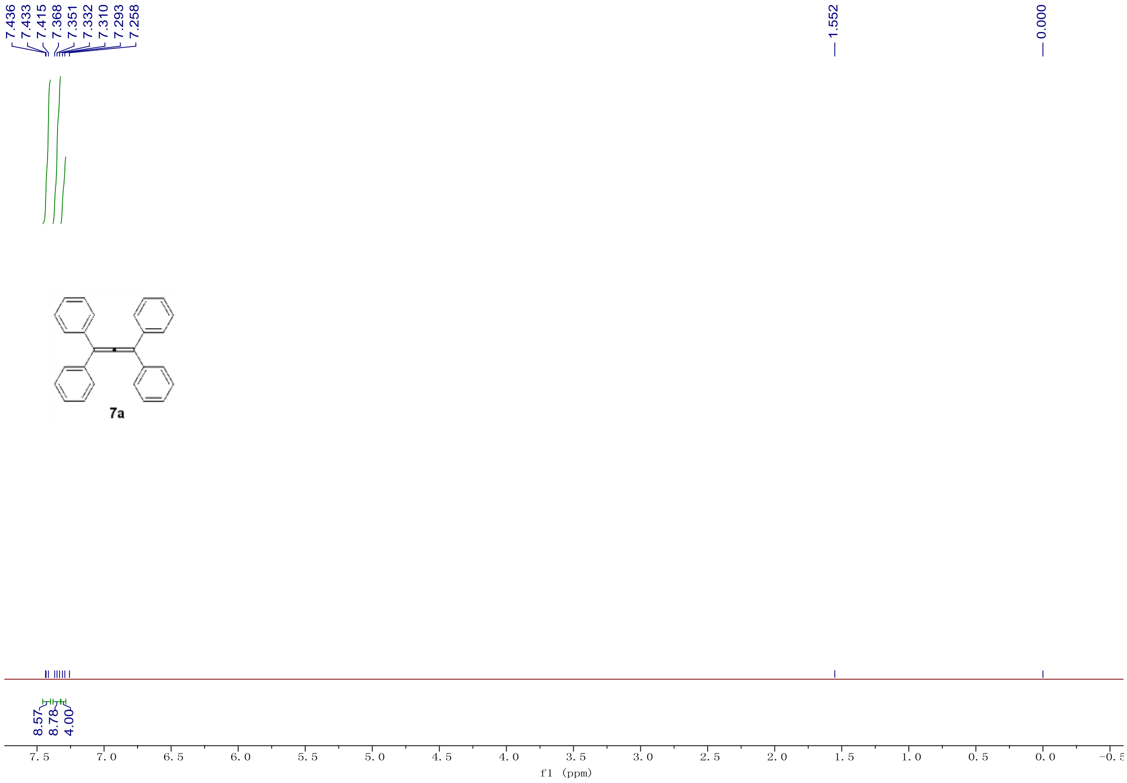
7.436
7.433
7.415
7.368
7.351
7.332
7.310
7.293
7.258
1.552
0.000
7a
8.57
8.78
4.00
7.5 7.0 6.5 6.0 5.5 5.0 4.5 4.0 3.5 3.0 2.5 2.0 1.5 1.0 0.5 0.0
f1 (ppm)

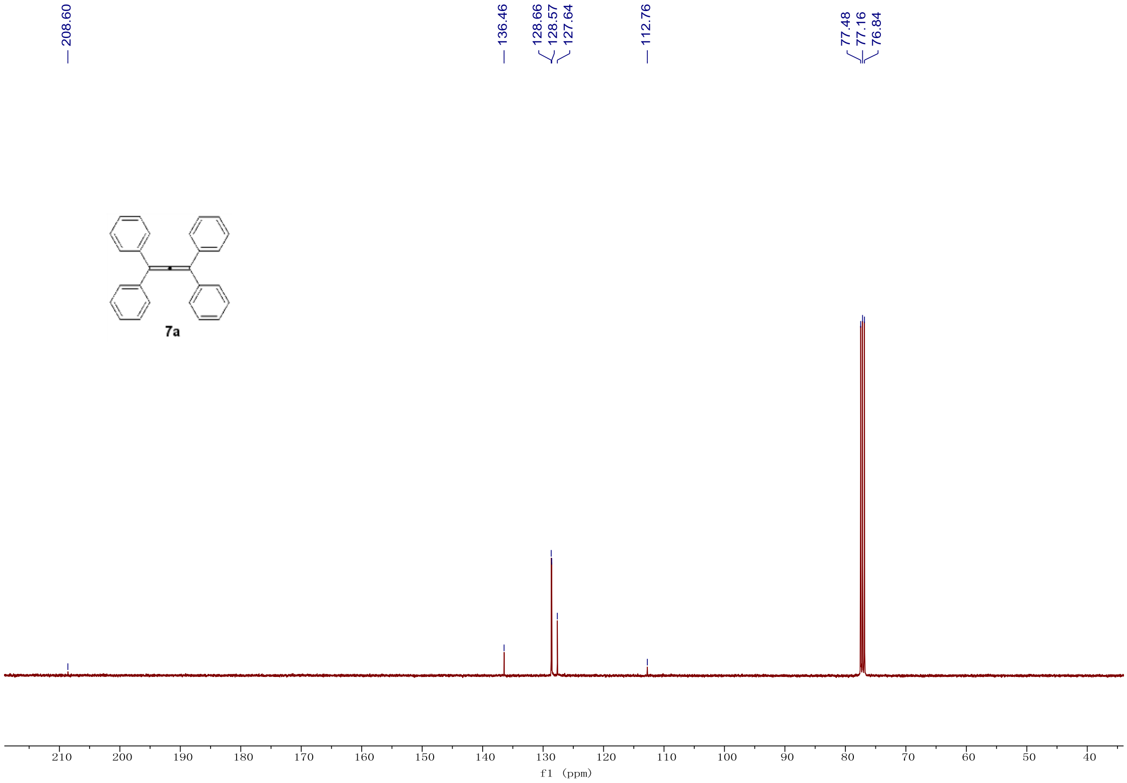
208.60
136.46
128.66
128.57
127.64
112.76
77.48
77.16
76.84
7a
210 200 190 180 170 160 150 140 130 120 110 100 90 80 70 60 50 40
f1 (ppm)

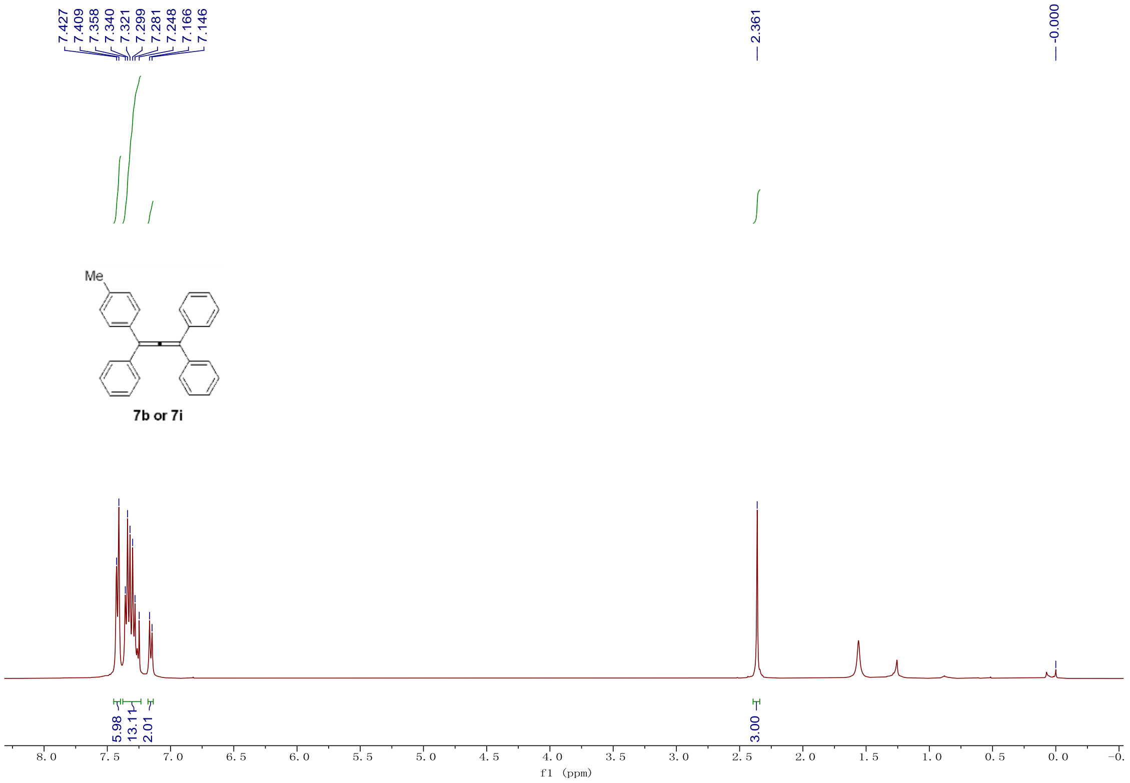
7.427
7.409
7.358
7.340
7.321
7.299
7.281
7.248
7.166
7.146
2.361
-0.000
Me
7b or 7i
5.98
13.11
2.01
3.00
8.0 7.5 7.0 6.5 6.0 5.5 5.0 4.5 4.0 3.5 3.0 2.5 2.0 1.5 1.0 0.5 0.0
f1 (ppm)

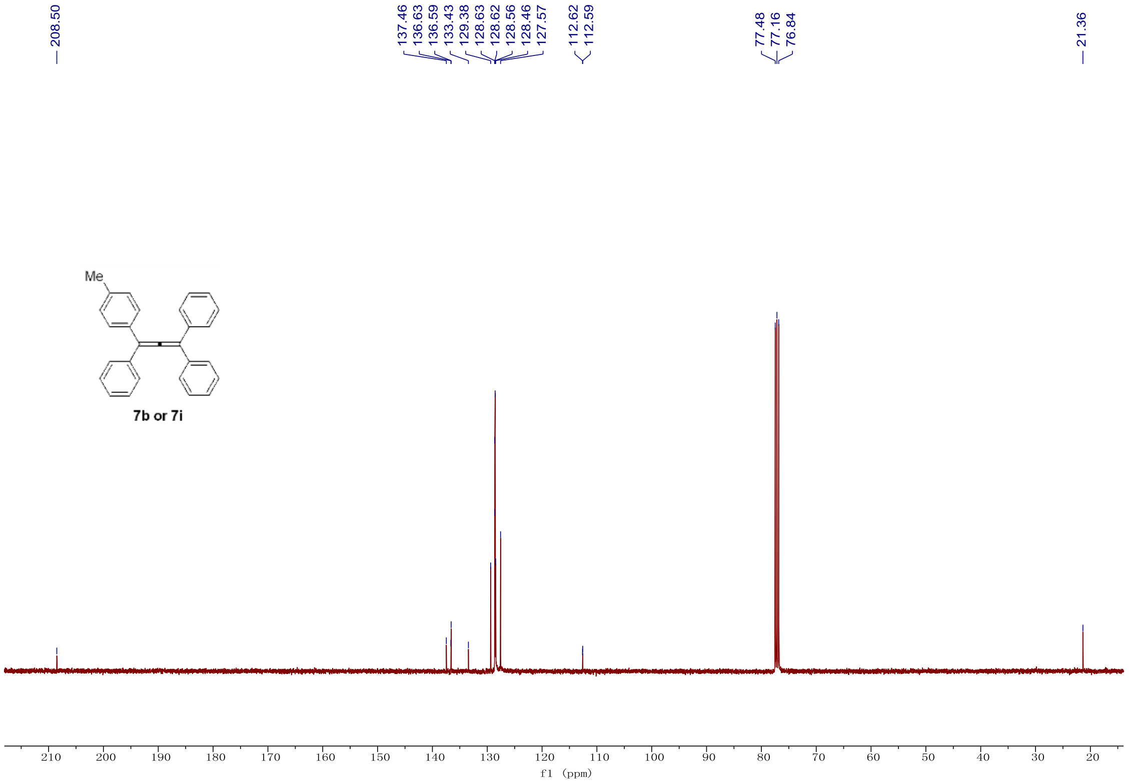
208.50
137.46
136.63
136.59
133.43
129.38
128.63
128.62
128.56
128.46
127.57
112.62
112.59
77.48
77.16
76.84
21.36
Me
7b or 7i
210 200 190 180 170 160 150 140 130 120 110 100 90 80 70 60 50 40 30 20
f1 (ppm)

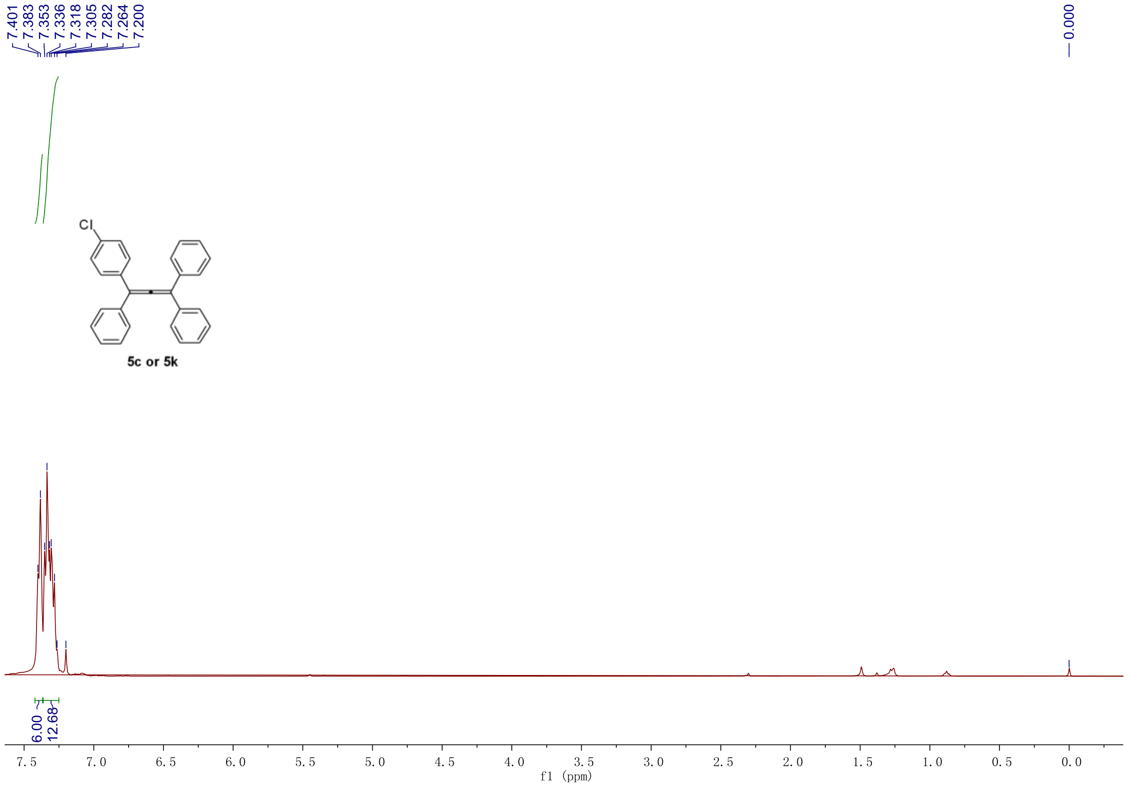
7.401
7.383
7.353
7.336
7.318
7.305
7.282
7.264
7.200
0.000
Cl
5c or 5k
6.00
12.68
7.5 7.0 6.5 6.0 5.5 5.0 4.5 4.0 3.5 3.0 2.5 2.0 1.5 1.0 0.5 0.0
f1 (ppm)

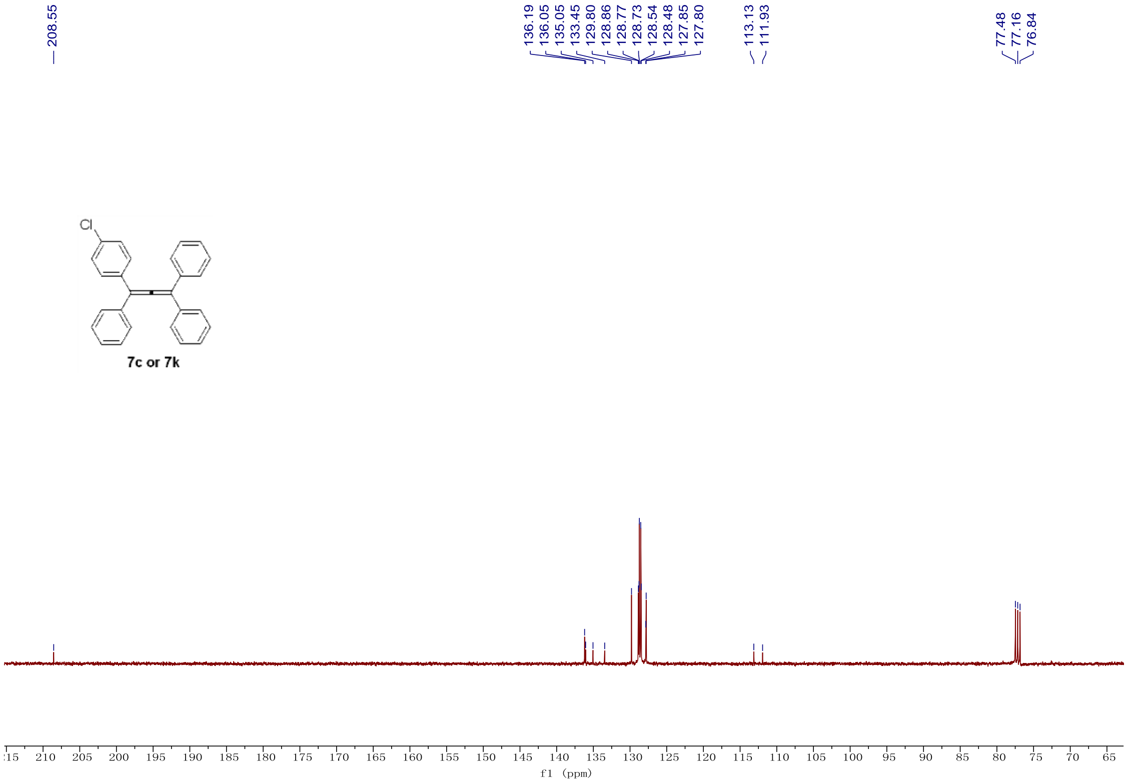
208.55
136.19
136.05
135.05
133.45
129.80
128.86
128.77
128.73
128.54
128.48
127.85
127.80
113.13
111.93
77.48
77.16
76.84
Cl
7c or 7k
210 205 200 195 190 185 180 175 170 165 160 155 150 145 140 135 130 125 120 115 110 105 100 95 90 85 80 75 70 65
f1 (ppm)

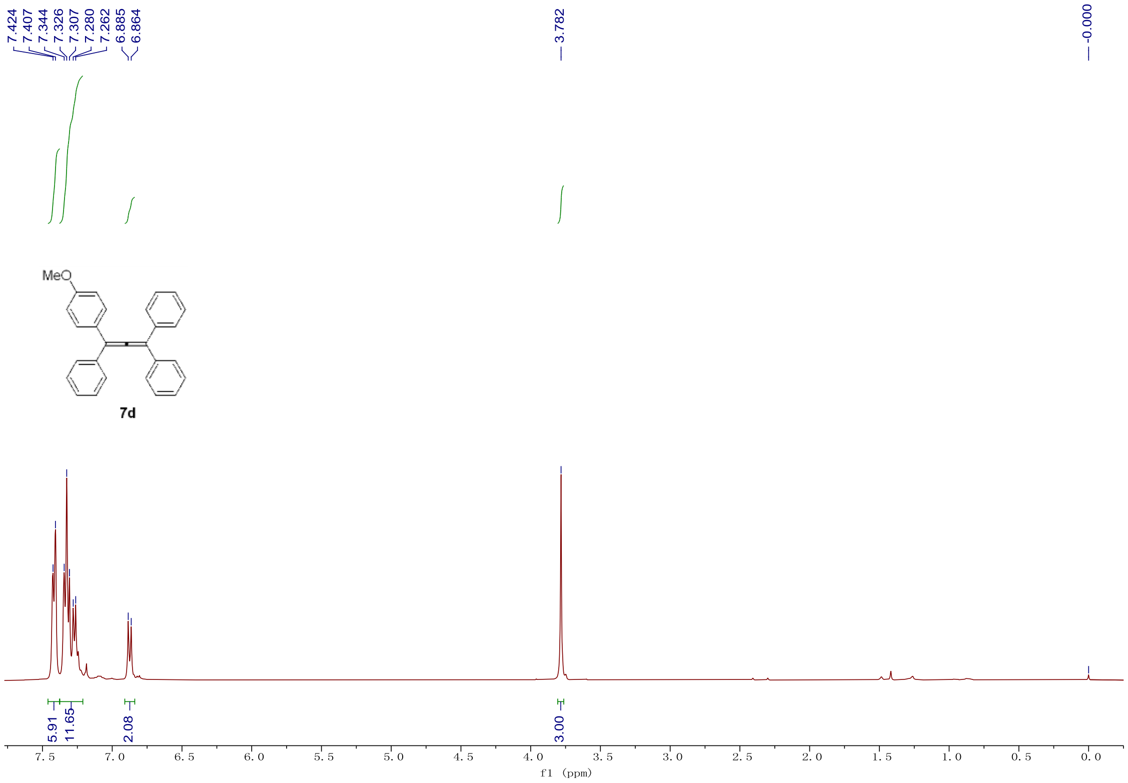
7.424
7.407
7.344
7.326
7.307
7.280
7.262
6.885
6.864
3.782
-0.000
MeO
7d
5.91
11.65
2.08
3.00
7.5 7.0 6.5 6.0 5.5 5.0 4.5 4.0 3.5 3.0 2.5 2.0 1.5 1.0 0.5 0.0
f1 (ppm)

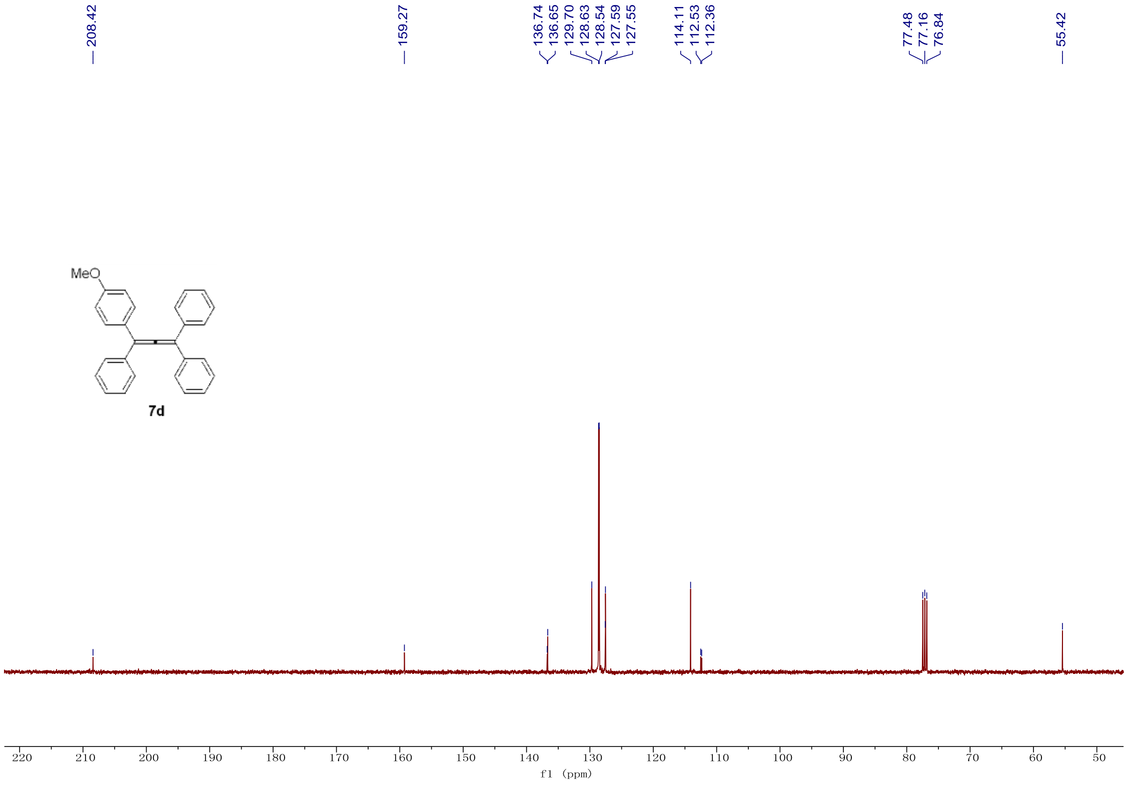
208.42
159.27
136.74
136.65
129.70
128.63
128.54
127.59
127.55
114.11
112.53
112.36
77.48
77.16
76.84
55.42
MeO
7d
220 210 200 190 180 170 160 150 140 130 120 110 100 90 80 70 60 50
f1 (ppm)

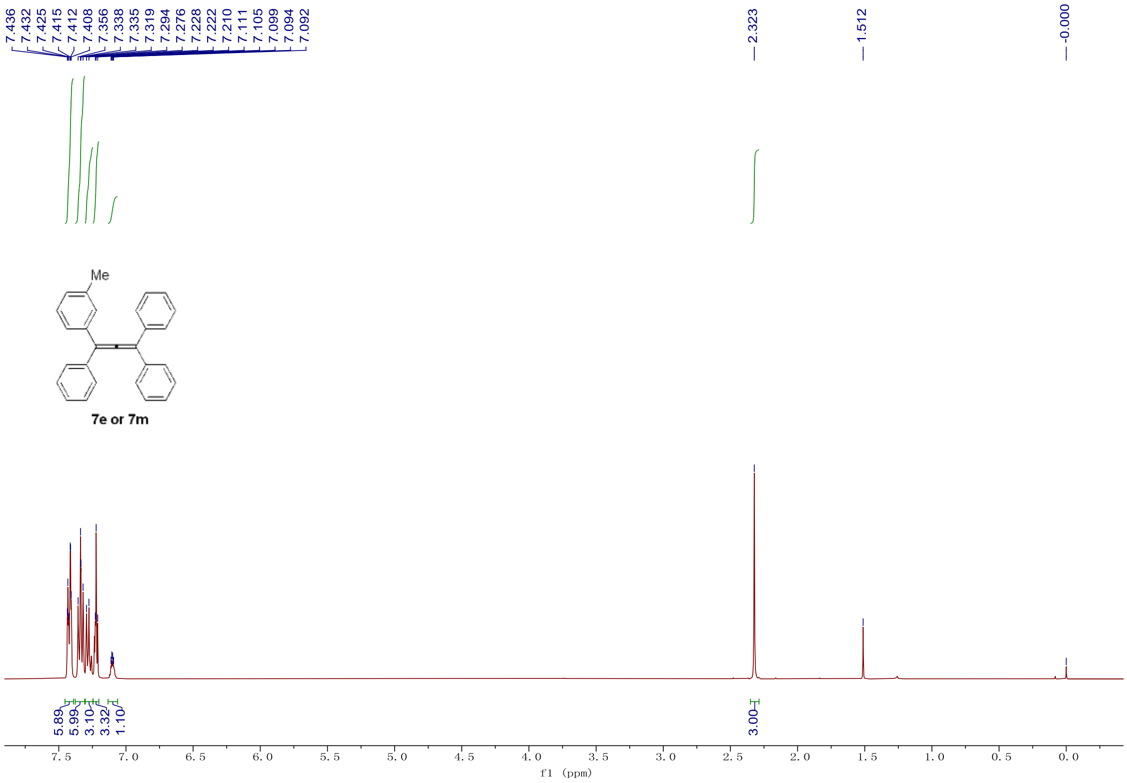
7.436
7.432
7.425
7.415
7.412
7.408
7.356
7.338
7.335
7.319
7.294
7.276
7.228
7.222
7.210
7.111
7.105
7.099
7.094
7.092
2.323
1.512
-0.000
Me
7e or 7m
5.89
5.99
3.10
3.32
1.10
3.00
f1 (ppm)

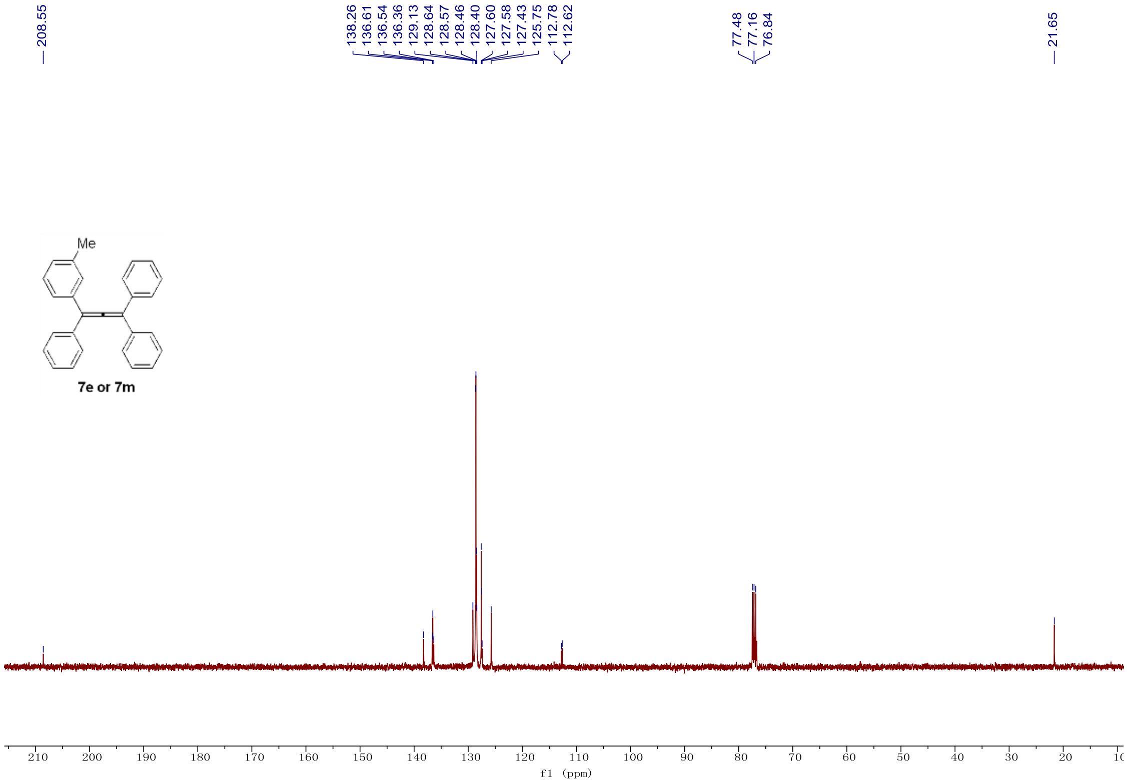
208.55
138.26
136.61
136.54
136.36
129.13
128.64
128.57
128.46
128.40
127.60
127.58
127.43
125.75
112.78
112.62
77.48
77.16
76.84
21.65
Me
7e or 7m
f1 (ppm)

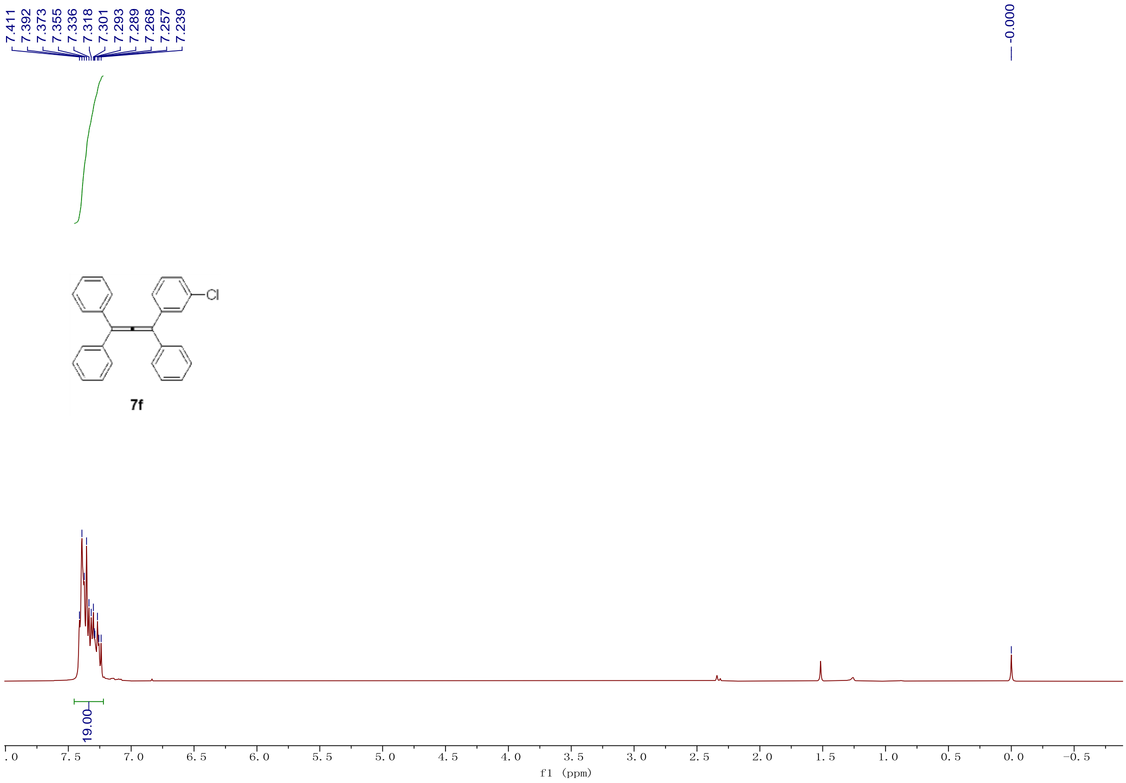
7.411
7.392
7.373
7.355
7.336
7.318
7.301
7.293
7.289
7.268
7.257
7.239
-0.000
Cl
7f
19.00
f1 (ppm)

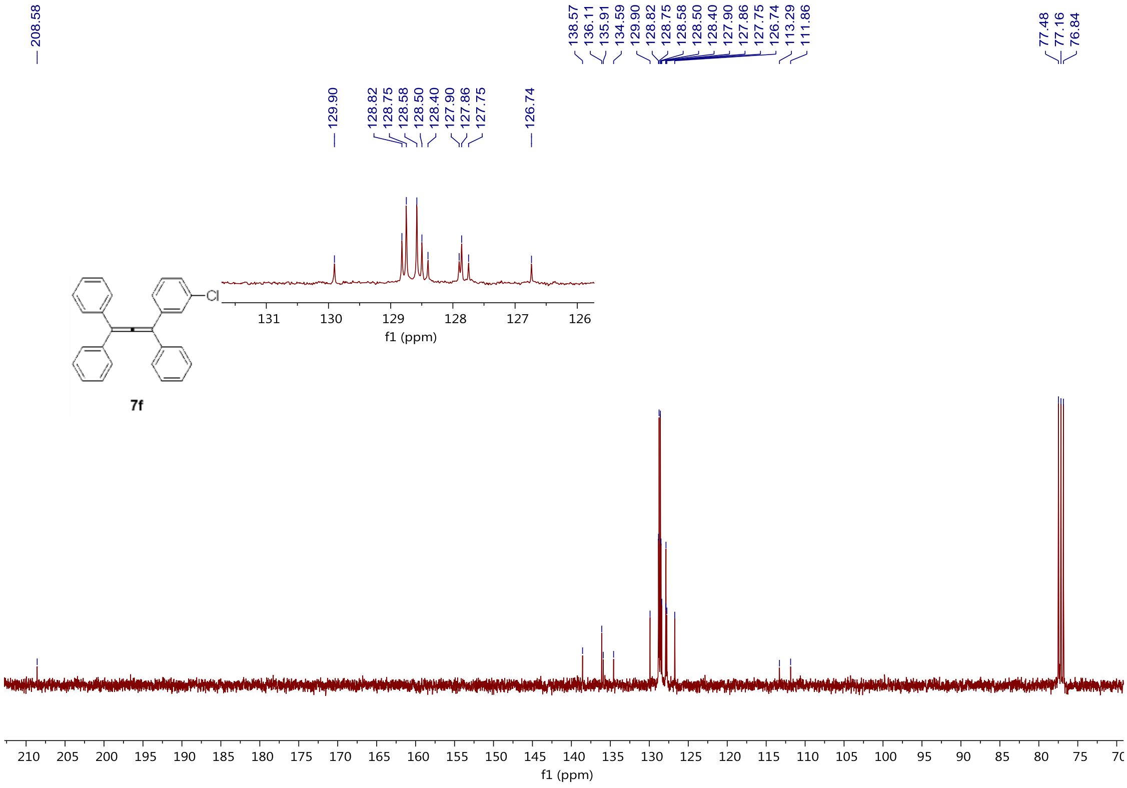
208.58
138.57
136.11
135.91
134.59
129.90
128.82
128.75
128.58
128.50
128.40
127.90
127.86
127.75
126.74
113.29
111.86
77.48
77.16
76.84
Cl
7f
f1 (ppm)

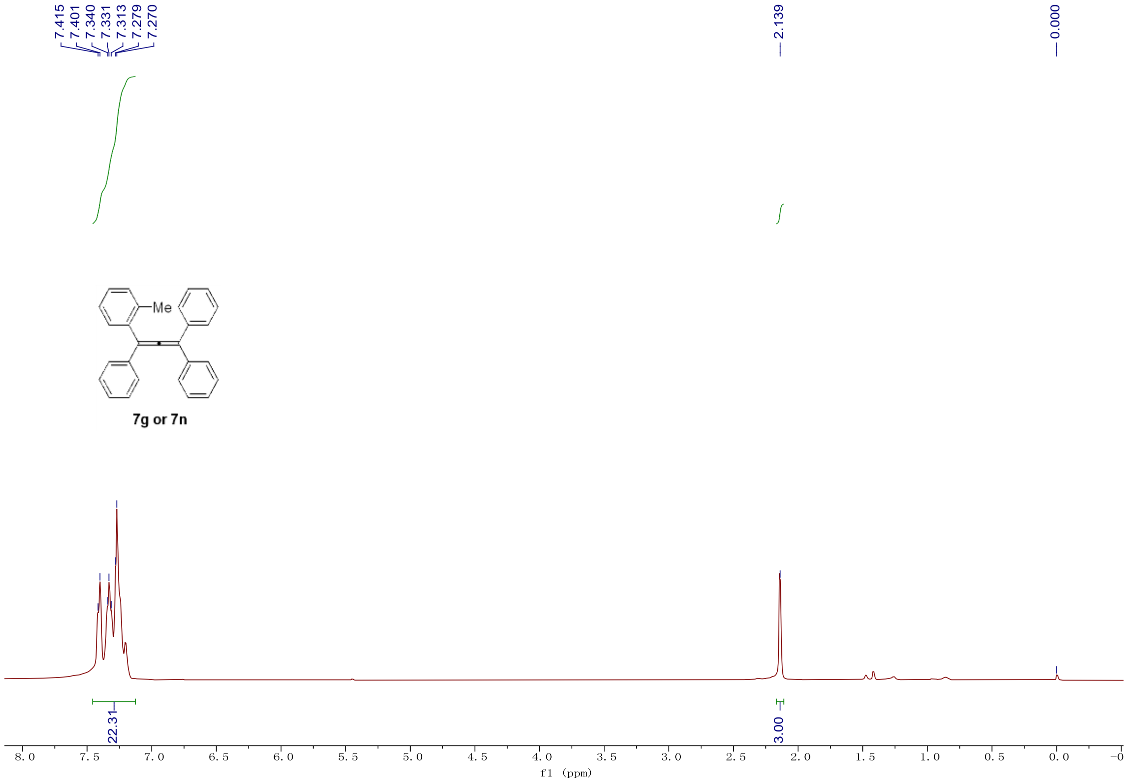
7.415
7.401
7.340
7.331
7.313
7.279
7.270
2.139
0.000
Me
7g or 7n
22.31
3.00
8.0 7.5 7.0 6.5 6.0 5.5 5.0 4.5 4.0 3.5 3.0 2.5 2.0 1.5 1.0 0.5 0.0
f1 (ppm)

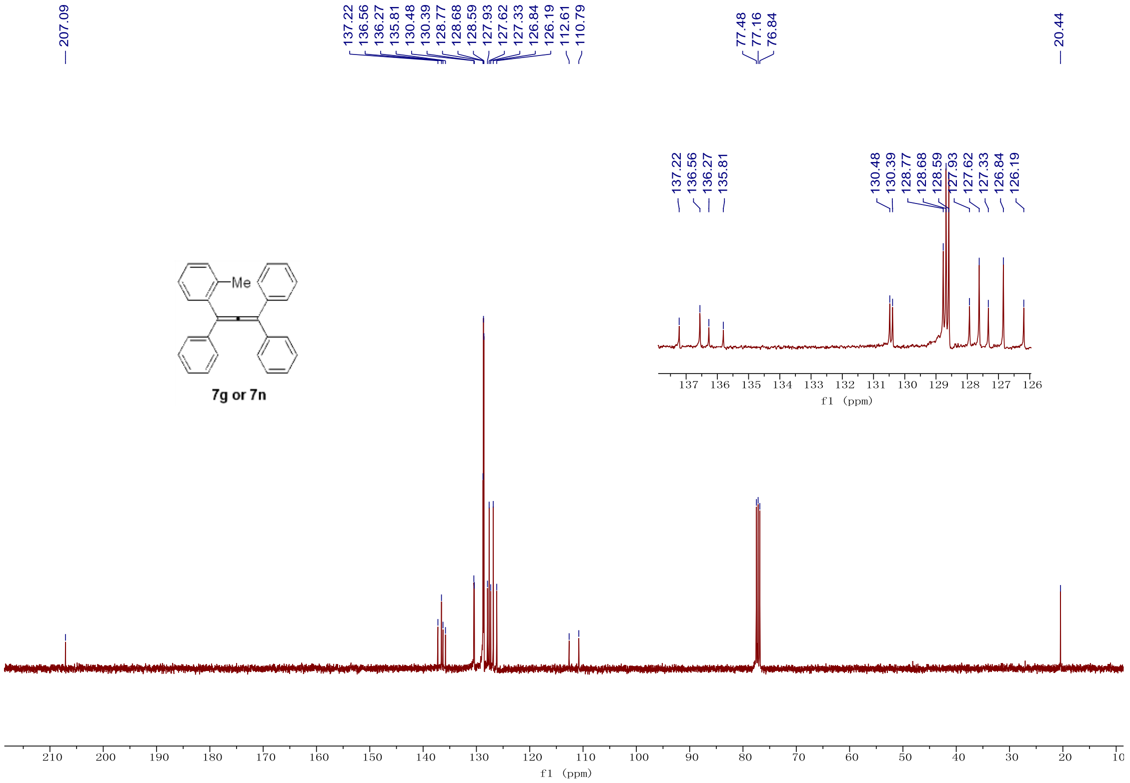
207.09
137.22
136.56
136.27
135.81
130.48
130.39
128.77
128.68
128.59
127.93
127.62
127.33
126.84
126.19
112.61
110.79
77.48
77.16
76.84
20.44
Me
7g or 7n
137 136 135 134 133 132 131 130 129 128 127 126
f1 (ppm)
210 200 190 180 170 160 150 140 130 120 110 100 90 80 70 60 50 40 30 20 10
f1 (ppm)

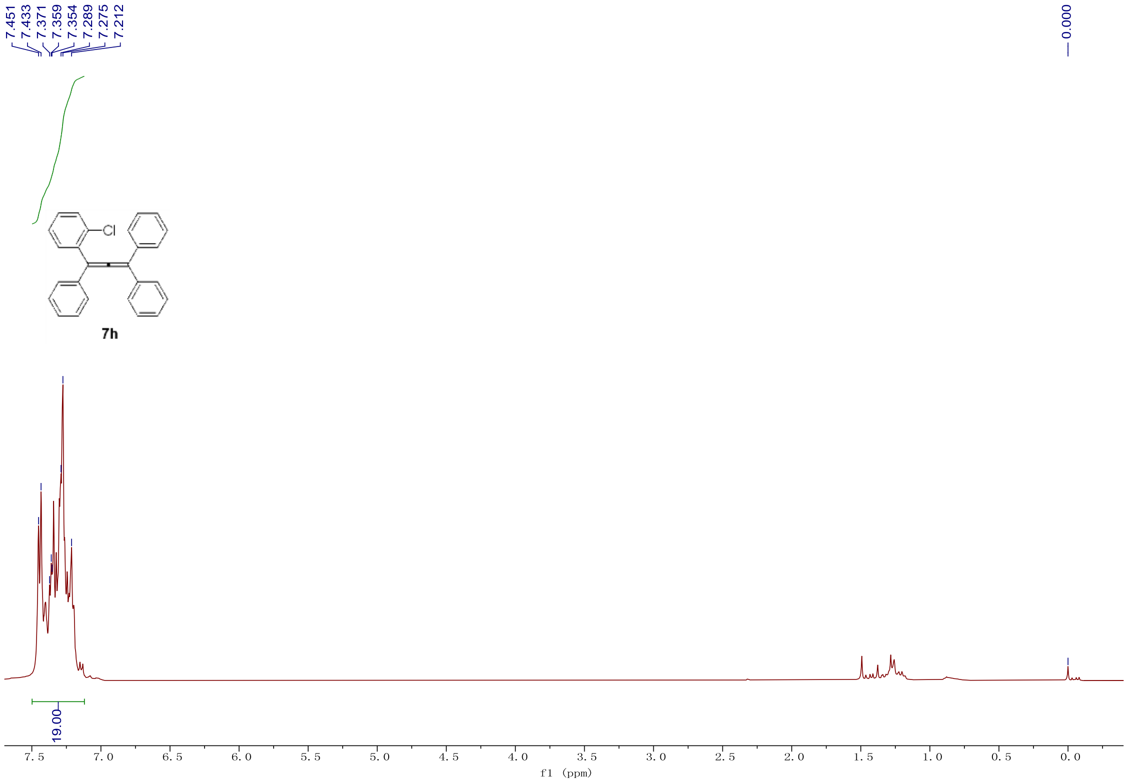
7.451
7.433
7.371
7.359
7.354
7.289
7.275
7.212
0.000
Cl
7h
19.00
7.5 7.0 6.5 6.0 5.5 5.0 4.5 4.0 3.5 3.0 2.5 2.0 1.5 1.0 0.5 0.0
f1 (ppm)

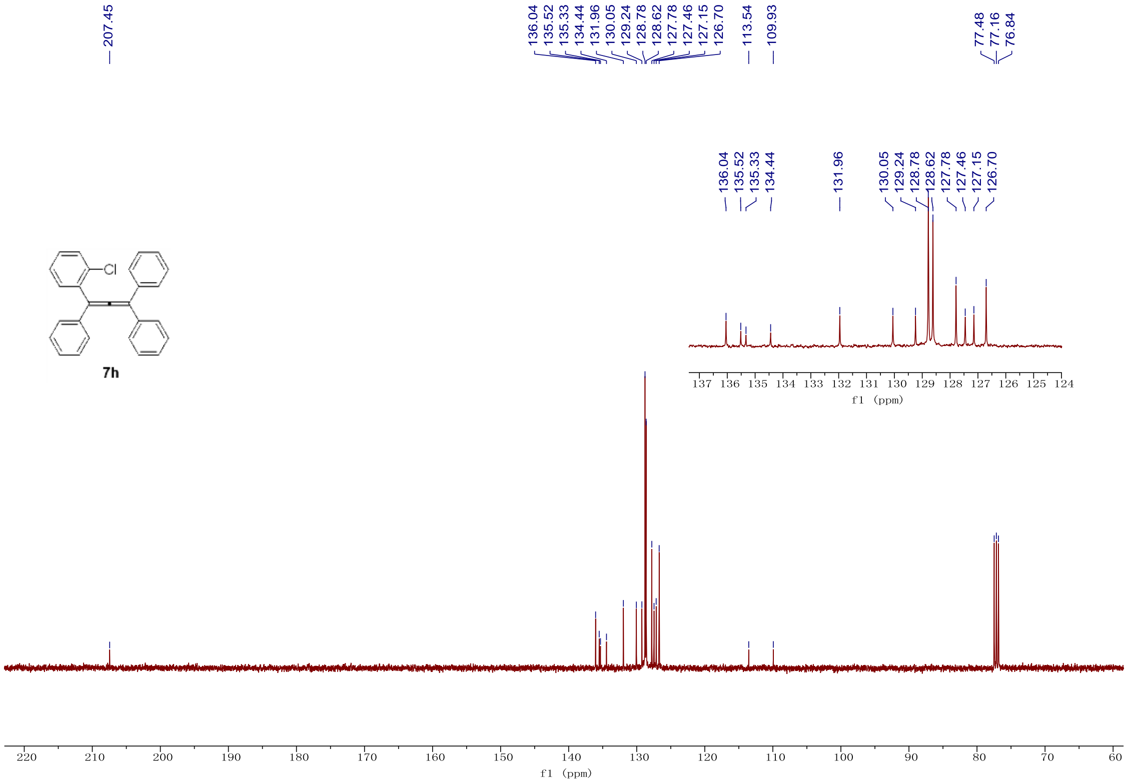
207.45
136.04
135.52
135.33
134.44
131.96
130.05
129.24
128.78
128.62
127.78
127.46
127.15
126.70
113.54
109.93
77.48
77.16
76.84
Cl
7h
137 136 135 134 133 132 131 130 129 128 127 126 125 124
f1 (ppm)
220 210 200 190 180 170 160 150 140 130 120 110 100 90 80 70 60
f1 (ppm)

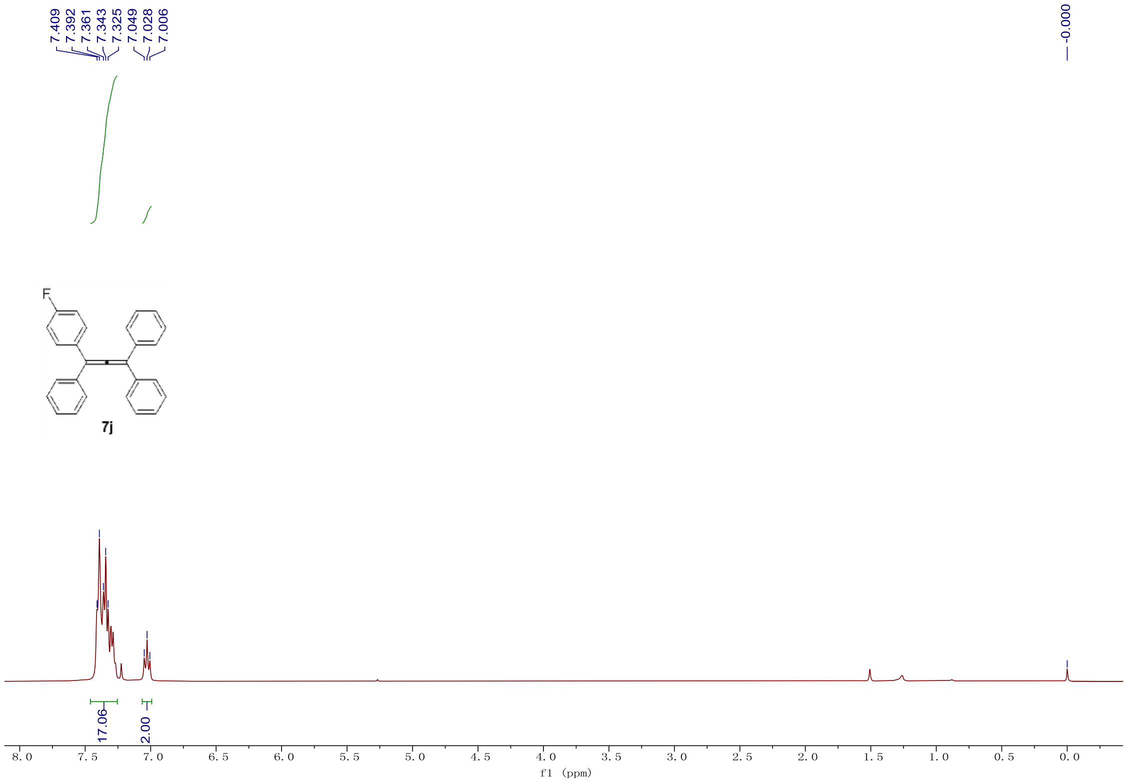
7.409
7.392
7.361
7.343
7.325
7.049
7.028
7.006
-0.000
F
7j
17.06
2.00
8.0 7.5 7.0 6.5 6.0 5.5 5.0 4.5 4.0 3.5 3.0 2.5 2.0 1.5 1.0 0.5 0.0
f1 (ppm)

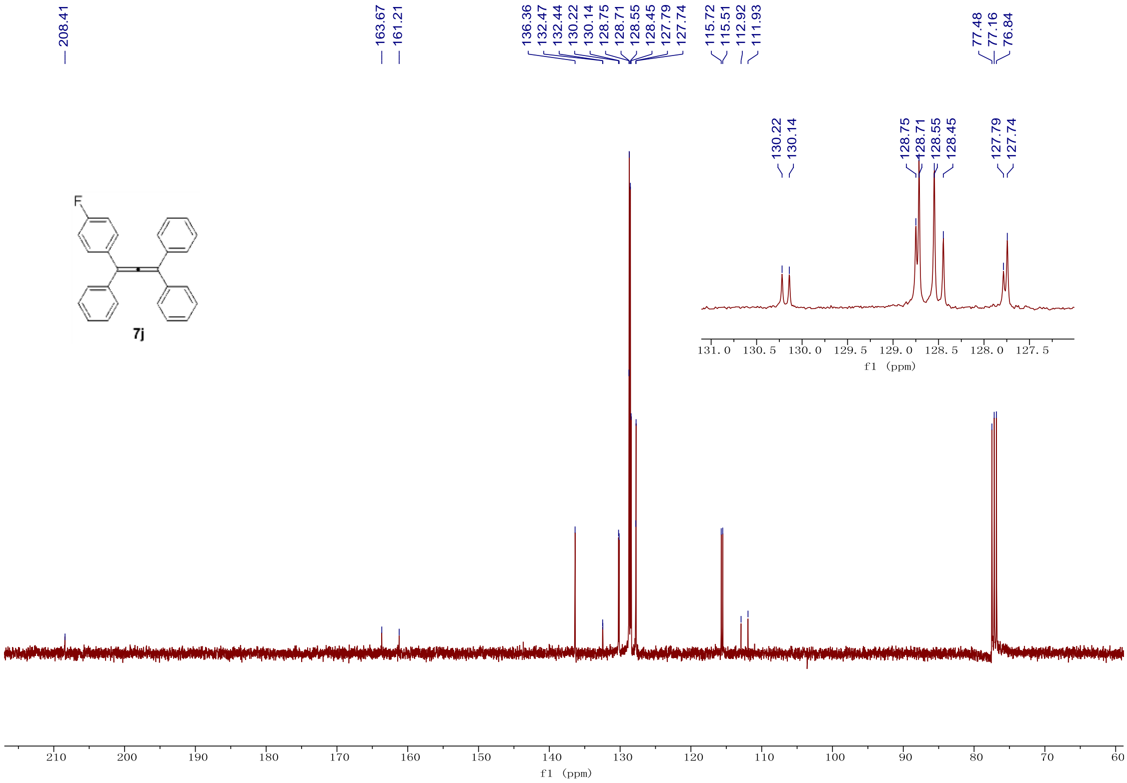
208.41
163.67
161.21
136.36
132.47
132.44
130.22
130.14
128.75
128.71
128.55
128.45
127.79
127.74
115.72
115.51
112.92
111.93
77.48
77.16
76.84
F
7j
131.0 130.5 130.0 129.5 129.0 128.5 128.0 127.5
210 200 190 180 170 160 150 140 130 120 110 100 90 80 70 60
f1 (ppm)

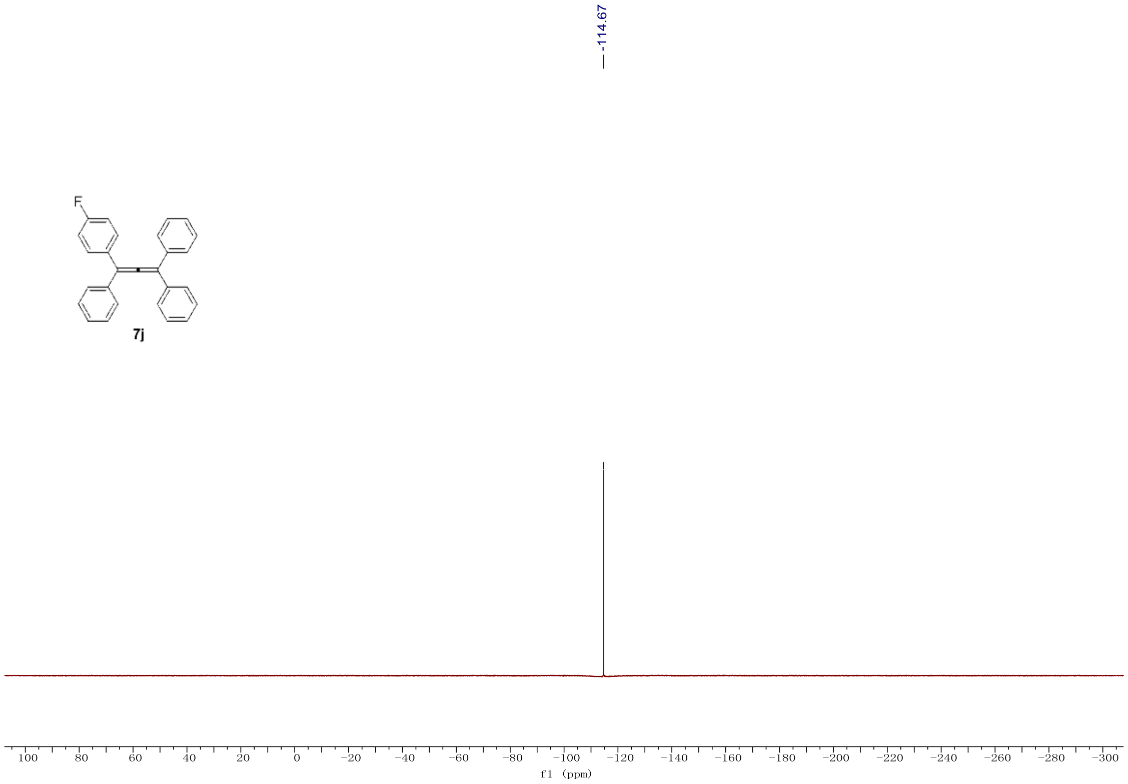

-114.67
F
7j
100 80 60 40 20 0 −20 −40 −60 −80 −100 −120 −140 −160 −180 −200 −220 −240 −260 −280 −300
f1 (ppm)


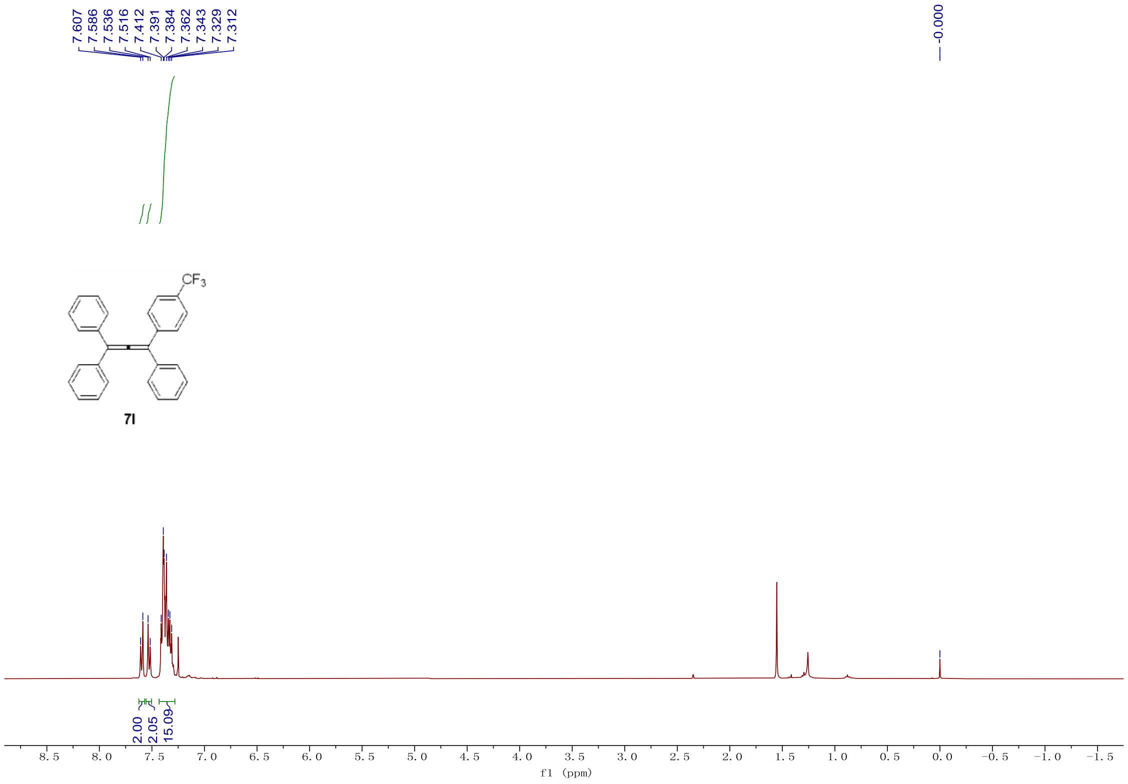

7.607
7.586
7.536
7.516
7.412
7.391
7.384
7.362
7.343
7.329
7.312
-0.000
CF3
7l
2.00
2.05
15.09
8.5 8.0 7.5 7.0 6.5 6.0 5.5 5.0 4.5 4.0 3.5 3.0 2.5 2.0 1.5 1.0 0.5 0.0 −0.5 −1.0 −1.5
f1 (ppm)

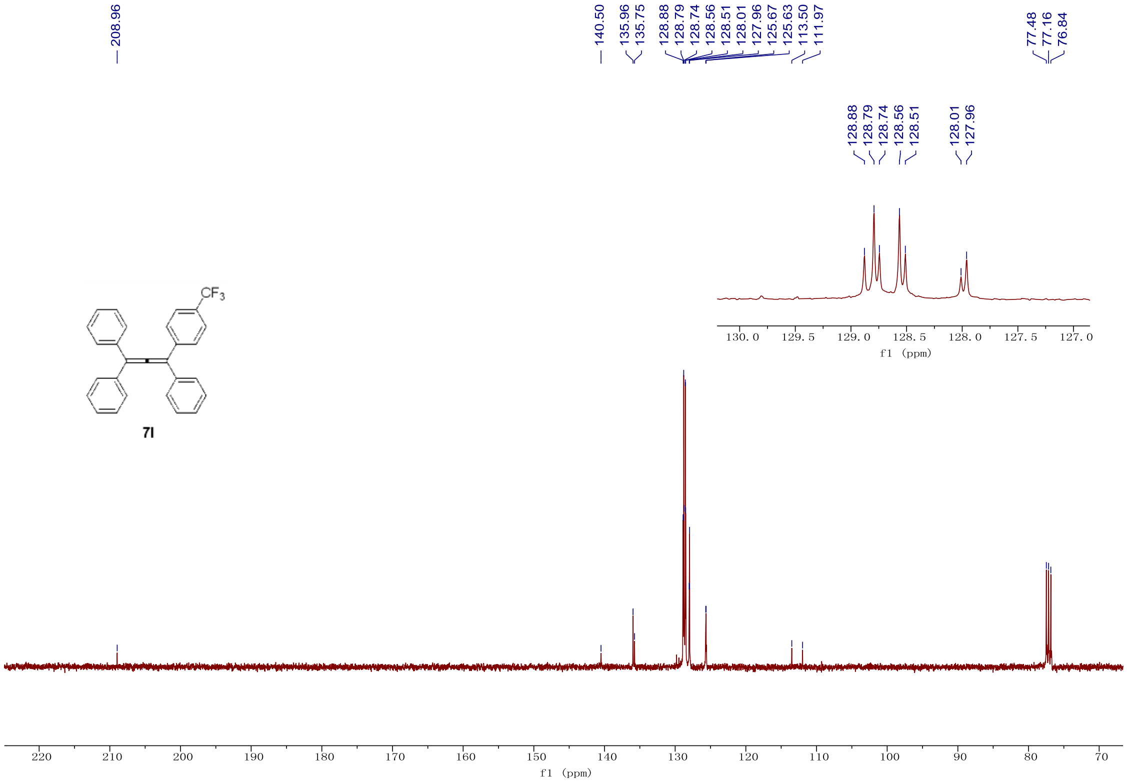
208.96
140.50
135.96
135.75
128.88
128.79
128.74
128.56
128.51
128.01
127.96
125.67
125.63
113.50
111.97
77.48
77.16
76.84
CF3
7l
f1 (ppm)

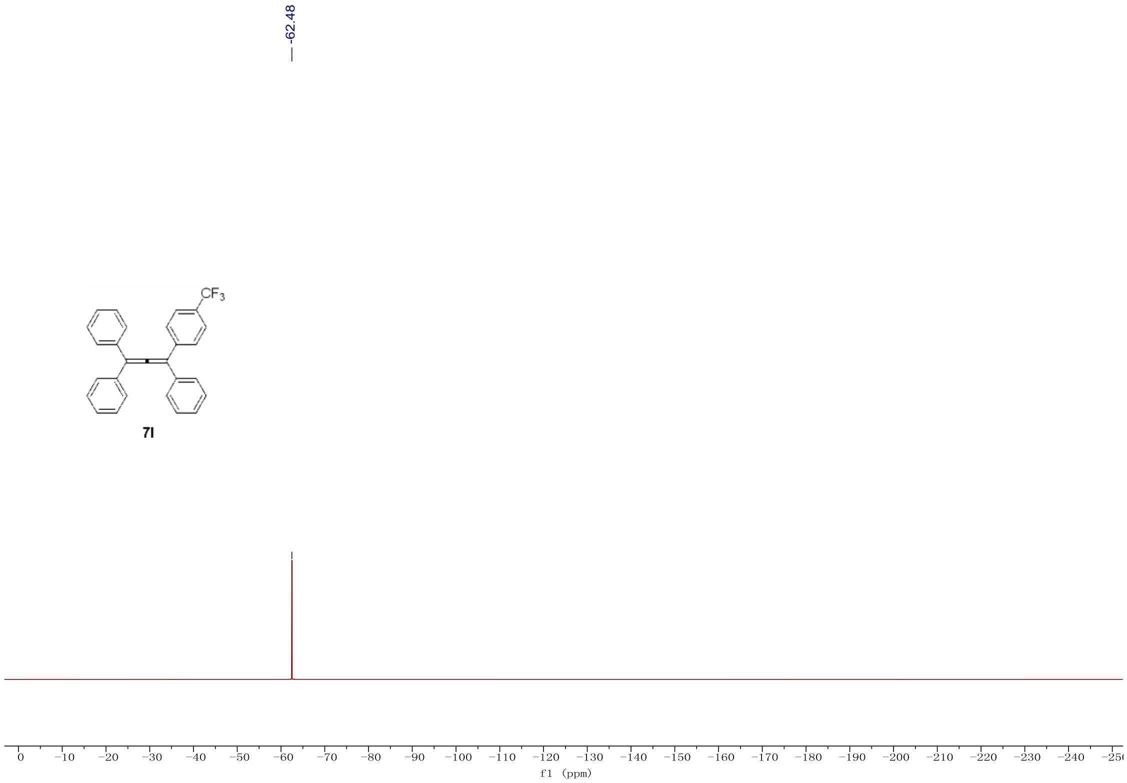
-62.48
CF3
7l
f1 (ppm)

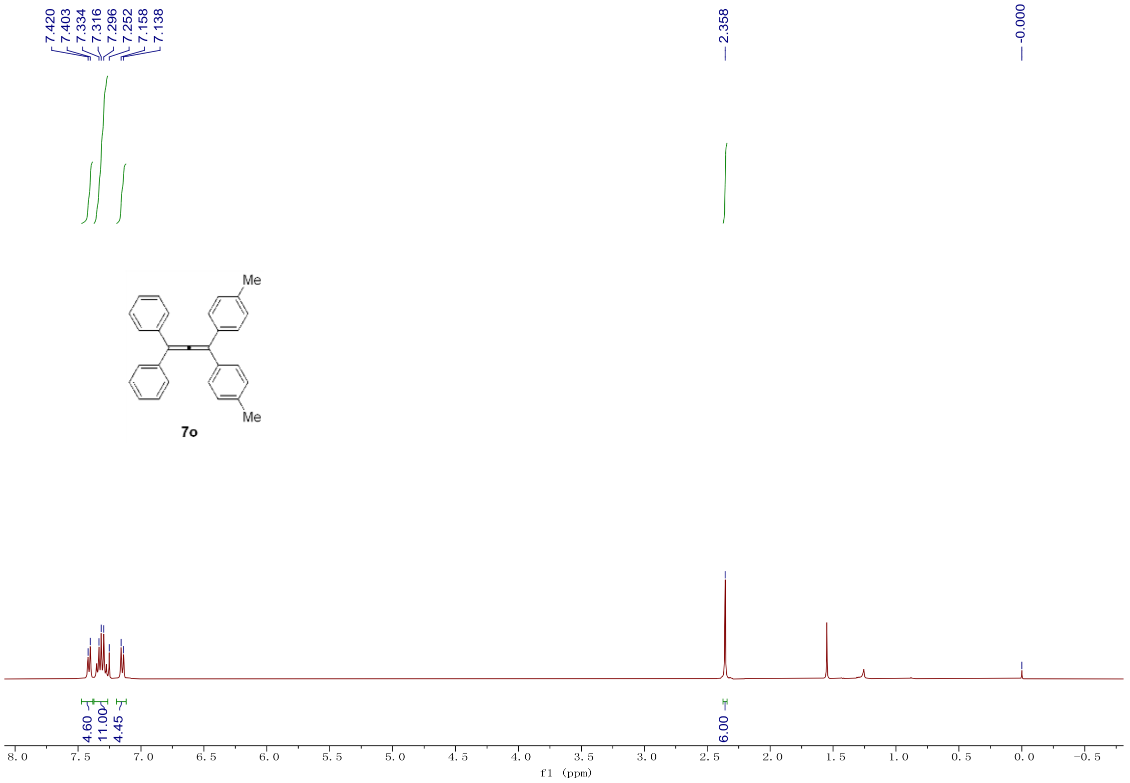
7.420
7.403
7.334
7.316
7.296
7.252
7.158
7.138
2.358
-0.000
Me
Me
7o
4.60
11.00
4.45
6.00
8.0 7.5 7.0 6.5 6.0 5.5 5.0 4.5 4.0 3.5 3.0 2.5 2.0 1.5 1.0 0.5 0.0 -0.5
f1 (ppm)

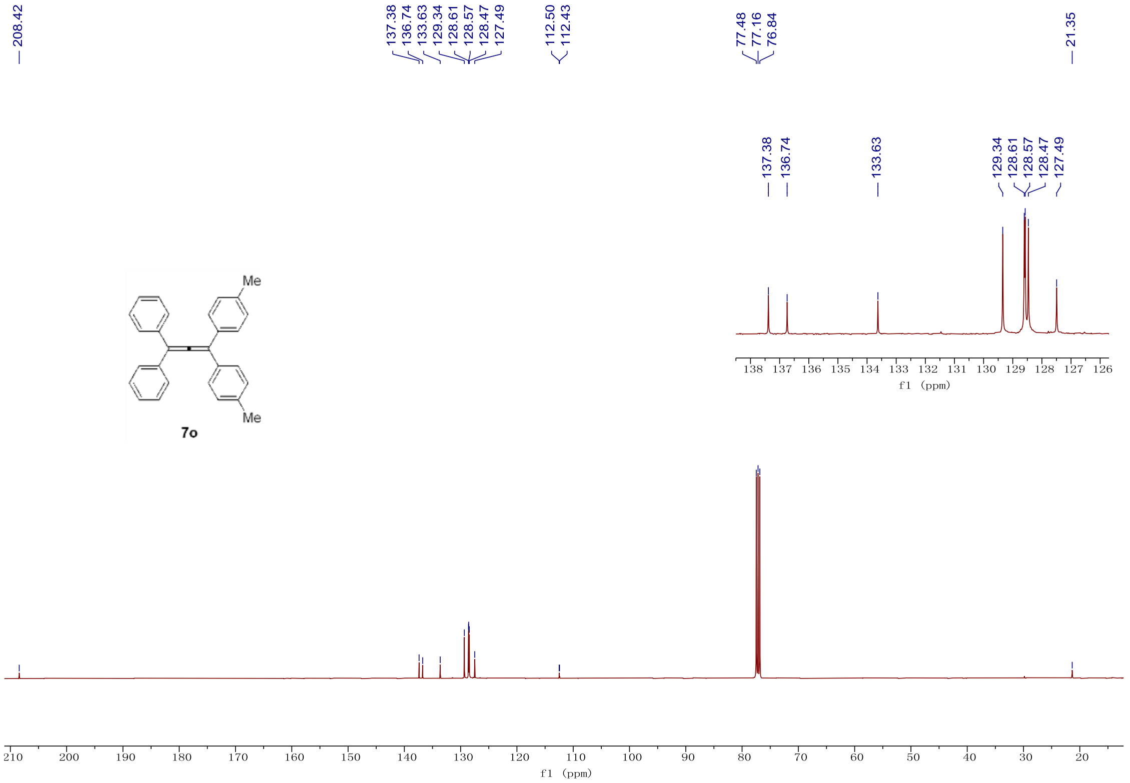
208.42
137.38
136.74
133.63
129.34
128.61
128.57
128.47
127.49
112.50
112.43
77.48
77.16
76.84
21.35
Me
Me
7o
138 137 136 135 134 133 132 131 130 129 128 127 126
f1 (ppm)
210 200 190 180 170 160 150 140 130 120 110 100 90 80 70 60 50 40 30 20
f1 (ppm)

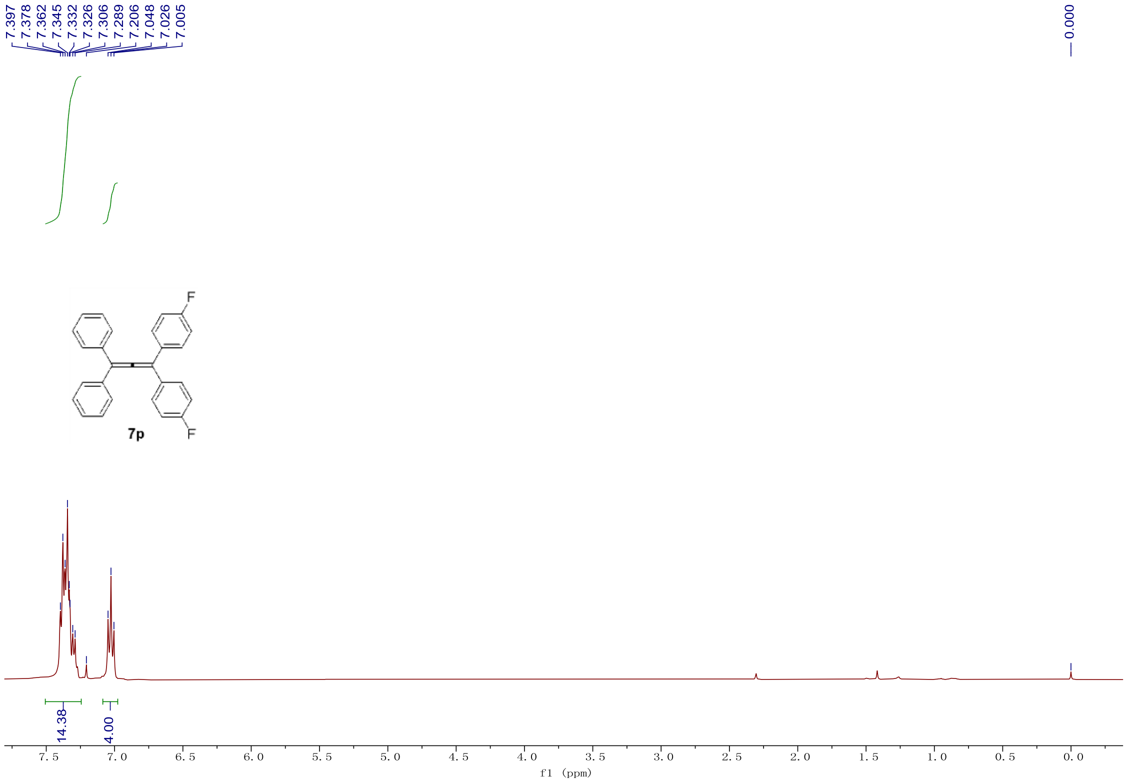
7.397
7.378
7.362
7.345
7.332
7.326
7.306
7.289
7.206
7.048
7.026
7.005
0.000
F
F
7p
14.38
4.00
f1 (ppm)

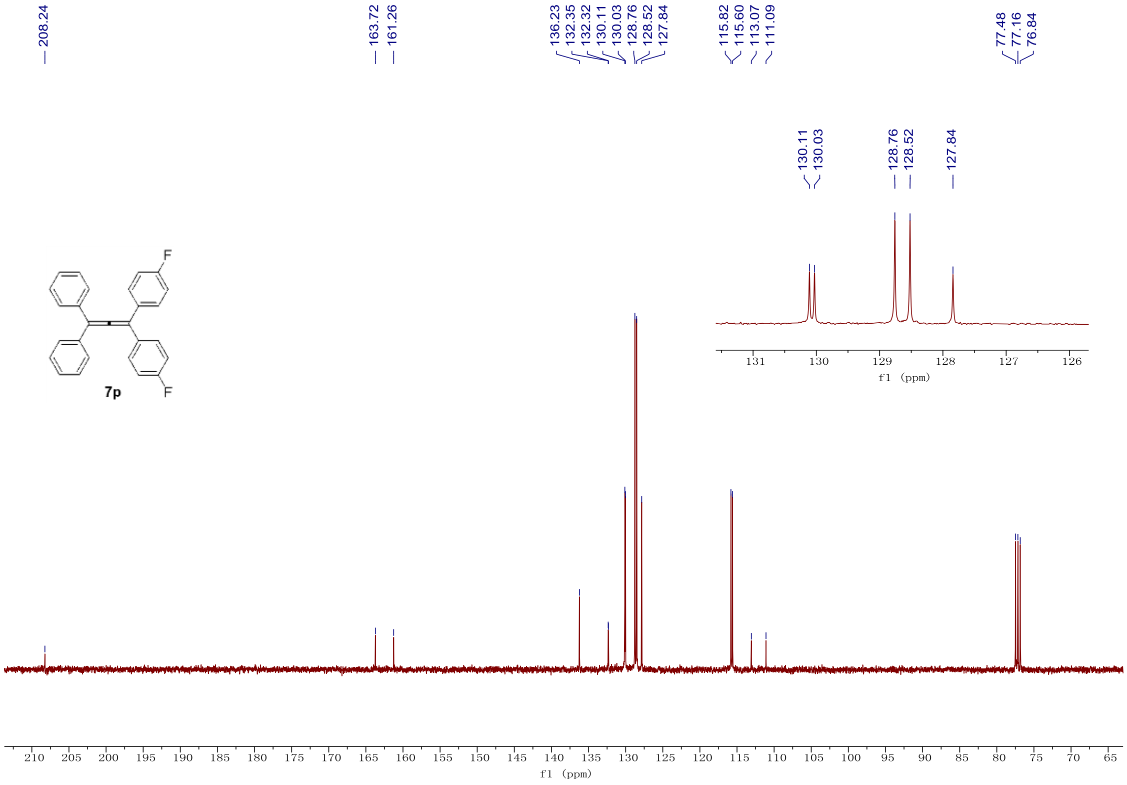
208.24
163.72
161.26
136.23
132.35
132.32
130.11
130.03
128.76
128.52
127.84
115.82
115.60
113.07
111.09
77.48
77.16
76.84
130.11
130.03
128.76
128.52
127.84
f1 (ppm)
F
F
7p
f1 (ppm)

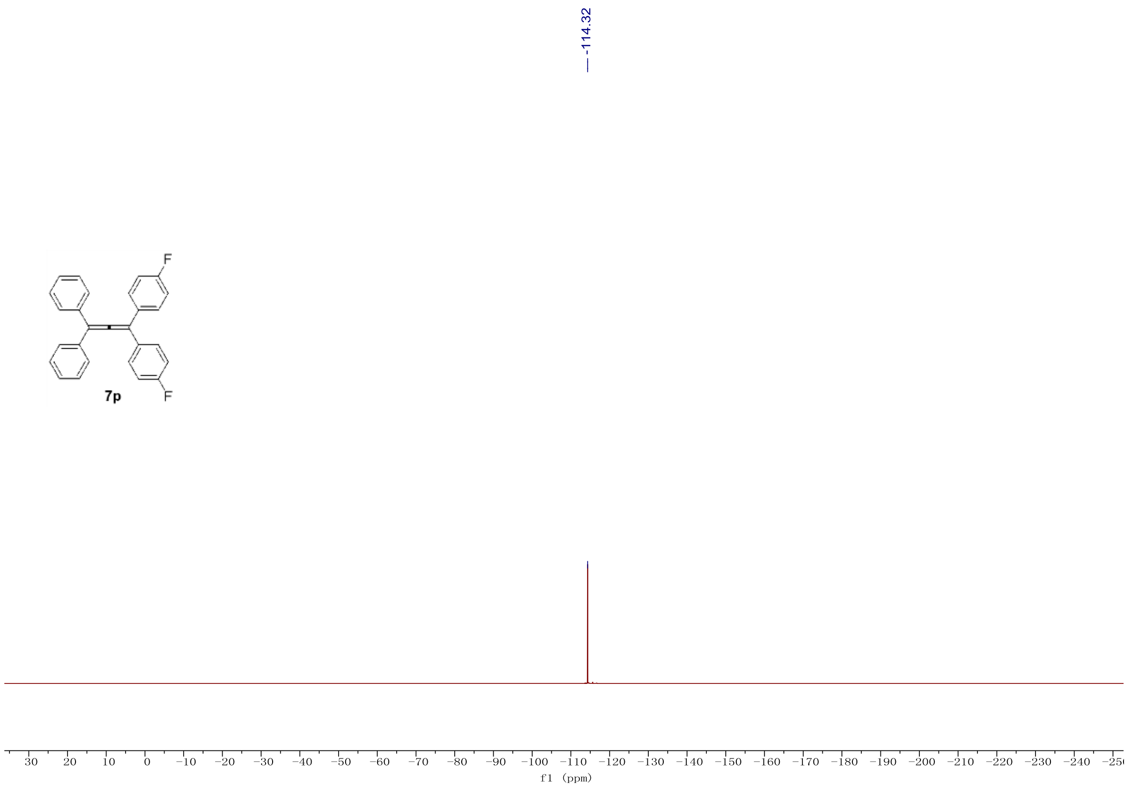
-114.32
F
F
7p
30 20 10 0 −10 −20 −30 −40 −50 −60 −70 −80 −90 −100 −110 −120 −130 −140 −150 −160 −170 −180 −190 −200 −210 −220 −230 −240
f1 (ppm)

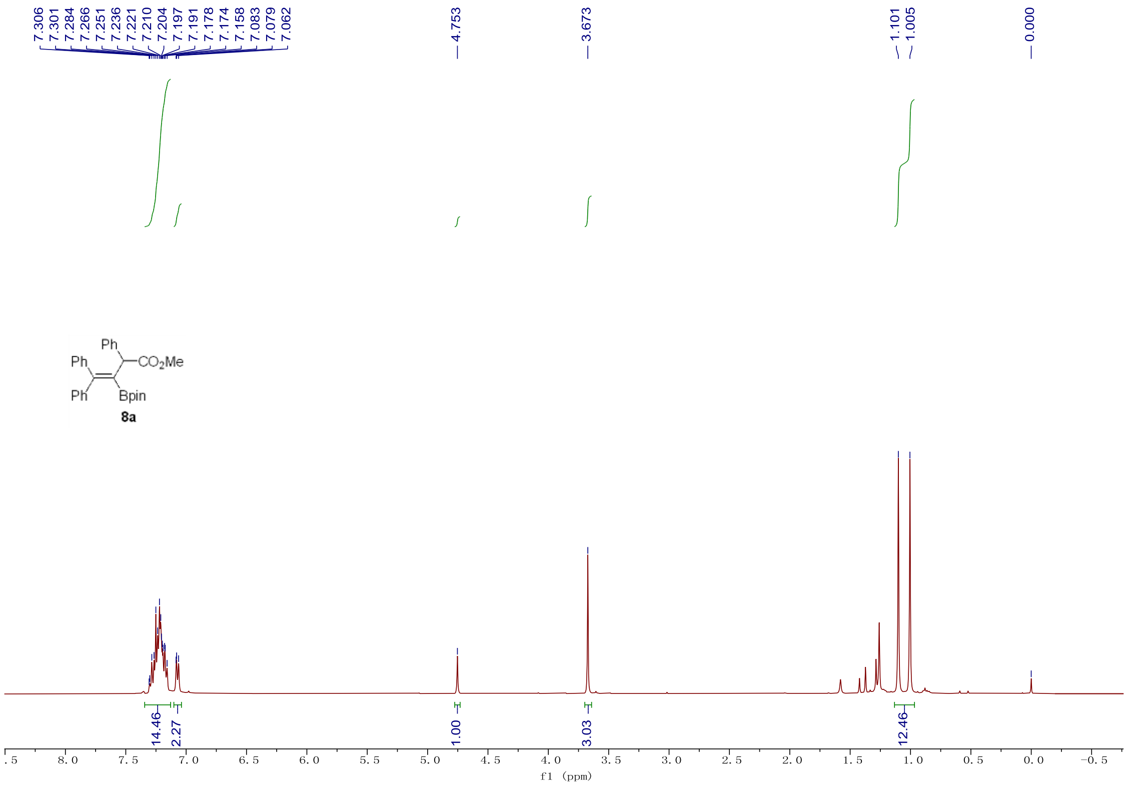
7.306
7.301
7.284
7.266
7.251
7.236
7.221
7.210
7.204
7.197
7.191
7.178
7.174
7.158
7.083
7.079
7.062
4.753
3.673
1.101
1.005
0.000
Ph
Ph
CO2Me
Ph
Bpin
8a
14.46
2.27
1.00
3.03
12.46
8.0 7.5 7.0 6.5 6.0 5.5 5.0 4.5 4.0 3.5 3.0 2.5 2.0 1.5 1.0 0.5 0.0 −0.5
f1 (ppm)

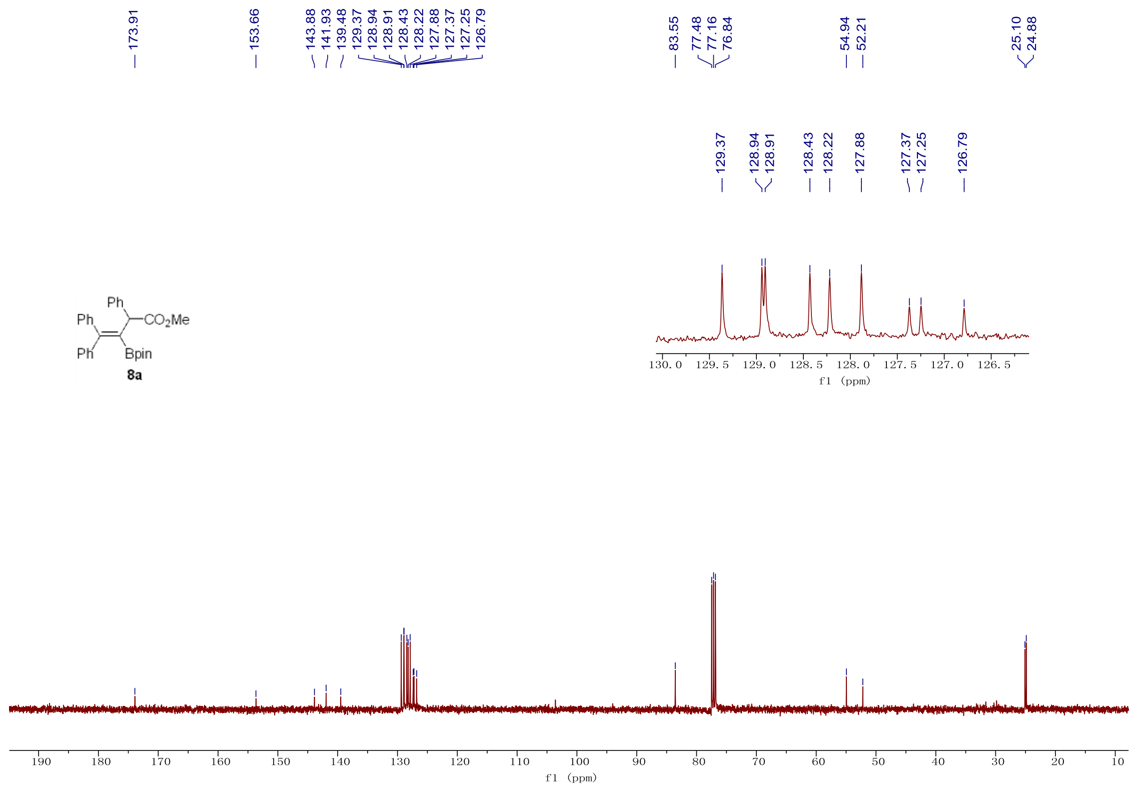

173.91
153.66
143.88
141.93
139.48
129.37
128.94
128.91
128.43
128.22
127.88
127.37
127.25
126.79
83.55
77.48
77.16
76.84
54.94
52.21
25.10
24.88
Ph
Ph
CO2Me
Ph
Bpin
8a
f1 (ppm)


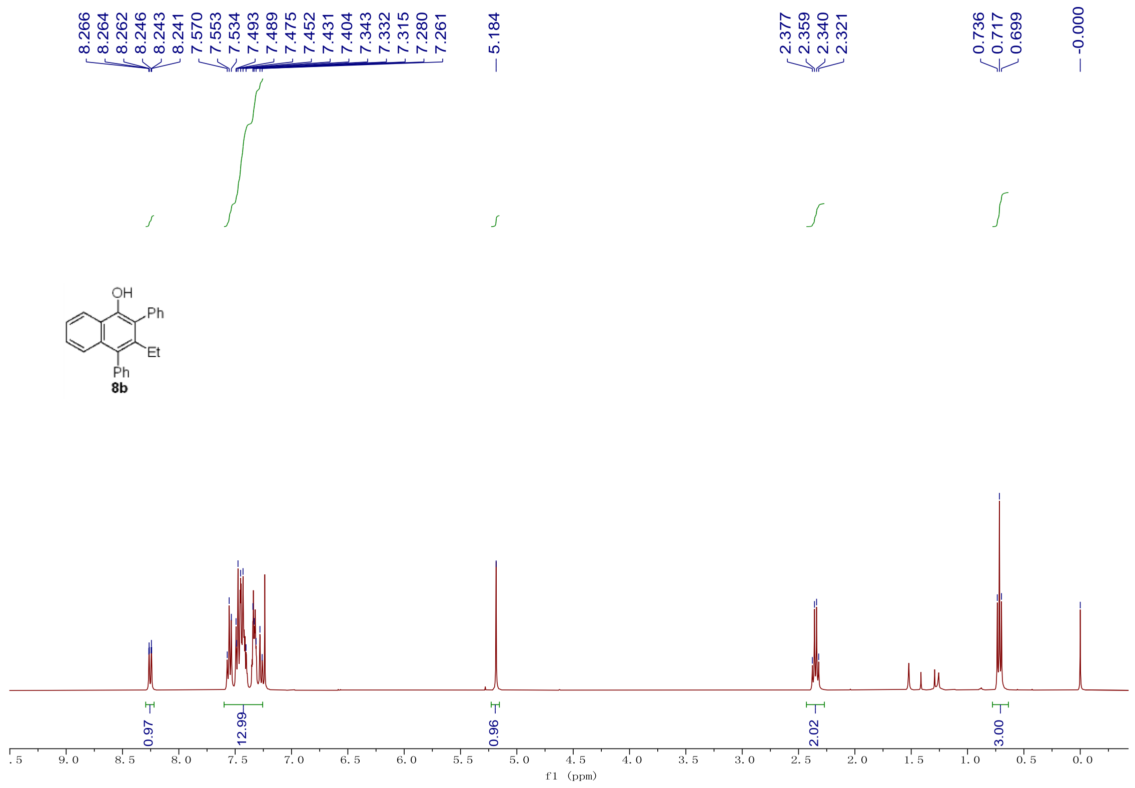

8.266
8.264
8.262
8.246
8.243
8.241
7.570
7.553
7.534
7.493
7.489
7.475
7.452
7.431
7.404
7.343
7.332
7.315
7.280
7.261
5.184
2.377
2.359
2.340
2.321
0.736
0.717
0.699
-0.000
OH
Ph
Et
Ph
8b
0.97
12.99
0.96
2.02
3.00
f1 (ppm)

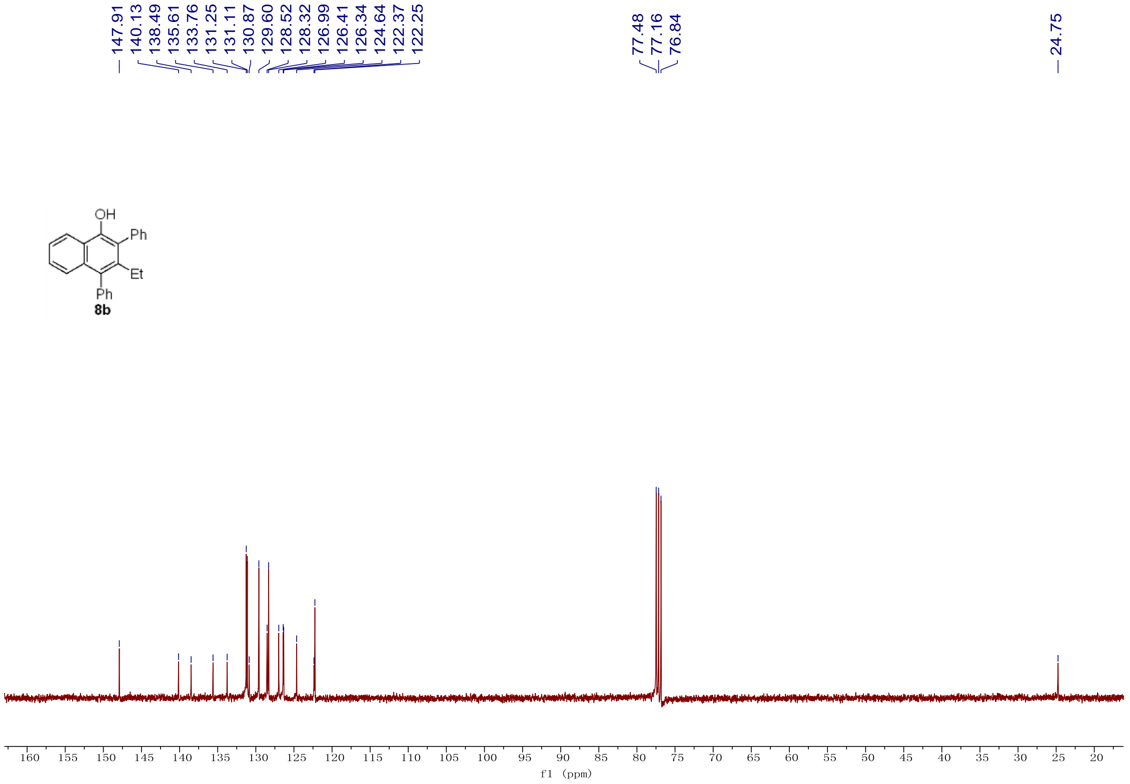
147.91
140.13
138.49
135.61
133.76
131.25
131.11
130.87
129.60
128.52
128.32
126.99
126.41
126.34
124.64
122.37
122.25
77.48
77.16
76.84
24.75
OH
Ph
Et
Ph
8b
160 155 150 145 140 135 130 125 120 115 110 105 100 95 90 85 80 75 70 65 60 55 50 45 40 35 30 25 20
f1 (ppm)

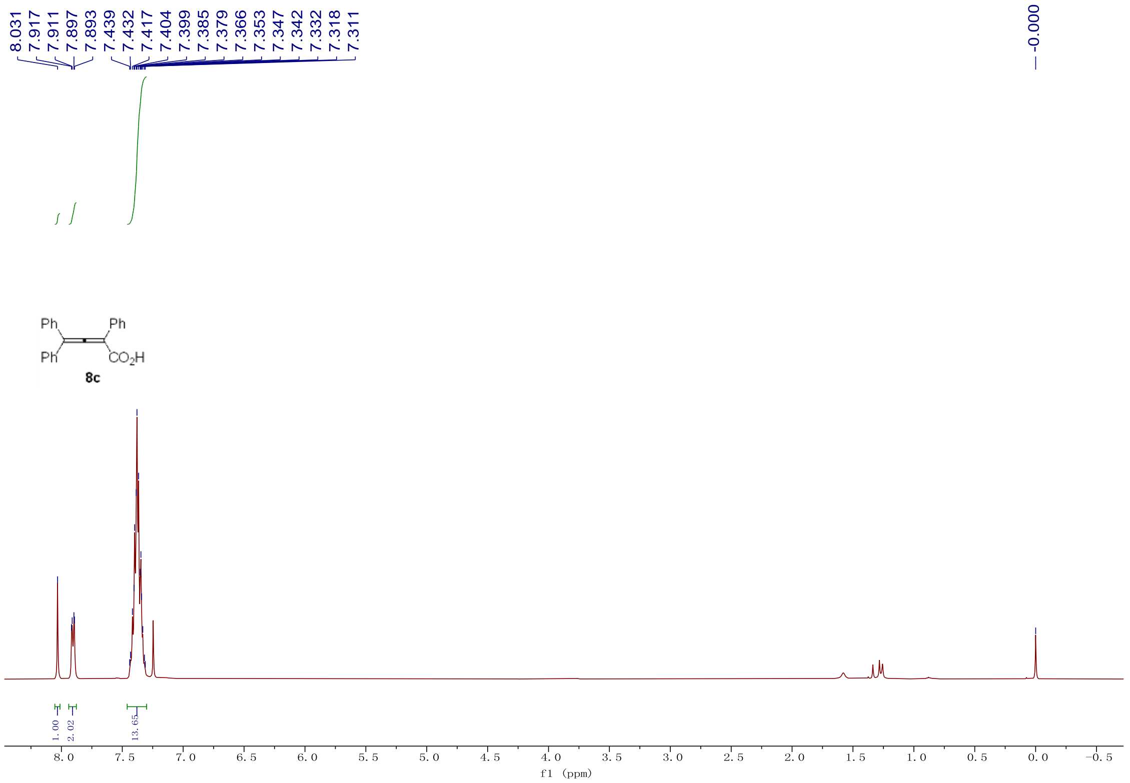
8.031
7.917
7.911
7.897
7.893
7.439
7.432
7.417
7.404
7.399
7.385
7.379
7.366
7.353
7.347
7.342
7.332
7.318
7.311
-0.000
Ph
Ph
Ph
CO2H
8c
1.00
2.02
13.65
8.0 7.5 7.0 6.5 6.0 5.5 5.0 4.5 4.0 3.5 3.0 2.5 2.0 1.5 1.0 0.5 0.0 -0.5
f1 (ppm)

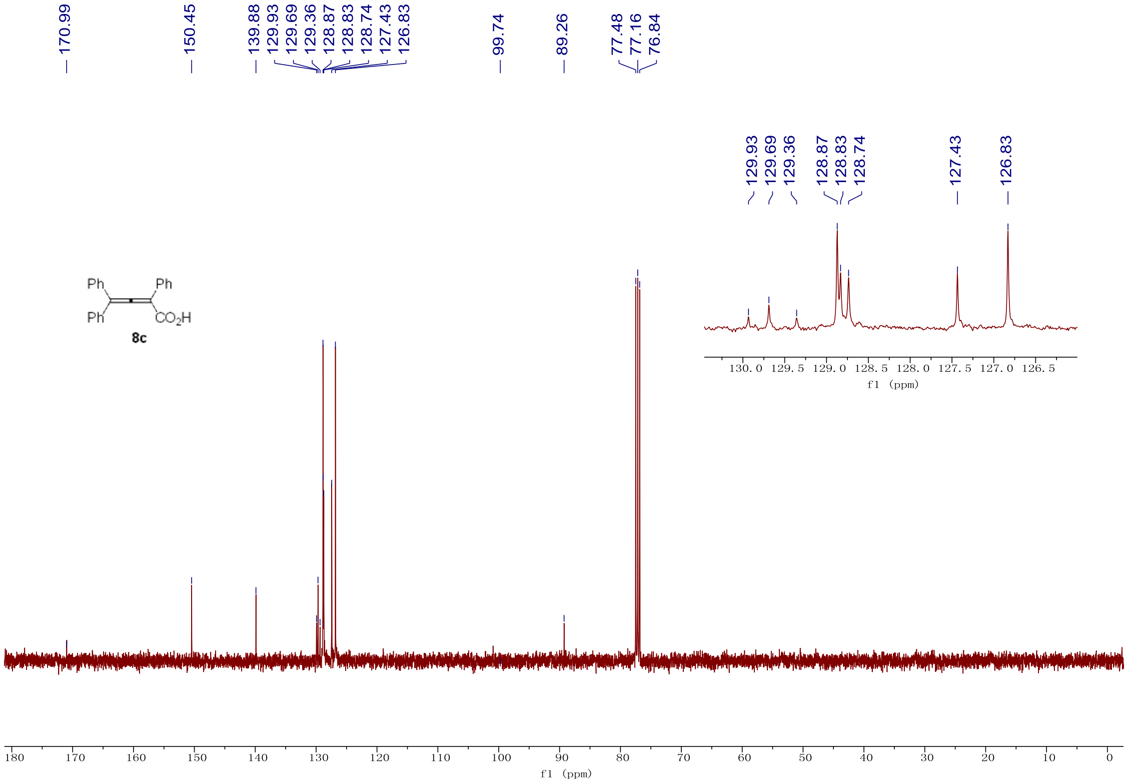
170.99
150.45
139.88
129.93
129.69
129.36
128.87
128.83
128.74
127.43
126.83
99.74
89.26
77.48
77.16
76.84
Ph
Ph
Ph
CO2H
8c
130.0 129.5 129.0 128.5 128.0 127.5 127.0 126.5
f1 (ppm)
180 170 160 150 140 130 120 110 100 90 80 70 60 50 40 30 20 10 0
f1 (ppm)

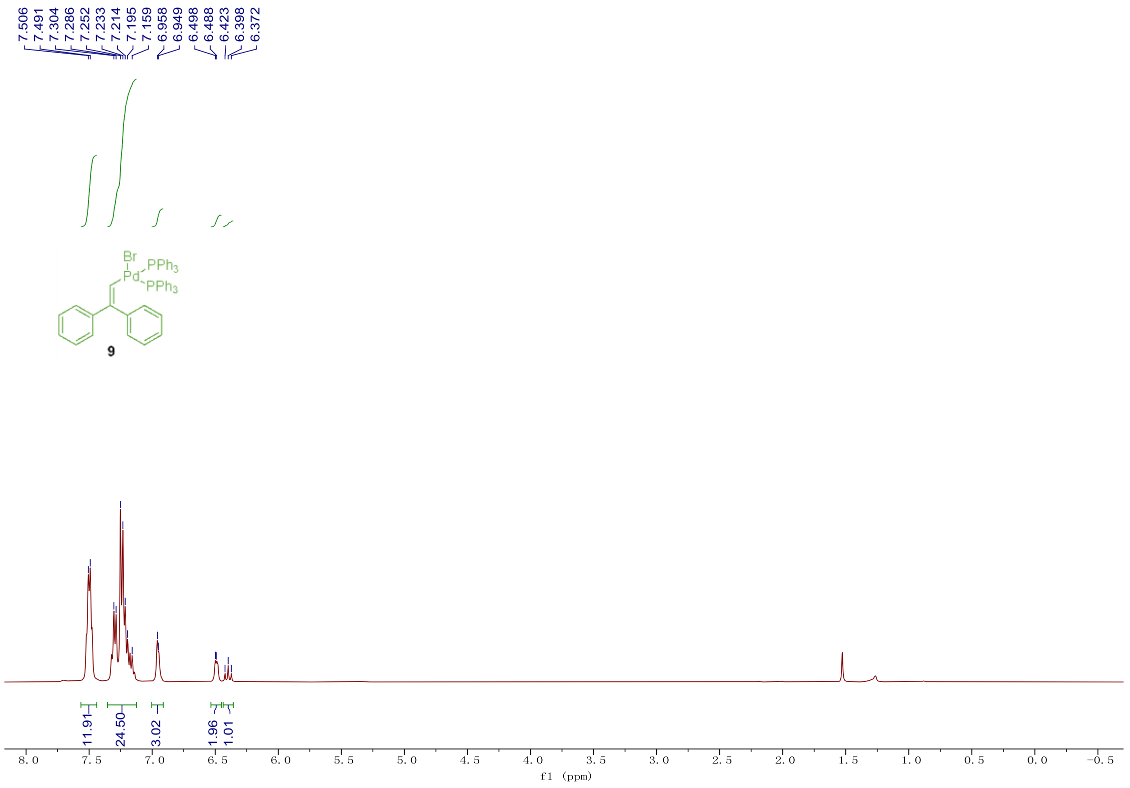
7.506
7.491
7.304
7.286
7.252
7.233
7.214
7.195
7.159
6.958
6.949
6.498
6.488
6.423
6.398
6.372
Br
Pd
PPh3
PPh3
9
11.91
24.50
3.02
1.96
1.01
8.0 7.5 7.0 6.5 6.0 5.5 5.0 4.5 4.0 3.5 3.0 2.5 2.0 1.5 1.0 0.5 0.0 -0.5
f1 (ppm)

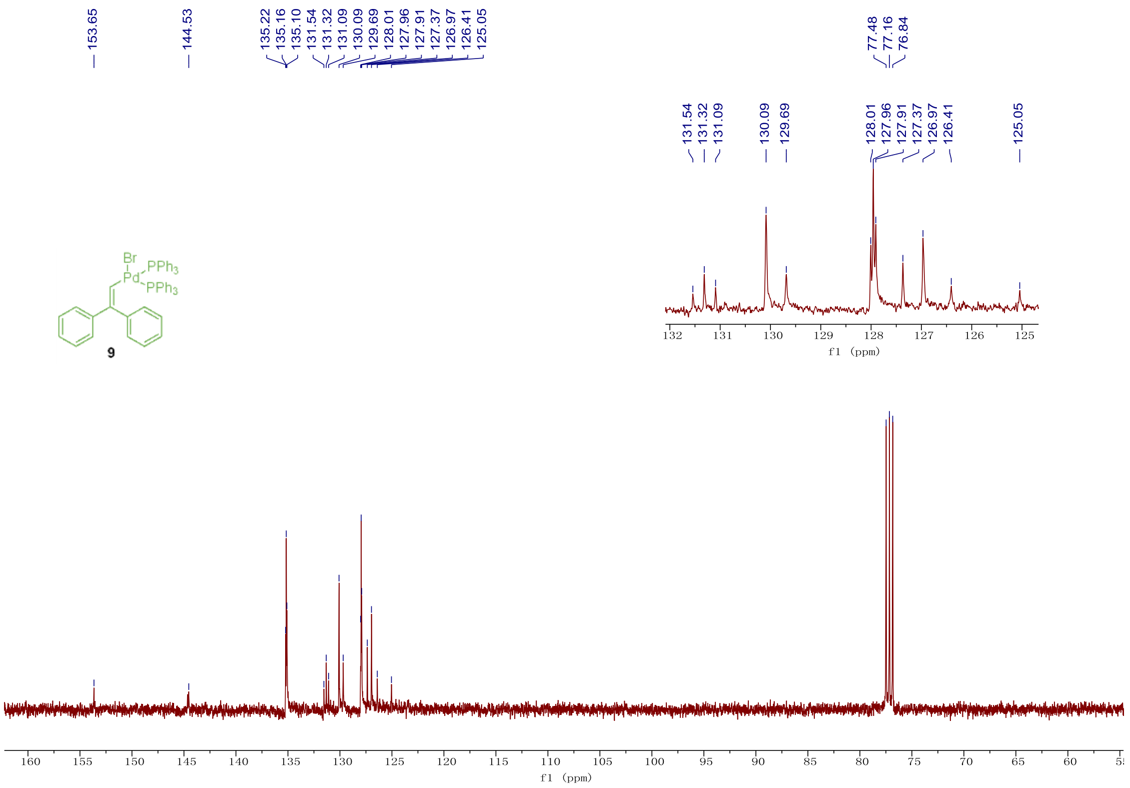
153.65
144.53
135.22
135.16
135.10
131.54
131.32
131.09
130.09
129.69
128.01
127.96
127.91
127.37
126.97
126.41
125.05
77.48
77.16
76.84
Br
PPh3
Pd
PPh3
9
f1 (ppm)

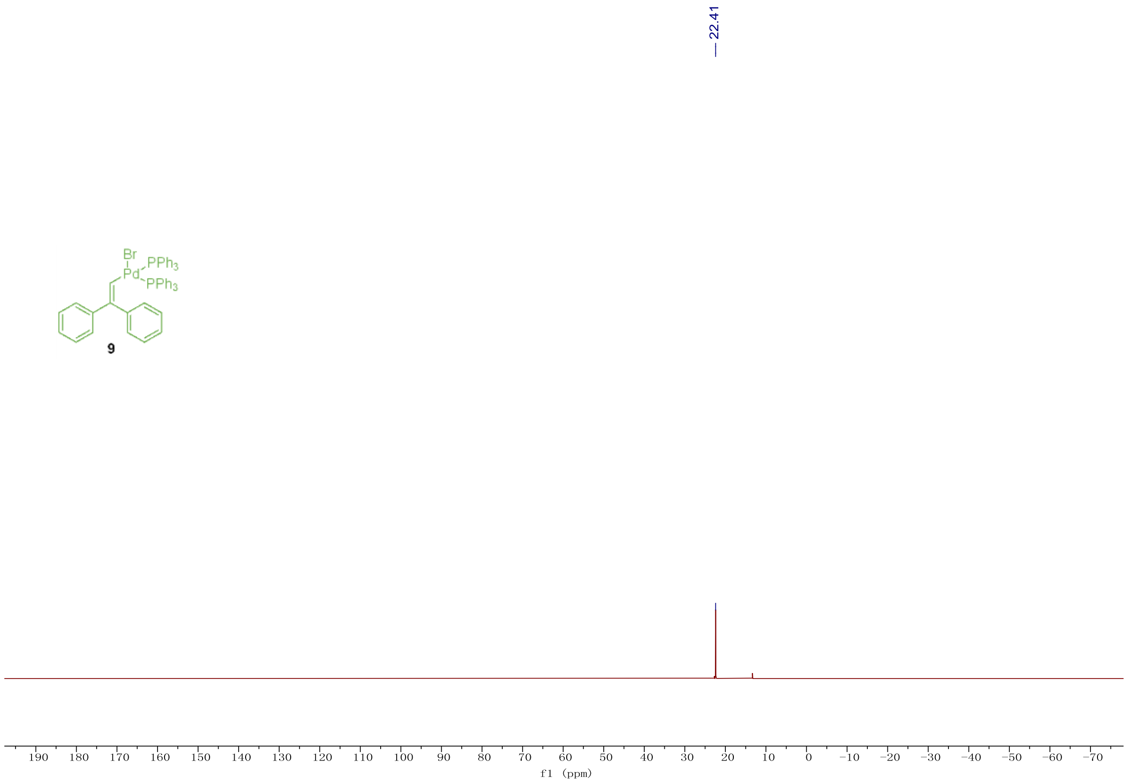
22.41
Br
PPh3
Pd
PPh3
9
f1 (ppm)

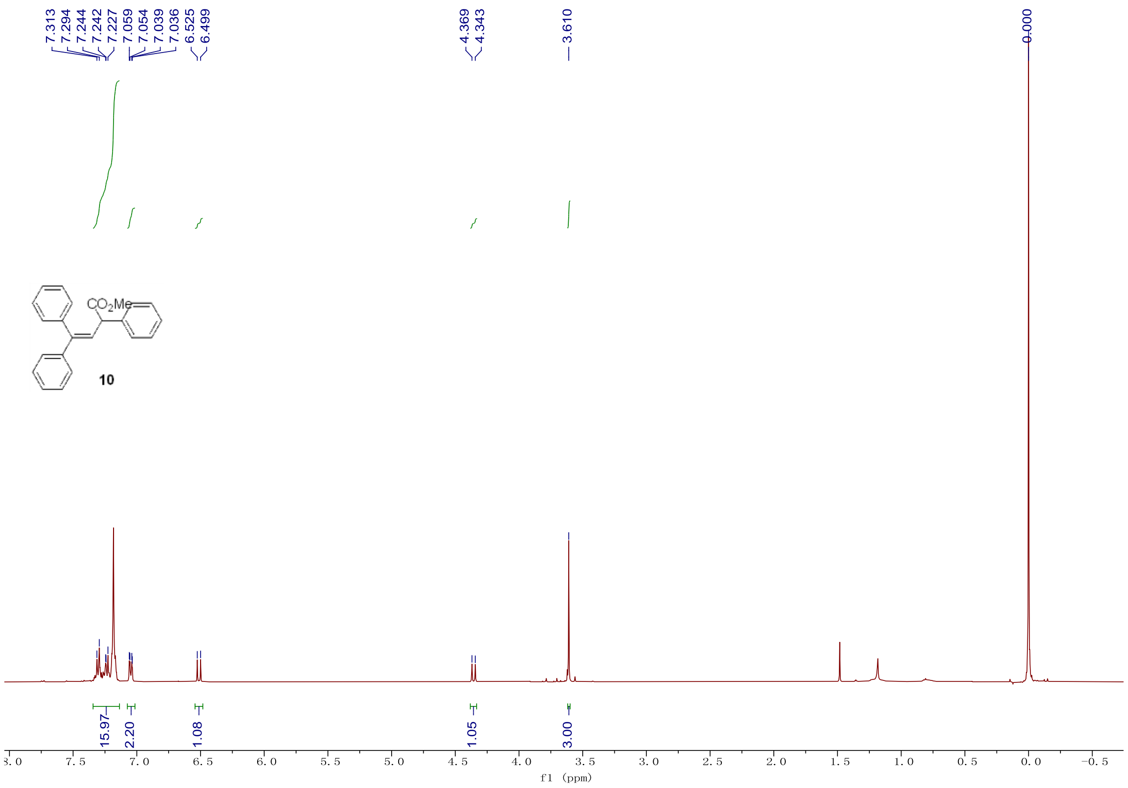
7.313
7.294
7.244
7.242
7.227
7.059
7.054
7.039
7.036
6.525
6.499
4.369
4.343
3.610
0.000
CO2Me
10
15.97
2.20
1.08
1.05
3.00
f1 (ppm)

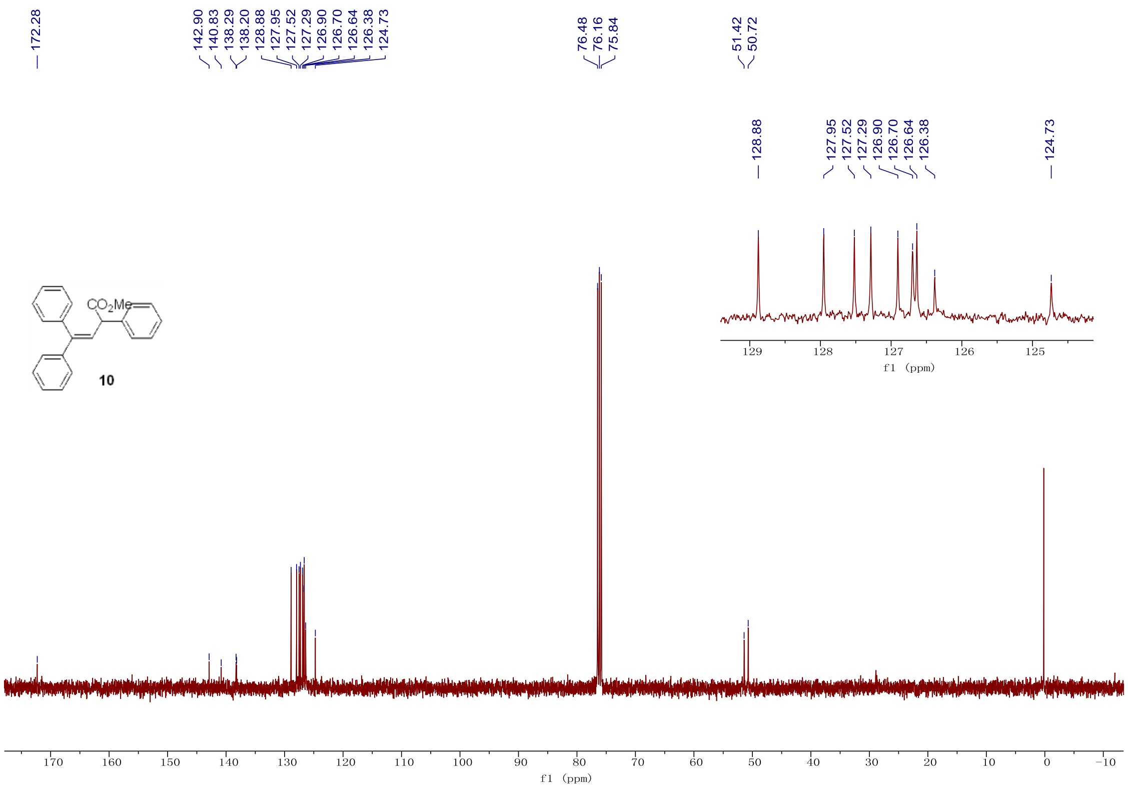
172.28
142.90
140.83
138.29
138.20
128.88
127.95
127.52
127.29
126.90
126.70
126.64
126.38
124.73
76.48
76.16
75.84
51.42
50.72
CO2Me
10
f1 (ppm)

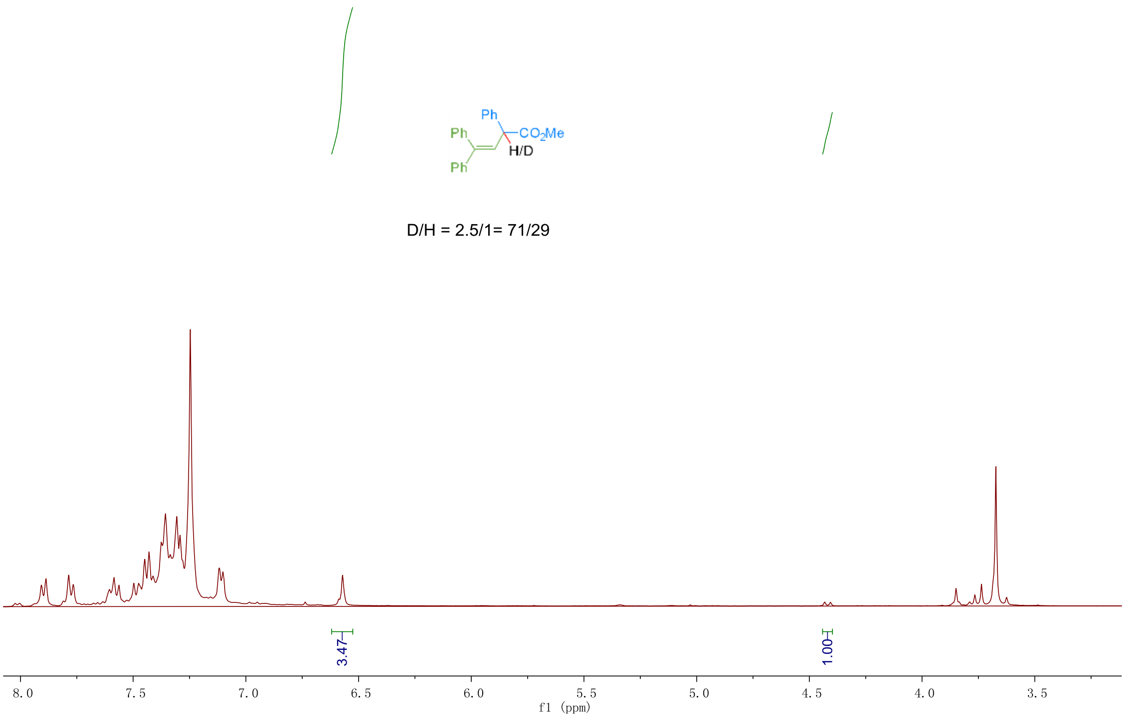
Ph
Ph
Ph
CO2Me
H/D
D/H = 2.5/1= 71/29
3.47
1.00
8.0
7.5
7.0
6.5
6.0
5.5
5.0
4.5
4.0
3.5
f1 (ppm)

# 6. Supplementary References


1 Zhang, G., Bai, R.-X., Li, C.-H., Feng, C.-G., Lin, G.-Q. Halogenation of 1,1-diarylethylenes by *N*-halosuccinimides. *Tetrahedron* **75**, 1658-1662 (2019).

2 Chen, G., Gui, J., Li, L., Liao, J. Chiral sulfoxide-olefin ligands: completely switchable stereoselectivity in rhodium-catalyzed asymmetric conjugate additions. *Angew. Chem. Int. Ed.* **50**, 7681-7685 (2011).

3 Tian, Y., Qi, J., Sun, C., Yin, D., Wang, X., Xiao, Q. One-pot synthesis of methylisoquinolines via a sequential Pd-catalyzed Heck reaction and intramolecular cyclization. *Org. Biomol. Chem*. **11**, 7262-7266 (2013).

4 Pietruszka, J., Scholzel, M. Ene reductase-catalysed synthesis of (*R*)-profen derivatives. *Adv. Synth. Catal.* **354**, 751-756 (2012).

5 Hsieh, J. C., Cheng, A. Y., Fu, J. H.,Kang, T. W. Copper-catalyzed domino coupling reaction: an efficient method to synthesize oxindoles. *Org. Biomol. Chem.* **10**, 6404-6409 (2012).
