# Supplementary material for: Palladium-catalyzed allene synthesis enabled by β-hydrogen elimination from sp2-carbon
Source: Nat Commun. 2021 Feb 1;12:728. doi: 10.1038/s41467-020-20740-w (PMC7851150; doi:10.1038/s41467-020-20740-w)
Supplement: Supplementary file 3 — Supplementary Data 1 [file 41467_2020_20740_MOESM3_ESM.zip › 264485_2_data_set_5139150_qkqvk3 (3).docx]

**Computational details**

**Computational Methods**

The geometries of all species were optimized using B3LYP functional. The 6-31G* basis set was applied for H, C, O, N, P and Br atoms, while LANL2DZ basis set was used for Pd atom (abbreviated as B1). Frequency calculations at B3LYP/B1 level were performed to obtain the Gibbs free energies and to characterize the intermediates and the transition states, which respectively have all real or only one imaginary vibrational frequency. Each transition state was further confirmed to connect the reactant and product by the intrinsic reaction coordinate (IRC) calculation. Solvent effect was considered by performing single point calculations on the optimized gas-phase geometries with SMD model using tetrahydrofuran as solvent. The extended all-electron basis set def2-TZVP (abbreviated as B2 was used to correct the relative energies of the species. The energy data reported in the manuscript have been calculated at M06/B2//B3LYP/B1 level of theory by adding free energy and solvation corrections. All calculations were performed by Gaussian09 software.

**Cartesian coordinates for optimized geometries**

**1a**

C 0.20460900 -0.34409000 -0.04548100

C -0.35595900 -1.56489000 -0.11247500

H 0.23458800 -2.46375000 -0.23220700

C -0.54962200 0.93810900 0.03657300

C -0.21212200 1.88444500 1.01893900

C -1.57580200 1.24702600 -0.86998800

C -0.89953800 3.09381500 1.10889900

H 0.59000200 1.66345600 1.71726500

C -2.25467800 2.46291200 -0.78711800

H -1.83434000 0.53491700 -1.64671000

C -1.92314500 3.38753300 0.20497800

H -0.63293500 3.80863100 1.88284500

H -3.04217100 2.68796900 -1.50141600

H -2.45498900 4.33298000 0.27036200

Br -2.21245500 -1.96609300 0.02081000

C 1.69685500 -0.27067900 -0.06323600

C 2.48280300 -1.19027800 0.65235600

C 2.34928800 0.72283900 -0.81491500

C 3.87563600 -1.13393500 0.59910200

H 1.99614400 -1.93642900 1.27394900

C 3.74083900 0.77717000 -0.86836400

H 1.75764000 1.44760900 -1.36582300

C 4.51025600 -0.15225800 -0.16391100

H 4.46451400 -1.85048800 1.16561400

H 4.22576700 1.54706400 -1.46272700

H 5.59518800 -0.10501100 -0.20165600

**Pd(0)**

P 2.32405500 -0.00192700 0.00297100

P -2.32790500 -0.00154400 0.00044900

C 3.11099800 -0.12437100 -1.66841100

C 4.32582100 -0.78259200 -1.91323100

H 4.85302700 -1.27031900 -1.09870600

C 4.86130200 -0.82532000 -3.20291000

H 5.80106600 -1.34289300 -3.37831700

C 4.19234000 -0.21013800 -4.26247300

H 4.60946300 -0.24657000 -5.26550300

C 2.97903200 0.44247100 -4.03020700

H 2.44659700 0.91415700 -4.85202000

C 2.43862400 0.47766200 -2.74463800

H 1.48079000 0.96081500 -2.56613600

C 3.10372900 1.51291300 0.72800900

C 4.32428800 2.04692400 0.28749400

H 4.85840700 1.57502800 -0.53170200

C 4.85528700 3.19094200 0.88805700

H 5.79937700 3.59649800 0.53307600

C 4.17608900 3.81346700 1.93677600

H 4.58966500 4.70490700 2.40120000

C 2.95749600 3.29301100 2.37927800

H 2.41778400 3.77885200 3.18795200

C 2.42156000 2.15603000 1.77399600

H 1.45995100 1.76447100 2.09739900

C 3.11308300 -1.38593300 0.94554200

C 2.45996600 -2.62962000 0.93322000

H 1.51538400 -2.72597000 0.40307900

C 3.00290000 -3.72501600 1.60473500

H 2.48563000 -4.68073300 1.58448800

C 4.19921900 -3.58946200 2.31339000

H 4.61814600 -4.43980200 2.84523200

C 4.84886200 -2.35445300 2.34490500

H 5.77533900 -2.23990800 2.90195100

C 4.31160700 -1.25892100 1.66436800

H 4.82432800 -0.30241800 1.70122600

C -3.11397500 -0.50311300 -1.59849700

C -2.44699600 -1.46915000 -2.37013000

H -1.49450700 -1.86041200 -2.02028700

C -2.98554000 -1.91014300 -3.57891100

H -2.45721500 -2.65889600 -4.16338100

C -4.19137400 -1.37934900 -4.04351400

H -4.60710100 -1.71485000 -4.99006200

C -4.85493100 -0.40759500 -3.29244900

H -5.78898800 0.01611400 -3.65269500

C -4.32163500 0.02794700 -2.07678900

H -4.84494900 0.78887400 -1.50539400

C -3.10799700 1.63651900 0.36661700

C -2.44235600 2.78489600 -0.09335400

H -1.49539800 2.67330900 -0.61623400

C -2.97579700 4.05403700 0.13075600

H -2.44894900 4.93255300 -0.23264600

C -4.17489100 4.19559200 0.83356800

H -4.58624400 5.18464500 1.01790700

C -4.83722500 3.06214500 1.30808800

H -5.76596400 3.16593600 1.86339100

C -4.30942000 1.78967900 1.07516800

H -4.83148900 0.91620000 1.45420800

C -3.11264400 -1.13676700 1.23415400

C -2.43008400 -1.34906600 2.44313300

H -1.46696700 -0.86808800 2.59716100

C -2.96734800 -2.17907000 3.42734400

H -2.42674800 -2.33236800 4.35772100

C -4.18823500 -2.82241000 3.21078900

H -4.60317600 -3.47702000 3.97282300

C -4.86813700 -2.63045700 2.00666800

H -5.81386900 -3.13583800 1.82804200

C -4.33557500 -1.79240900 1.02406400

H -4.87027500 -1.65777600 0.08847200

Pd -0.00181800 -0.00173300 0.00171300

**2a**

N 0.84912500 1.67297500 -0.00029600

C 0.50061100 0.40180300 -0.00017900

C 1.62140500 -0.55111000 -0.00008700

O 1.51745300 -1.76314200 0.00014600

O 2.81573200 0.09401100 0.00001900

C -0.94297100 0.07874400 -0.00004700

C -1.90466000 1.10724600 0.00029500

C -1.38764400 -1.25616700 -0.00028100

C -3.26524200 0.81158800 0.00038600

H -1.59517400 2.14883600 0.00050400

C -2.75324300 -1.53949400 -0.00017800

H -0.66244400 -2.05861600 -0.00052500

C -3.70037400 -0.51507700 0.00015000

H -3.98601600 1.62488700 0.00065200

H -3.07464100 -2.57778300 -0.00036400

H -4.76203400 -0.74529600 0.00022300

N 1.12144300 2.77822300 -0.00028200

C 3.97075800 -0.75870500 0.00019300

H 3.98043800 -1.39396400 -0.88942700

H 4.82726500 -0.08401500 -0.00080200

H 3.98132200 -1.39236200 0.89095900

**L**

P 0.00020700 0.00009100 1.20650000

C 1.11548400 1.24337100 0.40248500

C 2.34870900 1.48966900 1.03025800

C 0.79731500 1.96871400 -0.75633400

C 3.24771800 2.41756200 0.50413600

H 2.60273800 0.95262100 1.94140000

C 1.69255200 2.90627700 -1.27708400

H -0.15472500 1.80458400 -1.25201300

C 2.92011200 3.13069200 -0.65137000

H 4.19819500 2.59157200 1.00195200

H 1.42900900 3.46129600 -2.17399800

H 3.61468400 3.86140800 -1.05745000

C 0.51961300 -1.58673000 0.40232900

C 0.12858400 -2.77797500 1.03764000

C 1.29383400 -1.67291200 -0.76511700

C 0.48234800 -4.02025900 0.51053800

H -0.45357100 -2.72949600 1.95511500

C 1.65795500 -2.91651500 -1.28697100

H 1.61696100 -0.76544600 -1.26622000

C 1.25095300 -4.09223000 -0.65351500

H 0.16752200 -4.93072000 1.01397300

H 2.26038400 -2.96540500 -2.19065000

H 1.53642300 -5.05874100 -1.06052900

C -1.63438200 0.34364300 0.40300300

C -2.46645400 1.28496000 1.03344000

C -2.10160000 -0.29208500 -0.75781700

C -3.71994900 1.59876400 0.50755700

H -2.12971900 1.77138500 1.94618500

C -3.36129700 0.01367600 -1.27842100

H -1.48183800 -1.03213700 -1.25537400

C -4.17164900 0.96119600 -0.65035300

H -4.34767200 2.33218000 1.00712900

H -3.70866600 -0.49009100 -2.17701700

H -5.15191500 1.19671800 -1.05641200

**N_2_**

N 0.00000000 0.00000000 0.55265200

N 0.00000000 0.00000000 -0.55265200

**OAc^-^**

C -0.22041500 0.00175300 0.00012100

O -0.80849600 -1.10877200 -0.00005200

O -0.69643400 1.16608300 -0.00008500

C 1.35447500 -0.05484400 0.00003800

H 1.75064000 0.47286700 -0.88022100

H 1.75066400 0.47209700 0.88075300

H 1.73376600 -1.08490900 -0.00039000

**Br^-^**

Br 0.00000000 0.00000000 0.00000000

**HOAc**

H 1.72398900 -0.80302000 -0.00004000

C 0.09238700 0.12562700 0.00004400

O 0.64580900 1.20192700 -0.00001000

O 0.77886500 -1.04651000 -0.00001500

C -1.39779500 -0.10982800 -0.00000100

H -1.68543900 -0.69171200 -0.88196200

H -1.68549900 -0.69160900 0.88201000

H -1.91800000 0.84822300 -0.00006800

**Int1**

P -2.32647400 -0.40993700 -0.05029200

P 0.99105200 1.82452300 0.08456600

C -3.17339100 -0.14126400 1.57126300

C -4.50625600 -0.51534000 1.81367000

H -5.07997000 -1.00851700 1.03445600

C -5.09643700 -0.27401100 3.05498600

H -6.12793700 -0.57009700 3.22780200

C -4.36303500 0.33928600 4.07403800

H -4.82319000 0.52286700 5.04141500

C -3.03405900 0.70100600 3.84979400

H -2.45057400 1.16196900 4.64227000

C -2.44159500 0.45628700 2.60891600

H -1.39728000 0.70908000 2.44836300

C -3.07315600 0.84522600 -1.18863500

C -4.30298000 1.48060100 -0.96186000

H -4.86474800 1.27404200 -0.05658300

C -4.81315800 2.39005800 -1.89021400

H -5.76558300 2.87737100 -1.69705300

C -4.10723200 2.67219800 -3.06108900

H -4.50690300 3.37985000 -3.78269600

C -2.88082800 2.04773700 -3.29612700

H -2.31847600 2.26948500 -4.19916800

C -2.36382600 1.14906100 -2.36219300

H -1.39802600 0.68124000 -2.53726100

C -3.11776000 -1.97898000 -0.64733100

C -3.03216600 -3.12879900 0.15962500

H -2.55293700 -3.07407300 1.13261000

C -3.55165600 -4.34705700 -0.27792100

H -3.47749100 -5.22141700 0.36344900

C -4.15848100 -4.44514900 -1.53263700

H -4.56036300 -5.39544100 -1.87385600

C -4.24133400 -3.31396800 -2.34453200

H -4.71295200 -3.37686800 -3.32198800

C -3.72723100 -2.09030100 -1.90696800

H -3.81063800 -1.22054800 -2.55027700

C -0.00919800 3.30155000 -0.43348300

C -1.20359100 3.58315100 0.25197800

H -1.51401200 2.95581300 1.08303900

C -1.99755700 4.66835600 -0.11384100

H -2.91489600 4.87096500 0.43232800

C -1.62110600 5.48610600 -1.18276200

H -2.24248800 6.33005200 -1.47058400

C -0.44447500 5.20996200 -1.87780000

H -0.14077500 5.84002200 -2.71007300

C 0.35805300 4.12800000 -1.50493900

H 1.27626800 3.93465000 -2.04942600

C 2.57660700 2.10875300 -0.83427500

C 2.73665700 1.46079300 -2.07036400

H 1.96209600 0.79085200 -2.43238800

C 3.89462800 1.64649100 -2.82752100

H 4.00000200 1.12999900 -3.77734200

C 4.91805100 2.46803900 -2.35150100

H 5.82526300 2.60414000 -2.93439500

C 4.77657400 3.10445300 -1.11639900

H 5.57318700 3.73840600 -0.73525300

C 3.61320200 2.93088600 -0.36433400

H 3.51934800 3.43067200 0.59454200

C 1.40081300 2.25497400 1.84104300

C 1.61827300 1.20419400 2.74760200

H 1.53021300 0.17565100 2.40753900

C 1.93842200 1.46932500 4.08104200

H 2.10411600 0.64282900 4.76680700

C 2.03837900 2.78731900 4.52949700

H 2.28308200 2.99406700 5.56817100

C 1.81506500 3.84071200 3.63950300

H 1.88428400 4.86958500 3.98348600

C 1.49690200 3.57773900 2.30609300

H 1.31002500 4.40507100 1.62812700

Pd 0.09694700 -0.43554500 -0.01297700

C 0.42775900 -2.53868200 0.15611900

C 1.67894300 -2.13971600 -0.36116500

H -0.29455900 -3.10187400 -0.42336100

Br 0.23722500 -3.04867500 2.03643100

C 2.93275900 -2.20088900 0.47137500

C 3.37798300 -3.45304600 0.93006300

C 3.72963300 -1.08043600 0.74016600

C 4.56348700 -3.57486000 1.65480400

H 2.78542400 -4.33755400 0.71591500

C 4.91859600 -1.19879700 1.46347200

H 3.41805800 -0.11036400 0.37189500

C 5.33843600 -2.44577600 1.92846000

H 4.88193500 -4.55396100 2.00393200

H 5.51429300 -0.31127900 1.66141500

H 6.26191600 -2.53852400 2.49435800

C 1.86456300 -2.18324700 -1.84996900

C 3.15482700 -2.18253000 -2.41589700

C 0.77179900 -2.24411700 -2.74583600

C 3.34454700 -2.23463000 -3.79820400

H 4.02175800 -2.14484900 -1.76688600

C 0.96465200 -2.29997100 -4.12354700

H -0.24413000 -2.23164000 -2.36507900

C 2.25389900 -2.29279300 -4.66491200

H 4.35743500 -2.23631000 -4.19407700

H 0.09843800 -2.34822900 -4.77921700

H 2.40167300 -2.33464100 -5.74071300

**TS1**

P 0.85746700 1.93974100 0.05402600

P -2.45731200 -0.48895200 0.07286300

C -0.33965000 3.35334600 0.04057100

C -0.34301500 4.34194500 -0.95592400

H 0.41603800 4.33683300 -1.73096300

C -1.31658100 5.34501600 -0.95950000

H -1.30038600 6.10293700 -1.73865800

C -2.29597000 5.38134500 0.03317200

H -3.05102100 6.16294900 0.02920600

C -2.29951200 4.40432400 1.03232100

H -3.05861900 4.41679800 1.80956100

C -1.33586700 3.39703600 1.03300100

H -1.35830300 2.64151300 1.81216100

C 1.71528300 2.14983800 1.67888000

C 1.76761100 3.37075300 2.37196300

H 1.26986800 4.24649200 1.96729500

C 2.45119600 3.46741100 3.58537300

H 2.48008400 4.41799400 4.11192000

C 3.09721900 2.35008500 4.11935600

H 3.63057000 2.42821700 5.06319000

C 3.05447500 1.13348700 3.43607500

H 3.55968000 0.25977700 3.83827100

C 2.36278700 1.03148700 2.22809700

H 2.32967000 0.08120700 1.70455500

C 2.12937100 2.47947600 -1.17985600

C 1.91908100 2.13974000 -2.52809300

H 1.04201100 1.55929800 -2.80495500

C 2.83288300 2.52376200 -3.51109200

H 2.65283200 2.25311200 -4.54808400

C 3.98022000 3.23794500 -3.15883000

H 4.69774700 3.52906700 -3.92144400

C 4.20601600 3.56760400 -1.82106700

H 5.10039600 4.11699200 -1.53869000

C 3.28752300 3.19398400 -0.83787900

H 3.47761500 3.45665800 0.19772400

C -3.59079100 0.29729300 -1.16133900

C -3.16852700 1.48296800 -1.78438200

H -2.19772100 1.90209700 -1.53966400

C -3.98482100 2.12674200 -2.71500200

H -3.64297100 3.04614300 -3.18269200

C -5.22910500 1.58782300 -3.04803900

H -5.86144900 2.08424700 -3.77955600

C -5.65467200 0.40382300 -2.44276100

H -6.61931900 -0.02522000 -2.70157200

C -4.84289500 -0.23762500 -1.50476900

H -5.18348600 -1.15972100 -1.04424000

C -3.01097200 0.22341300 1.68955600

C -2.14383300 0.09893300 2.78835500

H -1.18026500 -0.38768700 2.65798700

C -2.49650400 0.61151900 4.03739600

H -1.81343000 0.50563200 4.87595100

C -3.71701600 1.27160700 4.20373600

H -3.98904400 1.67957800 5.17364100

C -4.58230400 1.40930500 3.11684700

H -5.53234100 1.92363100 3.23823800

C -4.23406200 0.88680500 1.86832800

H -4.91488100 1.00213600 1.03068000

C -3.10980200 -2.22203400 0.16001200

C -4.02594000 -2.65689600 1.13068600

H -4.38050500 -1.96877100 1.89148800

C -4.49006200 -3.97514800 1.12742400

H -5.19690900 -4.29737400 1.88792600

C -4.05181600 -4.87227000 0.15203300

H -4.41480800 -5.89677000 0.14996200

C -3.13676000 -4.44941300 -0.81515600

H -2.77766400 -5.14464800 -1.56905300

C -2.66177900 -3.13814000 -0.80680700

H -1.92443600 -2.82885300 -1.54181400

Pd -0.03744300 -0.25789900 -0.44897900

C 1.88126700 -1.07055700 -1.08418200

C 2.53487600 -1.98829200 -0.29166400

H 2.44726400 -0.27478500 -1.55208800

Br 0.57031000 -1.77517400 -2.59269300

C 1.91268700 -3.22352000 0.23790500

C 2.59265900 -4.45315300 0.11026500

C 0.64738600 -3.24018600 0.85232400

C 2.02133800 -5.64274700 0.55334300

H 3.57222800 -4.46830600 -0.35871900

C 0.06950800 -4.43341500 1.29016100

H 0.12595200 -2.29680800 0.99971600

C 0.75362400 -5.63990900 1.14417200

H 2.56405000 -6.57699200 0.43169300

H -0.91363900 -4.41375900 1.75234100

H 0.30921700 -6.56844600 1.49325700

C 3.95921000 -1.72616900 0.03993900

C 4.47865700 -2.09347100 1.30063100

C 4.84121200 -1.10203700 -0.86731400

C 5.80574900 -1.83738000 1.63948700

H 3.82680100 -2.58568100 2.01629400

C 6.16629600 -0.84180700 -0.52436000

H 4.49110300 -0.84325000 -1.86211500

C 6.65854600 -1.20460300 0.73164400

H 6.17490300 -2.13194300 2.61902000

H 6.82203700 -0.36617300 -1.24949200

H 7.69381200 -1.00577500 0.99545200

**Int2**

P -2.38382700 -0.69901000 -0.16063400

P 0.52832800 1.67474500 0.00598000

C -3.31656300 0.58950900 0.80755100

C -3.63156500 0.41340700 2.16316900

H -3.35457600 -0.50391300 2.67097300

C -4.31808200 1.40258700 2.87110800

H -4.55777200 1.23998300 3.91863500

C -4.70398800 2.58423000 2.23888800

H -5.24788400 3.34813100 2.78844000

C -4.38833000 2.77590700 0.89196100

H -4.68418800 3.69086600 0.38504300

C -3.69788300 1.79158900 0.18505600

H -3.47502300 1.95667400 -0.86368100

C -3.42465700 -0.82966500 -1.68696200

C -4.81615500 -0.62632100 -1.65716500

H -5.30993200 -0.37957800 -0.72265400

C -5.57445400 -0.72667500 -2.82407900

H -6.64900100 -0.56859000 -2.78212300

C -4.95505200 -1.02600100 -4.03976900

H -5.54630000 -1.10145300 -4.94870400

C -3.57549400 -1.23023300 -4.08029200

H -3.08544300 -1.47157100 -5.01972500

C -2.81333700 -1.13471900 -2.91344900

H -1.74708300 -1.33208700 -2.94599100

C -2.72747000 -2.23301100 0.80764000

C -1.86034300 -2.54915400 1.86548300

H -0.99270300 -1.92472500 2.05852700

C -2.09406300 -3.67029900 2.66166000

H -1.41627900 -3.90111800 3.47941500

C -3.18322300 -4.50335400 2.39738600

H -3.35789100 -5.38414500 3.00952000

C -4.03878800 -4.20574400 1.33623700

H -4.88141400 -4.85584600 1.11592400

C -3.81640800 -3.07499900 0.54768200

H -4.48537000 -2.86189700 -0.27913200

C -0.33407000 2.86834200 -1.10911700

C -1.09975500 2.37992300 -2.17712700

H -1.20163700 1.30756100 -2.31407800

C -1.71963500 3.25807900 -3.07042100

H -2.30646600 2.85985500 -3.89356800

C -1.57881800 4.63609200 -2.90596800

H -2.05962900 5.32064600 -3.59961700

C -0.80256000 5.13476800 -1.85573000

H -0.67512600 6.20696100 -1.73303000

C -0.17501500 4.25893900 -0.97040200

H 0.45434800 4.65826900 -0.18064100

C 2.24093900 2.37551900 -0.03426000

C 2.83266600 2.61914300 -1.28552700

H 2.28912900 2.38771000 -2.19732300

C 4.11040400 3.16791700 -1.37098900

H 4.55185800 3.35389200 -2.34633300

C 4.82198700 3.47513300 -0.20790400

H 5.81957600 3.90039500 -0.27485000

C 4.24440000 3.23166400 1.03693600

H 4.78813400 3.46947200 1.94738900

C 2.96052800 2.68504500 1.12617300

H 2.52636400 2.50601100 2.10317100

C -0.02969200 2.00028500 1.73337600

C 0.38206200 1.08179300 2.71548200

H 0.96946600 0.21171000 2.43709100

C 0.04933700 1.28408300 4.05522500

H 0.38804100 0.56986700 4.80041800

C -0.71411100 2.39194800 4.42908200

H -0.97756600 2.54703800 5.47202900

C -1.14634200 3.29368600 3.45601100

H -1.75557000 4.14862500 3.73613700

C -0.80518700 3.10324100 2.11538200

H -1.16030300 3.80800400 1.37248600

Pd 0.13399900 -0.56235300 -0.61499400

C 2.09354000 -0.77379100 -1.09362900

C 3.14459200 -1.10938400 -0.32140100

H 2.23681000 -0.67141100 -2.16909800

Br -0.04431700 -2.94293000 -1.49334000

C 4.49753500 -1.30298500 -0.93343700

C 5.35416300 -2.31791200 -0.46729800

C 4.94680300 -0.51125100 -2.00537400

C 6.59724800 -2.54129600 -1.05840900

H 5.03348100 -2.94599100 0.35831600

C 6.18875400 -0.73492300 -2.59830100

H 4.31976900 0.29910200 -2.36241400

C 7.02240300 -1.75202100 -2.12898900

H 7.23324600 -3.33944200 -0.68320000

H 6.51078500 -0.10453200 -3.42397500

H 7.99226400 -1.92436000 -2.58846000

C 3.02883900 -1.36049800 1.14751700

C 2.06706400 -2.24747700 1.66301800

C 3.90219500 -0.72829500 2.05178400

C 1.97272100 -2.47694600 3.03894900

H 1.41298500 -2.77065100 0.97249100

C 3.79855700 -0.94862100 3.42550800

H 4.66377000 -0.05563000 1.66792500

C 2.83123300 -1.82469700 3.92700100

H 1.23584200 -3.18370500 3.41376000

H 4.47866000 -0.44121300 4.10557000

H 2.76063700 -2.00944800 4.99616300

**Int3**

P -2.23002300 -0.72209500 -0.38614500

C -2.69146800 -1.73535600 -1.84602400

C -1.90314600 -2.84451300 -2.18955600

H -1.04169400 -3.09712700 -1.57969700

C -2.24998000 -3.63495900 -3.28449100

H -1.63561800 -4.49408200 -3.53923300

C -3.37775500 -3.32766600 -4.04837500

H -3.64195400 -3.94340900 -4.90374100

C -4.16659200 -2.22771700 -3.70855800

H -5.04751100 -1.98444800 -4.29615200

C -3.82714700 -1.43406700 -2.61228600

H -4.44297700 -0.57921800 -2.35483800

C -3.16691700 0.83070700 -0.65821300

C -2.78945100 1.67128000 -1.71618800

H -1.96898200 1.38202600 -2.36321500

C -3.45431700 2.87458100 -1.93588400

H -3.13784700 3.52481000 -2.74556300

C -4.51283500 3.25038100 -1.10366100

H -5.02766900 4.19226900 -1.27083300

C -4.90531300 2.41210200 -0.06056700

H -5.72994700 2.69547800 0.58748100

C -4.23343900 1.20884800 0.16544800

H -4.53214300 0.57178000 0.98951000

C -3.08346800 -1.48191500 1.03481800

C -2.65389400 -1.11805900 2.31755000

H -1.80134100 -0.45937200 2.43261800

C -3.31899700 -1.60639300 3.43902700

H -2.98296000 -1.31459600 4.42989800

C -4.40179600 -2.47610700 3.28746100

H -4.91367400 -2.86586000 4.16304000

C -4.82187100 -2.85086600 2.01012100

H -5.65842200 -3.53351500 1.88968400

C -4.16814500 -2.35193800 0.88235600

H -4.49659600 -2.64234700 -0.11037600

Pd 0.11047500 -0.63068400 -0.09792800

C 0.17059700 1.26976600 -0.73090100

C -0.15145900 2.35958300 -0.00825000

H 0.58969500 1.40498100 -1.72742600

C 0.04090900 3.72784400 -0.55234200

C 0.36172400 4.79484800 0.30536000

C -0.01467800 3.99100700 -1.93229100

C 0.64129300 6.06512100 -0.19788300

H 0.40457800 4.61761900 1.37517300

C 0.26605800 5.25784000 -2.43648900

H -0.27934900 3.19026000 -2.61454000

C 0.59935000 6.30384300 -1.57246600

H 0.89443800 6.86981200 0.48754800

H 0.21802000 5.43165800 -3.50836600

H 0.81483100 7.29343100 -1.96558300

C -0.64364000 2.23750600 1.39200500

C 0.07591300 1.50989000 2.35188800

C -1.84961600 2.84109200 1.77978100

C -0.39942900 1.37268900 3.65674800

H 1.02006300 1.05719300 2.07927800

C -2.33709100 2.68914000 3.07682000

H -2.41272200 3.41089300 1.04842800

C -1.61397900 1.95564900 4.02182300

H 0.18236900 0.80646400 4.37907700

H -3.28610400 3.14246700 3.35006700

H -1.99336600 1.84469800 5.03395500

P 2.47239600 -0.55943400 -0.04575200

C 3.24089200 -2.00506400 -0.85455600

C 3.19804400 0.87297600 -0.92718100

C 3.19383500 -0.47157100 1.63565800

C 4.54734700 -2.40726100 -0.55186400

C 2.51404900 -2.68705700 -1.83915900

C 3.71506400 0.73539000 -2.22137500

C 3.07037800 2.15887500 -0.37559000

C 2.52049500 -1.09932700 2.69560200

C 4.39507000 0.20826800 1.88740900

H 5.10908700 -1.89071500 0.22001100

C 5.12279000 -3.47931600 -1.23321200

C 3.09678700 -3.75076500 -2.52613200

H 1.49087000 -2.39124100 -2.04664600

H 3.81510700 -0.25071200 -2.66235800

C 4.09495200 1.86435600 -2.95102300

C 3.44503400 3.28267000 -1.10661000

H 2.65575600 2.28420600 0.61803900

H 1.60016500 -1.64041800 2.49831900

C 3.04499700 -1.04112300 3.98615400

C 4.91364000 0.26236900 3.18149800

H 4.91618500 0.70685100 1.07700800

H 6.13384000 -3.79309200 -0.98943800

C 4.40000200 -4.14925000 -2.22253100

H 2.52732600 -4.27794900 -3.28615600

H 4.49544800 1.74447300 -3.95382600

C 3.95545000 3.13748300 -2.39887200

H 3.30739600 4.27053200 -0.67838500

H 2.51772100 -1.53185800 4.79938100

C 4.23836600 -0.35904300 4.23306700

H 5.84250700 0.79439200 3.36684000

H 4.84945100 -4.98556300 -2.75062600

H 4.23530700 4.01565300 -2.97364300

H 4.64066400 -0.31112300 5.24115200

Br 0.12265600 -3.04775800 0.80025400

**Int4**

Pd 0.16029000 -0.67150900 -0.41931800

C -0.78875400 1.09418000 -0.62791900

C -1.75127000 1.67337500 0.12511800

H -0.46688300 1.62159300 -1.52694000

C -2.35487800 2.96416400 -0.34328800

C -2.62650500 4.00439500 0.56542100

C -2.62535300 3.19405700 -1.70552900

C -3.13063800 5.22956300 0.12867300

H -2.43535700 3.85010500 1.62353800

C -3.13109800 4.41963300 -2.14117100

H -2.45256900 2.39624000 -2.42242800

C -3.38452100 5.44491300 -1.22783100

H -3.32551300 6.01817400 0.85152700

H -3.33595600 4.56848700 -3.19845800

H -3.78232900 6.39760000 -1.56743000

C -2.23445000 1.12550300 1.42827000

C -1.35675100 0.53672200 2.35535100

C -3.60005600 1.17936500 1.76760100

C -1.82289200 0.00415100 3.55838700

H -0.29592200 0.49807500 2.13548200

C -4.06946600 0.64411900 2.96654800

H -4.29970200 1.64272600 1.07874300

C -3.18278100 0.05117900 3.86969200

H -1.11581800 -0.44110500 4.25341500

H -5.13106400 0.69089200 3.19632600

H -3.54724400 -0.35980300 4.80749300

P 2.11867900 0.46561700 0.10162900

C 3.54817300 -0.17011500 -0.86500200

C 2.13593800 2.27692700 -0.24765800

C 2.60353100 0.33596700 1.87651800

C 4.85188500 -0.11673300 -0.35218700

C 3.33861000 -0.66221500 -2.16130700

C 2.86045800 2.80490400 -1.32639600

C 1.36289900 3.14745500 0.54082400

C 2.30766100 -0.84068100 2.58557900

C 3.29152300 1.37416600 2.52838900

H 5.02929600 0.24874400 0.65424200

C 5.92916100 -0.54688300 -1.12863600

C 4.41892900 -1.07911100 -2.93836400

H 2.32876700 -0.73766700 -2.55236600

H 3.46259800 2.15399300 -1.95071800

C 2.81767800 4.17329900 -1.60573400

C 1.32838000 4.51242800 0.26335100

H 0.78389700 2.75958500 1.37216000

H 1.79798300 -1.65795100 2.08339800

C 2.69306600 -0.96914300 3.92081000

C 3.67216700 1.23776700 3.86447400

H 3.52659200 2.29178400 1.99964400

H 6.93466300 -0.51108700 -0.71815200

C 5.71554300 -1.02491700 -2.42246800

H 4.24379800 -1.46267100 -3.93956100

H 3.38573300 4.56565500 -2.44485100

C 2.05464300 5.02928500 -0.81277400

H 0.72029200 5.16885000 0.87884800

H 2.46072400 -1.88577800 4.45599300

C 3.37165300 0.06780200 4.56403300

H 4.20174500 2.04910200 4.35663600

H 6.55552100 -1.36179600 -3.02399600

H 2.02042000 6.09283900 -1.03259800

H 3.66577500 -0.03486700 5.60524100

Br 1.34648700 -2.95395500 -0.21473600

C -1.81771500 -1.92590100 -1.46677900

C -2.78037400 -0.89721000 -1.99114900

N -1.03368700 -2.32351700 -2.49400500

C -2.20448900 -3.00264600 -0.49361200

O -2.62277000 -0.29505000 -3.03638900

O -3.83986900 -0.76474100 -1.18452800

N -0.39091100 -2.64003700 -3.36247100

C -2.09967000 -4.35900200 -0.83147900

C -2.69916600 -2.65111600 0.77081800

C -4.85949800 0.14244000 -1.64260100

C -2.47913000 -5.34588400 0.07732700

H -1.71411100 -4.65601500 -1.80259400

C -3.08712600 -3.64201300 1.67017200

H -2.78120900 -1.60814100 1.04900700

H -4.47762400 1.16458900 -1.67085900

H -5.66604100 0.05227200 -0.91501000

C -2.97707300 -4.99216100 1.33137200

H -2.38450300 -6.39199900 -0.20042900

H -3.46622300 -3.34946300 2.64540700

H -3.27304900 -5.76126200 2.03957700

H -5.20345800 -0.14705900 -2.63870000

**TS2**

Pd 0.02268500 -0.73948400 -0.45215000

C -0.69428700 1.14094900 -0.65165700

C -1.55743300 1.85031700 0.10926800

H -0.27257800 1.63150800 -1.52994100

C -1.91625700 3.24847900 -0.29455800

C -2.11075900 4.25396400 0.67145100

C -2.03175600 3.61557000 -1.64918100

C -2.39420300 5.56870800 0.29981600

H -2.03454700 4.00038000 1.72461400

C -2.31575500 4.92938300 -2.02133700

H -1.90406300 2.85642600 -2.41600900

C -2.49801300 5.91520600 -1.04892700

H -2.53435900 6.32456900 1.06890500

H -2.40292500 5.18058100 -3.07557000

H -2.72486900 6.93792700 -1.33823500

C -2.15285300 1.33109200 1.37767600

C -1.37836700 0.64018400 2.32517800

C -3.51590400 1.52863300 1.66686200

C -1.94432100 0.14973100 3.50369900

H -0.31844400 0.49673600 2.14403500

C -4.08528900 1.03379000 2.83891900

H -4.13406200 2.07393800 0.95966000

C -3.30173000 0.33989900 3.76487300

H -1.31556300 -0.37171800 4.22132700

H -5.14387300 1.19112000 3.03063600

H -3.74375400 -0.03911300 4.68268500

P 2.18060500 0.19924400 0.09864200

C 3.55628000 -0.61061300 -0.82074700

C 2.41919500 1.98919500 -0.29631000

C 2.64514000 0.07161000 1.88221200

C 4.84715600 -0.70803900 -0.28329400

C 3.31446800 -1.09595400 -2.11435700

C 3.23729400 2.40436900 -1.35753400

C 1.71645900 2.96336800 0.43461600

C 2.23905400 -1.06072500 2.60905200

C 3.41222200 1.05621400 2.52734100

H 5.04845800 -0.34951700 0.72133100

C 5.87957700 -1.27715000 -1.03109300

C 4.35066500 -1.65241700 -2.86388300

H 2.31059200 -1.05300900 -2.52570700

H 3.78926800 1.67299500 -1.93743300

C 3.35316500 3.75974800 -1.67661900

C 1.83705200 4.31508900 0.11658200

H 1.07038500 2.66822200 1.25436700

H 1.67312000 -1.84268200 2.11098500

C 2.59031800 -1.19674900 3.95289500

C 3.75813300 0.91458800 3.87267500

H 3.73668100 1.93780400 1.98480100

H 6.87443000 -1.35560800 -0.60079100

C 5.63458200 -1.74657600 -2.32248200

H 4.14999500 -2.02748400 -3.86368900

H 3.99307900 4.06207500 -2.50136700

C 2.65513100 4.71750300 -0.94188400

H 1.27712700 5.05063000 0.68672600

H 2.27273700 -2.07990400 4.50084400

C 3.34568100 -0.20981400 4.58929700

H 4.34956000 1.68623500 4.35841100

H 6.43898500 -2.19124900 -2.90237700

H 2.74298600 5.77104900 -1.19324600

H 3.61363100 -0.31691500 5.63715600

Br 1.05474600 -3.12410900 -0.18163300

C -1.86467000 -1.53221100 -1.12058000

C -2.66584400 -0.59550000 -2.00486600

N -1.21599600 -2.50330900 -2.45571900

C -2.63533600 -2.52482100 -0.32549000

O -2.38129900 -0.28368000 -3.14427200

O -3.73633200 -0.14313100 -1.33647400

N -0.67411900 -3.21148400 -3.11225500

C -3.79438300 -3.13751600 -0.84762700

C -2.23246400 -2.84472900 0.98054900

C -4.54933700 0.82397200 -2.02785300

C -4.52342500 -4.04336700 -0.08736000

H -4.12024300 -2.90377000 -1.85808200

C -2.98076700 -3.73387600 1.75207500

H -1.33779400 -2.38810700 1.38133700

H -4.01641300 1.77241900 -2.11677400

H -5.43761600 0.94402500 -1.40763200

C -4.11824300 -4.33928700 1.21948500

H -5.40827200 -4.51472200 -0.50570000

H -2.66387900 -3.96181700 2.76543600

H -4.69149500 -5.04254900 1.81786700

H -4.81420100 0.45565600 -3.02165400

**Int5**

Pd -0.09805900 -0.71020400 -0.50186100

C -0.34276700 1.27782600 -0.77742700

C -1.06897500 2.11117000 -0.01226100

H 0.23168400 1.67745900 -1.61256400

C -1.10567400 3.57119500 -0.27259900

C -1.23737400 4.47832600 0.79324300

C -0.93851000 4.09447000 -1.56656800

C -1.17283600 5.85459000 0.57737200

H -1.37594100 4.09416500 1.79904900

C -0.87294300 5.46810500 -1.78311400

H -0.85960100 3.41137800 -2.40644700

C -0.98510100 6.35748800 -0.71128100

H -1.26825300 6.53537000 1.41925400

H -0.74084700 5.84723000 -2.79309200

H -0.93693700 7.42947200 -0.88073300

C -1.81362500 1.59296900 1.16910800

C -1.14589100 0.91318400 2.19794400

C -3.20737500 1.72751300 1.24791400

C -1.85197900 0.35598200 3.26282400

H -0.06817200 0.81213700 2.15771200

C -3.91790100 1.15901000 2.30407300

H -3.73081000 2.24912100 0.45308200

C -3.24308600 0.46824000 3.31358300

H -1.31144200 -0.17251800 4.04363100

H -5.00056600 1.24602200 2.33526700

H -3.79853700 0.02140900 4.13343100

P 2.17250600 -0.18909400 0.11901900

C 3.46884000 -1.24105200 -0.61943300

C 2.70023000 1.51365500 -0.30827800

C 2.40765100 -0.30562100 1.93316300

C 4.73288000 -1.36980600 -0.02944900

C 3.19858500 -1.88460100 -1.83403500

C 3.51941500 1.74887200 -1.42009900

C 2.13583900 2.61022400 0.36555200

C 1.71571500 -1.30767900 2.63253000

C 3.24309200 0.57337800 2.63767800

H 4.94179700 -0.88416000 0.91884800

C 5.71980500 -2.13058600 -0.65436300

C 4.19199200 -2.63683000 -2.46043800

H 2.20722600 -1.81378200 -2.26879700

H 3.95924900 0.91380100 -1.95505100

C 3.76638600 3.05466200 -1.84987000

C 2.37854300 3.91078800 -0.06842600

H 1.48677900 2.44847900 1.21809900

H 1.08941400 -2.00401100 2.08299300

C 1.85350200 -1.41660300 4.01545500

C 3.37467500 0.46020500 4.02244700

H 3.77846000 1.35405500 2.10783100

H 6.69665100 -2.23359900 -0.19023000

C 5.45129800 -2.76122500 -1.87160300

H 3.97467800 -3.13827200 -3.39889200

H 4.40428900 3.22253200 -2.71314400

C 3.19294500 4.13571200 -1.18076100

H 1.90702100 4.74292800 0.44508400

H 1.31653700 -2.19726500 4.54717600

C 2.67709900 -0.53081400 4.71416300

H 4.02061600 1.14932400 4.55918300

H 6.22104600 -3.35587000 -2.35562300

H 3.37277000 5.14968700 -1.52636900

H 2.77674800 -0.61396500 5.79282700

Br 0.36661500 -3.21427900 -0.20413900

C -2.04455700 -0.90829000 -0.85835500

C -2.49040800 -0.31937300 -2.14573200

C -3.00802900 -1.55106900 -0.03222000

O -2.19987500 -0.79072200 -3.22643000

O -3.20850500 0.80938100 -1.97701800

C -4.37970900 -1.62296600 -0.40578800

C -2.60813800 -2.14982700 1.19142500

C -3.43834900 1.57719100 -3.17128700

C -5.29851100 -2.26648400 0.40269200

H -4.70283200 -1.16400300 -1.33285400

C -3.53724000 -2.79236900 1.99744300

H -1.56573200 -2.10274500 1.47057900

H -2.49999200 1.72315700 -3.71128900

H -3.83698500 2.53167800 -2.82899500

C -4.87564800 -2.85420300 1.60436800

H -6.34196200 -2.32098800 0.10853500

H -3.22089400 -3.24732200 2.93049700

H -5.59981100 -3.36203500 2.23573400

H -4.15208100 1.06752800 -3.82377300

**Int6**

Pd -0.44041400 0.34674200 0.02972500

C -1.80802400 -1.04121400 1.05388900

C -0.84865500 -1.87807200 0.39933900

H -1.65612800 -0.93007200 2.12297700

C 0.02644000 -2.75016900 1.24859700

C 1.19352500 -3.31378900 0.70183000

C -0.28136300 -3.05346000 2.58667800

C 2.03565800 -4.11397700 1.46870200

H 1.44751500 -3.11665600 -0.33395800

C 0.56407800 -3.85377800 3.35692000

H -1.20443100 -2.69518300 3.03106000

C 1.73073400 -4.38311700 2.80511200

H 2.93430000 -4.52675600 1.01849600

H 0.29566900 -4.07717800 4.38621200

H 2.38683600 -5.00904900 3.40370900

P 1.93896000 0.58384500 -0.22236200

C 2.60370700 2.14567500 -0.95249500

C 2.81799900 0.50968200 1.39990800

C 2.72782600 -0.73036500 -1.25091300

C 3.34916100 2.16876700 -2.13938800

C 2.36182800 3.35432700 -0.27697400

C 4.09982200 1.06091100 1.57113700

C 2.20107900 -0.12107100 2.48954300

C 2.15665700 -1.01644700 -2.50305600

C 3.85843300 -1.45163200 -0.84090100

H 3.55404800 1.24924800 -2.67639000

C 3.84544300 3.37534600 -2.64020800

C 2.86913100 4.55310400 -0.77384700

H 1.76940900 3.36061300 0.63066900

H 4.58796800 1.56566200 0.74339500

C 4.74686400 0.97472000 2.80422100

C 2.85130200 -0.20840100 3.72175900

H 1.21162100 -0.54876200 2.37267000

H 1.27487900 -0.47262200 -2.82786100

C 2.71282700 -1.99325300 -3.32959200

C 4.40570600 -2.43764300 -1.66578500

H 4.31157400 -1.25090400 0.12400100

H 4.42165300 3.37564200 -3.56173100

C 3.60943600 4.56841400 -1.95853000

H 2.67258100 5.47881500 -0.24014800

H 5.73610300 1.40858000 2.92366700

C 4.12411200 0.34032400 3.88201100

H 2.35902700 -0.70514900 4.55310400

H 2.26621000 -2.19736100 -4.29909100

C 3.83650800 -2.71061400 -2.91030700

H 5.28020400 -2.99060100 -1.33300200

H 3.99735400 5.50610200 -2.34768400

H 4.62857000 0.27839300 4.84264800

H 4.26420000 -3.47750700 -3.55035400

C -2.78611000 -0.18474300 0.50055400

C -3.51324900 -0.47694000 -0.77463100

C -4.12975200 -1.72649800 -0.94978900

C -3.70610000 0.51862200 -1.74728300

C -4.91252400 -1.98121500 -2.07927400

H -4.00910000 -2.49639900 -0.19302700

C -4.47943400 0.25740700 -2.87690800

H -3.22506500 1.48307300 -1.61210900

C -5.08847200 -0.99047500 -3.04650900

H -5.38868500 -2.95137900 -2.19453500

H -4.61157700 1.03406600 -3.62573300

H -5.69962800 -1.18602900 -3.92386600

C -1.10150400 -2.48670100 -0.97054400

O -1.32176600 -3.66848800 -1.13140700

O -1.03094400 -1.60607300 -1.98616000

C -3.45489400 0.77521200 1.43163500

C -4.83607200 1.02197000 1.32857700

C -2.74832200 1.41403900 2.46961500

C -5.48749200 1.85302200 2.24024600

H -5.40945100 0.54653800 0.54085300

C -3.39980500 2.24070800 3.37961300

H -1.67075500 1.30036000 2.53068300

C -4.77529300 2.46347900 3.27201200

H -6.55682400 2.01995300 2.14087900

H -2.82670300 2.73198800 4.16138100

H -5.28181200 3.11660300 3.97756400

C -1.32651300 -2.14014800 -3.28984100

H -1.26660900 -1.28915200 -3.96810400

H -0.59518100 -2.90748100 -3.55731700

H -2.32971200 -2.56998500 -3.30194500

Br -0.96971300 2.77483600 -0.45312000

**Int7**

Pd -0.51241200 0.24058800 -0.00899400

C -1.84618100 -0.98876000 1.21801000

C -0.79801200 -1.84655600 0.73922900

H -1.76402500 -0.67751600 2.25462400

C 0.14493800 -2.46048900 1.73363500

C 1.31782700 -3.08354300 1.27072400

C -0.09657600 -2.46641900 3.11804300

C 2.22517400 -3.66282500 2.15384000

H 1.52621700 -3.10365000 0.20566300

C 0.81416200 -3.04418200 4.00396700

H -1.01392600 -2.04724500 3.51848900

C 1.98200800 -3.64134900 3.52892300

H 3.12562200 -4.12931600 1.76360600

H 0.59734600 -3.03982800 5.06904400

H 2.68804000 -4.09375000 4.21983300

P 1.85082600 0.55946700 -0.37448400

C 2.32625200 2.00221400 -1.43575700

C 2.77085600 0.88734200 1.18625200

C 2.74086200 -0.85666200 -1.16345600

C 2.81325000 1.85167900 -2.74207500

C 2.17905600 3.29812700 -0.91109400

C 4.05475200 1.45957700 1.17852500

C 2.17978700 0.55818600 2.41263000

C 2.19462200 -1.41753600 -2.33084600

C 3.92363400 -1.40253600 -0.64386000

H 2.95308700 0.86386900 -3.16657200

C 3.14258700 2.97136400 -3.50997300

C 2.51524300 4.41150500 -1.68047400

H 1.78374800 3.43735500 0.08963600

H 4.51888100 1.74020800 0.23779900

C 4.73383900 1.68348000 2.37591500

C 2.86232600 0.78103900 3.60973600

H 1.18243800 0.13364400 2.42835400

H 1.26553100 -1.02474200 -2.73139400

C 2.82882500 -2.48150000 -2.97334400

C 4.54888100 -2.47748500 -1.28054700

H 4.35750400 -0.99497600 0.26234200

H 3.52102000 2.83451500 -4.51966400

C 2.99626600 4.25371800 -2.98246000

H 2.39695600 5.40611500 -1.25867000

H 5.72482800 2.12929100 2.35775300

C 4.13882300 1.34343700 3.59373300

H 2.39039100 0.51998100 4.55270100

H 2.39876600 -2.89665000 -3.88105100

C 4.00710100 -3.01738800 -2.44751300

H 5.46330600 -2.88975700 -0.86203000

H 3.25554900 5.12397100 -3.57969900

H 4.66758100 1.52335000 4.52608000

H 4.49630600 -3.85168800 -2.94296100

C -2.79990800 -0.27994900 0.45944900

C -3.35991100 -0.74826300 -0.85016400

C -4.01401600 -1.98655700 -0.94057400

C -3.34841800 0.10224500 -1.96959800

C -4.63233000 -2.37504300 -2.13284900

H -4.04381300 -2.64403300 -0.07693600

C -3.95539200 -0.29558600 -3.15972200

H -2.84393000 1.06178200 -1.89409800

C -4.60257700 -1.53256500 -3.24526300

H -5.14153900 -3.33390300 -2.18475300

H -3.92959400 0.36621800 -4.02159700

H -5.08559500 -1.83395300 -4.17121100

C -0.86179200 3.13784100 0.03714800

O -1.02480200 2.15326600 -0.78994900

O -0.29851300 3.06102800 1.13952400

C -1.42861200 4.46479000 -0.46393300

H -1.20490700 5.26514100 0.24478600

H -1.00723700 4.70728800 -1.44524000

H -2.51424700 4.37875100 -0.58496900

C -1.05479000 -2.79826000 -0.42166300

O -1.47673500 -3.92277400 -0.25344900

O -0.73545700 -2.30435300 -1.63121200

C -3.58929400 0.78222900 1.15802300

C -4.96610000 0.92242000 0.90091200

C -3.00488400 1.62842200 2.12012600

C -5.73459600 1.85292500 1.59965000

H -5.44233300 0.28614000 0.16295900

C -3.77484900 2.56133900 2.81129600

H -1.93145200 1.61233400 2.27610700

C -5.14435000 2.67474500 2.56058000

H -6.79798600 1.93358900 1.38971100

H -3.29377000 3.21671500 3.53209600

H -5.74183800 3.40612300 3.09842800

C -0.98491000 -3.19133000 -2.73759000

H -0.70726700 -2.62757500 -3.62835900

H -0.37699200 -4.09485700 -2.64334800

H -2.04147900 -3.46237600 -2.77133500

**Int8**

Pd 0.30352500 1.06088600 0.30468200

C -2.21002700 0.36856200 0.74512600

C -1.00952600 -0.40826600 1.13828600

H -2.18274000 1.38151300 1.13479200

C -0.62180400 -0.32036100 2.59325900

C 0.55883900 -0.93445000 3.06331000

C -1.39842600 0.38930700 3.52655000

C 0.94754100 -0.83076900 4.39452600

H 1.17215200 -1.50710200 2.37533200

C -1.00443700 0.49944400 4.86177000

H -2.32886500 0.85056900 3.21466100

C 0.17025000 -0.10570200 5.30431200

H 1.86082700 -1.31916100 4.72549400

H -1.62779000 1.05614900 5.55671900

H 0.47520600 -0.02255700 6.34408100

P 2.11777400 -0.09694200 -0.60262900

C 2.12523600 0.03072500 -2.44075300

C 3.58449300 0.87751800 -0.03468000

C 2.64416200 -1.83897100 -0.27123900

C 3.00459700 -0.71482300 -3.24493400

C 1.24701900 0.93762300 -3.05351200

C 4.68612700 1.13993600 -0.86114700

C 3.59399600 1.35863200 1.28472600

C 2.14301700 -2.90051800 -1.04445000

C 3.53991400 -2.12826700 0.77235700

H 3.68505200 -1.42927300 -2.79261800

C 3.00230000 -0.55515300 -4.63123500

C 1.24883400 1.09506300 -4.44026200

H 0.57196900 1.52519800 -2.43960700

H 4.69099200 0.79855000 -1.89104700

C 5.77946000 1.85795800 -0.37338300

C 4.69343900 2.06553900 1.77311600

H 2.73363900 1.18847600 1.92682800

H 1.42963400 -2.70512400 -1.83586900

C 2.55244300 -4.21130100 -0.79424600

C 3.93153300 -3.44316000 1.03032900

H 3.94870300 -1.32527100 1.37686700

H 3.68542900 -1.14057800 -5.24094000

C 2.12373600 0.34909500 -5.23148900

H 0.56137900 1.79936000 -4.90048000

H 6.62324900 2.06054000 -1.02772700

C 5.78837000 2.31794500 0.94436200

H 4.68623300 2.42992400 2.79686200

H 2.16852900 -5.01781000 -1.41368200

C 3.44530600 -4.48813500 0.24364100

H 4.62829300 -3.64580000 1.83925400

H 2.12012500 0.46919500 -6.31161600

H 6.64030200 2.87782900 1.32095900

H 3.76070600 -5.51015000 0.43588400

C -3.29767400 0.07741700 -0.02839300

C -3.69258900 -1.29768000 -0.45172000

C -4.01036800 -2.26869800 0.51029900

C -3.85081100 -1.62127100 -1.80997200

C -4.46650900 -3.53251400 0.12509200

H -3.91156500 -2.02325400 1.56362500

C -4.28987500 -2.88581100 -2.19478900

H -3.61795900 -0.87373700 -2.56266300

C -4.60442500 -3.84599900 -1.22781100

H -4.72189800 -4.26688200 0.88498700

H -4.39289500 -3.12248100 -3.25060600

H -4.96148500 -4.82766400 -1.52845700

C 0.24145000 3.67698700 0.30760400

O -0.67405600 3.07437700 0.94653000

O 1.13154500 3.02477700 -0.33083200

C 0.31041900 5.18854400 0.32054800

H 1.04115200 5.50594000 1.07410300

H 0.64906300 5.56319900 -0.64939300

H -0.66126800 5.61634400 0.57788400

C -0.87676600 -1.76759100 0.48168900

O -0.71898100 -1.96002800 -0.70828200

O -0.98722800 -2.77413800 1.37633700

C -0.92628100 -4.10645300 0.84203500

H -1.70527900 -4.25116100 0.09044700

H -1.09020900 -4.76480200 1.69658300

H 0.05342700 -4.29554700 0.39893400

C -4.23362600 1.17237100 -0.40694600

C -5.59915000 0.90498400 -0.63144300

C -3.79355300 2.50533100 -0.54591400

C -6.48889100 1.92455800 -0.96523500

H -5.96767700 -0.11027000 -0.52923400

C -4.68645100 3.52186800 -0.87885900

H -2.74636500 2.74660100 -0.39550000

C -6.03805300 3.23959300 -1.09167600

H -7.53834800 1.68941900 -1.12382700

H -4.32008900 4.54008900 -0.98295700

H -6.73007100 4.03456400 -1.35713500

**TS4**

Pd 0.11302800 0.56789700 0.25905500

C -1.93892400 0.05613700 0.46221600

C -1.16568500 -1.16220900 0.32682100

H -1.60525700 0.98801200 1.41968700

C -0.76787200 -1.93981000 1.54404000

C 0.21376000 -2.95028900 1.49866200

C -1.35121100 -1.65026600 2.79214000

C 0.58345600 -3.64367200 2.64827700

H 0.69117500 -3.18909500 0.55521400

C -0.97725000 -2.34376000 3.94393700

H -2.10108900 -0.86803500 2.86053800

C -0.00936200 -3.34648500 3.87943300

H 1.34277000 -4.41899100 2.58283800

H -1.44800500 -2.09742600 4.89223000

H 0.28097600 -3.89002600 4.77467700

P 2.35079700 0.18531100 -0.38810700

C 3.02895200 1.54109300 -1.44289900

C 3.51633400 0.10682200 1.03976300

C 2.70730200 -1.33932900 -1.36352700

C 3.89948200 1.30671500 -2.51862000

C 2.65675600 2.86203600 -1.13694400

C 4.84997000 0.53659100 0.95234600

C 3.04128600 -0.40041200 2.25961000

C 1.90045100 -1.60823000 -2.48364000

C 3.73672400 -2.23425400 -1.03643400

H 4.19014300 0.29241500 -2.77350900

C 4.39206400 2.37365700 -3.27343900

C 3.15823600 3.92338800 -1.89154500

H 1.97277600 3.05615100 -0.31542700

H 5.22857000 0.94510600 0.02018900

C 5.69252100 0.45187000 2.06201300

C 3.88830500 -0.48974500 3.36536100

H 2.00721300 -0.72161400 2.34268400

H 1.07715900 -0.94550200 -2.73402500

C 2.13791600 -2.73654900 -3.26873300

C 3.96035500 -3.37164900 -1.81711500

H 4.36777900 -2.04525800 -0.17402400

H 5.06232900 2.17837600 -4.10655300

C 4.02398700 3.68320000 -2.96106800

H 2.86405200 4.94060100 -1.64641300

H 6.72175400 0.79229100 1.98358500

C 5.21409600 -0.06272500 3.26919300

H 3.50655300 -0.88558600 4.30249200

H 1.51387100 -2.92411200 -4.13867600

C 3.16666200 -3.62283800 -2.93664200

H 4.75967700 -4.05775500 -1.54928300

H 4.40644200 4.51210500 -3.55099600

H 5.87100900 -0.12437000 4.13289300

H 3.34518800 -4.50502300 -3.54580700

C -3.14800800 0.41971100 -0.06755600

C -4.17361400 -0.58749200 -0.44420900

C -4.30799200 -1.79898400 0.25960900

C -5.02828200 -0.35601000 -1.53998600

C -5.25352800 -2.74814400 -0.12597800

H -3.68010800 -1.98746100 1.12377500

C -5.96442200 -1.31101800 -1.93018800

H -4.93595000 0.56835800 -2.10044900

C -6.08344400 -2.51052900 -1.22371000

H -5.34607600 -3.67184000 0.43906300

H -6.60137600 -1.11839900 -2.78949700

H -6.82066700 -3.25093700 -1.52293500

C -0.59316400 2.80746600 2.01270600

O -1.51610700 1.97351800 2.31097900

O 0.28125600 2.64617800 1.11588300

C -0.59407300 4.10906800 2.79439600

H 0.36266900 4.62394900 2.68952200

H -1.39120200 4.74979000 2.39966200

H -0.81272300 3.91839800 3.84832700

C -1.30476300 -1.85388700 -1.00409800

O -1.41127100 -1.29667100 -2.08084400

O -1.35462900 -3.20218600 -0.88367100

C -1.55268200 -3.92031400 -2.10909100

H -2.48808100 -3.61420100 -2.58454000

H -1.59184300 -4.97294000 -1.82507000

H -0.72312700 -3.74032100 -2.79790400

C -3.50417500 1.85403100 -0.22455600

C -4.76272500 2.33620000 0.18067500

C -2.58767700 2.75999400 -0.78540700

C -5.08212300 3.68645300 0.05592300

H -5.48281300 1.64773400 0.61281500

C -2.91248200 4.11006500 -0.91935000

H -1.62574900 2.39152300 -1.12782300

C -4.15781800 4.57850000 -0.49523900

H -6.05270800 4.04382200 0.38981900

H -2.19369000 4.79386200 -1.36321400

H -4.41090600 5.63035000 -0.60043300

**Int9**

Pd 0.00287300 0.47737900 -0.00607400

C -1.97112700 -0.07565000 0.02529100

C -1.22831900 -1.28663800 -0.00357300

H -1.29399200 1.47697000 1.72140700

C -0.92881800 -2.05892800 1.24829000

C 0.03522500 -3.08470400 1.26609300

C -1.57582700 -1.74698300 2.45807200

C 0.33181000 -3.76965500 2.44276400

H 0.55843300 -3.34294100 0.35205800

C -1.27639300 -2.43145000 3.63627600

H -2.32458700 -0.95984900 2.47061000

C -0.32007300 -3.44833100 3.63652300

H 1.08120700 -4.55704400 2.42637800

H -1.79753300 -2.17021500 4.55395200

H -0.08666400 -3.98462100 4.55246400

P 2.41426300 0.15210600 -0.29427500

C 3.29895300 1.32847300 -1.41477600

C 3.33365400 0.28009800 1.30300200

C 2.91291000 -1.49728700 -0.96191400

C 4.39502400 0.94913300 -2.20586900

C 2.84444000 2.65688800 -1.47429700

C 4.61959200 0.83020400 1.41500700

C 2.69298300 -0.19197500 2.46148100

C 2.21955700 -1.98088000 -2.08538600

C 3.93426600 -2.28023300 -0.40438800

H 4.75279300 -0.07555700 -2.18219700

C 5.02718300 1.87933200 -3.03351300

C 3.48314700 3.58437100 -2.29897500

H 1.99171600 2.96212100 -0.87571300

H 5.12670300 1.20816400 0.53252600

C 5.25261000 0.90181100 2.65809500

C 3.33178500 -0.12831700 3.70030500

H 1.69195400 -0.60937300 2.38933900

H 1.40411200 -1.40269100 -2.51097400

C 2.55686800 -3.21315500 -2.64595200

C 4.25941200 -3.52034900 -0.96105600

H 4.47674200 -1.92590400 0.46655900

H 5.87239700 1.57004700 -3.64303800

C 4.57360800 3.19870900 -3.08134800

H 3.12091500 4.60856500 -2.33643900

H 6.24705500 1.33466500 2.73100400

C 4.61209600 0.42122500 3.80172900

H 2.82457400 -0.50169800 4.58589400

H 2.01843300 -3.56989300 -3.52026600

C 3.57554500 -3.98775400 -2.08391500

H 5.05019500 -4.11846800 -0.51548700

H 5.06398600 3.92069800 -3.72914200

H 5.10626800 0.47870500 4.76809700

H 3.83146400 -4.95117700 -2.51708200

C -3.20035500 0.43052300 -0.18255700

C -4.41015700 -0.43324300 -0.34278600

C -4.48669200 -1.72498900 0.21116700

C -5.50663300 0.01984400 -1.10178600

C -5.60904600 -2.52689200 0.01261100

H -3.66706100 -2.10384400 0.81027000

C -6.62696200 -0.78571700 -1.30313600

H -5.47381500 1.00721300 -1.55002400

C -6.68689700 -2.06348600 -0.74518500

H -5.64185300 -3.51745400 0.45928000

H -7.45384100 -0.41165600 -1.90168100

H -7.56213300 -2.68970400 -0.89698100

C -0.40434800 3.12765700 1.57667300

O -1.24965500 2.33419700 2.22385900

O 0.25187300 2.75785400 0.60473000

C -0.35501500 4.52596000 2.12837500

H -1.25292500 5.05867200 1.79478800

H -0.35976700 4.50981400 3.22141600

H 0.53008900 5.04225500 1.75526500

C -1.25102000 -1.98631300 -1.33733800

O -1.12868800 -1.45312400 -2.42420500

O -1.50182300 -3.31191800 -1.20645400

C -1.62130300 -4.03781400 -2.43752700

H -2.43354100 -3.63192400 -3.04612800

H -1.83498200 -5.06763300 -2.14813700

H -0.68966300 -3.98581600 -3.00806400

C -3.38097300 1.91137500 -0.30907600

C -4.33143000 2.59508500 0.46878500

C -2.59443900 2.65161600 -1.20610700

C -4.47492900 3.97870500 0.36960200

H -4.95526200 2.03553800 1.16060800

C -2.74227800 4.03642800 -1.31124200

H -1.87078000 2.12806200 -1.82382500

C -3.67971600 4.70602400 -0.52147100

H -5.20944800 4.48967100 0.98706600

H -2.12867900 4.58977400 -2.01773100

H -3.79982200 5.78308600 -0.60779700

**Int10**

Pd -0.15657600 -0.59518100 0.03574800

C 1.95631600 0.22012900 -0.65683900

C 2.86773200 1.16673800 -0.24472100

H 1.17854900 0.61280400 -1.34126200

C 2.53621800 2.60435400 -0.37624600

C 3.49055100 3.51966000 -0.86536500

C 1.25708000 3.08847900 -0.04352600

C 3.16646100 4.86249600 -1.03940400

H 4.48182700 3.16490700 -1.12944700

C 0.93632900 4.43455500 -0.21468400

H 0.52678500 2.40667500 0.38085700

C 1.88787500 5.32497700 -0.71502100

H 3.91208400 5.54923700 -1.43113700

H -0.05673000 4.78407500 0.05060500

H 1.63906700 6.37510800 -0.84326400

C 4.22969900 0.81112000 0.21490300

C 4.94281400 -0.24621200 -0.38036700

C 4.85154800 1.54319700 1.24435300

C 6.22647700 -0.57212600 0.05332500

H 4.49474300 -0.80218400 -1.19784200

C 6.12970200 1.20730700 1.68431500

H 4.31576600 2.36291800 1.71246500

C 6.82284800 0.14931500 1.08973100

H 6.76232200 -1.38852200 -0.42277300

H 6.58622600 1.77255900 2.49243100

H 7.82300000 -0.10698600 1.42853100

P -2.39305800 0.34314400 0.06941600

C -3.20594400 0.52862400 1.71001100

C -2.50810400 2.01507000 -0.71132900

C -3.57883400 -0.66168400 -0.92791200

C -4.58016200 0.79956900 1.83467700

C -2.42469700 0.38617400 2.86610300

C -2.95002900 3.15505500 -0.02518200

C -2.09172900 2.14987600 -2.04844400

C -3.42002500 -2.05782400 -0.93238500

C -4.63474700 -0.08815200 -1.65506900

H -5.20222700 0.88507000 0.94833500

C -5.15606400 0.94764700 3.09518000

C -3.00930000 0.53365300 4.12745100

H -1.37831700 0.10650500 2.78629100

H -3.27452300 3.07487500 1.00698500

C -2.98947100 4.39770600 -0.66634800

C -2.13800400 3.38720700 -2.68717200

H -1.74120400 1.27787000 -2.59580300

H -2.59788000 -2.50755100 -0.38178400

C -4.31279400 -2.85998100 -1.64572600

C -5.52134200 -0.89700600 -2.36786000

H -4.76049000 0.99009700 -1.67661100

H -6.21939200 1.15555500 3.18175500

C -4.36912200 0.81910400 4.24427500

H -2.39682700 0.40788500 5.01587900

H -3.34257700 5.26998200 -0.12218900

C -2.58908100 4.51663800 -1.99724500

H -1.81971200 3.47109900 -3.72276400

H -4.18006500 -3.93863400 -1.64514600

C -5.36273300 -2.28425500 -2.36360400

H -6.33335100 -0.44149200 -2.92883400

H -4.82160900 0.92900600 5.22650400

H -2.62500300 5.48180700 -2.49522800

H -6.05186200 -2.91253700 -2.92195900

C 1.84828400 -1.21897300 -0.43859900

C 2.40196700 -1.85305500 0.80963700

C 1.69588000 -2.08718200 -1.64456100

O 2.62971300 -3.04240600 0.93309900

O 2.58656000 -0.95910700 1.79896300

C 1.19096000 -3.40180800 -1.56049000

C 2.05747500 -1.59804400 -2.91609700

C 2.98583100 -1.50242400 3.06703900

C 1.06018500 -4.18386300 -2.70498600

H 0.88057300 -3.78516000 -0.59923200

C 1.92494200 -2.38580900 -4.05907400

H 2.46501700 -0.59480900 -3.00691000

H 2.13626800 -2.02217900 3.51419900

H 3.26844600 -0.63992700 3.67209400

C 1.42611000 -3.68506200 -3.95847700

H 0.66488200 -5.19261500 -2.61703800

H 2.21976500 -1.98481400 -5.02551400

H 1.32374400 -4.30346000 -4.84645100

H 3.83234800 -2.18231000 2.94485500

C -0.57057700 -2.68629700 2.07140900

O -0.75394800 -2.50631800 0.79587200

O -0.16812200 -1.83459500 2.87077500

C -0.85578400 -4.11512200 2.53223200

H -1.08192200 -4.12510800 3.60106500

H -1.67586000 -4.56442400 1.96573600

H 0.04442700 -4.71735300 2.35983500

**TS5**

Pd -0.10085100 -0.52721100 0.15181400

C 2.06229900 -0.00165300 -0.16309400

C 2.78268000 1.12387100 -0.14414200

H 0.43071400 0.63095300 -0.77324100

C 2.16368400 2.47071800 0.00324800

C 2.54224300 3.52688600 -0.84243900

C 1.19757400 2.70932800 0.99408100

C 1.94868500 4.78224200 -0.71777600

H 3.29959500 3.35725500 -1.60260900

C 0.61310400 3.96990800 1.12382500

H 0.92307800 1.90344900 1.66795600

C 0.98200900 5.00745200 0.26582600

H 2.24275400 5.58587500 -1.38790300

H -0.12869000 4.14065200 1.89931000

H 0.52379100 5.98774900 0.36591700

C 4.27310600 1.02890900 -0.22352600

C 4.91480900 0.01253900 -0.95281300

C 5.07106500 1.95849400 0.46766000

C 6.30536300 -0.07513400 -0.98258500

H 4.32373600 -0.70717700 -1.50889200

C 6.46205200 1.86651300 0.43840000

H 4.59672500 2.74828200 1.04081900

C 7.08641000 0.85052000 -0.28732000

H 6.77888900 -0.86646000 -1.55759200

H 7.05800200 2.59071800 0.98764900

H 8.17059600 0.78213800 -0.31352400

P -2.27642200 0.31375000 -0.18041700

C -3.29411000 0.43584600 1.34374900

C -2.41062100 1.96835800 -0.98559200

C -3.22180400 -0.81585700 -1.28744200

C -4.69715900 0.37628700 1.28913600

C -2.65829600 0.60685300 2.58191000

C -3.28141600 2.96297900 -0.51869000

C -1.62066100 2.23443800 -2.11608800

C -3.10418800 -2.20044100 -1.07081400

C -4.04834700 -0.34440400 -2.31836600

H -5.19950400 0.21865700 0.33906600

C -5.45107400 0.50925100 2.45491300

C -3.41962300 0.73765300 3.74571500

H -1.57535500 0.58074600 2.64724000

H -3.89895600 2.78047700 0.35407400

C -3.35933700 4.19708000 -1.17021000

C -1.70864100 3.46121600 -2.77060500

H -0.93012200 1.47951800 -2.48212700

H -2.45715900 -2.56794200 -0.27824900

C -3.81386300 -3.09168600 -1.87563500

C -4.75228800 -1.24540700 -3.12084000

H -4.14132000 0.72146000 -2.50101400

H -6.53556200 0.46036800 2.40371900

C -4.81289100 0.69468100 3.68442900

H -2.91632900 0.85517000 4.70125000

H -4.03411700 4.96047700 -0.79204600

C -2.57725100 4.44832700 -2.29676000

H -1.09061400 3.65023200 -3.64397300

H -3.71645800 -4.16030500 -1.70405700

C -4.63665600 -2.61855800 -2.90099200

H -5.38836300 -0.87094400 -3.91863300

H -5.40198400 0.79146900 4.59268400

H -2.63889200 5.40861600 -2.80170900

H -5.18276100 -3.31795800 -3.52862500

C 1.98942500 -1.37660300 -0.00506900

C 2.38572800 -1.93358600 1.35034500

C 1.82602300 -2.29078200 -1.18080600

O 2.33603300 -3.10847500 1.65869900

O 2.84020900 -0.97223700 2.16612500

C 1.50616300 -3.65430000 -1.04149700

C 1.97700000 -1.77197200 -2.48289800

C 3.07604800 -1.37673100 3.52630400

C 1.36718500 -4.46431100 -2.16852700

H 1.37093400 -4.06731100 -0.05248400

C 1.83492500 -2.58596400 -3.60349900

H 2.20558500 -0.71797600 -2.61182700

H 2.12050400 -1.63758500 3.98619800

H 3.51419500 -0.50379200 4.01129300

C 1.53110200 -3.94099500 -3.45183000

H 1.12319600 -5.51535700 -2.03730600

H 1.96292100 -2.16045600 -4.59545300

H 1.42087500 -4.57943500 -4.32438200

H 3.76517200 -2.22412700 3.56157000

C -0.64572700 -2.17444900 2.50178200

O -0.88817200 -2.20344800 1.22816600

O -0.10081600 -1.23276500 3.09860800

C -1.04283300 -3.44075000 3.25265900

H -1.21923300 -3.21557500 4.30738200

H -1.92720800 -3.90760400 2.81063600

H -0.21257100 -4.15397500 3.18414300

**Int11**

Pd -0.08531400 -0.37505800 0.12352900

C 2.20423000 -0.14778700 -0.17485200

C 2.87571200 0.98967400 -0.31804200

H 0.21687500 0.35289200 -1.21813400

C 2.21305500 2.31935100 -0.18731600

C 2.42502200 3.32402400 -1.14412800

C 1.37873800 2.58319200 0.91173100

C 1.79773800 4.56346900 -1.01875600

H 3.07892100 3.12933100 -1.98982300

C 0.76377600 3.83056200 1.03815900

H 1.22598300 1.81397500 1.66555600

C 0.96609100 4.82049000 0.07409800

H 1.96098000 5.32910400 -1.77279400

H 0.12807500 4.02919100 1.89711500

H 0.48421200 5.78939800 0.17613200

C 4.35330500 0.93319400 -0.54367100

C 4.95790200 -0.14500500 -1.21343300

C 5.17870700 1.96050000 -0.05312200

C 6.34028300 -0.19710700 -1.38033800

H 4.34112800 -0.94319400 -1.61362000

C 6.56209300 1.90527000 -0.22079300

H 4.73330000 2.79883000 0.47197200

C 7.14997300 0.82790800 -0.88556300

H 6.78469200 -1.03833100 -1.90576900

H 7.18105300 2.70712900 0.17326300

H 8.22759500 0.78815400 -1.01979400

P -2.28588300 0.26827000 -0.28341600

C -3.16617000 0.88665700 1.20515200

C -2.58631300 1.53751300 -1.58824000

C -3.25834300 -1.20061900 -0.82260600

C -4.55574200 0.73483400 1.34458800

C -2.43840200 1.53516000 2.21280100

C -3.50351900 2.58303300 -1.41003200

C -1.89547200 1.44001300 -2.80712300

C -3.09613600 -2.39922100 -0.10435300

C -4.14866500 -1.15628600 -1.90524000

H -5.12663100 0.21476200 0.58098200

C -5.20735700 1.24255300 2.46887300

C -3.09594800 2.03884500 3.33674300

H -1.35848100 1.60461300 2.14274600

H -4.04477800 2.67978000 -0.47520300

C -3.72383300 3.51136800 -2.43099600

C -2.12760200 2.35932900 -3.82849500

H -1.16879900 0.64588200 -2.95322300

H -2.39998500 -2.43671000 0.73006700

C -3.82702800 -3.52954900 -0.46898500

C -4.87318400 -2.29554700 -2.26437100

H -4.27698700 -0.23898200 -2.47069600

H -6.28231600 1.11887100 2.56989100

C -4.47854700 1.89779200 3.46473700

H -2.52024600 2.52557000 4.11893100

H -4.43042600 4.32197700 -2.27430500

C -3.04081500 3.40059700 -3.64158000

H -1.58635300 2.26884800 -4.76632400

H -3.69674500 -4.45279500 0.08918300

C -4.71430100 -3.48200400 -1.54770700

H -5.55954500 -2.25239500 -3.10596000

H -4.98700800 2.28576900 4.34346700

H -3.21282300 4.12404900 -4.43399400

H -5.27723800 -4.36779700 -1.83014100

C 2.00470000 -1.45776500 0.13992400

C 2.35748500 -1.86334400 1.56745500

C 1.70572300 -2.50579900 -0.88951500

O 2.04911100 -2.91250600 2.09523900

O 3.10186700 -0.92084200 2.16023200

C 1.26965900 -3.79811300 -0.54743100

C 1.87036700 -2.19397300 -2.25396000

C 3.35895200 -1.10525900 3.56310500

C 1.02358000 -4.74455900 -1.54330500

H 1.12828100 -4.05004800 0.49434500

C 1.62360800 -3.14269300 -3.24140000

H 2.18802000 -1.19441700 -2.53503600

H 2.44111400 -0.89236700 4.11594300

H 4.13288500 -0.37831000 3.81117600

C 1.19919000 -4.42738400 -2.89025100

H 0.68872700 -5.73837200 -1.25782200

H 1.76420200 -2.87886700 -4.28639600

H 1.00673200 -5.17034300 -3.65968200

H 3.70331400 -2.12252400 3.76212400

C -0.38260200 -1.00585900 3.00700400

O -0.75179900 -1.46574700 1.85525800

O 0.29271600 0.02183500 3.18578700

C -0.80987400 -1.86817000 4.19110900

H -0.72022700 -1.30897600 5.12545100

H -1.83561600 -2.22659900 4.06284100

H -0.15457500 -2.74612800 4.23507300

**Int6’**

Pd -0.26677700 -0.69044300 -0.42216600

C -1.76704000 0.46932200 -1.54187600

C -1.48366500 1.55284100 -0.68473900

H -1.34364500 0.54011000 -2.53792900

C -0.80217100 2.74591800 -1.27389600

C -0.04636500 3.60145400 -0.44874000

C -0.92739400 3.08551000 -2.63437100

C 0.57749000 4.73337000 -0.96605200

H 0.05215900 3.36540000 0.60601400

C -0.30546600 4.22239300 -3.15117500

H -1.54601300 2.48065200 -3.29012500

C 0.45282500 5.04982900 -2.32116000

H 1.16347500 5.37086000 -0.30960100

H -0.43161100 4.47021100 -4.20182200

H 0.93191900 5.93837700 -2.72309300

C -2.11156800 1.80585700 0.65923700

C -1.91778800 0.99871000 1.78831900

C -2.94125200 2.93689700 0.78628700

C -2.54672500 1.29572100 2.99748300

H -1.26579000 0.13332400 1.72269900

C -3.58120000 3.22571000 1.98964800

H -3.09456000 3.58595500 -0.07085300

C -3.38543700 2.40521900 3.10261700

H -2.37405200 0.65510000 3.85735600

H -4.22920400 4.09561000 2.05760400

H -3.87761400 2.63308000 4.04433700

P 1.95167100 -0.20764500 0.37490300

C 3.18955800 -1.57495700 0.42437300

C 2.91121700 1.07814900 -0.53856100

C 1.82213700 0.36443300 2.11870000

C 4.09126100 -1.73746000 1.48564600

C 3.28472300 -2.42232700 -0.68979700

C 4.14968400 1.55240100 -0.07176900

C 2.44780700 1.50811300 -1.78819700

C 1.36659700 -0.56312900 3.07448000

C 2.04699400 1.69147600 2.51173800

H 4.02758100 -1.09873100 2.36094100

C 5.07327300 -2.72927300 1.43073100

C 4.27293100 -3.40391900 -0.74563800

H 2.57282800 -2.32675800 -1.50283800

H 4.53997800 1.20871000 0.88171000

C 4.89373300 2.45167600 -0.83330200

C 3.19954200 2.40122100 -2.55585900

H 1.49683900 1.14103100 -2.16123600

H 1.16400500 -1.58934600 2.77800300

C 1.16586400 -0.17070700 4.39703000

C 1.83579000 2.08109700 3.83721600

H 2.39061100 2.42301500 1.78751800

H 5.76235300 -2.84924000 2.26263800

C 5.16802300 -3.56133500 0.31491700

H 4.33218700 -4.05705000 -1.61188900

H 5.84923800 2.81159600 -0.46096700

C 4.42004700 2.87687800 -2.07833100

H 2.82365100 2.72708800 -3.52122200

H 0.82210300 -0.89940700 5.12623000

C 1.39966900 1.15277300 4.78227700

H 2.01560100 3.11265700 4.12828800

H 5.93166500 -4.33352200 0.27398900

H 5.00568900 3.57216200 -2.67395400

H 1.23885200 1.45772000 5.81277800

Br -0.02297000 -3.16302600 0.01383200

C -2.36173500 -0.79905000 -1.22894900

C -2.33532400 -1.84814600 -2.31051800

C -3.48092100 -0.98256400 -0.25114400

O -3.02998500 -2.84091700 -2.32057500

O -1.44338800 -1.56942300 -3.30048400

C -4.54570300 -0.06793600 -0.23867400

C -3.54775400 -2.10571100 0.58752800

C -1.32695700 -2.58690600 -4.30510000

C -5.63610400 -0.25198400 0.61127100

H -4.52193800 0.78979000 -0.90480000

C -4.63618600 -2.28570800 1.43911000

H -2.73465600 -2.82293900 0.57322100

H -0.99203600 -3.52448400 -3.85417800

H -0.58558600 -2.21428900 -5.01343800

C -5.68271900 -1.36040900 1.45774800

H -6.44890400 0.46963200 0.60759400

H -4.66794200 -3.15742800 2.08772100

H -6.53120900 -1.50605400 2.12155900

H -2.28649600 -2.75184000 -4.80285500

**Int7’**

Pd -0.30538400 -0.58284500 -0.39775300

C -1.73787100 0.59411300 -1.57582900

C -1.33209300 1.68823700 -0.78946100

H -1.36410100 0.55429200 -2.59522300

C -0.55670000 2.78656600 -1.44569200

C 0.22464200 3.65492700 -0.65883900

C -0.61399600 3.02667400 -2.83176500

C 0.93427800 4.70567700 -1.23400700

H 0.27443700 3.49517300 0.41359700

C 0.09349200 4.08285600 -3.40634700

H -1.24019500 2.40861300 -3.46711600

C 0.87293000 4.92597300 -2.61208500

H 1.53645800 5.35496200 -0.60424400

H 0.01945600 4.25501900 -4.47686100

H 1.41839400 5.75180800 -3.06060400

C -1.95730800 2.08517700 0.52414600

C -1.74088600 1.42903000 1.74144100

C -2.79900700 3.21372800 0.51583700

C -2.35757800 1.87355200 2.91258000

H -1.08199700 0.56808000 1.77621100

C -3.42541700 3.64925200 1.68141000

H -2.96732900 3.74769900 -0.41522600

C -3.20532300 2.98021000 2.88767500

H -2.16675600 1.34904300 3.84444100

H -4.08143500 4.51516400 1.64705800

H -3.68687300 3.32289100 3.79966800

P 1.95048100 -0.25405600 0.38085000

C 2.99181900 -1.77543600 0.45795000

C 3.05648500 0.89148900 -0.54482700

C 1.87966500 0.34423900 2.11853000

C 3.85433200 -2.04672700 1.53076000

C 2.97429400 -2.64209900 -0.64561100

C 4.34105500 1.22348900 -0.07921600

C 2.64747000 1.35426800 -1.80185400

C 1.28053300 -0.50849300 3.06542900

C 2.27963600 1.62786200 2.51537500

H 3.87462300 -1.39184400 2.39626400

C 4.68636900 -3.16752600 1.49904800

C 3.81018500 -3.75902700 -0.67271000

H 2.28249600 -2.47151100 -1.46433700

H 4.68630600 0.84800000 0.87977500

C 5.18573800 2.02015800 -0.84983900

C 3.49966900 2.14431200 -2.57761800

H 1.66226300 1.09405400 -2.17544500

H 0.93622700 -1.49360600 2.76018800

C 1.11434100 -0.08984000 4.38463500

C 2.10244600 2.04516900 3.83770100

H 2.73144300 2.30469400 1.79725400

H 5.34724100 -3.36998700 2.33796900

C 4.66772200 -4.02411100 0.39700500

H 3.77817900 -4.42788800 -1.52824700

H 6.17618100 2.27137000 -0.47961300

C 4.76586500 2.48150700 -2.10145000

H 3.16576200 2.49784800 -3.54850100

H 0.65820800 -0.76108100 5.10745000

C 1.52594100 1.18840600 4.77500900

H 2.41839600 3.04252100 4.13195900

H 5.31411200 -4.89762000 0.37502100

H 5.42937400 3.09638800 -2.70383900

H 1.39251500 1.51435600 5.80299900

C -2.38031600 -0.61751500 -1.16439900

C -2.43899000 -1.63177000 -2.28621100

C -3.43311800 -0.71843200 -0.09985300

O -2.03837400 -1.42339100 -3.41661700

O -3.01068600 -2.78431000 -1.90067900

C -4.54973200 0.13014100 -0.15802100

C -3.37643200 -1.68922700 0.91206900

C -2.97124100 -3.83900100 -2.87759200

C -5.57790400 0.02321100 0.77902800

H -4.61242200 0.87681500 -0.94501500

C -4.40406600 -1.79252100 1.84871300

H -2.51167100 -2.34194200 0.96663400

H -3.43283200 -3.51376800 -3.81325000

H -3.53607200 -4.66126400 -2.43593500

C -5.50817200 -0.93872800 1.78749600

H -6.43250800 0.69218300 0.71849400

H -4.34148600 -2.54505300 2.63103000

H -6.30815200 -1.02361500 2.51857300

H -1.93208300 -4.12687100 -3.05141800

C -0.12975000 -3.52237700 -0.44894100

O -0.23038300 -2.54849700 0.40819900

O 0.03572400 -3.39331800 -1.66554700

C -0.25457100 -4.90575400 0.18889800

H 0.06150800 -5.67569400 -0.51822400

H 0.34280400 -4.96725500 1.10335000

H -1.30086400 -5.08395500 0.46479000

Int8’

Pd -0.47301500 -1.20199800 0.07328900

C 2.29656800 -1.07666400 0.05367300

C 1.29415700 -0.07255000 0.57033400

H 1.94966100 -2.10537600 0.01661600

C 1.10952500 -0.08780000 2.06328700

C 0.25435500 0.82967100 2.71402300

C 1.75580600 -1.05058900 2.86580900

C 0.05972700 0.78478100 4.09116400

H -0.26743600 1.57852200 2.12974800

C 1.55142800 -1.09945200 4.24547000

H 2.42650400 -1.76750900 2.40436700

C 0.70522100 -0.18240100 4.86848800

H -0.60364800 1.50804700 4.55892800

H 2.06431100 -1.85631200 4.83328300

H 0.55169200 -0.21637400 5.94372400

P -2.20378200 0.35729900 -0.29101600

C -2.58302800 0.50177500 -2.09070700

C -3.69814400 -0.45691400 0.42502100

C -2.32178800 2.07687900 0.36969400

C -3.17184800 1.63714000 -2.66802300

C -2.29606500 -0.60842500 -2.90449900

C -4.95612500 -0.37297500 -0.18883100

C -3.57314400 -1.15521800 1.63593300

C -1.56646800 3.11256000 -0.20592000

C -3.12576500 2.36744100 1.48585400

H -3.40496600 2.50231300 -2.05627400

C -3.45589700 1.66623300 -4.03551700

C -2.58802400 -0.57583600 -4.26784100

H -1.86042500 -1.49999200 -2.46161400

H -5.06869500 0.15368200 -1.13130200

C -6.06938400 -0.97237900 0.40238800

C -4.68976100 -1.74452300 2.22956900

H -2.59807300 -1.24391100 2.10707500

H -0.92733600 2.91552100 -1.05910300

C -1.63384000 4.40782800 0.30889500

C -3.18123700 3.66248800 2.00268500

H -3.71575000 1.58521400 1.95015600

H -3.90711900 2.55392300 -4.47092300

C -3.16319300 0.56266300 -4.83776700

H -2.36244400 -1.44110700 -4.88524000

H -7.03813100 -0.90607900 -0.08567200

C -5.93926600 -1.65639800 1.61243200

H -4.57943500 -2.28279400 3.16702700

H -1.04389300 5.19375200 -0.15378600

C -2.43964300 4.68788100 1.41342700

H -3.81241600 3.86859800 2.86303300

H -3.38325500 0.58855200 -5.90171000

H -6.80713100 -2.12415300 2.06974100

H -2.48770900 5.69708900 1.81365400

C 3.57050300 -0.88082300 -0.37204900

C 4.37932300 0.36171800 -0.21885600

C 4.61220900 0.88583300 1.06195500

C 4.98232900 0.98855600 -1.32026200

C 5.41155100 2.01633300 1.23719000

H 4.17039800 0.39626000 1.92510800

C 5.77058900 2.12462300 -1.14690500

H 4.83243800 0.58042800 -2.31426100

C 5.98972300 2.64208200 0.13233500

H 5.58487700 2.40346500 2.23815800

H 6.22266600 2.60236200 -2.01229800

H 6.61341300 3.52236700 0.26596000

C -0.87044100 -3.77371100 -0.05081300

O 0.25336800 -3.33217300 0.34597500

O -1.79656800 -2.97357400 -0.39933100

C -1.12642300 -5.26442800 -0.07309900

H -1.61077800 -5.55781600 0.86612600

H -1.80090600 -5.52337900 -0.89351000

H -0.18669900 -5.81470500 -0.16160900

C 1.39448200 1.25053500 -0.15403600

C 1.73532400 2.45644500 0.47795700

C 1.24735600 1.26778800 -1.55600300

C 1.92544500 3.62882800 -0.25841700

H 1.89181800 2.47559300 1.55022400

C 1.43031200 2.43558000 -2.29166900

H 0.98846100 0.34495200 -2.06754700

C 1.77410100 3.62780200 -1.64405900

H 2.20715300 4.54218000 0.25917500

H 1.30990500 2.41465500 -3.37180700

H 1.92957800 4.53881700 -2.21610300

C 4.31286100 -2.03287700 -0.98537400

O 5.44719300 -1.96359300 -1.42017900

O 3.59211900 -3.17895400 -1.02432000

C 4.26860700 -4.30719200 -1.58998500

H 4.54523900 -4.11067400 -2.62982100

H 3.55756100 -5.13190300 -1.53101900

H 5.17497600 -4.53932800 -1.02340700

**TS4’**

Pd 0.15429200 0.73240300 0.05670300

C -1.96943500 0.66734300 0.31688200

C -1.43087400 -0.67016400 0.49360500

H -1.36510800 1.75830700 0.92141400

C -1.06622400 -1.12536500 1.87530800

C -0.25475300 -2.25917500 2.08911200

C -1.50655800 -0.41238600 3.00753000

C 0.09327900 -2.66096500 3.37647900

H 0.11815900 -2.81569200 1.23556000

C -1.15436900 -0.81529100 4.29624000

H -2.12492300 0.47033000 2.87479200

C -0.35481600 -1.94270900 4.48967700

H 0.72308100 -3.53683400 3.51022400

H -1.51161100 -0.24497500 5.14972800

H -0.08353300 -2.25934600 5.49318400

P 2.30583600 -0.15621600 -0.36118800

C 3.09611700 0.60364400 -1.84516900

C 3.44236900 0.26488200 1.02902300

C 2.54085400 -1.96872900 -0.60349100

C 3.87369500 -0.13027200 -2.75320400

C 2.89808800 1.97887700 -2.06494000

C 4.77899900 0.64140000 0.82861200

C 2.93933300 0.20296300 2.33894400

C 1.64287500 -2.64540000 -1.44572300

C 3.56756300 -2.69462400 0.01937600

H 4.03331600 -1.19265400 -2.59889300

C 4.44370500 0.49824300 -3.86307000

C 3.47736100 2.60105800 -3.17099200

H 2.29547800 2.55599600 -1.36905000

H 5.18054800 0.70447100 -0.17821600

C 5.59750500 0.94491300 1.91866900

C 3.76205300 0.49999800 3.42574100

H 1.90201100 -0.07303700 2.50672900

H 0.82956600 -2.10377000 -1.91790300

C 1.77524800 -4.01626000 -1.66745000

C 3.69422000 -4.06823500 -0.20052100

H 4.26787600 -2.19016000 0.67724600

H 5.04080800 -0.08237300 -4.56143700

C 4.24753100 1.86358900 -4.07399000

H 3.32025600 3.66463400 -3.33066500

H 6.62981600 1.24027700 1.75017200

C 5.09180300 0.87350500 3.21797300

H 3.35940800 0.44757800 4.43365900

H 1.06684900 -4.52359900 -2.31628800

C 2.80075300 -4.73124800 -1.04367200

H 4.49284800 -4.61890200 0.28973000

H 4.69047400 2.35083500 -4.93868300

H 5.72964500 1.11325600 4.06467100

H 2.89983500 -5.80064600 -1.21032000

C -3.16731400 1.11469100 -0.15237100

C -4.42168100 0.32449900 -0.15482000

C -4.66677300 -0.65118900 0.82766200

C -5.39976700 0.52798600 -1.14713000

C -5.83660600 -1.40781600 0.80969900

H -3.94602200 -0.80037600 1.62431300

C -6.56287700 -0.23740100 -1.17030500

H -5.24780100 1.28864500 -1.90486900

C -6.78789800 -1.20876200 -0.19225500

H -6.00758700 -2.14901500 1.58595400

H -7.29884600 -0.06965800 -1.95215600

H -7.70091500 -1.79811900 -0.20592500

C 0.02517200 3.43481700 1.14624900

O -1.07583400 2.93371200 1.55896400

O 0.85000600 2.86216700 0.38030000

C 0.36736500 4.82336900 1.66120500

H 0.80381900 4.72755800 2.66251400

H 1.09892100 5.30686500 1.01066800

H -0.53484300 5.43374000 1.74974700

C -1.77658600 -1.68972900 -0.55565500

C -2.21679600 -2.98156300 -0.22031700

C -1.73228600 -1.35097800 -1.92278800

C -2.58049500 -3.90014000 -1.20770000

H -2.29174300 -3.26986800 0.82238300

C -2.09842100 -2.26471500 -2.90752300

H -1.39196000 -0.35932600 -2.20645400

C -2.52071600 -3.55086700 -2.55592100

H -2.92011500 -4.89044700 -0.91486000

H -2.04904500 -1.97313200 -3.95362000

H -2.80352800 -4.26610900 -3.32372500

C -3.29304400 2.54442600 -0.60697600

O -4.29097000 3.22688100 -0.48765500

O -2.16539200 2.98751000 -1.20559400

C -2.17818000 4.37257500 -1.57852900

H -2.96795900 4.56687200 -2.30950500

H -1.19569100 4.56512900 -2.00986900

H -2.34548000 5.00131600 -0.69972500

**Int9’**

Pd 0.01180600 0.60405200 -0.12824000

C -2.03155100 0.47979400 0.02092700

C -1.57169200 -0.83239100 0.28905200

H -0.96994600 2.19749300 1.31987100

C -1.26430000 -1.26278500 1.69545000

C -0.48371900 -2.40713200 1.95255700

C -1.72396600 -0.52325900 2.80273500

C -0.17780600 -2.79078500 3.25728200

H -0.09998000 -2.98816600 1.12052500

C -1.41487300 -0.90691500 4.10751500

H -2.33237200 0.36150500 2.63775400

C -0.63978100 -2.04416900 4.34447600

H 0.43065000 -3.67619400 3.42321800

H -1.78882100 -0.31656900 4.94019900

H -0.40162100 -2.34623900 5.36074600

P 2.33876000 -0.14650100 -0.30170500

C 3.30931900 0.51634100 -1.73037100

C 3.31480100 0.37301000 1.17946900

C 2.64390700 -1.96712900 -0.39406800

C 4.24552500 -0.24898100 -2.44240300

C 3.07730500 1.84609600 -2.12421800

C 4.63055100 0.85346400 1.10866000

C 2.68888000 0.28439200 2.43494300

C 1.79474800 -2.73710500 -1.20566600

C 3.67789600 -2.60876800 0.30470900

H 4.43391700 -1.27934300 -2.15801400

C 4.93701900 0.30339900 -3.52322000

C 3.77670600 2.39575600 -3.19904600

H 2.35412100 2.44865000 -1.58326600

H 5.12656600 0.93491000 0.14631200

C 5.30825100 1.23287500 2.27013900

C 3.37143200 0.65496900 3.59377800

H 1.66576000 -0.07690900 2.50221500

H 0.97502400 -2.26184800 -1.73572500

C 1.98092000 -4.11508800 -1.32296400

C 3.85862200 -3.98933800 0.19112200

H 4.34168600 -2.03292400 0.94196700

H 5.65594500 -0.30347700 -4.06761000

C 4.70553200 1.62596600 -3.90349000

H 3.58922800 3.42571500 -3.49176300

H 6.32670700 1.60641500 2.20076900

C 4.68212100 1.13280700 3.51364700

H 2.87594100 0.57561000 4.55777700

H 1.30991600 -4.69417000 -1.95150600

C 3.01270900 -4.74497200 -0.62269900

H 4.66154200 -4.47302000 0.74154900

H 5.24275300 2.05354400 -4.74602300

H 5.21123600 1.42844500 4.41586400

H 3.15325800 -5.81941200 -0.70695300

C -3.16276300 1.15773700 -0.26503800

C -4.53000400 0.56971200 -0.12761000

C -4.80417900 -0.42469100 0.82699000

C -5.57346500 0.95591300 -0.99036600

C -6.06505100 -1.01297900 0.91553600

H -4.02852200 -0.73477500 1.51722700

C -6.83177800 0.36303900 -0.90454900

H -5.40117000 1.72758300 -1.73066500

C -7.08724400 -0.62373400 0.04921400

H -6.24773700 -1.77432700 1.66974900

H -7.61657500 0.67714200 -1.58806700

H -8.07111700 -1.08037700 0.11848700

C 0.31639600 3.54267900 1.03979300

O -0.73141100 3.07734700 1.71648600

O 0.85964800 2.91773900 0.13505200

C 0.76120700 4.90382300 1.50722600

H -0.07544300 5.60810600 1.46090600

H 1.08025000 4.84408300 2.55325000

H 1.58754100 5.25704200 0.89008800

C -1.85287900 -1.87087700 -0.76285400

C -2.42107800 -3.11123300 -0.42516100

C -1.64608200 -1.59287400 -2.12708200

C -2.75424600 -4.04178300 -1.41143100

H -2.61524300 -3.34777900 0.61556700

C -1.98285000 -2.51993500 -3.11128400

H -1.20590600 -0.63875700 -2.40487300

C -2.53504300 -3.75471100 -2.75859400

H -3.19432500 -4.99233700 -1.12049700

H -1.80784000 -2.27849300 -4.15667000

H -2.79276500 -4.48087000 -3.52497000

C -3.08771500 2.57181400 -0.75254200

O -3.98671700 3.38864900 -0.67968600

O -1.88898000 2.87517300 -1.31409800

C -1.76157600 4.22263400 -1.78304500

H -2.47405100 4.41912200 -2.58938900

H -0.73697400 4.30688900 -2.14603200

H -1.94766100 4.93604000 -0.97455600

**Int10’**

Pd -0.13318300 -0.36170700 0.06332500

C 1.81866400 0.74070000 -0.74566700

C 2.65128800 1.76909400 -0.38456200

H 0.86747300 1.09196300 -1.20184400

C 4.10774600 1.63759000 -0.16152400

C 4.87632200 0.80986900 -0.99918900

C 4.76307800 2.34877500 0.86085600

C 6.25114200 0.68512000 -0.81521300

H 4.39021800 0.27645600 -1.81024500

C 6.13651400 2.21435500 1.04909400

H 4.19263400 3.00276200 1.50938200

C 6.88662400 1.38490400 0.21233800

H 6.82654100 0.04464800 -1.47809300

H 6.62308100 2.76348300 1.85070900

H 7.95947100 1.28888000 0.35678900

P -2.48909400 0.18805300 0.17136800

C -3.25692400 0.64398300 1.78034400

C -2.87516800 1.59949700 -0.95852000

C -3.54620400 -1.18032900 -0.47192700

C -4.65144000 0.69817000 1.95126000

C -2.41759700 0.94540000 2.86294900

C -3.52205400 2.76995900 -0.53674300

C -2.44812300 1.50781700 -2.29612100

C -3.17338000 -2.49799200 -0.15671400

C -4.70351800 -0.95699100 -1.23478400

H -5.31220800 0.44048600 1.12853800

C -5.19528300 1.07044000 3.17962500

C -2.96985600 1.31702400 4.09216600

H -1.34233400 0.83553400 2.76135700

H -3.85644500 2.86471600 0.49100000

C -3.74591900 3.81952400 -1.43374500

C -2.67834500 2.55212300 -3.18982100

H -1.93509800 0.61258700 -2.64040300

H -2.26434500 -2.67757600 0.41224300

C -3.96007000 -3.56817800 -0.58638200

C -5.48191100 -2.03274900 -1.66585500

H -4.99401900 0.05481200 -1.50172900

H -6.27451700 1.10788900 3.30308600

C -4.35370900 1.38524000 4.25120800

H -2.31102100 1.53695400 4.92762700

H -4.25258200 4.71811300 -1.09112300

C -3.32817700 3.71351700 -2.76069100

H -2.34748900 2.46133600 -4.22100100

H -3.66449000 -4.58411400 -0.33778900

C -5.11333000 -3.33948600 -1.33988900

H -6.37460400 -1.84856400 -2.25805000

H -4.77964400 1.66985600 5.20994100

H -3.50534300 4.52899800 -3.45672700

H -5.71970100 -4.17600600 -1.67745100

C 1.90583700 -0.71040400 -0.64229600

C 1.45629100 -1.48369000 -1.84106200

C 1.02218700 -2.82254100 -1.71044900

C 1.43188500 -0.90220900 -3.12478500

C 0.58891700 -3.54080600 -2.82075800

H 0.99512800 -3.26941600 -0.72238700

C 0.99803300 -1.62762100 -4.23498200

H 1.77909000 0.11917800 -3.25578600

C 0.57625800 -2.94985600 -4.08882200

H 0.25064300 -4.56594200 -2.69544300

H 0.99978900 -1.15985200 -5.21617800

H 0.23985400 -3.51587700 -4.95326800

C -0.00758500 -1.91785500 2.51176300

O -0.21982200 -2.15214500 1.25078400

O 0.17886900 -0.80439600 3.01656200

C 0.04765300 -3.17902000 3.37330200

H -0.18915600 -2.93273400 4.41106300

H -0.63148100 -3.95065400 3.00082200

H 1.06774600 -3.58013500 3.33617700

C 2.09531400 3.16281700 -0.34069700

O 2.76232400 4.16687000 -0.17863700

O 0.75228500 3.20474600 -0.51009800

C 0.17742400 4.52064100 -0.53043000

H 0.35029000 5.02491200 0.42409200

H -0.88774800 4.36858500 -0.70122600

H 0.61848600 5.11986400 -1.33182900

C 2.85919800 -1.35342000 0.31137900

C 3.69251400 -2.40940600 -0.10090900

C 2.97749400 -0.89634300 1.63660200

C 4.60811000 -2.98808500 0.77821400

H 3.63786200 -2.77070400 -1.12255100

C 3.88822100 -1.47841000 2.51390600

H 2.31481900 -0.11705600 1.99398000

C 4.71041000 -2.52648600 2.09048100

H 5.24392100 -3.79893800 0.43180400

H 3.94727900 -1.11622600 3.53660200

H 5.42028700 -2.97919200 2.77785700

**TS5’**

Pd -0.10008000 -0.32700500 0.16668800

C 1.87203600 0.51119600 -0.22430200

C 2.47430200 1.70476100 -0.08284300

H 0.24525400 0.85304500 -0.82222000

C 3.92928400 1.87832600 -0.35835700

C 4.60298600 1.06276600 -1.28380100

C 4.67768400 2.84165300 0.34348100

C 5.97323800 1.19949400 -1.49725700

H 4.05053800 0.32317400 -1.85266900

C 6.04857600 2.97276000 0.13186800

H 4.18532500 3.49314700 1.05532100

C 6.70460000 2.15441100 -0.78918100

H 6.46765800 0.56064300 -2.22431200

H 6.60484600 3.72074700 0.69054600

H 7.77284100 2.26257500 -0.95653800

P -2.40404400 0.21445100 -0.10995900

C -3.36679000 0.48947700 1.43060200

C -2.70732300 1.71918900 -1.13676900

C -3.30144800 -1.12966800 -0.99553600

C -4.76843700 0.38563000 1.44857300

C -2.68332300 0.82901300 2.60742500

C -3.53075700 2.77235300 -0.71579300

C -2.06313600 1.81917900 -2.38254600

C -2.99789300 -2.45956000 -0.65478200

C -4.27631200 -0.87156200 -1.97165200

H -5.30587500 0.09882200 0.54929200

C -5.47545300 0.64094900 2.62302100

C -3.39891800 1.08240500 3.78087600

H -1.59790500 0.83243200 2.62328500

H -4.03781100 2.71852600 0.24167500

C -3.70382000 3.90189500 -1.52224900

C -2.24823200 2.93937900 -3.18986500

H -1.40900000 1.01851200 -2.71859100

H -2.23128800 -2.66260700 0.08968900

C -3.67434500 -3.50834200 -1.27895500

C -4.94524600 -1.92825500 -2.59321300

H -4.51074000 0.15014500 -2.25423100

H -6.55899600 0.55679900 2.62917900

C -4.79109400 0.99493400 3.78975100

H -2.85907700 1.32795500 4.69106900

H -4.33955300 4.71319400 -1.17764400

C -3.06708600 3.98793100 -2.75930200

H -1.74493500 2.99930900 -4.15103000

H -3.43415100 -4.53389000 -1.01112500

C -4.64682100 -3.24710400 -2.24721100

H -5.69735000 -1.71798400 -3.34916200

H -5.34392200 1.18728500 4.70561300

H -3.20189500 4.86668400 -3.38396200

H -5.16694500 -4.06822100 -2.73361000

C 2.09987500 -0.85699200 -0.33088000

C 1.79054900 -1.59574800 -1.59401400

C 1.51420200 -2.97565700 -1.56179800

C 1.75015500 -0.93259900 -2.83477300

C 1.21890900 -3.66722600 -2.73477100

H 1.50967500 -3.49299000 -0.60830700

C 1.45912800 -1.62906800 -4.00647500

H 1.95165000 0.13403700 -2.87688100

C 1.19365300 -2.99980200 -3.96205200

H 1.00158400 -4.73092600 -2.68856400

H 1.44342000 -1.10025500 -4.95581900

H 0.96650500 -3.54258500 -4.87551100

C -0.44102100 -1.87578200 2.64616900

O -0.53342600 -2.05882900 1.36439400

O -0.14119400 -0.80705200 3.19733400

C -0.72203200 -3.12804400 3.47478900

H -0.71773800 -2.88854500 4.54016200

H -1.68833800 -3.56126000 3.19488700

H 0.04488400 -3.88386700 3.26991700

C 1.70297200 2.92925400 0.31511700

O 2.01746400 4.06312700 0.00809300

O 0.61302400 2.64660100 1.05706200

C -0.19249200 3.78008200 1.41966600

H 0.39599800 4.49486400 2.00074400

H -1.00823400 3.37352300 2.01706500

H -0.58014400 4.27687400 0.52637300

C 2.93921300 -1.50719200 0.72496900

C 3.95997400 -2.39840600 0.34594800

C 2.80431800 -1.17558800 2.08373200

C 4.82125400 -2.94024400 1.29985600

H 4.09254700 -2.65510900 -0.69980400

C 3.66331900 -1.72669400 3.03229800

H 1.99284600 -0.53071400 2.40381800

C 4.67614700 -2.60846400 2.64730600

H 5.60830100 -3.62028300 0.98449500

H 3.52833200 -1.47177300 4.07984800

H 5.34268800 -3.03684000 3.39124800

**Int11’**

Pd 0.13206600 -0.19214000 -0.08915400

C -2.19454200 0.32604400 0.06643100

C -2.65048800 1.52812800 -0.25954900

H 0.06918400 1.03398000 0.88048200

C -3.99101300 2.02733600 0.16643500

C -5.02265200 1.11861300 0.46600200

C -4.25513000 3.40390100 0.27227800

C -6.27727300 1.57304500 0.86376400

H -4.84465000 0.05211900 0.36963700

C -5.51444400 3.85223800 0.67334200

H -3.46818800 4.11513700 0.05216400

C -6.52995100 2.94341600 0.97105400

H -7.06240100 0.85411700 1.08276500

H -5.69798000 4.92051500 0.75389300

H -7.50978900 3.29693000 1.28071800

P 2.37267700 0.29088200 0.16584100

C 3.54170200 -0.92305100 -0.57294100

C 2.88284200 1.92007700 -0.52469400

C 2.85944000 0.37576900 1.94137000

C 4.43342200 -1.66439100 0.21650500

C 3.52512100 -1.12208800 -1.96424600

C 4.14506900 2.11004800 -1.11056600

C 2.01049100 3.01435400 -0.41594100

C 2.10303500 -0.32845200 2.88890800

C 3.98084800 1.10636900 2.36509600

H 4.45613500 -1.52776800 1.29227600

C 5.30273800 -2.58288400 -0.37667500

C 4.40210000 -2.03493900 -2.54926400

H 2.80747500 -0.59229800 -2.58340900

H 4.82966000 1.27471800 -1.21405000

C 4.52496800 3.37142300 -1.57376700

C 2.39628200 4.27390000 -0.87598200

H 1.02311500 2.88345800 0.01401200

H 1.22460200 -0.88246300 2.57206500

C 2.46719000 -0.31042600 4.23624000

C 4.33720200 1.12848700 3.71407200

H 4.57084000 1.66629000 1.64581200

H 5.98812200 -3.15139300 0.24635300

C 5.29252800 -2.76699400 -1.75926500

H 4.38044800 -2.18016500 -3.62594200

H 5.50307800 3.50307900 -2.02875100

C 3.65335400 4.45525600 -1.45679500

H 1.70286900 5.10567000 -0.79041500

H 1.86812300 -0.85808000 4.95816500

C 3.58260700 0.41890700 4.65134100

H 5.20223600 1.70406700 4.03253200

H 5.97033100 -3.48120200 -2.21942600

H 3.94965900 5.43488800 -1.82222900

H 3.86026500 0.44074300 5.70188300

C -2.23089600 -0.97601500 0.42834900

C -1.80831700 -1.43870700 1.78891500

C -1.10975200 -2.64884600 1.95096900

C -2.13750900 -0.68046100 2.92354000

C -0.75208200 -3.08643200 3.22588600

H -0.83233800 -3.22203600 1.07161600

C -1.78062500 -1.12435000 4.19655800

H -2.68301600 0.25071700 2.80072100

C -1.08971900 -2.32901100 4.35201500

H -0.20711800 -4.01971600 3.33946300

H -2.04960900 -0.53264200 5.06735100

H -0.82084500 -2.67923000 5.34526200

C 0.40683100 -1.79121400 -2.54441000

O 0.24576500 -1.96607400 -1.27478500

O 0.56739400 -0.69458200 -3.10815100

C 0.41702100 -3.08561300 -3.35753900

H 0.44582300 -2.86613600 -4.42740200

H 1.29778500 -3.67935200 -3.08573700

H -0.46686500 -3.68724800 -3.12293500

C -1.77707900 2.41475900 -1.11700800

O -1.45699900 3.55992100 -0.86098700

O -1.42052400 1.74805800 -2.22142700

C -0.52146000 2.38513500 -3.15228400

H -1.10661900 2.96875900 -3.86979600

H 0.00037300 1.56037700 -3.63457300

H 0.17201600 3.04047400 -2.62463200

C -2.87474400 -1.94254400 -0.51592400

C -3.57224000 -3.05742400 -0.02025100

C -2.87835400 -1.70368100 -1.90236600

C -4.25856800 -3.90826900 -0.88784300

H -3.59318900 -3.25187600 1.04663800

C -3.56241700 -2.55785600 -2.76291400

H -2.32531500 -0.85984900 -2.30164800

C -4.25494700 -3.66396700 -2.26104200

H -4.79766100 -4.76160700 -0.48502800

H -3.54607300 -2.36334100 -3.83177700

H -4.78433700 -4.33055800 -2.93662500

**PC**

Pd 0.05092200 0.18078400 -0.16919500

C 2.06305300 0.73839600 -0.08272100

C 1.32298700 1.95535900 -0.03952100

C 1.01075900 2.62705400 1.26268400

C 0.23081900 3.79754200 1.34594000

C 1.47405100 2.06999000 2.47297600

C -0.06075900 4.38200900 2.57855900

H -0.14770200 4.25580900 0.44150400

C 1.17743500 2.65329500 3.70369900

H 2.08230400 1.17148900 2.43498300

C 0.40635500 3.81664200 3.76714200

H -0.66400500 5.28614500 2.60553400

H 1.55829800 2.19993900 4.61584900

H 0.17839400 4.27702700 4.72488700

P -2.28085900 1.01022300 -0.35160800

C -3.41994600 0.31273400 -1.63644000

C -3.15862000 0.79937300 1.26144500

C -2.50985900 2.81535400 -0.72437200

C -4.73165400 0.78711700 -1.81301500

C -2.93519200 -0.66304800 -2.51684200

C -4.36357400 0.10117100 1.41767300

C -2.53181500 1.32996500 2.40476100

C -1.91670700 3.31162600 -1.89832700

C -3.26252600 3.68866200 0.07292000

H -5.11594500 1.57035500 -1.16590300

C -5.54292000 0.27253000 -2.82354900

C -3.74434200 -1.17370600 -3.53598500

H -1.91508900 -1.01685100 -2.41219100

H -4.85330600 -0.34169600 0.55794100

C -4.93939600 -0.04589200 2.68256800

C -3.11613300 1.19214400 3.66317500

H -1.58799100 1.85886200 2.31248800

H -1.31094000 2.65879600 -2.52042200

C -2.08421500 4.64632700 -2.26706800

C -3.41877000 5.02864300 -0.29388300

H -3.72682900 3.32970600 0.98537200

H -6.55608400 0.64709400 -2.94459300

C -5.05142100 -0.71149900 -3.68682300

H -3.34676700 -1.92791600 -4.20999100

H -5.87155000 -0.59529700 2.78438600

C -4.32378600 0.50407600 3.80640700

H -2.62059400 1.62033800 4.53040000

H -1.62674600 5.01118800 -3.18316300

C -2.83317600 5.51130600 -1.46413000

H -4.00363600 5.69239700 0.33797500

H -5.68291700 -1.10591700 -4.47863500

H -4.77643100 0.39184900 4.78825500

H -2.95825400 6.55243800 -1.74981200

C 3.34353300 0.35400000 -0.21819100

C 4.47409100 1.34404800 -0.24133000

C 4.44766200 2.54770200 0.48605200

C 5.60939300 1.08685300 -1.03523500

C 5.50445600 3.45486600 0.41658000

H 3.60285400 2.77280000 1.12456800

C 6.66185600 1.99838800 -1.11043200

H 5.66150200 0.16737600 -1.60876000

C 6.61760300 3.18870800 -0.38292000

H 5.45676000 4.37234800 0.99804600

H 7.51893900 1.77482200 -1.74110400

H 7.44041800 3.89714900 -0.43497800

C 1.39239100 2.72018200 -1.32716500

O 1.28497800 2.24283700 -2.44357900

O 1.68456600 4.03173600 -1.13508900

C 1.89259600 4.79613600 -2.32912500

H 2.72680000 4.38793500 -2.90577100

H 2.12025000 5.80793500 -1.99037000

H 0.99454600 4.79474800 -2.95278600

C 3.75531700 -1.06918100 -0.43504200

C 4.60857800 -1.70985000 0.47900600

C 3.38907200 -1.75695400 -1.59873100

C 5.06161400 -3.00671900 0.24477800

H 4.91933600 -1.18080500 1.37571800

C 3.84536300 -3.05601500 -1.83873900

H 2.76277700 -1.25187200 -2.32587400

C 4.68281800 -3.68498600 -0.91762100

H 5.71378500 -3.48851600 0.96877300

H 3.54725600 -3.57092500 -2.74833600

H 5.04347000 -4.69341900 -1.10460200

P -0.21717100 -2.20321300 0.19506100

C -1.79895700 -2.84799300 0.94376800

C -1.93456300 -3.02668700 2.32915200

C -2.90797000 -3.12412700 0.12530100

C -3.13510800 -3.48216200 2.87715400

H -1.09716500 -2.82513800 2.98861800

C -4.10511000 -3.58527300 0.67408500

H -2.84057500 -2.99353100 -0.94943800

C -4.22320600 -3.76956000 2.05277500

H -3.21294200 -3.61943200 3.95252600

H -4.94423600 -3.80412700 0.01848600

H -5.15389300 -4.13405600 2.47976500

C -0.10149700 -3.24406300 -1.33045400

C -0.41033200 -4.61546300 -1.35810800

C 0.26301000 -2.61464700 -2.53017200

C -0.32568400 -5.33960800 -2.54773300

H -0.73890600 -5.11648900 -0.45260100

C 0.33866100 -3.33691500 -3.72399100

H 0.48079800 -1.54996800 -2.52265500

C 0.05148800 -4.70253600 -3.73305700

H -0.56391400 -6.40007200 -2.55065700

H 0.61957200 -2.82970900 -4.64322300

H 0.11017600 -5.26720800 -4.65988700

C 1.02738000 -2.86876900 1.39437100

C 1.59327300 -4.14867300 1.32250000

C 1.36540800 -2.04166400 2.47862400

C 2.46005100 -4.59696500 2.32109800

H 1.37674600 -4.79672200 0.48080800

C 2.21858900 -2.49734200 3.48451200

H 0.95970000 -1.03478100 2.52894800

C 2.76716300 -3.77896900 3.40894700

H 2.89643800 -5.58954100 2.24504400

H 2.46171900 -1.84641700 4.32038100

H 3.43772700 -4.13353600 4.18739300
